# Photocatalytic α‐Tertiary Amine Synthesis via C−H Alkylation of Unmasked Primary Amines†

**DOI:** 10.1002/anie.202005294

**Published:** 2020-06-11

**Authors:** Alison S. H. Ryder, William B. Cunningham, George Ballantyne, Tom Mules, Anna G. Kinsella, Jacob Turner‐Dore, Catherine M. Alder, Lee J. Edwards, Blandine S. J. McKay, Matthew N. Grayson, Alexander J. Cresswell

**Affiliations:** ^1^ Centre for Sustainable Chemical Technologies University of Bath 1 South, Claverton Down Bath BA2 7AY UK; ^2^ Department of Chemistry University of Bath 1 South, Claverton Down Bath BA2 7AY UK; ^3^ Medicines Design GSK Medicines Research Centre Gunnels Wood Rd Stevenage SG1 2NY UK

**Keywords:** amines, C−H activation, photocatalysis, radicals, spiro compounds

## Abstract

A practical, catalytic entry to α,α,α‐trisubstituted (α‐tertiary) primary amines by C−H functionalisation has long been recognised as a critical gap in the synthetic toolbox. We report a simple and scalable solution to this problem that does not require any in situ protection of the amino group and proceeds with 100 % atom‐economy. Our strategy, which uses an organic photocatalyst in combination with azide ion as a hydrogen atom transfer (HAT) catalyst, provides a direct synthesis of α‐tertiary amines, or their corresponding γ‐lactams. We anticipate that this methodology will inspire new retrosynthetic disconnections for substituted amine derivatives in organic synthesis, and particularly for challenging α‐tertiary primary amines.

## Graphical abstract

**Catalytic strategies** for the α‐C−H functionalisation of primary amines are a major challenge in organic synthesis. A photocatalytic protocol for the α‐C−H alkylation of unprotected primary amines that is amenable to the direct synthesis of α‐tertiary primary amines is reported. This process is readily scalable in continuous flow to provide access to decagram quantities of valuable γ‐lactams and azaspirocycles, for application in drug discovery.

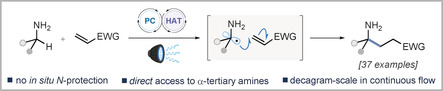

## Introduction

**Figure 1:**
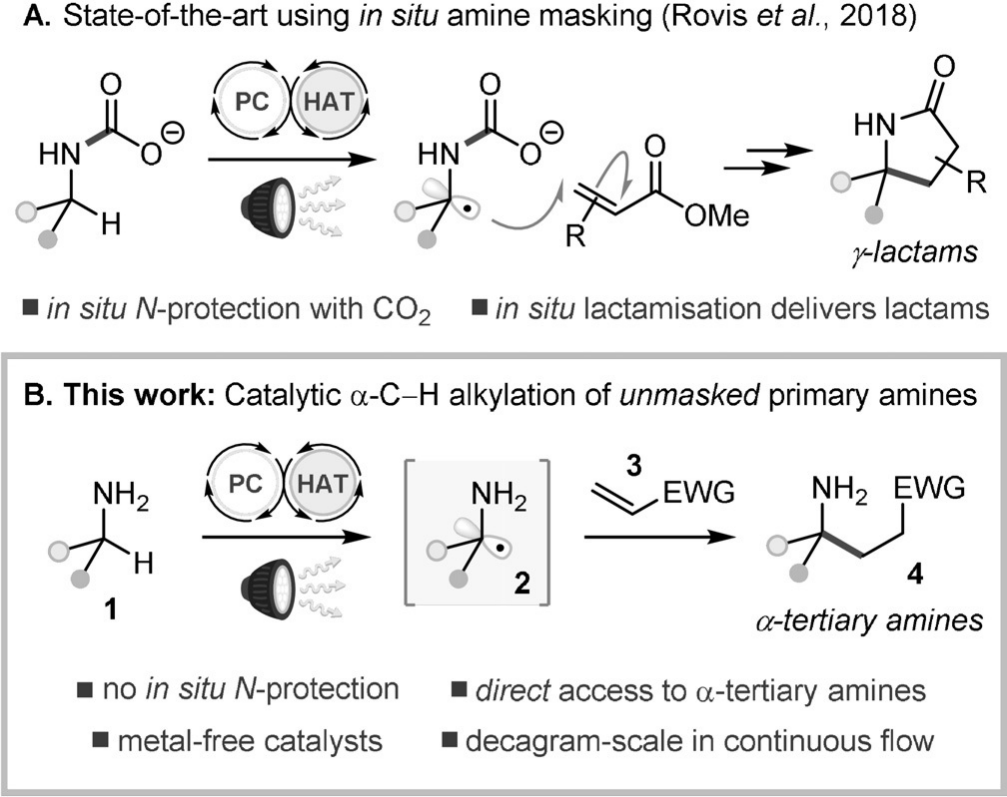
A) Prior art for catalytic α‐C−H alkylation of primary amines; B) This work. EWG=electron‐withdrawing group.

Aliphatic amines and their simple derivatives are pervasive in bioactive molecules, and their centrality in medicinal chemistry is evidenced by their occurrence in over 40 % of drug candidates.^1^ Saturated azacyclic motifs such as piperidines and pyrrolidines^2^—as well as more conformationally‐constrained analogues such as azaspirocycles^3^—are now a mainstay in drug discovery programmes, where their high fraction of saturated carbon (Fsp^3^) can markedly reduce compound attrition rates. Unsurprisingly, the growing demand for functionalised aliphatic amines and saturated azacycles in drug design has continued to spur the development of practical, catalytic methods for their synthesis.^4^ The alkylation of α‐C−H bonds in aliphatic amines provides a powerful alternative to established C−N bond‐forming strategies,^5^ but novel methods for the synthesis of α‐tertiary amines via C−H functionalisation are still urgently needed.^1^ Given that primary alkyl amines offer the greatest potential for diversification, and are prevalent in pharmaceutical compound libraries (e.g., >2700 non‐benzylic primary amines in GSK′s internal chemical inventory), methods for the α‐C−H alkylation of this particular amine class could be transformative. Despite isolated reports of non‐catalytic α‐C−H alkylations of primary aliphatic amines,^6^ including an amine dehydrogenation sequence with stoichiometric quinones,^7^ catalytic protocols to directly access *C*‐alkylated primary amines have proven elusive, especially for non‐benzylic amines.^8^ Indirect methods reliant on *N*‐protection, catalytic C−H alkylation9a, 9b or arylation,9b and subsequent *N*‐deprotection have been realised, but the triflamide or benzamide groups that are required suffer from harsh deprotection protocols. Notably, a recent study from Rovis, Schoenebeck, and co‐workers showed that in situ *N*‐protection of aliphatic primary amines with CO_2_ enables a catalytic α‐C−H alkylation process, leading to γ‐lactam products (Figure 1 A).^10^

The lack of a practical and scalable catalytic entry to α‐tertiary primary amines by C−H functionalisation was recently stressed as a key unsolved problem for synthetic chemistry.1a, 7 To address this challenge, our lab has initiated a research programme on the use of unprotected aliphatic primary amines as formal C‐nucleophiles in catalytic C−C bond‐formation, seeking to avoid N‐protection strategies altogether. With carbon electrophiles as reactants, a critical issue is to outcompete the innate background reactivity of the free amines, leading to *N*‐alkylation, and to steer the reactions towards an unconventional (“umpolung”) *C*‐alkylation. In this work, we report a practical and scalable solution to this problem, based on photoredox catalysis,^11^ for the catalytic generation of unprotected α‐amino radicals^12^ **2** from primary amines **1**, and their interception with electrophilic Michael acceptors **3** to give α‐tertiary amines **4** (Figure 1 B). We also demonstrate that these products can be readily elaborated—in a telescoped process—to *N*‐functionalised α‐tertiary amine derivatives^13^ or pharmacologically‐valuable γ‐lactams.^3^

## Results and Discussion

**Figure 2:**
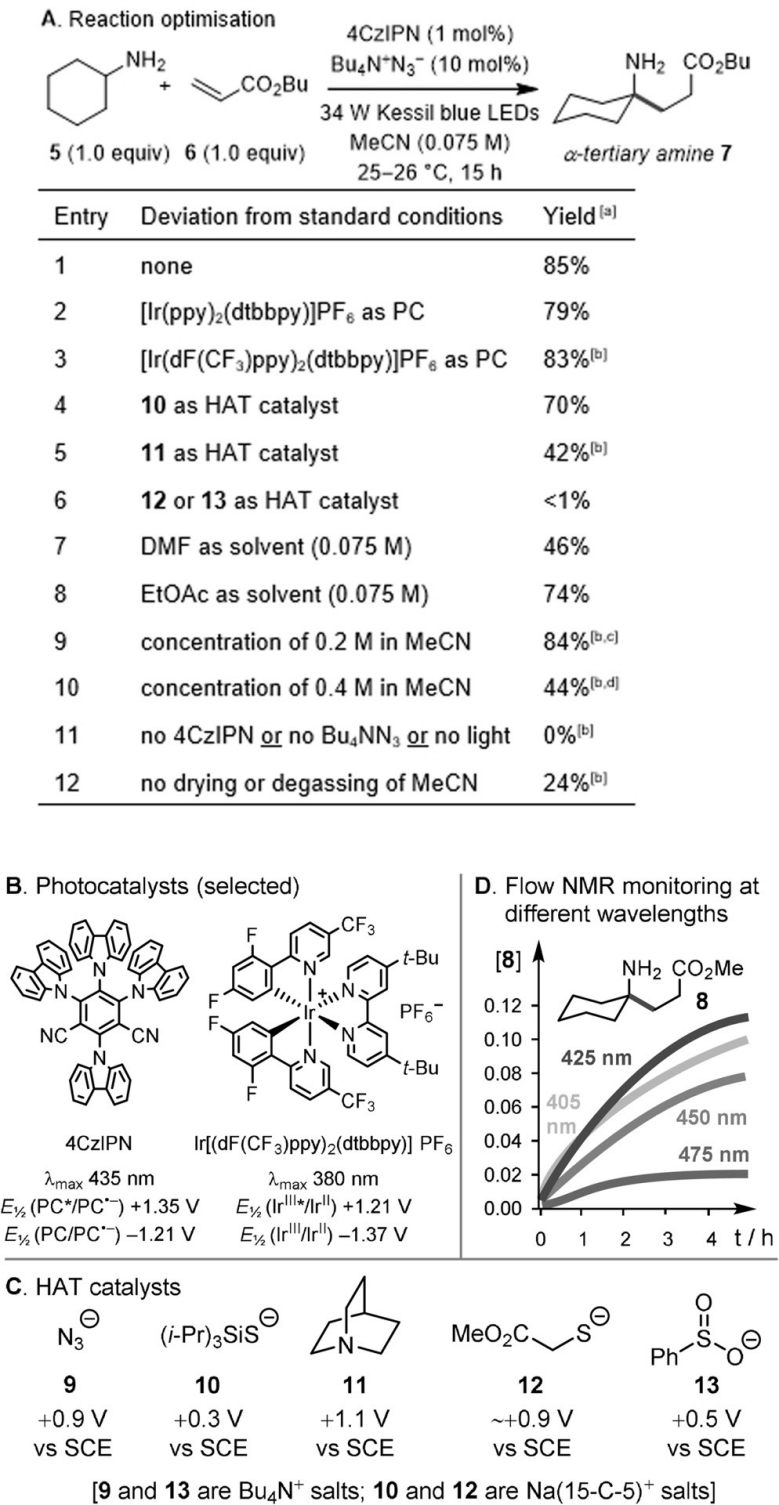
A) Reaction optimisation. [a] Measured by GC (gas chromatography) against dodecane as an internal standard; note that lactamisation occurs under the analysis conditions. [b] Measured by ^1^H NMR against mesitylene as an internal standard. [c] <1 % of the aza‐Michael adduct was formed. [d] 4 % of the aza‐Michael adduct was formed. B) Selected photocatalysts. C) HAT catalysts. D) Concentration‐time plots, at different irradiation wavelengths, for formation of the α‐tertiary amine product **8** (using methyl acrylate).

We began our investigations by using cyclohexylamine **5** (*E*_p/2_=+1.53 V vs. SCE, in MeCN) and butyl acrylate **6** as model substrates (Figure 2 A). By irradiating **5** and **6** in MeCN with 34 W Kessil blue LEDs, in the presence of various photoredox catalysts (PCs) and hydrogen atom transfer (HAT) catalysts (Figure 2 B and C),^14^ we found that α‐C−H alkylation could be cleanly effected in 85 % NMR yield using 4CzIPN^15^ as the photocatalyst (PC) and tetrabutylammonium azide (Bu_4_N^+^N_3_^−^) as the HAT catalyst (*E*_p/2_ of N_3_^−^= +0.87 V vs. SCE in MeCN^16^). Surprisingly, azide ion outperformed both quinuclidine **11** 10 (42 %) and tri(isopropyl)silanethiolate **10** (70 %), despite the well‐established pedigree of these species as HAT catalysts. Control experiments verified that 4CzIPN, visible light, and azide catalyst are all necessary components for reactivity. To determine the optimal wavelength of light to use in the reaction, we tested a series of pseudo‐monochromatic LED light sources (405–475 nm), and used flow NMR spectroscopy^17^ to monitor the reaction (Figure 2 D). A 425 nm light source proved to be optimal, consistent with the absorption maximum of 4CzIPN at *λ*_max_=435 nm. These NMR experiments also revealed that lactamisation proceeds to a negligible extent under the reaction conditions (at 25 °C), such that α‐tertiary amines are the primary products.

**Figure 3:**
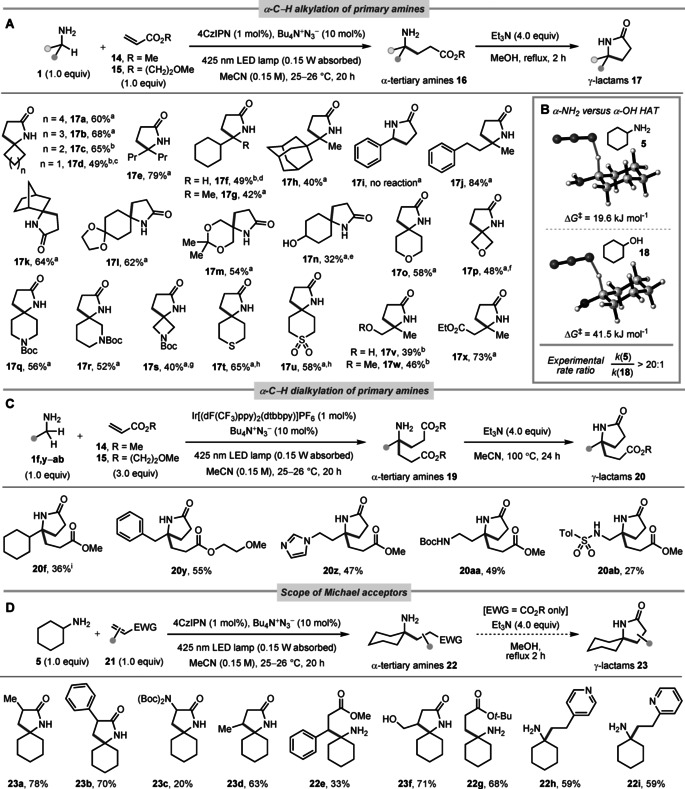
A) Scope for α‐C−H alkylation of primary amines. [a] Used methyl acrylate **14** as the acceptor. [b] Used 2‐methoxyethyl acrylate **15** as the acceptor, and performed the lactamisation step with Et_3_N (4.0 equiv) in MeCN at 100 °C for 24 h. [c] ≈20 % unreacted amine **1 d**. [d] Used 2.0 equiv of amine **1 f**, 1.0 equiv of acrylate **15**, and Ir[(dF(CF_3_)ppy)_2_(dtbbpy)]PF_6_ (1 mol %) as the photocatalyst. [e] A 59:42 ratio of **17 n** to unreacted amine **1 n** was observed. [f] 3.0 equiv of amine **1 p** was used (N.B. 26 % yield with only 1.0 equiv of **1 p**, with >50 % unreacted **1 p**). [g] ≈25 % unreacted amine **1 s**. [h] The amine hydrochloride salt was used and Cs_2_CO_3_ (1.0 equiv) was added. B) Transition states and experimental rate ratio for α‐NH_2_ versus α‐OH C−H abstraction by azidyl radical. C) Scope for α‐C−H dialkylation of primary amines. [i] Used 4CzIPN (1 mol %) as the photocatalyst. [g] Used acrylate **15**. Boc=*tert*‐butoxycarbonyl; Tol=4‐tolyl. D) Scope with respect to the alkene acceptor.

**Figure 4:**
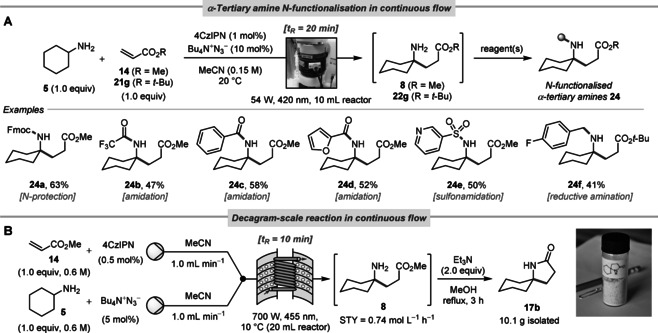
A) Continuous flow synthesis of α‐tertiary primary amine derivatives **24**. Yields reported for products **24** are w.r.t. the γ‐amino ester **8**/**22 g**. Fmoc, fluorenylmethoxycarbonyl. B) Decagram‐scale reaction in continuous flow.

With optimised conditions in hand, we next investigated the scope of the reaction with respect to the amine partner **1** (Figure 3 A). Methyl acrylate **14** [or in some cases 2‐methoxyethyl acrylate **15** 18] was used as the alkylating agent, and a 1:1 stoichiometry of amine:acrylate was employed in all cases (except **1 f** and **1 p**). As the initially‐formed γ‐amino esters **16** are prone to lactamisation on heating, we chose to purposefully convert them to their γ‐lactams **17** in a subsequent lactamisation step, though other manipulations of the amino group are possible (Figure 4 A). Under these generalised conditions, an array of primary amines **1** were assessed, providing C−H alkylated products **17** in yields ranging from 32–84 %. Cyclic primary amines with 5–7‐membered ring sizes delivered the corresponding azaspirocycles (**17 a**–**c**) in 60–68 % yield. Unsurprisingly, cyclobutyl amine **1 d** proved more challenging—a consequence of the stronger α‐C−H bond (see Supporting Information)—although the alkylated product **17 d** could still be isolated in 49 % yield. A range of acyclic amines (**17 e**–**j**) were also surveyed, and we found that tertiary (**1 f**,**g**) or quaternary carbons (**1 h**) β‐ to the amino group are tolerated. For α‐monosubstituted amine **1 f**, a mixture of α‐mono and α‐dialkylated products was evident on using a 1:1 amine:acrylate stoichiometry, due to overalkylation of the intermediate γ‐amino ester **16 f** (see Supporting Information). However, after some re‐optimisation (i.e., photocatalyst, amine equiv), α‐monosubstituted γ‐lactam **17 f** could be isolated in 49 % yield. One notable limitation is the lack of reactivity of benzylamine **1 i**, which may arise from the increased stability of the α‐amino radical, rendering the addition step to the acrylate reversible. That said, the presence of benzylic hydrogens elsewhere in the molecule poses no issue, as evidenced by the successful α‐alkylation of amine **1 j**. Diastereoselective α‐alkylation of norbornyl amine **1 k** also proved possible, with the radical intercepting the acrylate species on the expected *exo* face. We also tested functionalised amines containing acetal (**1 l**,**m**), hydroxyl (**1 n**,**v**), ether (**1 o**,**p**,**w**), carbamate (**1 q**–**s**), thioether (**1 t**), sulfone (**1 u**), and ester moieties (**1 x**). In several cases, competitive HAT at other “hydridic” C−H bonds is thought to be operative, including tertiary C−H (**17 f**–**h**) and C−H bonds α‐ to hydroxyl (**17 n**,**v**), ether (**17 m**,**o**,**p**,**w**), and carbamate moieties (**17 q**–**s**). Though all of these C−H bonds are generally stronger or less “hydridic” than those α‐ to the unprotected amine, statistical effects (i.e., relative number of C−H bonds) and the impact of reaction conversion on kinetic partitioning will influence this competition. Isolation of the minor by‐products in these cases proved fruitless, but the ≈3.6 ppm region in the crude NMR spectra did evidence complex mixtures of minor (<5 %) singlets (i.e., methyl ester‐containing by‐products), supportive of acrylate addition to sites other than the amine α‐C−H bond. To further our understanding, we attempted to quantify the relative ease of α‐C−H abstraction by azidyl radical (N_3_^.^) from primary amines versus alcohols, as a representative example. DFT calculations using the M06‐2X functional indicate that the barrier to HAT is approximately doubled from cyclohexylamine **5** to cyclohexanol **18**, and an intermolecular competition experiment between **5** and **18** gave a relative rate ratio of >20:1 (Figure 3 B). In a standalone experiment, cyclohexanol **18** itself was α‐C−H alkylated in only 12 % yield under our conditions, with the remaining mass balance being mainly unreacted **18** (see Supporting Information). Despite this low reactivity of alcohols, we observed incomplete conversion in the case of 4‐hydroxycyclohexylamine **1 n** (i.e., 59:42 ratio of **17 n** to unreacted **1 n**), which may be due to intramolecular hydrogen‐bonding^19^ in the *syn*‐diastereomer, raising the barrier to HAT. In some other cases, such as the 4‐membered ring substrates **1 d**,**p**,**s** (bearing α‐C−H bonds strengthened by ring strain), incomplete conversion of the amine was also primarily responsible for the lower (<50 %) isolated yields, as opposed to extensive by‐product formation. Finally, a robustness screen^20^ with 15 different functional group additives was performed, including ketones, alkynes, bromoarenes, nitriles, amides, benzofurans, imidazoles, and pyridines (see Supporting Information), though it should be qualified that these results are only strictly diagnostic of intermolecular competitions.

We next sought to access the prospect of an unprecedented α‐C−H *dialkylation* of primary amines **1 f,y**–**ab** bearing two α‐C−H bonds, to generate α‐tertiary amines **19** directly (Figure 3 C). This transformation proved more challenging—possibly due to side‐reactions (e.g., telomerisation, aza‐Michael) promoted by the higher acrylate concentration—but dialkylated γ‐lactams **20** could still be isolated in yields of 27–55 %.^21^ The scope of the α‐C−H monoalkylation process with respect to the alkene acceptor (**21**) was also evaluated (Figure 3 D). Acrylates **21 a**–**e** bearing α‐ or β‐substituents, including methyl, phenyl, and ‐N(Boc)_2_ were all tolerated, as was an unsaturated γ‐lactone acceptor (giving **23 f**). The use of *tert*‐butyl acrylate (**21 g**) led to a γ‐amino ester product (**22 g**) that proved substantially more resistant to lactamisation. Preliminary examination of non‐acrylate partners was also carried out, with vinyl pyridines **21 h**,**i** successfully delivering α‐alkylated products. No reactivity was observed with unactivated alkenes (i.e., 1‐hexene) as the acceptor.

To validate the fact that our chemistry is useful for the rapid and modular assembly of α‐tertiary amine derivatives, we next effected a series of *N*‐functionalisations of the γ‐amino ester products, *in lieu* of lactamisation (Figure 4 A). In order to reduce reaction times and facilitate scale‐up, we conducted the reactions in continuous flow,^22^ using a Vapourtec UV‐150 reactor equipped with a 54 W output 420 nm LED lamp. With cyclohexylamine **5** as a representative amine, this led to a straightforward preparation of a series of α‐tertiary amine derivatives **24 a**–**f**. To assess the scalability of our α‐C−H alkylation process, we ran the reaction of cyclohexylamine **5** with methyl acrylate **14** on a decagram‐scale in continuous flow, using a prototype of the recently commercialised Uniqsis PhotoSyn reactor (Figure 4 B). Although the centre wavelength of 455 nm was sub‐optimal for our purposes, a space‐time yield (STY) of 0.74 mol L^−1^ h^−1^ for γ‐amino ester **8** still proved possible, and we were able to isolate 10.1 g of product **17 b** from a single 5.3 h run, post‐lactamisation. Notably, this chemistry provides a scalable access to spirocyclic pyrrolidine building blocks for drug discovery that outcompetes the current most practical synthetic routes for process‐scale work (5–7 steps).3a

**Figure 5:**
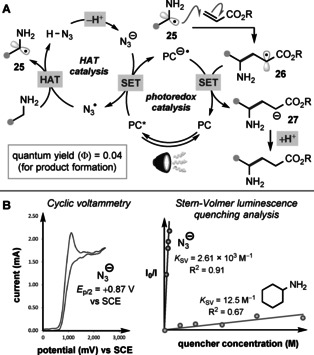
A) Proposed catalytic mechanism.B) Evidence for azide ion as reductive quencher.

Our proposed catalytic cycle for the α‐C−H alkylation process is outlined in Figure 5 A. Initial oxidation of azide ion (*E*_p/2_ of N_3_^−^=+0.87 V vs. SCE, in MeCN) by the photoexcited 4CzIPN [*E*_1/2_ (PC*/PC^.−^)=+1.35 V vs. SCE] generates the azidyl radical, N_3_^.^—a potent oxidant that is capable of hydrogen atom abstraction even from unactivated alkanes.^23^ This reductive quenching step is supported by cyclic voltammetry and Stern–Volmer luminescence quenching experiments (Figure 5 B).^24^ Subsequent HAT from the relatively weak α‐C−H bond of the primary amine (BDE=89–91±2 kcal mol^−1^)^25^, ^26^ is thought to occur selectively, possibly augmented by a polarity‐matching effect between the electrophilic N_3_^.^ radical and the “hydridic” C−H bond.4d The resultant α‐amino radical **25** can undergo a rapid and polarity‐matched addition to the acrylate acceptor to give an α‐carboxy stabilised radical **26** [*E*_1/2_≈−0.63 V vs. SCE^27^]. Reduction of this radical to the corresponding enolate **27** (p*K*_aH_≈24 in H_2_O) by the [4CzIPN]^.−^ radical anion (*E*_1/2_=−1.21 V vs. SCE, in MeCN) is presumably followed by proton transfer from HN_3_ (p*K*_a_=4.72 in H_2_O) to give the γ‐amino ester product and regenerate the azide ion. Alternatively, a chain process involving direct and polarity‐matched HAT from the primary amine (α‐C−H BDE≈90 kcal mol^−1^)^26^ to the α‐carboxy radical **26** (BDE of α‐CO_2_Me C−H ≈96 kcal mol^−1^]^26^ is plausible.6c, 23a However, the low quantum yield for product formation (*Φ*=0.04) rules out the presence of efficient chain processes, although it does not exclude the possibility of an inefficient photoredox process followed by a short radical chain, where quantum yields can be <1.0.

## Conclusion

In summary, we have developed the first visible‐light photocatalysed α‐C−H alkylation of primary aliphatic amines with electrophilic alkenes that does not rely on in situ masking of the amino group's intrinsic reactivity. Our dual catalytic approach, which uses azide ion as a HAT catalyst, is amenable to the decagram‐scale preparation of hitherto difficult‐to‐access α‐tertiary amines and aza(spiro)cyclic building blocks.3a We anticipate that this technology will open up new retrosynthetic strategies for the disconnection of substituted amine derivatives in organic synthesis, and find immediate application in conventional and fragment‐based lead identification programmes.

## Conflict of interest

The authors declare no conflict of interest.

## Supporting information

As a service to our authors and readers, this journal provides supporting information supplied by the authors. Such materials are peer reviewed and may be re‐organized for online delivery, but are not copy‐edited or typeset. Technical support issues arising from supporting information (other than missing files) should be addressed to the authors.

Supplementary
