# Supplementary material for: Photocatalytic α‐Tertiary Amine Synthesis via C−H Alkylation of Unmasked Primary Amines
Source: Angew Chem Int Ed Engl. 2020 Jun 11;59(35):14986–91. doi: 10.1002/anie.202005294 (PMC7496683; doi:10.1002/anie.202005294)
Supplement: Supplementary file 1 — Supplementary [file ANIE-59-14986-s001.pdf]

Angewandte Chemie
Eine Zeitschrift der Gesellschaft Deutscher Chemiker

Supporting Information

Photocatalytic α-Tertiary Amine Synthesis via C–H Alkylation of Unmasked Primary Amines**

*Alison S. H. Ryder, William B. Cunningham, George Ballantyne, Tom Mules, Anna G. Kinsella, Jacob Turner-Dore, Catherine M. Alder, Lee J. Edwards, Blandine S. J. McKay, Matthew N. Grayson, and Alexander J. Cresswell**

anie_202005294_sm_miscellaneous_information.pdf

A. General Experimental ***S3***

B. General Procedures ***S11***

C. Preparation of Starting Materials & Authentic Samples *S13*

C.1. Literature Preparations *S13*

C.2. Preparation of Photocatalyst *S13*

C.3. Preparation of HAT Catalysts *S14*

C.4. Preparation of Authentic Samples *S16*

D. Reaction Optimisation *S20*

D.1. Measurement of GC Relative Response Factors *S20*

D.2. Screening of Reaction Conditions *S24*

D.3. Effect of Light Source: Reaction Monitoring by Flow NMR *S26*

E. Reaction Generality *S27*

E.1. Scope of α-C–H Alkylation with α-Disubstituted Primary Amines *S27*

E.2. Challenges with α-Monosubstituted Primary Amines *S53*

E.3. Robustness Screen with Functional Group Additives *S64*

E.4. Scope of α-C–H Dialkylation of α-Monosubstituted Primary Amines *S65*

E.5. Scope of Michael Acceptors *S71*

E.6. Unsuccessful Substrates *S82*

F. Synthesis of α-Tertiary Amine Derivatives 24a–f in Flow *S83*

F.1. Optimisation on Vapourtec UV-150 Photoreactor *S83*

F.2. Synthesis of α-Tertiary Amine Derivatives *S87*

G. Scale-up of α-C–H Alkylation in Flow *S98*

G.1. Gram-Scale Reaction on Vapourtec UV-150 Reactor *S98*

G.2. Optimisation on Uniqsis PhotoSyn Reactor *S99*

G.3. Decagram-Scale Reaction on Uniqsis PhotoSyn Reactor *S108*

H. Mechanistic Studies *S111*

H.1. Reactivity of Cyclohexanol ***18*** *towards α-C–H Alkylation* *S111*

H.2. Cyclic Voltammetry (CV) Measurements *S112*

H.3. Stern-Volmer Luminescence Quenching Analysis *S114*

H.4. Quantum Yield Measurement *S117*

H.5. Computational Details *S120*

I. ^{1}H and ^{13}C{^{1}H} NMR Spectra *S130*

J. References and Notes *S184*

A. General Experimental

General Setup: Procedures employing oxygen- and/or moisture-sensitive materials were performed with anhydrous solvents (*vide infra*) using standard inert atmosphere techniques (atmosphere of anhydrous nitrogen or argon). Room temperature (rt) typically ranged between 20–25 °C, depending on the time of day. "Brine" refers to a saturated solution of sodium chloride in H_2O. Spiral evaporation, where stated, was performed on a DrySyn Spiral Evaporator.

Photoreactors: All batch photoreactions were conducted in commercially-available EvoluChem PhotoRedOx Box reactors purchased from HepatoChem Inc. (100 Cummings Center, Suite 451C, Beverly, MA 01915 USA).

Light Sources: The light sources employed in this work are shown in Table S1, with the relevant spectra given in Figs. S1 and S2.

**Supplier**	**Light Source**	**λ_{max} (nm)**	**LED**	**Beam Angle (°)**	**Irradiance[a] (mW cm^{-2})**
HepatoChem	Kessil H150-Blue	463,424[b]	–	40	22
HepatoChem	EvoluChem 18 W	405	LG	25	28
HepatoChem	EvoluChem 18 W	425	EPILED	25	33
HepatoChem	EvoluChem 18 W	450	CREE XPE	25	34
HepatoChem	EvoluChem 18 W	475	CREE XPE	25	23

Table S1. LED light sources used in our laboratory. [a] Average irradiance measured in the PhotoRedOx Box reactor using an ILT spectroradiometer 550 [reported by HepatoChem Inc.; see: https://www.hepatochem.com/photochemistry/led-evoluchem/]. [b] Measured in our lab.

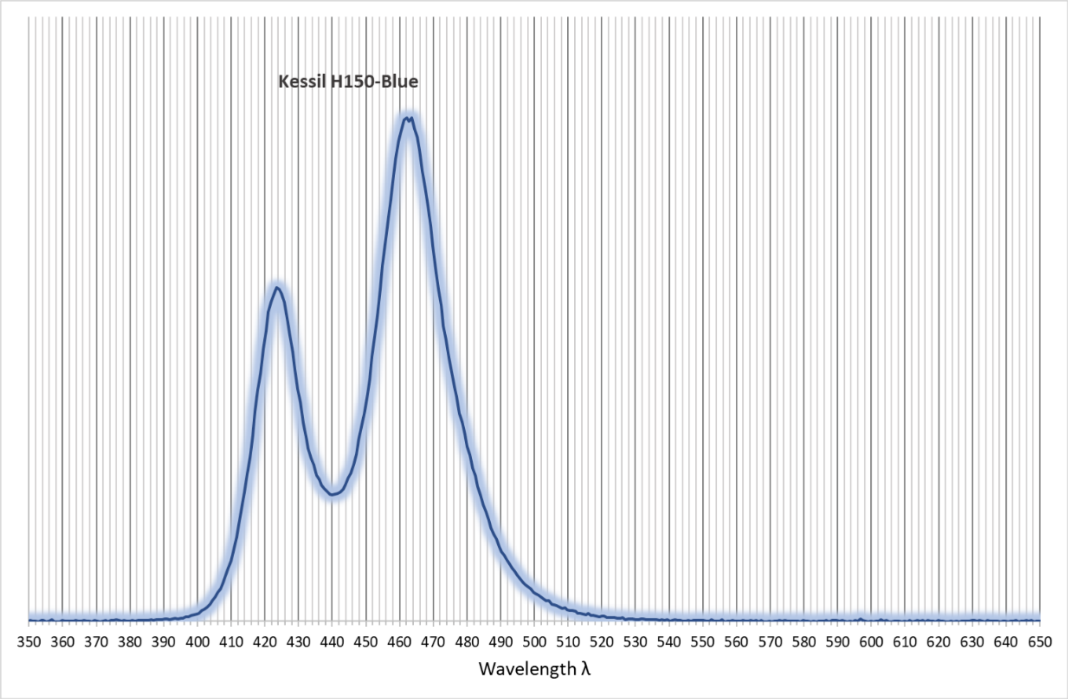

Fig. S1. Spectrum of the Kessil H150-Blue lamp used in our laboratory.

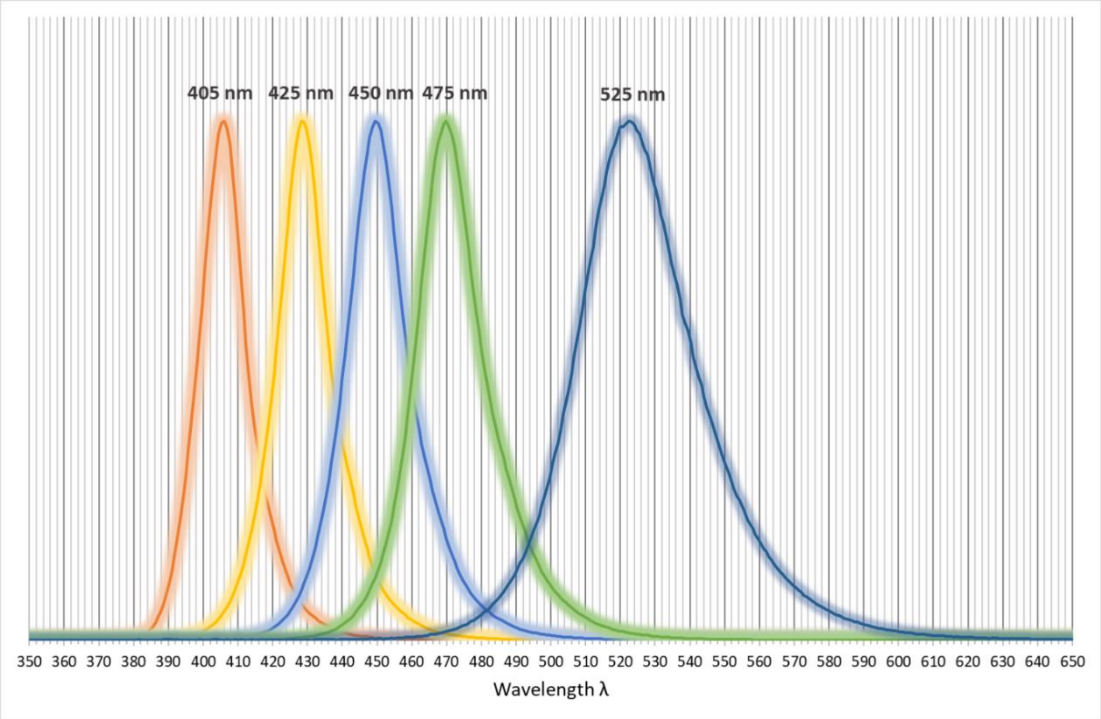

Fig. S2. Spectra for each of the EvoluChem LED lamps used in our laboratory (with intensities arbitrarily normalised to the same value).

NMR Spectroscopy: ^{1}H and ^{13}C{^{1}H} spectra were recorded at 300/500 MHz and 126 MHz, respectively, using a Bruker Avance spectrometer (300 MHz) or a Agilent ProPulse spectrometer equipped with a OneNMR probe (500 MHz). Spectra were recorded at 295 K (22 °C) on the Bruker instrument and 298 K (25 °C) on the Agilent instrument, unless stated otherwise; ^{1}H and ^{13}C{^{1}H} NMR spectra were referenced to residual solvent peaks; chemical shifts are reported in parts per million (ppm) relative to residual chloroform (δ = 7.26 ppm, ^{1}H; 77.16 ppm, ^{13}C). All ^{13}C{^{1}H} resonances are assumed to be singlets, unless stated otherwise. Coupling constants, *J*, reported in Hertz (Hz), were calculated using *Mestrenova* 9.0 to the nearest 0.1 Hz. The following abbreviations (and their combinations) are used to label the multiplicities: app (apparent), br (broad), s (singlet), d (doublet), t (triplet), q (quartet), sept (septet), and m (multiplet). ^{1}H and ^{13}C{^{1}H} assignments for novel compounds are corroborated through 2D (COSY, HSQC, HMBC).

Flow NMR Spectroscopy: A peristaltic pump (Vapourtec SF-10) was used to circulate the mixture around the system to an InsightMR flow tube (Bruker) located within the spectrometer (Bruker Avance III + 500 MHz Ultrashield equipped with a Prodigy cryoprobe). In order to minimise the delay time between a change occurring in the reaction vessel and the arrival of the sample to the spectrometer for detection it is desirable to ensure that the volume of the tubing connecting the reaction vessel to the spectrometer is minimised, therefore narrow diameter polyetheretherketone (PEEK) tubing (0.762 mm i.d., Upchurch Scientific) was used. The PEEK tubing offers high chemical and mechanical stability (pH 0–14, –50–100 °C, >300 bar) along with good flexibility and low gas permeability. All other connections were made using standard HPLC-type PEEK connectors (Upchurch Scientific). All equipment was positioned inside a fume hood, located approximately 1 m from the shielded NMR spectrometer, without experiencing any adverse magnetic effects.

Data acquisition was performed without lock and with shimming performed using automated ^{1}H shimming routines, followed by manual fine adjustment. Data processing was performed using commercially available software. Solvent suppression of the MeCN resonance using a WET pulse sequence with a shaped pulse and low power ^{13}C broadband decoupling during acquisition was carried out with the Bruker pulse program “wetdc” (using a standard LC-NMR automated acquisition program, “au_lc1d” that first acquires a scan prior to starting the experiment to identify and subsequently suppress the desired number of solvent peaks areas throughout the experiment).

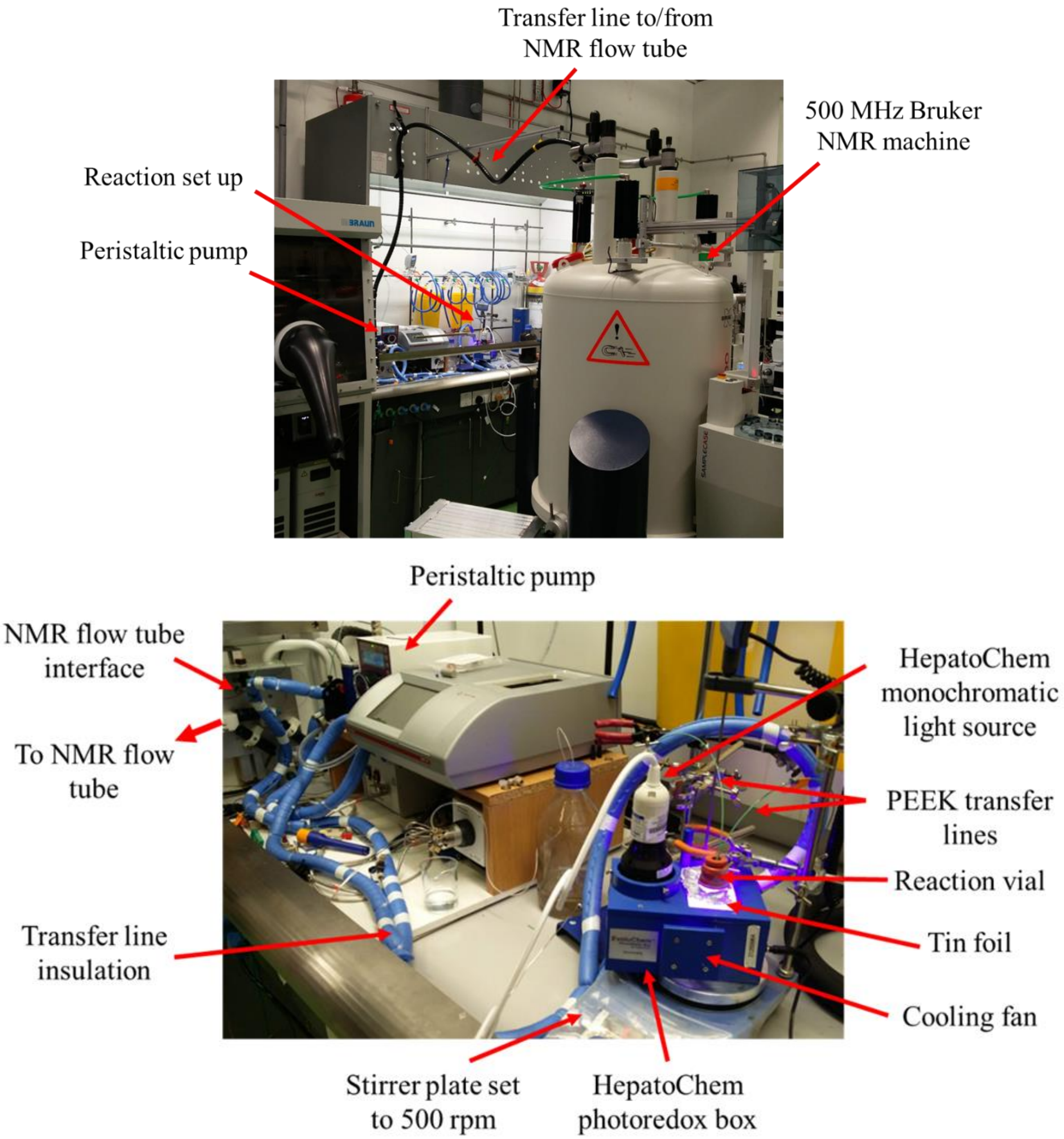

Fig. S3. Photographs of the set-up used for flow NMR spectroscopy.

A 20-mL scintillation vial equipped with a stirrer bar was flame-dried and transferred to a nitrogen-filled purge box whilst still hot, then was allowed to cool under the N_2 atmosphere. The vial was then charged with stock solutions of 4CzIPN (2.28 mM in MeCN, 6.58 mL, 15.0 µmol, 1 mol%) and tetrabutylammonium azide (70.3 mM in MeCN, 2.13 mL, 150 µmol, 10 mol%), and made up to a total volume of 10.0 mL by addition of MeCN (1.29 mL). Cyclohexylamine **5** (174 µL, 151 mg, 1.50 mmol, 1.0 equiv) was then transferred into the vial by microlitre syringe. Finally, methyl acrylate **14** (138 µL, 131 mg, 1.50 mmol, 1.0 equiv) was added, and the vial was sealed using an up-turned B24 rubber septa. It was then removed from the purge box, wrapped in tin foil before being transferred to the photoreactor.

The FlowNMR apparatus was purged with MeCN and then argon. The apparatus was connected to the reaction vessel by pushing the transfer lines through the septa, and the pump was started. The flow tube was then inserted into the spectrometer and automated shimming and tuning routines were performed. Best results were obtained if automated shimming and tuning was performed on static samples; however, acceptable results were still obtained in flow. Frequency lock was switched off when using non-deuterated solvents, and shimming was performed on proton peaks. Manual fine tuning of X and Y shims was often required to obtain a good peak line width. Spectra of the reagents were recorded without flow and again at the flow rate desired for the reaction. Comparison of the integral areas of the peaks in each spectrum was used to calculate a correction factor for each reagent peak (I = peak integral, α = correction factor).[1]

$$I_{\text{corrected}} = \alpha I \qquad \text{(where } \alpha = \ I_{\text{static}}/I_{\text{flow}}\text{)}$$

With the sample flowing, data acquisition was started using dedicated InsightMR reaction monitoring software, with solvent-suppressed ^{1}H spectra recorded at specified time intervals. To start the reaction, the light source was switched on. At the end of the reaction, or if intermediates of interest were observed, additional spectra were recorded with and without flow, and correction factors were calculated for the intermediate or product peaks, which were applied to each spectrum to give the final peak areas for calculation of species concentration and plotting of kinetic data. Concentrations of species were determined by peak integrals referenced to tetrabutylammonium azide.

Infrared Spectroscopy: Infrared (IR) spectra of neat compounds were recorded over the range 4000–650 cm^{-1} using a PerkinElmer Spectrum 100 ATR-FTIR spectrometer. Peaks are reported in cm^{-1} with indicated relative intensities: s (strong, 0–33% T); m (medium, 34–66% T), w (weak, 67–100% T), and br (broad).

Mass Spectrometry: Electrospray ionisation (ESI^+) spectra were recorded on an Agilent Electrospray Quadrupole Time-of-Flight mass spectrometer (ESI-QTOF). Data are reported in the form of m/z (intensity relative to the base peak = 100).

Melting Points: Uncorrected melting points (mp) were determined on a Stanford Research Systems OptiMelt automated capillary melting point apparatus in open capillary tubes.

Chromatography: Analytical thin-layer chromatography was performed on Merck silica gel 60 F_{254} aluminium-backed plates. Visualisation was accomplished with UV light (254 nm), iodine (I_2) on silica, and/or aqueous basic potassium permanganate ($KMnO_4$) solution. Automated flash column chromatography (normal and reversed phase) was performed using a CombiFlash NextGen 300+ System equipped with UV and ELSD detectors. Manual flash column chromatography was performed using high-purity grade silica gel, pore size 60 Å, 200-400 mesh particle size (Sigma-Aldrich, Cat. No. 288594) or silica gel 90 C_{18}-reversed phase (Sigma-Aldrich, Cat. No. 60757), as specified.

Gas Chromatography: Analytical capillary gas chromatography (GC) was performed using an Agilent 7890B gas chromatograph fitted with a flame ionisation detector (He carrier gas). Injections were made onto an Agilent DB-FFAP column (30 m × 250 mm, 0.25 micron, 40–250 °C temperature limits). The parameters employed for all runs are outlined in Table S2.

**Inlet type**	Split	**Column flow**	1.2 mL / min
**Split ratio**	5 : 1	**Column pressure**	18.715 psi
**Split flow**	6 mL / min	**Average velocity**	25.918 cm / sec
**Inlet temp.**	250 °C	**Hold-up time**	1.9291 min
**Total flow**	10.2 mL/mon	**Post run**	1.5676 mL / min
**Septum purge flow**	3 mL / min	**Run time**	18.5 min
**Total pressure**	20.3 psi	**Injection volume**	1 µL
**Oven temp. (initial)**	80 °C	**Plunger speed**	Fast
**Oven temp. (final)**	250 °C	**Detector**	FID
**Oven Temp. hold**	3 min	**Detector temp.**	300 °C
**Oven temp. ramp**	20 °C / min	**He make-up flow**	5 mL / min
**Oven run time**	7 min	**Carrier gas**	2.1 mL / min
**Oven equilb. time**	0.5 min		

Table S2. Parameters employed for gas chromatography (GC) analysis.

Solvents: Reaction solvents tetrahydrofuran (THF) and acetonitrile (MeCN) were dried by percolation through columns packed with neutral alumina under a positive pressure of nitrogen. Dimethylformamide (DMF) (extra dry, with molecular sieves), ethanol (EtOH), ethyl acetate (EtOAc), *tert*-amyl alcohol (*t*-AmOH), and toluene were used as received. Solvents for filtration, transfers, chromatography, and recrystallisation, including acetonitrile (MeCN), chloroform ($CHCl_3$), dichloromethane (CH_2Cl_2), diethyl ether (Et_2O), ethanol (EtOH), ethyl acetate (EtOAc), hexane, methanol (MeOH), and 40-60° petroleum ether (petrol) were used as received.

Chemicals: Sodium hydride (NaH) was washed with dry hexane to remove the mineral oil, and then stored in a glovebox. The following chemicals were purchased from commercial suppliers and used as received (with quantity calculations taking into account the quoted purity): $[Ir(ppy)_2(dtbbpy)]PF_6$, $[Ir(dF(CF_3)ppy)_2(dtbbpy)]PF_6$, carbazole, tetrafluorophthalonitrile, tri(isopropyl)silanethiol, 15-crown-5, methyl thioglycolate, sodium benzenesulfinate, tetrabutylammonium hydroxide hydrate ($Bu_4NOH•30H_2O$), cyclohexylamine, methyl acrylate, di-*tert*-butyl peroxide, butyl acrylate, *tert*-butyl acrylate, 9-fluorenylmethoxycarbonyl chloride, trifluoroacetic anhydride, triethylamine (Et_3N), benzoyl chloride, furoyl chloride, pyridine-3-sulfonyl chloride, 4-fluorobenzaldehyde, acetic acid (AcOH), sodium triacetoxyborohydride, cycloheptylamine, cyclopentylamine, 2-methoxyethyl acrylate, cyclobutylamine, heptan-4-amine, cyclohexanemethylamine, (*RS*)-1-cyclohexylethylamine, (*RS*)-1-(adamantan-1-yl)ethan-1-amine [Rimantidine], (*RS*)-4-phenylbutan-2-amine, *exo*-2-aminonorbornane , 1,4-dioxaspiro[4.5]decan-8-amine, 2,2-dimethyl-1,3-dioxan-5-amine, 4-aminocyclohexanol, 4-aminotetrahydrofuran, oxetan-3-amine, *tert*-butyl 4-aminopiperidine-1-carboxylate, *tert*-butyl (*RS*)-3-aminopiperidine-1-carboxylate, *tert*-butyl 3-aminoazetidine-1-carboxylate, tetrahydro-2*H*-thiopyran-4-amine, caesium carbonate (Cs_2CO_3), 4-aminotetrahydro-2*H*-thiopyran 1,1-dioxide hydrochloride, (*RS*)-2-amino-1-propanol, (*RS*)-1-methoxy-2-propylamine, ethyl (*RS*)-3-aminobutanoate, *N*-tosylethylenediamine, *tert*-butyl (3-aminopropyl)carbamate, (trimethylsilyl)methanamine, 3,5-dimethyl-4-isoxazolepropanamine, 3-(1*H*-imidazol-1-yl)propan-1-amine, 3-(4-methylthiazol-2-yl)propan-1-amine, methyl methacrylate, methyl crotonate, methyl cinnamate, furan-2(5*H*)-one, 4-vinylpyridine, 2-vinylpyridine, tetrabutylammonium chloride, sodium azide, tetrabutylammonium tetrafluoroborate.

B. General Procedures

General Procedure 1 for α-C–H monoalkylation of primary amines: A 20-mL scintillation vial equipped with a stirrer bar was flame-dried and transferred to a nitrogen-filled purge box whilst still hot, then was allowed to cool under the N_2 atmosphere.* In the case of solid or viscous oil amine substrates, the requisite amine **1** (0.45 mmol for 1.0 equiv) was then weighed into the empty vial at this point, and the stirrer bar replaced. The vial was then charged with stock solutions of the requisite photocatalyst [4CzIPN (2.28 mM in MeCN, 1.97 mL, 4.5 µmol, 1 mol%) or Ir[(dF(CF_3)ppy)$_2$(dtbbpy)]PF_6 (2.23 mM in MeCN, 2.02 mL, 4.5 µmol, 1 mol%)] and tetrabutylammonium azide** (70.3 mM in MeCN, 640 µL, 45 µmol, 10 mol%), and made up to a total volume of 3.0 mL by addition of MeCN. For liquid amines, the requisite amine **1** (0.45 mmol for 1.0 equiv) was then transferred into the vial by microlitre syringe at this point. Finally, the requisite Michael acceptor **14**, **15**, or **21** (0.45 mmol, 1.0 equiv) was added, and the vial was sealed using an up-turned B24 rubber septa. It was then removed from the purge box and transferred to a photoreactor, and irradiated (with stirring) for 20 h at 425 nm (for both photocatalysts). Fan cooling was used to maintain an external temperature of 25–26 °C, which matched the internal reaction temperature at all stages of the reaction. Following irradiation, the reaction mixture was concentrated *in vacuo*.

Modification when employing primary amine hydrochloride salts: As for *General Procedure 1*, but the requisite amine hydrochloride salt **1**•HCl (0.45 mmol, 1.0 equiv) was dissolved in MeCN (780 µL) and stirred with Cs_2CO_3 (158 mg, 0.45 mmol, 1.0 equiv) for 15 min in the nitrogen-filled purge box. The photocatalyst, tetrabutylammonium azide, and Michael acceptor **14**, **15**, or **21** were then added as above, but no additional MeCN. In this case, the total solvent volume is 3.39 mL, corresponding to a concentration (w.r.t. amine) of 0.13 M.

General Procedure 2 for α-C–H dialkylation of primary amines: A 20-mL scintillation vial equipped with a stirrer bar was flame-dried and transferred to a nitrogen-filled purge box whilst still hot, then was allowed to cool under the N_2 atmosphere.* In the case of solid or viscous oil amine substrates, the requisite amine **1** (0.45 mmol, 1.0 equiv) was then weighed into the empty vial at this point, and the stirrer bar replaced. The vial was then charged with stock solutions of Ir[(dF(CF_3)ppy)$_2$(dtbbpy)]PF_6 (2.23 mM in MeCN, 2.02 mL, 4.5 µmol, 1 mol%) and tetrabutylammonium azide** (70.3 mM in MeCN, 640 µL, 45 µmol, 10 mol%), and made up to a total volume of 3.0 mL by addition of MeCN. For liquid amines, the requisite amine **1** (0.45 mmol,

1.0 equiv) was then transferred into the vial by microlitre syringe at this point. Finally, the requisite acrylate acceptor **14** or **15** (1.35 mmol, 3.0 equiv) was added, and the vial was sealed using an up-turned B24 rubber septa. It was then removed from the purge box and transferred to a photoreactor, and irradiated (with stirring) for 20 h at 425 nm. Fan cooling was used to maintain an external temperature of 25–26 °C, which matched the internal reaction temperature at all stages of the reaction. Following irradiation, the reaction mixture was concentrated *in vacuo*.

* The reaction is relatively sensitive to air and moisture. Using undried glassware, benchtop MeCN, and taking no precautions whatsoever to exclude air, the GC/NMR yield of compound **17b** was reduced from 85% to 24%.

** Tetrabutylammonium azide is extremely hygroscopic. It can however be conveniently prepared *in situ* by stirring Bu_4NCl with NaCl in MeCN, followed by filtration of NaCl (see section G.3.1). We found that the commercial material (Sigma-Aldrich) is contaminated with NaCl, such that dissolution in MeCN is visibly incomplete, leading to a slightly cloudy solution. Upon standing, the NaCl residues will settle and the clear supernatant solution can be used.

C. Preparation of Starting Materials & Authentic Samples

C.1. Literature Preparations

The following compounds were prepared *via* literature procedures: methyl 2-phenylacrylate **21b**,[2] methyl 2-[di(*tert*-butoxycarbonyl)amino]prop-2-enoate **21c**.[3]

C.2. Preparation of Photocatalyst

Preparation of 2,4,5,6-tetra(9*H*-carbazol-9-yl)isophthalonitrile (4CzIPN)

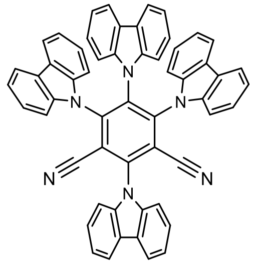

In an argon-filled glove box, NaH (washed with hexane, 180 mg, 7.50 mmol, 7.5 equiv) was added to an oven-dried, 3-necked, 50-mL, round-bottomed flask equipped with a stirrer bar, and the necks of the flask were all sealed with rubber septa. The flask was then removed from the glove box and connected to a N_2 line *via* an inlet needle. Dry THF (20 mL) was added *via* syringe and the mixture was cooled in an ice/water bath. Carbazole (836 mg, 5.00 mmol, 5.0 equiv) was then added portionwise under a stream of N_2, and the reaction mixture was allowed to warm to rt. After 30 min, tetrafluorophthalonitrile (200 mg, 1.00 mmol, 1 equiv) was added and the solution was stirred at rt for 72 h. H_2O (0.5 mL) was then added and the mixture was concentrated *in vacuo*. The resulting yellow solid residue was collected *via* filtration under house vacuum, and was washed sequentially with H_2O (25 mL), EtOH (25 mL), and Et_2O (25 mL). Purification *via* recrystallisation from hot $CHCl_3$ (ca. 10 mL) and hexane (ca. 2 mL) gave 4CzIPN as a bright yellow solid (733 mg, 93%). The NMR spectroscopic data was in accordance with the literature.[4]

Data for 2,4,5,6-tetra(9*H*-carbazol-9-yl)isophthalonitrile (4CzIPN):

^{1}H NMR: (500 MHz, $CDCl_3$)
8.26–8.20 (m, 2H), 7.75–7.65 (m, 8H), 7.53–7.46 (m, 2H), 7.36–7.31 (m, 2H), 7.27–7.18 (m, 4H), 7.14–7.04 (m, 8H), 6.87–6.79 (m, 4H), 6.67–6.60 (m, 2H)

^{13}C NMR: (126 MHz, $CDCl_3$)
145.4, 144.8, 140.1, 138.3, 137.1, 134.9, 127.1, 125.9, 125.1, 124.7, 124.0, 122.5, 122.1, 121.5, 121.1, 120.6, 119.8, 116.5, 111.75, 110.1, 109.62, 109.57

C.3. Preparation of HAT Catalysts

Preparation of triisopropylsilanethiolate sodium(15-crown-5) salt (10)

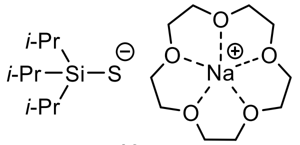

10

NaH (washed with hexane, 5.05 mg, 0.22 mmol, 1.1 equiv) was weighed into a vial in an argon-filled glove box, then the vial was sealed and transferred to a nitrogen-filled purge box (suitable for organic solvent usage). Tri(isopropyl)silanethiol (44.5 µL, 39.5 mg, 0.20 mmol, 1.0 equiv) was dissolved in the requisite dry, degassed solvent (100 µL) and the NaH was then added. The vial was swirled to aid mixing, and 15-crown-5 (40.5 µL, 45.1 mg, 0.21 mmol, 1.04 equiv) was added *via* microlitre syringe. The vial was again swirled to aid mixing, and the supernatant solution was transferred to a 2.0-mL volumetric flask and diluted with dry, degassed solvent. This 100 mM solution of thiolate $Na(15\text{-}C\text{-}5)^+$ salt **10** was generally used immediately, but it could be stored in a nitrogen-filled purge box for at least 2 weeks without decomposition.

Preparation of thioglycolate sodium(15-crown-5) salt (12)

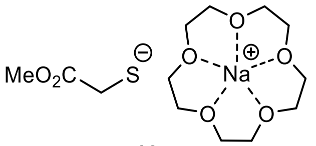

12

NaH (washed with hexane, 5.05 mg, 0.22 mmol, 1.1 equiv) was weighed into a vial in an argon-filled glove box, then the vial was sealed and transferred to a nitrogen-filled purge box (suitable for organic solvent usage). Methyl thioglycolate (19 µL, 22.5 mg, 202 µmol, 1.0 equiv) was dissolved in the requisite dry, degassed solvent (100 µL) and the NaH was then added. The vial was swirled to aid mixing, and 15-crown-5 (40.5 µL, 45.1 mg, 0.21 mmol, 1.04 equiv) was added *via* microlitre syringe. The vial was again swirled to aid mixing, and the supernatant solution was transferred to a 2.0-mL volumetric flask and diluted with dry, degassed solvent. This 100 mM solution of thiolate $Na(15\text{-}C\text{-}5)^+$ salt **12** was used immediately.

Preparation of tetrabutylammonium benzenesulfinate (13)

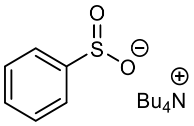

13

A 100-mL, Erlenmeyer flask equipped with a stirrer bar was charged with sodium benzenesulfinate (2.0 g, 12.2 mmol, 1.0 equiv) and H_2O (20 mL), and the resultant mixture was stirred for ca. 5 min until a clear, homogeneous solution was obtained. Et_2O (20 mL) and conc. aq. HCl (1.0 mL, 10.2 mmol, 0.84 equiv) were added sequentially, and the mixture was stirred for a further 5 min. The organic and aqueous layers were separated, and the aqueous phase was washed with portions of Et_2O (28 mL). The combined organic layers were then concentrated *in vacuo* (in air) and the residue was dried under vacuum (ca. 0.05 mmHg) for 4 h to give benzenesulfinic acid as a white crystalline solid (1.20 g, 71%). This material was stored under N_2 in a –20 °C freezer before use. Next, aqueous solutions of $Bu_4NOH{\bullet}30H_2O$ and the freshly-prepared benzenesulfinic acid were made up in 25-mL volumetric flasks. These were each titrated against 1.00 M solutions of HCl and NaOH, respectively, using universal indicator solution to determine the end-points (in conjunction with pH test strips). The concentration of the $Bu_4NOH{\bullet}30H_2O$ solution was 0.48 M, and the solution of benzenesulfinic acid was 0.27 M. Thus, a portion of the $Bu_4NOH{\bullet}30H_2O$ solution (17.0 mL) was mixed with a portion of the benzenesulfinic acid solution (9.75 mL), and the resultant mixture was stirred for 5 min. The solution was then diluted with portions of EtOH and concentrated *in vacuo*. Drying the residue under vacuum (ca. 0.05 mmHg) for 30 min gave **13** as a colourless, viscous oil which was stored under N_2 and used without further purification.

Data for **13**:

^{1}H NMR: (500 MHz, DMSO-d_6)
7.49–7.45 (m, 2H), 7.36–7.32 (m, 2H), 7.29–7.25 (m, 1H), 3.22–3.10 (m, 8H), 1.61–1.51 (m, 8H), 1.31 (m, 8H), 0.93 (t, J = 7.3 Hz, 12H)

^{13}C NMR: (126 MHz, DMSO-d_6)
127.7, 124.3, 57.5, 23.05, 19.2, 13.5

C.4. Preparation of Authentic Samples

Preparation of 1-azaspiro[4.5]decan-2-one (17b)

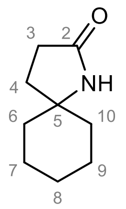

17b

A 50-mL, round-bottomed ampoule flask equipped with a water condenser, nitrogen inlet and a stirrer bar was charged with cyclohexylamine **5** (7.64 mL, 6.62 g, 66.1 mmol, 1.3 equiv) and methyl acrylate **14** (4.53 mL, 4.35 g, 50.0 mmol, 1.0 equiv), then was heated to 160 °C. A 1.0 mL portion of a mixture of di-*tert*-butyl peroxide (940 µL, 750 mg, 5.08 mmol, 10 mol%) and cyclohexylamine **5** (6.95 mL, 6.02 g, 60.0 mmol) was added, followed by further 1.0 mL portions every 15 min for 2 h. Following the final addition, the reaction was stirred for a further 30 min and then left to cool to rt over 30 min. The excess cyclohexylamine was removed by concentration in *vacuo* (42 mbar, 55 °C) to give a yellow oil. Purification *via* flash column chromatography on silica gel (653 g, 50 mm Ø) in 10:90 40-60° petrol-EtOAc (2 L) then 90:9:1 CH_2Cl_2–MeOH–aq. NH_4OH (1 L) gave a yellow crystalline solid contaminated with oil. Trituration with Et_2O at –78 °C, with the supernatant liquid removed *via* pipette each time, gave **17b** as a white crystalline solid (621 mg, 8%). The NMR spectroscopic data was in accordance with the literature.[5,6]

Data for **17b**:

mp: 127–130 °C {lit.[6] 106–108 °C}

^{1}H NMR: (500 MHz, $CDCl_3$)

6.33 (s, 1H, N*H*), 2.37 (t, *J* = 8.1 Hz, 2H, C(3)H_2), 1.90 (t, *J* = 8.1, Hz, 2H, C(4)H_2), 1.60–1.47 (m, 8H, C(6)H_2, C(7)H_2, C(9)H_2, C(10)H_2), 1.46–1.36 (m, 2H, C(8)H_2)

^{13}C NMR: (126 MHz, $CDCl_3$)

177.3 (*C*(2)), 59.4 (*C*(5)), 38.5 (*C*(6), *C*(10)), 32.9 (*C*(4)), 30.0 (*C*(3)), 25.3 (*C*(8)), 23.2 (*C*(7), *C*(9))

IR: (neat)

3204 (w), 3084 (w), 2920 (m), 2865 (w), 1739 (w), 1684 (s), 1653 (m), 1455 (m), 1423 (w), 1374 (w), 1340 (m), 1305 (w), 1276 (m), 1259 (m), 1236 (w), 1210 (w), 1164 (w), 1150 (w), 1101 (w), 1075 (w), 1038 (w), 1006 (w), 983 (w), 932 (w), 894 (w), 848 (w), 837 (w), 820 (w), 794 (w), 739 (m)

MS: (ESI$^+$)

307 (24%), 176 ([M+Na]$^+$, 26%), 154 ([M+H]$^+$, 100%)

HRMS: (ESI$^+$)

calcd for $C_9H_{16}NO$: 154.1226, found: 154.1229

Preparation of butyl 3-(cyclohexylamino)propanoate (28)

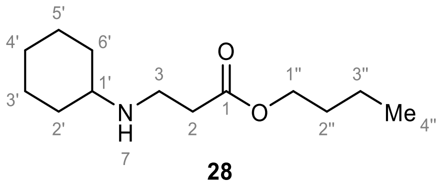

28

A flame-dried, 25-mL Schlenk flask equipped with a stirrer bar, and an N_2 inlet was charged with cyclohexylamine **5** (1.00 mL, 867 mg, 8.74 mmol, 1.0 equiv), butyl acrylate **6** (1.46 mL, 1.30 g, 10.1 mmol, 1.16 equiv), and toluene (2.0 mL). The mixture was stirred at 100 °C for 24 h, then was allowed to cool to rt and was concentrated *in vacuo*. Purification *via* flash column chromatography on silica gel (80 g) in 100:0:0→90:9:1 CH_2Cl_2–MeOH–aq. NH_4OH (over 10 CV) gave **28** as a yellow oil (89.3 mg, 45%). The NMR spectroscopic data was in accordance with the literature.[7]

Data for **28**:

^{1}H NMR: (500 MHz, $CDCl_3$)

4.04–3.96 (m, 2H, C(1″)H_2), 2.86–2.76 (m, 2H, C(3)H_2), 2.45–2.30 (m, 3H, C(2)H_2, C(1′)H), 1.85–1.47 (m, 8H, C(2′)H_A, C(6′)H_A, C(3′)H_A, C(5′)H_A, C(2″)H_2, C(4′)H_2), 1.36–0.90 (m, 6H, C(3″)H_2, C(2′)H_B, C(6′)H_B, C(3′)H_B, C(5′)H_B), 0.90–0.79 (m, 3H, C(4″)H_3)

^{13}C NMR: (126 MHz, $CDCl_3$)

178.85 (*C*(1)), 64.2 (*C*(1″)), 56.45 (*C*(1′)), 42.05 (*C*(3)), 35.0 (*C*(2)), 33.5 (*C*(2′), *C*(6′)), 30.6 (*C*(2″)), 26.1 (*C*(4′)), 25.0 (*C*(3′), *C*(5′)), 19.1 (*C*(3″)), 13.6 (*C*(4″))

IR: (neat)

2957 (w), 2926 (m), 2854 (w), 1730 (m), 1681 (w), 1463 (w), 1449 (w), 1391 (w), 1365 (w), 1345 (w), 1305 (w), 1259 (w), 1239 (w), 1170 (m), 1147 (w), 1130 (w), 1064 (w), 1047 (w), 1024 (w), 978 (w), 960 (w), 946 (w), 888 (w), 843 (w), 799 (w), 782 (w)

MS: (ESI$^+$)

228 ([M+H]$^+$, 100%)

HRMS: (ESI$^+$)

calcd for $C_{13}H_{26}NO_2$: 228.1958, found: 228.1963

Preparation of methyl 3-(cyclohexylamino)propanoate (29)

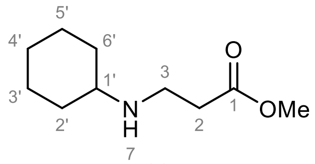

29

A 25-mL round bottomed flask equipped with a stirrer bar was charged with cyclohexylamine **5** (1.16 mL, 1.0 g, 10.0 mmol, 1.0 equiv), methyl acrylate **14** (0.91 mL, 869 mg, 10.0 mmol, 1.0 equiv), and MeOH (8.0 mL). The mixture was stirred at rt for 24 h, and was then concentrated *in vacuo*. Purification *via* flash column chromatography on silica gel (200 mL, 50 mm Ø) in 50:50 40-60° petrol-EtOAc followed by 90:9:1 CH_2Cl_2–MeOH–aq. NH_4OH gave, after trituration in Et_2O to remove an insoluble contaminant, **29** as a yellow oil (1.12 g, 60%). The NMR spectroscopic data was in accordance with the literature.[8]

Data for **29**:

^{1}H NMR: (500 MHz, $CDCl_3$)

3.57 (s, 3H, O*Me*), 2.80 (t, *J* = 6.7 Hz, 2H, C(3)H_2), 2.41 (t, *J* = 6.6 Hz, 2H, C(2)H_2), 2.31 (m, C(1′)*H*) 1.87–0.83 (m, 11H, N*H*, C(2′)H_2, C(3′)H_2, C(4′)H_2, C(5′)H_2, C(6′)H_2)

^{13}C NMR: (126 MHz, $CDCl_3$)

173.25 (*C*(1)), 56.5 (*C*(1′)), 51.5 (O*Me*), 42.05 (*C*(3)), 34.9 (*C*(2)), 33.5 (*C*(2′), *C*(6′)), 26.15 (*C*(4′)), 25.0 (*C*(3′), *C*(5′))

IR: (neat)

2926 (m), 2854 (w), 1736 (m), 1650 (w), 1578 (w), 1544 (w), 1449 (w), 1437 (w), 1363 (w), 1348 (w), 1317 (w), 1253 (w), 1196 (m), 1173 (m), 1150 (w), 1127 (w), 1041 (w), 1015 (w), 998 (w), 966 (w), 926 (w), 891 (w), 840 (w), 799 (w), 788 (w), 748 (w), 707 (w)

MS: (ESI$^+$)

272 (18%), 253 (14%), 186 ([M+H]$^+$, 100%)

<u>HRMS:</u> (ESI^+)

calcd for $C_{10}H_{20}NO_2$: 186.1489, found: 186.1493

D. Reaction Optimisation

D.1. Measurement of GC Relative Response Factors

Stock solutions of the various analytes in EtOAc, plus one for a dodecane standard, were first prepared in 5.0-mL volumetric flasks, with concentrations in the range 48.8–52.3 mM. Each of these stock solutions were used to prepare a series of 5 calibration samples for each analyte, each containing a fixed quantity of dodecane and a varying quantity of the analyte. These calibration samples were prepared in 10-mL volumetric flasks, using EtOAc as the solvent, and 1.00 mL of each solution was then transferred to a GC vial for analysis. Three injections were performed for each individual vial, to compensate for errors in injection volume. Relative response factors for quantitative GC-FID analysis were obtained by regression analysis using the following equation: relative response factor = (area analyte × mmol dodecane)/(mmol analyte × area dodecane).

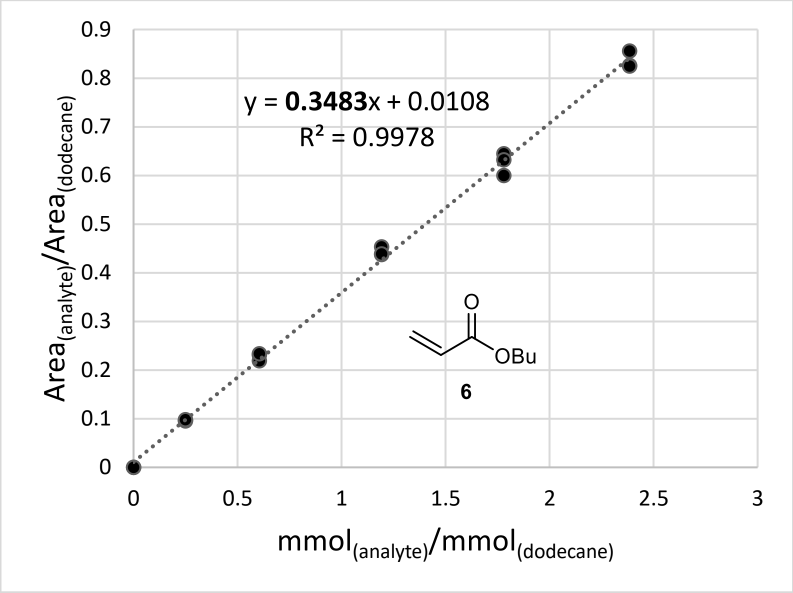

sample	dodecane (mmol)	dodecane (area)	**6** (mmol)	**6** (area)
1	0.028223	3541258.59	0.067296	2924249.5
	0.028223	3322226.05	0.067296	2740052.4
	0.028223	3861436.21	0.067296	3303147.1
2	0.028223	2836197.36	0.050221	1700373.2
	0.028223	3467555.06	0.050221	2234039.6
	0.028223	3860125.31	0.050221	2439760.2
3	0.028223	3073673.92	0.033648	1392072.2
	0.028223	3948670.15	0.033648	1727005.5
	0.028223	2923330.82	0.033648	1282254
4	0.028223	3996291.37	0.017075	921675.03
	0.028223	3220143.53	0.017075	704748.91
	0.028223	3322298.22	0.017075	774904.14
5	0.028223	3344472.21	0.007031	319040.01
	0.028223	2896202.95	0.007031	280127.19
	0.028223	2612955	0.007031	255603.48

Table S3. Relative response factor measurement for **6**.

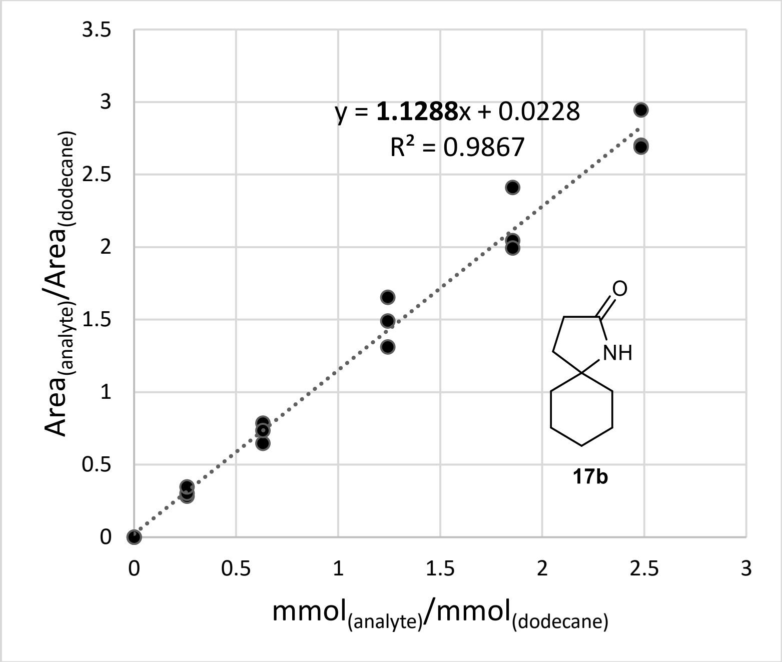

sample	dodecane (mmol)	dodecane (area)	**17b** (mmol)	**17b** (area)
1	0.028223	3541258.59	0.070135	9568766.9
	0.028223	3322226.05	0.070135	9779191.1
	0.028223	3861436.21	0.070135	10382137
2	0.028223	2836197.36	0.052340	6834289.2
	0.028223	3467555.06	0.052340	7090288.2
	0.028223	3860125.31	0.052340	7688787.2
3	0.028223	3073673.92	0.035068	4576715.4
	0.028223	3948670.15	0.035068	5172722.9
	0.028223	2923330.82	0.035068	4833132.6
4	0.028223	3996291.37	0.017795	2581461.2
	0.028223	3220143.53	0.017795	2526861.8
	0.028223	3322298.22	0.017795	2437532.9
5	0.028223	3344472.21	0.007328	945516.71
	0.028223	2896202.95	0.007328	877423.32
	0.028223	2612955	0.007328	901801.68

Table S4. Relative response factor measurement for **17b**.

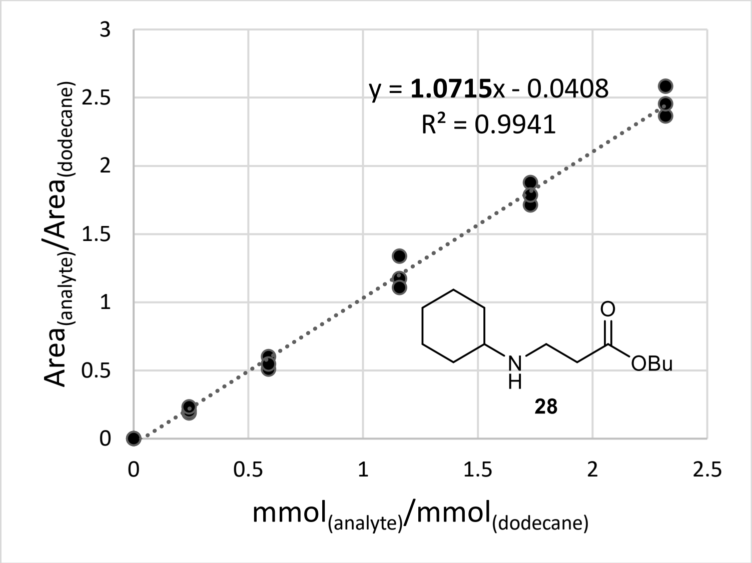

sample	dodecane (mmol)	dodecane (area)	**28** (mmol)	**28** (area)
1	0.028223	3541258.59	0.065423	8378908.3
	0.028223	3322226.05	0.065423	8579817.4
	0.028223	3861436.21	0.065423	9473555
2	0.028223	2836197.36	0.048823	5323607.7
	0.028223	3467555.06	0.048823	5939866.7
	0.028223	3860125.31	0.048823	6892541.6
3	0.028223	3073673.92	0.032712	3603544.2
	0.028223	3948670.15	0.032712	4374016.1
	0.028223	2923330.82	0.032712	3908271.9
4	0.028223	3996291.37	0.016600	2033264.7
	0.028223	3220143.53	0.016600	1934718.2
	0.028223	3322298.22	0.016600	1814203.3
5	0.028223	3344472.21	0.006835	628957.75
	0.028223	2896202.95	0.006835	589976.84
	0.028223	2612955	0.006835	607233.95

Table S5. Relative response factor measurement for **28**.

D.2. Screening of Reaction Conditions

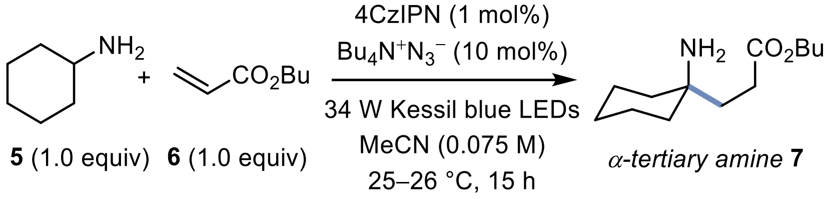

Entry	Deviation from above conditions	**7** yield[a]
1	$[Ir(ppy)_2(dtbbpy)]PF_6$ as PC	79%
2	$[Ir(dF(CF_3)ppy)_2(dtbbpy)]PF_6$ as PC	83%[b]
3	**10** as HAT catalyst	70%
4	**11** as HAT catalyst	42%[b]
5	**12** or **13** as HAT catalyst	<1%
6	none	85%
7	DMF as solvent (0.075 M)	46%
8	DMF as solvent (0.075 M) with $[Ir(ppy)_2(dtbbpy)]PF_6$ as PC	77%
9	DMF as solvent (0.075 M) with **10** as HAT catalyst	73%
10	EtOAc as solvent (0.075 M)	74%
11	concentration of 0.2 M in MeCN	84%[b,c]
12	concentration of 0.4 M in MeCN	44%[b,d]
13	no 4CzIPN or no $Bu_4N^+N_3^-$ or no light	0%[b]
14	no drying or degassing of solvent	24%[b]

Table S6. Optimisation of reaction conditions. [a] Measured by GC against dodecane as an internal standard; note that lactamisation occurs under the analysis conditions. [b] Measured by ^{1}H NMR against mesitylene as an internal standard. [c] <1% of the aza-Michael adduct was formed. [d] 4% of the aza-Michael adduct was formed.

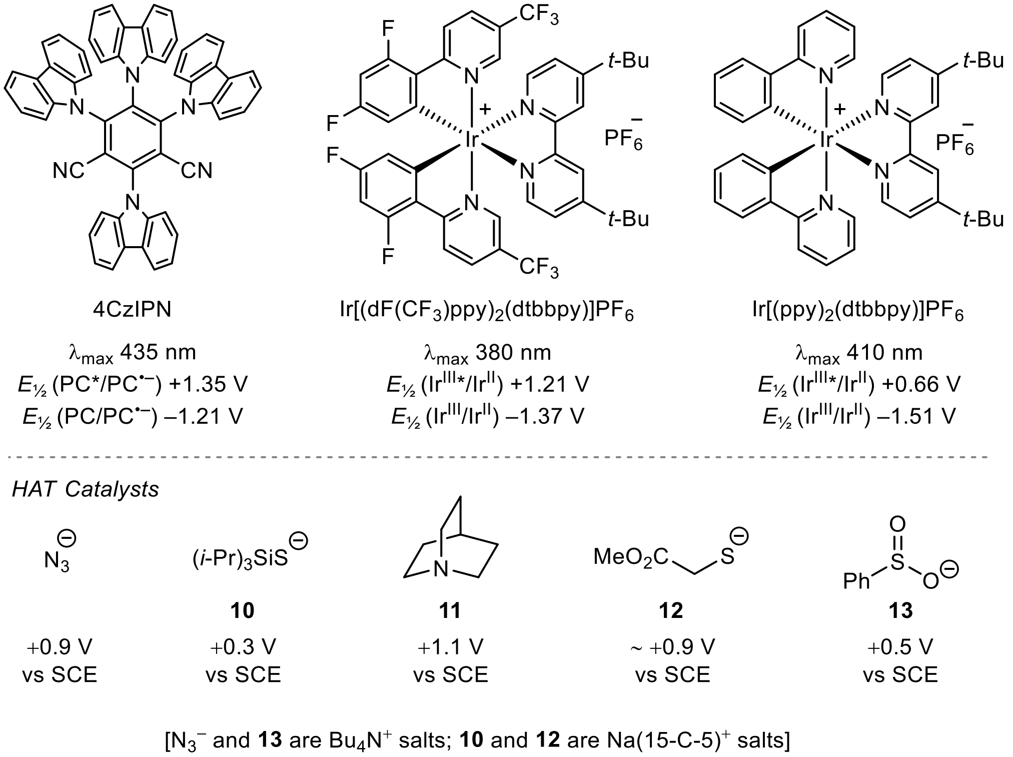

Fig. S4. Photocatalysts and HAT catalysts employed in reaction optimisation studies.

Inside a nitrogen-filled purge box, a series of 0.3-mL, crimp-top vials were charged with stock solutions of cyclohexylamine **5** (0.25 M in MeCN, 45.0 µL, 11.25 µmol), butyl acrylate **6** (0.25 M in MeCN, 45.0 µL, 11.25 µmol), the requisite HAT catalyst (0.10 M in MeCN, 10.0 µL, 1.00 µmol), the requisite photoredox catalyst (5.0 mM in MeCN, 22.5 µL, 0.11 µmol), and dodecane (0.47 M in hexane, 10.0 µL, 4.70 µmol). Finally, additional MeCN (20.0 µL) was added to bring the reaction concentration to 0.075 M. The vials were capped with aluminium crimp seals and were removed from the purge box. They were then transferred to a photoreactor and irradiated with a single Kessil H150-Blue lamp for 15 h. Following irradiation, 80 µL of each reaction mixture was transferred to a screw-top GC vial and diluted with 0.80 mL of EtOAc, prior to GC-FID analysis. [*Note*: It appears that lactamisation to give the γ-lactam product **17b** occurs smoothly at the temperature of the GC injection block].

D.3. Effect of Light Source: Reaction Monitoring by Flow NMR

To determine the optimal irradiation wavelength, we tested 405, 425, 450, and 475 nm LED lamps as light sources, and used flow NMR spectroscopy to qualitatively ascertain the initial rate (see General Experimental section for details).

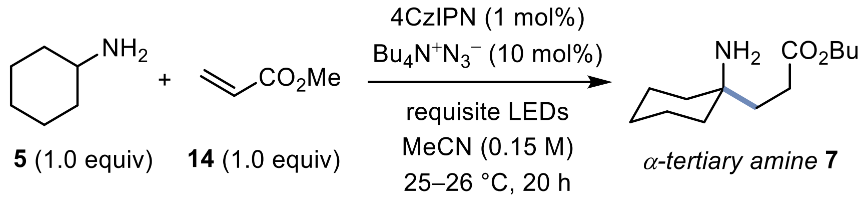

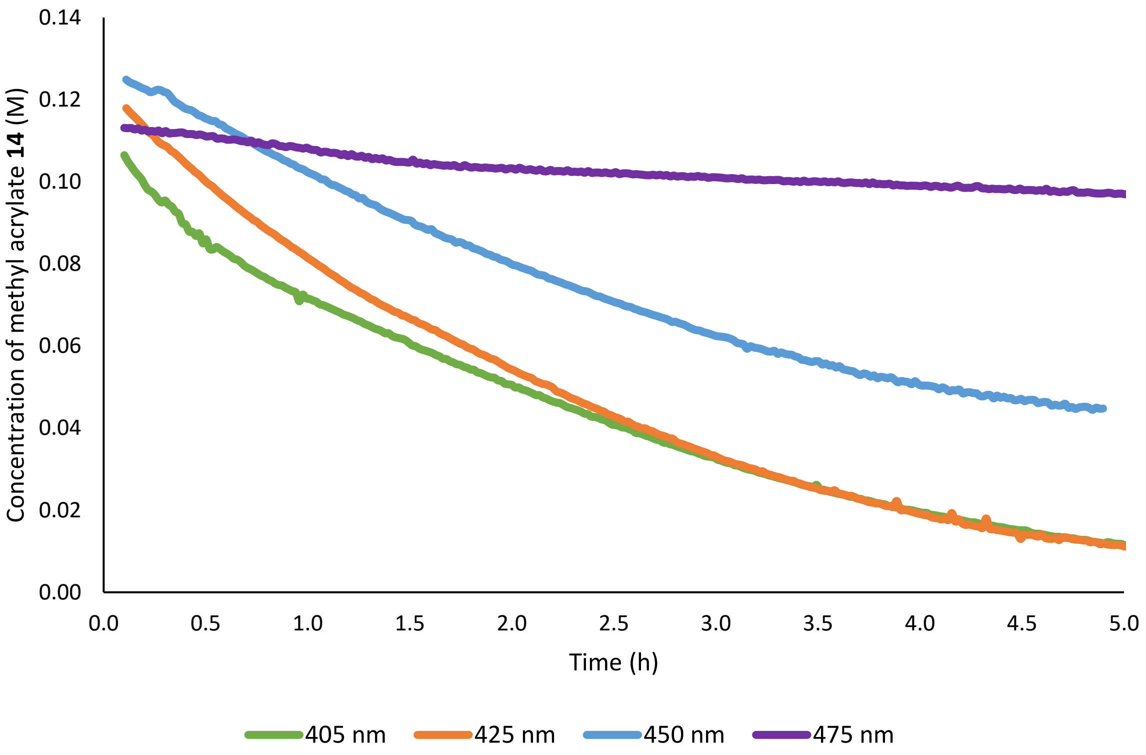

Fig. S5. Kinetic data for the reaction of cyclohexylamine **5** (1.5 mmol, 0.15 M in MeCN) with methyl acrylate **14** (1.5 mmol, 0.15 M in MeCN) in the presence of 4CzIPN (1 mol%, 1.5 mM) and tetrabutylammonium azide (10 mol%, 15 mM) and the respective wavelength of light as indicated in the chart.

E. Reaction Generality

E.1. Scope of α-C–H Alkylation with α-Disubstituted Primary Amines

Preparation of 1-azaspiro[4.6]undecan-2-one (17a)

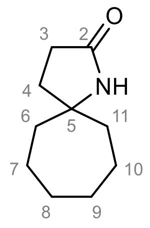

17a

Following *General Procedure 1*, cycloheptylamine **1a** (59 µL, 51 mg, 0.45 mmol, 1.0 equiv), methyl acrylate **14** (41 µL, 39 mg, 0.45 mmol, 1.0 equiv), 4CzIPN (2.28 mM in MeCN, 1.98 mL, 4.5 µmol, 1 mol%), tetrabutylammonium azide (70.3 mM in MeCN, 640 µL, 45 µmol, 10 mol%), and MeCN (390 µL) were reacted for 20 h with a 425 nm LED lamp. Following concentration *in vacuo*, the residue was dissolved in MeOH (5 mL) and Et_3N (0.25 mL, 0.18 g, 1.8 mmol, 4.0 equiv) was added. The reaction was then heated at reflux for 2 h, followed by concentration *in vacuo*. Purification *via* flash column chromatography on silica gel (12 g) in CH_2Cl_2 (5 CV) then 100:0:0→90:9:1 CH_2Cl_2–MeOH–aq. NH_4OH (over 15 CV) then 90:9:1 CH_2Cl_2–MeOH–aq. NH_4OH (17 CV), followed by reversed-phase flash column chromatography on C_{18} silica gel (12 g) in H_2O (3 CV) then 100:0→0:100 H_2O–MeOH (over 15 CV) then MeOH (16 CV), gave **17a** as a pale yellow-white solid (45.0 mg, 60%). The NMR spectroscopic data was in accordance with the literature.[9]

Data for **17a**:

mp: 132–133 °C

^{1}H NMR: (500 MHz, $CDCl_3$)
6.49 (br s, 1H, N*H*), 2.36 (t, J = 7.9 Hz, 2H, C(3)H_2), 1.90 (t, J = 7.9 Hz, 2H, C(4)H_2), 1.77–1.67 (m, 4H, C(6)H_2, C(11)H_2), 1.62–1.46 (m, 8H, C(7)H_2, C(8)H_2, C(9)H_2, C(10)H_2)

^{13}C NMR: (126 MHz, $CDCl_3$)
177.2 (*C*(2)), 62.7 (*C*(5)), 41.5 (*C*(6), *C*(11)), 34.8 (*C*(4)), 30.0 (*C*(3)), 29.0 (*C*(8), *C*(9)), 22.8 (*C*(7), *C*(10))

IR: (neat)
3171 (w), 3080 (w), 2919 (w), 2855 (w), 1689 (m), 1652 (m), 1460 (w), 1427 (w),

1376 (w), 1352 (w), 1285 (w), 1256 (w), 1217 (w), 1185 (w), 1114 (w), 1074 (w), 1051 (w), 989 (w), 954 (w), 920 (w), 896 (w), 853 (w), 841 (w), 824 (w), 779 (w), 734 (w)

MS: (ESI^+)
357 (11%), 190 ($[M+Na]^+$, 100%), 168 ($[M+H]^+$, 38%)

HRMS: (ESI^+)
calcd for $C_{10}H_{18}NO$: 168.1383, found: 168.1384

Preparation of 1-azaspiro[4.5]decan-2-one (17b)

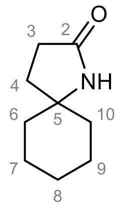

17b

Following *General Procedure 1*, cyclohexylamine **5** (52 µL, 45 mg, 0.45 mmol, 1.0 equiv), methyl acrylate **14** (41 µL, 39 mg, 0.45 mmol, 1.0 equiv), 4CzIPN (2.28 mM in MeCN, 1.98 mL, 4.5 µmol, 1 mol%), tetrabutylammonium azide (70.3 mM in MeCN, 640 µL, 45 µmol, 10 mol%), and MeCN (390 µL) were reacted for 20 h with a 425 nm LED lamp. Following concentration *in vacuo*, the residue was dissolved in MeOH (5 mL) and Et_3N (0.25 mL, 0.18 g, 1.8 mmol, 4.0 equiv) was added. The reaction was then heated at reflux for 2 h, followed by concentration *in vacuo*. Purification *via* flash column chromatography on silica gel (12 g) in CH_2Cl_2 (5 CV) then 100:0:0→90:9:1 CH_2Cl_2–MeOH–aq. NH_4OH (over 15 CV) then 90:9:1 CH_2Cl_2–MeOH–aq. NH_4OH (17 CV), followed by reversed-phase flash column chromatography on C_{18} silica gel (50 g, 20 mm Ø) in 70:30 MeOH–H_2O, gave **17b** as a white crystalline solid (47.2 mg, 68%). The NMR spectroscopic data was in accordance with the literature.[5,6]

Preparation of 1-azaspiro[4.4]nonan-2-one (17c)

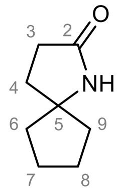

17c

Following *General Procedure 1*, cyclopentylamine **1c** (45 µL, 39 mg, 0.45 mmol, 1.0 equiv), 2-methoxyethyl acrylate **15** (59 µL, 60 mg, 0.45 mmol, 1.0 equiv), 4CzIPN (2.28 mM in MeCN, 1.98 mL, 4.5 µmol, 1 mol%), tetrabutylammonium azide (70.3 mM in MeCN, 640 µL, 45 µmol, 10 mol%), and MeCN (390 µL) were reacted for 20 h with a 425 nm LED lamp. Following concentration *in vacuo*, the residue was dissolved in MeCN (5 mL) and Et_3N (0.25 mL, 0.18 g, 1.8 mmol, 4.0 equiv) was added. The reaction was then heated at reflux for 12 h, followed by concentration *in vacuo*. Purification *via* flash column chromatography on silica gel (12 g) in CH_2Cl_2 (5 CV) then 100:0:0→90:9:1 CH_2Cl_2–MeOH–aq. NH_4OH (over 15 CV) then 90:9:1 CH_2Cl_2–MeOH–aq. NH_4OH (17 CV), followed by reversed-phase flash column chromatography on C_{18} silica gel (15.5 g) in H_2O (3 CV) then 100:0→0:100 H_2O–MeOH (over 15 CV) then MeOH (16 CV), gave **17c** as a white crystalline solid (40.7 mg, 65%). The NMR spectroscopic data was in accordance with the literature.[10]

Data for **17c**:

mp: 130–132 °C {lit.[10] 142–144 °C}

^{1}H NMR: (500 MHz, $CDCl_3$)
7.32 (br s, 1H, N*H*), 2.35 (t, *J* = 7.9 Hz, 2H, C(3)H_2), 1.97 (t, *J* = 7.9 Hz, 2H, C(4)H_2), 1.75–1.58 (m, 8H, C(6)H_2, C(7)H_2, C(8)H_2, C(9)H_2)

^{13}C NMR: (126 MHz, $CDCl_3$)
177.4 (*C*(2)), 67.1 (*C*(5)), 39.3 (*C*(6), *C*(9)), 34.0 (*C*(4)), 31.0 (*C*(3)), 23.3 (*C*(7), *C*(8))

IR: (neat)
3165 (w), 3079 (w), 2933 (w), 2870 (w), 2169 (w), 2105 (w), 1686 (m), 1650 (m), 1462 (w), 1432 (w), 1378 (w), 1300 (w), 1258 (w), 1033 (w), 1014 (w), 944 (w), 928 (w), 771 (m)

MS: (ESI^+)
301 (18%), 279 (11%), 162 ($[M+Na]^+$, 100%), 140 ($[M+H]^+$, 65%)

HRMS: (ESI^+)
calcd for $C_8H_{14}ON$: 140.1070, found: 140.1072

Preparation of 5-azaspiro[3.4]octan-6-one (17d)

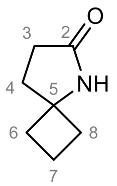

17d

Following *General Procedure 1*, cyclobutylamine **1d** (39 μL, 33 mg, 0.45 mmol, 1.0 equiv), 2-methoxyethyl acrylate **15** (59 μL, 60 mg, 0.45 mmol, 1.0 equiv), 4CzIPN (2.28 mM in MeCN, 1.98 mL, 4.5 μmol, 1 mol%), tetrabutylammonium azide (70.3 mM in MeCN, 640 μL, 45 μmol, 10 mol%), and MeCN (390 μL) were reacted for 20 h with a 425 nm LED lamp. Following concentration *in vacuo,* the residue was dissolved in MeCN (5 mL) and Et_3N (0.25 mL, 0.18 g, 1.8 mmol, 4.0 equiv) was added. The reaction was then heated at reflux for 12 h, followed by concentration *in vacuo*. Purification *via* flash column chromatography on high performance silica gel (12 g) in CH_2Cl_2 (5 CV) then 100:0:0→90:9:1 CH_2Cl_2–MeOH–aq. NH_4OH (over 25 CV) then 90:9:1 CH_2Cl_2–MeOH–aq. NH_4OH (17 CV) gave **17d** as a white solid (27.7 mg, 49%). The NMR spectroscopic data was in accordance with the literature.[11]

Data for **17d**:

mp: 64–66°C

^{1}H NMR: (500 MHz, $CDCl_3$)
6.62 (br s, 1H, N*H*), 2.39–2.31 (m, 2H, C(3)H_2), 2.28–2.17 (m, 4H, C(4)H_2, C(6)H_A, C(8)H_A), 2.09 (m, 2H, C(6)H_B, C(8)H_B), 1.70 (m, 2H, C(7)H_2)

^{13}C NMR: (126 MHz, $CDCl_3$)
177.5 (*C*(2)), 60.4 (*C*(5)), 36.5 (*C*(6), *C*(8)), 34.4 (*C*(4)), 30.4 (*C*(3)), 13.25 (*C*(7))

IR: (neat)
3214 (w), 2963 (w), 2930 (w), 2872 (w), 1683 (s), 1459 (w), 1423 (w), 1361 (w), 1264 (m), 1213 (w), 1175 (w), 1118 (w), 1075 (w), 1028 (w), 973 (w), 923 (w), 734 (m), 689 (m)

MS: (ESI^+)
241 (20%), 148 ($[M+Na]^+$, 100%), 126 ($[M+H]^+$, 43%)

HRMS: (ESI^+)
calcd for $C_7H_{12}ON$: 126.0913, found: 126.0916

Preparation of 5,5-dipropylpyrrolidin-2-one (17e)

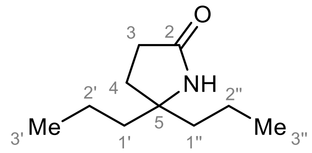

17e

Following *General Procedure 1*, heptan-4-amine **1e** (41 µL, 52 mg, 0.45 mmol, 1.0 equiv), methyl acrylate **14** (41 µL, 39 mg, 0.45 mmol, 1.0 equiv), 4CzIPN (2.28 mM in MeCN, 1.98 mL, 4.5 µmol, 1 mol%), tetrabutylammonium azide (70.3 mM in MeCN, 640 µL, 45 µmol, 10 mol%), and MeCN (390 µL) were reacted for 20 h with a 425 nm LED lamp. Following concentration *in vacuo*, the residue was dissolved in MeOH (5 mL) and Et_3N (0.25 mL, 0.18 g, 1.8 mmol, 4.0 equiv) was added. The reaction was then heated at reflux for 2 h, followed by concentration *in vacuo*. Purification *via* flash column chromatography on silica gel (12 g) in CH_2Cl_2 (5 CV) then 100:0:0→90:9:1 CH_2Cl_2–MeOH–aq. NH_4OH (over 15 CV) then 90:9:1 CH_2Cl_2–MeOH–aq. NH_4OH (17 CV), followed by reversed-phase flash column chromatography on C_{18} silica gel (12 g) in H_2O (3 CV) then 100:0→0:100 H_2O–MeOH (over 15 CV) then MeOH (16 CV), gave **17e** as a pale cream solid (60 mg, 79%).

Data for **17e**:

mp: 43–45 °C

^{1}H NMR: (500 MHz, $CDCl_3$)

6.14 (br s, 1H, N*H*), 2.36 (t, *J* = 8.1 Hz, 2H, C(3)H_2), 1.89 (t, *J* = 8.1 Hz, 2H, C(4)H_2), 1.54–1.40 (m, 4H, C(1′)H_2, C(1″)H_2), 1.39–1.19 (m, 4H, C(2′)H_2, C(2″)H_2), 0.92 (t, *J* = 7.3 Hz, 6H, C(3′)H_3, C(3″)H_3)

^{13}C NMR: (126 MHz, $CDCl_3$)

177.4 (*C*(2)), 61.9 (*C*(5)), 42.5 (*C*(1′), *C*(1″), 31.6 (*C*(4)), 30.6 (*C*(3)), 17.3 (*C*(2′), *C*(2″), 14.6 (*C*(3′), *C*(3″))

IR: (neat)

3170 (w), 3076 (w), 2961 (m), 2923 (w), 2875 (w), 1687 (m), 1458 (m), 1422 (w), 1380 (m), 1359 (w), 1322 (w), 1290 (w), 1255 (w), 1208 (w), 1153 (w), 1099 (w), 1026 (w), 937 (w), 899 (w), 877 (w), 858 (w), 802 (w), 779 (w), 748 (w), 671 (w)

MS: (ESI^+)

361 (14%), 192 ($[M+Na]^+$, 100%), 170 ($[M+H]^+$, 72%)

HRMS: (ESI^+)
calcd for $C_{10}H_{20}NO$: 170.1539, found: 170.1541

Preparation of (*RS*)-5-cyclohexylpyrrolidin-2-one (17f)

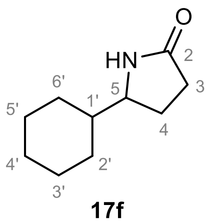

17f

Following *General Procedure 1*, cyclohexanemethylamine **1f** (120 µL, 104 mg, 0.90 mmol, 2.0 equiv), 2-methoxyethyl acrylate **15** (59 µL, 59 mg, 0.45 mmol, 1.0 equiv), $[Ir(dF(CF_3)ppy)_2(dtbbpy)]PF_6$ (5 mg, 4.5 µmol, 1 mol%), tetrabutylammonium azide (70.3 mM in MeCN, 640 µL, 45 µmol, 10 mol%), and MeCN (2.36 mL) were reacted for 20 h with a 425 nm LED lamp. Following concentration *in vacuo*, the residue was dissolved in MeCN (5 mL) and Et_3N (0.25 mL, 0.18 g, 1.8 mmol, 4.0 equiv) was added. The reaction was then heated at 100 °C in a microwave vial for 24 h, followed by concentration *in vacuo*. Purification *via* flash column chromatography on silica gel (12 g) in CH_2Cl_2 (5 CV) then 100:0:0→90:9:1 CH_2Cl_2–MeOH–aq. NH_4OH (over 15 CV) then 90:9:1 CH_2Cl_2–MeOH–aq. NH_4OH (17 CV), followed by reversed-phase flash column chromatography on C_{18} silica gel (15.5 g) in H_2O (3 CV) then 100:0→0:100 H_2O–MeOH (over 15 CV) then MeOH (16 CV), gave **17f** as a white crystalline solid (37.9 mg, 49%). The NMR spectroscopic data was in accordance with the literature.[12]

Data for **17f**:

mp: 120–121 °C {lit.[12] 117–118 °C (petroleum ether/acetone)}

^{1}H NMR: (500 MHz, $CDCl_3$)
5.77 (s, 1H, N*H*), 3.36 (app q, *J* = 7.1 Hz, 1H, C(5)*H*), 2.33–2.27 (m, 2H, C(3)H_2), 2.22–2.14 (m, 1H, C(4)H_A), 1.85–1.65 (m, 6H, C(4)H_B, C(2′)H_A, C(3′)H_A, C(4′)H_A, C(5′)H_A, C(6′)H_A), 1.35–1.08 (m, 4H, C(1′)*H*, C(3′)H_B, C(4′)H_B, C(5′)H_B), 1.04–0.85 (m, 2H, C(2′)H_B, C(6′)H_B)

^{13}C NMR: (126 MHz, $CDCl_3$)
178.2 (*C*(2)), 59.6 (*C*(5)), 43.55 (*C*(1′)), 30.3 (*C*(3)), 29.6 (*C*(2′) or *C*(6′)), 28.8 (*C*(6′) or *C*(2′)), 26.4 (*C*(4′)), 26.0 (*C*(3′) or *C*(5′)), 25.9 (*C*(5′) or *C*(3′)), 25.2 (*C*(4))

IR: (neat)

3172 (w), 3089 (w), 2985 (w), 2966 (w), 2938 (m), 2916 (m), 2855 (m), 1682 (s), 1454 (m), 1422 (w), 1394 (w), 1350 (m), 1340 (w), 1330 (w), 1313 (w), 1279 (m), 1263 (m), 1228 (w), 1206 (w), 1110 (w), 1101 (w), 1029 (w), 988 (w), 976 (w), 933 (w), 884 (w), 845 (w), 806 (m), 796 (m), 693 (m)

MS: (ESI^+)

335 (20%), 190 ($[M+Na]^+$, 47%), 169 (10%), 168 ($[M+H]^+$, 100%)

HRMS: (ESI^+)

calcd for $C_{10}H_{18}NO$: 168.1381, found: 168.1388

Preparation of (*RS*)-5-cyclohexyl-5-methylpyrrolidin-2-one (17g)

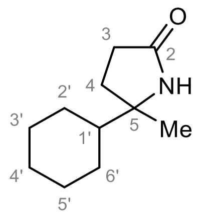

17g

Following *General Procedure 1*, (*RS*)-1-cyclohexylethylamine **1g** (58 mg, 0.45 mmol, 1.0 equiv), methyl acrylate **14** (41 µL, 39 mg, 0.45 mmol, 1.0 equiv), 4CzIPN (2.28 mM in MeCN, 1.98 mL, 4.5 µmol, 1 mol%), tetrabutylammonium azide (70.3 mM in MeCN, 640 µL, 45 µmol, 10 mol%), and MeCN (390 µL) were reacted for 20 h with a 425 nm LED lamp. Following concentration *in vacuo*, the residue was dissolved in MeOH (5 mL) and Et_3N (0.25 mL, 0.18 g, 1.8 mmol, 4.0 equiv) was added. The reaction was then heated at reflux for 2 h, followed by concentration *in vacuo*. Purification *via* flash column chromatography on silica gel (12 g) in CH_2Cl_2 (5 CV) then 100:0:0→90:9:1 CH_2Cl_2–MeOH–aq. NH_4OH (over 15 CV) then 90:9:1 CH_2Cl_2–MeOH–aq. NH_4OH (17 CV), followed by reversed-phase flash column chromatography on C_{18} silica gel (50 g, 20 mm Ø) in 80:20 MeOH–H_2O, gave **17g** as a white solid (33.7 mg, 42%).

Data for **17g**:

mp: 130–132 °C

^{1}H NMR: (500 MHz, $CDCl_3$)

6.45 (br s, 1H, N*H*), 2.45–2.26 (m, 2H, C(3)H_2), 2.07–1.97 (m, 1H, C(4)H_A), 1.84–1.63 (m, 6H, C(4)H_B, C(1′)*H*, C(2′)H_2, C(6′)H_2), 1.38–0.86 (m, 9H, C(5)*Me*, C(3′)H_2, C(4′)H_2, C(5′)H_2)

<u>^{13}C NMR:</u> (126 MHz, $CDCl_3$)

177.4 (*C*(2)), 62.05 (*C*(5)), 48.2 (*C*(1′)), 32.0 (*C*(4)), 30.6 (*C*(3)), 27.61, 27.59, 26.6, 26.51, 26.49, 24.4 (*C*(2′), *C*(3′), *C*(4′), *C*(5′), *C*(6′), C(5)*Me*)

<u>IR:</u> (neat)

3178 (w), 3066 (w), 2971 (w), 2931 (m), 2854 (m), 1690 (m), 1443 (m), 1429 (m), 1380 (m), 1351 (w), 1317 (w), 1302 (w), 1271 (w), 1253 (w), 1216 (m), 1153 (w), 1133 (w), 1075 (w), 1032 (w), 1003 (w), 986 (w), 949 (w), 937 (w), 923 (w), 914 (w), 891 (w), 945 (w), 925 (w), 794 (m), 767 (m), 733 (w), 696 (w)

<u>MS:</u> (ESI^+)

385 (21%), 363 (21%), 242 (10%), 204 ($[M+Na]^+$, 65%), 183 (11%), 182 ($[M+H]^+$, 100%)

<u>HRMS:</u> (ESI^+)

calcd for $C_{11}H_{20}NO$: 182.1539, found: 182.1545

Preparation of (*RS*)-5-(adamantan-1-yl)-5-methylpyrrolidin-2-one (17h)

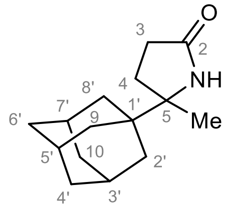

17h

Following *General Procedure 1*, (*RS*)-1-(adamantan-1-yl)ethan-1-amine [Rimantidine] **1h** (80 mg, 0.45 mmol, 1.0 equiv), methyl acrylate **14** (41 µL, 39 mg, 0.45 mmol, 1.0 equiv), 4CzIPN (2.28 mM in MeCN, 1.98 mL, 4.5 µmol, 1 mol%), tetrabutylammonium azide (70.3 mM in MeCN, 640 µL, 45 µmol, 10 mol%), and MeCN (390 µL) were reacted for 20 h with a 425 nm LED lamp. Following concentration *in vacuo*, the residue was dissolved in MeOH (5 mL) and Et_3N (0.25 mL, 0.18 g, 1.8 mmol, 4.0 equiv) was added. The reaction was then heated at reflux for 2 h, followed by concentration *in vacuo*. Purification *via* flash column chromatography on silica gel (12 g) in CH_2Cl_2 (5 CV) then 100:0:0→90:9:1 CH_2Cl_2–MeOH–aq. NH_4OH (over 15 CV) then 90:9:1 CH_2Cl_2–MeOH–aq. NH_4OH (17 CV), followed by reversed-phase flash column chromatography on C_{18} silica gel (12 g) in H_2O (3 CV) then 100:0→0:100 H_2O–MeOH (over 15 CV) then MeOH (16 CV), gave **17h** as a white crystalline solid (41.7 mg, 40%). The NMR spectroscopic data was in accordance with the literature.[13]

Data for **17h**:

mp: 166–168 °C {lit.[13] 168 °C (Et_2O)}

^{1}H NMR: (500 MHz, $CDCl_3$)

6.61 (br s, 1H, N*H*), 2.45–2.17 (m, 3H, C(3)H_2, C(4)H_A), 2.00 (m, 3H, C(3′)*H*, C(5′)*H*, C(7′)*H*), 1.72–1.42 (m, 13H, C(4)H_B, C(2′)H_2, C(4′)H_2, C(6′)H_2, C(8′)H_2, C(9′)H_2, C(10′)H_2), 1.19 (s, 3H, C(6)H_2)

^{13}C NMR: (126 MHz, $CDCl_3$)

177.6 (*C*(2)), 64.6 (*C*(5)), 38.6 (*C*(1′), 37.0 and 36.2 (*C*(2′), *C*(4′), *C*(6′), *C*(8′), *C*(9′), *C*(10′)), 30.95 (*C*(3)), 28.45 (*C*(3′), *C*(5′), *C*(7′)), 28.35 (*C*(4)), 23.3 (*C*(6))

IR: (neat)

3187 (w), 3066 (w), 2901 (m), 2846 (w), 2679 (w), 1681 (s), 1472 (w), 1449 (w), 1422 (w), 1384 (m), 1342 (w), 1306 (w), 1264 (w), 1234 (w), 1205 (w), 1160 (w), 1129 (w), 1105 (w), 1094 (w), 1065 (w), 997 (w), 985 (w), 972 (w), 912 (w), 805 (w), 773 (w), 713 (w), 657 (w)

MS: (ESI^+)

361 (14%), 192 ($[M+Na]^+$, 100%), 170 ($[M+H]^+$, 72%)

HRMS: (ESI^+)

calcd for $C_{15}H_{24}NO$: 234.1852, found: 234.1855

Preparation of (*RS*)-5-methyl-5-phenethylpyrrolidin-2-one (17j)

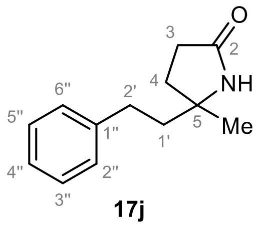

17j

Following *General Procedure 1*, (*RS*)-4-phenylbutan-2-amine **1j** (72 µL, 67 mg, 0.45 mmol, 1.0 equiv), methyl acrylate **14** (41 µL, 39 mg, 0.45 mmol, 1.0 equiv), 4CzIPN (2.28 mM in MeCN, 1.98 mL, 4.5 µmol, 1 mol%), tetrabutylammonium azide (70.3 mM in MeCN, 640 µL, 45 µmol, 10 mol%), and MeCN (390 µL) were reacted for 20 h with a 425 nm LED lamp. Following concentration *in vacuo*, the residue was dissolved in MeOH (5 mL) and Et_3N (0.25 mL, 0.18 g, 1.8 mmol, 4.0 equiv) was added. The reaction was then heated at reflux for 2 h, followed by concentration *in vacuo*. Purification *via* flash column chromatography on silica gel (12 g) in CH_2Cl_2 (5 CV) then 100:0:0→90:9:1 CH_2Cl_2–MeOH–aq. NH_4OH (over 15 CV) then 90:9:1

CH_2Cl_2–MeOH–aq. NH_4OH (17 CV), followed by reversed-phase flash column chromatography on C_{18} silica gel (12 g) in H_2O (3 CV) then 100:0→0:100 H_2O–MeOH (over 15 CV) then MeOH (16 CV), gave **17j** as a pale cream crystalline solid (77.1 mg, 84%).

Data for **17j**:

mp: 100–102 °C

^{1}H NMR: (500 MHz, $CDCl_3$)
7.36–7.25 (m, 2H, C(6″)*H*, C(2″)*H*), 7.22–7.13 (m, 3H, C(5″)*H*, C(3″)*H*, C(4″)*H*), 6.15 (br s, 1H, N*H*), 2.77–2.54 (m, 2H, C(3)H_2), 2.54–2.36 (m, 2H, C(4)H_2), 2.14–2.00 (m, 1H, C(2′)H_2), 2.00–1.77 (m, 3H, C(2′)H_2, C(1′)H_2), 1.35 (s, 3H, C(6)H_3)

^{13}C NMR: (126 MHz, $CDCl_3$)
177.2 (*C*(2)), 141.55 (*C*(1″), 128.7 (*C*(6″), *C*(2″)), 128.4 (*C*(4″), 126.2 (*C*(5″), *C*(3″)), 59.2 (*C*(5)), 44.2 (*C*(1′), 33.75 (*C*(2′)), 30.9 (*C*(3)), 30.5 (*C*(4)), 27.4 (*C*(6))

IR: (neat)
3653 (w), 3149 (w), 3062 (w), 3025 (w), 2981 (m), 2968 (m), 2931 (w), 2897 (w), 1952 (w), 1679 (s), 1602 (w), 1497 (w), 1453 (m), 1439 (w), 1419 (w), 1383 (m), 1349 (w), 1312 (w), 1284 (w), 1269 (w), 1239 (m), 1220 (m), 1170 (w), 1110 (w), 1066 (w), 1036 (w), 1001 (w), 958 (w), 906 (w), 820 (m), 785 (m), 748 (s), 698 (s), 668 (w)

MS: (ESI^+)
429 (43%), 226 ($[M+Na]^+$, 84%), 204 ($[M+H]^+$, 100%)

HRMS: (ESI^+)
calcd for $C_{13}H_{18}NO$: 204.1383, found: 204.1387

Preparation of (1*RS*,2*SR*,4*SR*)-spiro[bicyclo[2.2.1]heptane-2,2-pyrrolidin]-5-one (17k)

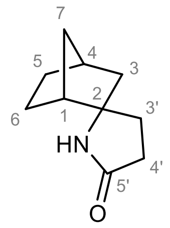

17k

Following *General Procedure 1*, *exo*-2-aminonorbornane **1k** (55 µL, 51 mg, 0.45 mmol, 1.0 equiv), methyl acrylate **14** (41 µL, 39 mg, 0.45 mmol, 1.0 equiv), 4CzIPN (2.28 mM in MeCN, 1.98 mL,

4.5 µmol, 1 mol%), tetrabutylammonium azide (70.3 mM in MeCN, 640 µL, 45 µmol, 10 mol%), and MeCN (390 µL) were reacted for 20 h with a 425 nm LED lamp. Following concentration *in vacuo*, the residue was dissolved in MeOH (5 mL) and Et_3N (0.25 mL, 0.18 g, 1.8 mmol, 4.0 equiv) was added. The reaction was then heated at reflux for 2 h, followed by concentration *in vacuo*. Purification *via* flash column chromatography on silica gel (12 g) in CH_2Cl_2 (5 CV) then 100:0:0→90:9:1 CH_2Cl_2–MeOH–aq. NH_4OH (over 15 CV) then 90:9:1 CH_2Cl_2–MeOH–aq. NH_4OH (17 CV), followed by reversed-phase flash column chromatography on C_{18} silica gel (50 g, 20 mm Ø) in 70:30 MeOH–H_2O, gave **17k** as a white crystalline solid (48.4 mg, 64%, >99:1 dr).

Data for **17k**:

mp: 167–169 °C

^{1}H NMR: (500 MHz, $CDCl_3$)
6.64 (s, 1H, N*H*), 2.47–2.25 (m, 2H, C(4′)H_2), 2.25–2.20 (m, 1H, C(4)*H*), 2.18–2.05 (m, 2H, C(1)*H*, C(3′)H_A), 1.94–1.83 (m, 1H, C(3′)H_B), 1.76–1.68 (m, 1H, C(3)H_A), 1.64–1.43 (m, 4H, C(5)H_A, C(6)H_A, C(7)H_2), 1.38–1.28 (m, 2H, C(3)H_B), 1.28–1.20 (m, 1H, C(5)H_B)

^{13}C NMR: (126 MHz, $CDCl_3$)
177.7 (*C*(5′)), 65.4 (*C*(2)), 46.9 (*C*(1)), 46.6 (*C*(3)), 37.9 (*C*(6)), 37.8 (*C*(3′)), 36.75 (*C*(4)), 30.9 (*C*(4′)), 28.6 (*C*(5)), 23.35 (*C*(7))

IR: (neat)
3173 (w), 3072 (w), 2940 (w), 2868 (w), 1696 (m), 1483 (w), 1440 (w), 1371 (m), 1308 (w), 1291 (w), 1248 (w), 1233 (w), 1216 (w), 1193 (w), 1173 (w), 1141 (w), 1121 (w), 1104 (w), 1072 (w), 1061 (w), 1044 (w), 1018 (w), 986 (w), 963 (w), 937 (w), 923 (w), 903 (w), 880 (w), 845 (w)

MS: (ESI^+)
353 (11%), 188 ($[M+Na]^+$, 100%), 166 ($[M+H]^+$, 62%)

HRMS: (ESI^+)
calcd for $C_{10}H_{16}NO$: 166.1226, found: 166.1229

Preparation of 1,4-dioxa-9-azadispiro[4.2.4^8.2^5]tetradecan-10-one (17l)

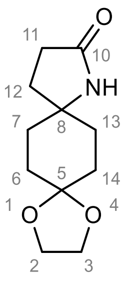

17l

Following *General Procedure 1*, 1,4-dioxaspiro[4.5]decan-8-amine **1l** (64 µL, 71 mg, 0.45 mmol, 1.0 equiv), methyl acrylate **14** (41 µL, 39 mg, 0.45 mmol, 1.0 equiv), 4CzIPN (2.28 mM in MeCN, 1.98 mL, 4.5 µmol, 1 mol%), tetrabutylammonium azide (70.3 mM in MeCN, 640 µL, 45 µmol, 10 mol%), and MeCN (390 µL) were reacted for 20 h with a 425 nm LED lamp. Following concentration *in vacuo*, the residue was dissolved in MeOH (5 mL) and Et_3N (0.25 mL, 0.18 g, 1.8 mmol, 4.0 equiv) was added. The reaction was then heated at reflux for 2 h, followed by concentration *in vacuo*. Purification *via* flash column chromatography on silica gel (12 g) in CH_2Cl_2 (5 CV) then 100:0:0→90:9:1 CH_2Cl_2–MeOH–aq. NH_4OH (over 15 CV) then 90:9:1 CH_2Cl_2–MeOH–aq. NH_4OH (17 CV), followed by reversed-phase flash column chromatography on C_{18} silica gel (15.5 g) in H_2O (3 CV) then 100:0→0:100 H_2O–MeOH (over 15 CV) then MeOH (16 CV), gave **17l** as a white crystalline solid (59.1 mg, 62%). The NMR spectroscopic data was in accordance with the literature.[14]

Data for **17l**:

mp: 203–205 °C

^{1}H NMR: (500 MHz, $CDCl_3$)
6.48 (br s, 1H, N*H*), 3.97–3.91 (m, C(2)H_2, C(3)H_2), 2.40 (t, J = 8.1 Hz, 2H, C(11)H_2), 1.97 (t, J = 8.1 Hz, 2H, C(12)H_2), 1.78–1.68 (m, 8H, C(6)H_2, C(7)H_2, C(13)H_2, C(14)H_2)

^{13}C NMR: (126 MHz, $CDCl_3$)
178.3 (*C*(10)), 108.9 (*C*(5)), 65.6 (*C*(2), *C*(3)), 58.5 (*C*(8)), 36.7 (*C*(7), *C*(13)), 33.3 (*C*(6), *C*(14)), 31.9 (*C*(12)), 31.0 (*C*(11))

IR: (neat)
3166 (w), 3077 (w), 2937 (w), 2888 (w), 1693 (m), 1442 (w), 1375 (m), 1348 (w), 1328 (w), 1264 (w), 1198 (w), 1172 (w), 1158 (w), 1105 (m), 1091 (m), 1036 (w), 995 (w), 972 (w), 933 (w), 883 (w), 829 (w), 810 (w), 760 (m), 669 (w)

MS: (ESI+)

235 ([M+Na]+, 12%), 234 (100%), 213 ([M+H]+, 14%), 212 (84%)

HRMS: (ESI+)

calcd for $C_{11}H_{17}NO_3{}^{23}Na$: 234.1101, found 234.1105

Preparation of 8,8-dimethyl-7,9-dioxa-1-azaspiro[4.5]decan-2-one (17m)

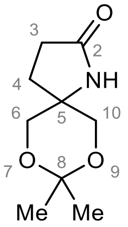

17m

Following *General Procedure 1*, 2,2-dimethyl-1,3-dioxan-5-amine **1m** (59 mg, 0.45 mmol, 1.0 equiv), methyl acrylate **14** (41 µL, 39 mg, 0.45 mmol, 1.0 equiv), 4CzIPN (2.28 mM in MeCN, 1.98 mL, 4.5 µmol, 1 mol%), tetrabutylammonium azide (70.3 mM in MeCN, 640 µL, 45 µmol, 10 mol%), and MeCN (390 µL) were reacted for 20 h with a 425 nm LED lamp. Following concentration *in vacuo*, the residue was dissolved in MeOH (5 mL) and Et_3N (0.25 mL, 0.18 g, 1.8 mmol, 4.0 equiv) was added. The reaction was then heated at reflux for 2 h, followed by concentration *in vacuo*. Purification *via* flash column chromatography on silica gel (12 g) in CH_2Cl_2 (5 CV) then 100:0:0→90:9:1 CH_2Cl_2–MeOH–aq. NH_4OH (over 15 CV) then 90:9:1 CH_2Cl_2–MeOH–aq. NH_4OH (17 CV), followed by followed by reversed-phase flash column chromatography on C_{18} silica gel (15.5 g) in H_2O (3 CV) then 100:0→0:100 H_2O–MeOH (over 15 CV) then MeOH (16 CV), gave **17m** as a white crystalline solid (45.0 mg, 54%). The NMR spectroscopic data was in accordance with the literature.[15]

Data for **17m**:

mp: 154–156 °C {lit.[15] 174 °C}

^{1}H NMR: (500 MHz, $CDCl_3$)

6.63 (br s, 1H, N*H*), 3.85–3.79 (m, 2H, C(6)H_A, C(10)H_A), 3.66–3.60 (m, 2H, C(6)H_B, C(10)H_B), 2.40 (t, J = 8.2 Hz, 2H, C(3)H_2), 1.77 (t, J = 8.6 Hz, 2H, C(4)H_2) 1.45 (s, 3H, C(8)Me_A), 1.41 (s, 3H, C(8)Me_B)

^{13}C NMR: (126 MHz, $CDCl_3$)

176.3 (*C*(2)), 98.6 (*C*(8)), 68.7 (*C*(6), *C*(10)), 55.9 (*C*(5)), 31.1 (*C*(3)), 29.25

(C(8)Me_A), 27.5 (*C*(4)), 19.8 (C(8)Me_B)

IR: (neat)

3363 (w), 3186 (w), 2995 (w), 2924 (w), 2878 (w), 1706 (m), 1650 (m), 1465 (w), 1455 (w), 1422 (w), 1393 (w), 1378 (w), 1305 (w), 1281 (w), 1262 (w), 1196 (m), 1153 (w), 1118 (w), 1072 (m), 1050 (m), 1036 (m), 1006 (w), 938 (w), 830 (m), 768 (m), 728 (m), 676 (w), 662 (w)

MS: (ESI^+)

208 ($[M+Na]^+$, 100%), 186 ($[M+H]^+$, 56%)

HRMS: (ESI^+)

calcd for $C_9H_{16}NO_3$: 186.1125, found 186.1126

Preparation of 8-hydroxy-1-azaspiro[4.5]decan-2-one (17n)

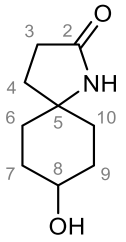

17n

Following *General Procedure 1*, 4-aminocyclohexanol **1n** (53 mg, 0.45 mmol, 1.0 equiv, ~1:1 dr), methyl acrylate **14** (41 µL, 39 mg, 0.45 mmol, 1.0 equiv), 4CzIPN (2.28 mM in MeCN, 1.98 mL, 4.5 µmol, 1 mol%), tetrabutylammonium azide (70.3 mM in MeCN, 640 µL, 45 µmol, 10 mol%), and MeCN (390 µL) were reacted for 20 h with a 425 nm LED lamp. Following concentration *in vacuo*, the residue was dissolved in MeOH (5 mL) and Et_3N (0.25 mL, 0.18 g, 1.8 mmol, 4.0 equiv) was added. The reaction was then heated at reflux for 2 h, followed by concentration *in vacuo*. 1H NMR analysis indicated a 59:42 ratio of **17n** to unreacted amine **1n**. Purification *via* flash column chromatography on silica gel (12 g) in CH_2Cl_2 (5 CV) then 100:0:0→90:9:1 CH_2Cl_2–MeOH–aq. NH_4OH (over 15 CV) then 90:9:1 CH_2Cl_2–MeOH–aq. NH_4OH (17 CV), followed by reversed-phase flash column chromatography on C_{18} silica gel (50 g, 20 mm Ø) in 80:20 MeOH–H_2O, gave **17n** as a cream solid (24.5 mg, 32%, 54:46 dr).

Data for **17n** (both diastereomers):

^{1}H NMR: (500 MHz, $CDCl_3$)

7.05 (s, N*H* for diastereomer 1), 6.58 (s, N*H* for diastereomer 2), 3.87–3.61 (m, 1H,

C(8)*H* for both diastereomers), 2.43–2.35 (m, 2H, C(3)H_2 for both diastereomers), 2.03–1.75 (m, 7H, O*H*, C(4)H_2, C(6)H_2, C(10)H_2 for both diastereomers), 1.60–1.44 (m, 4H, C(7)H_2, C(9)H_2 for both diastereomers)

^{13}C NMR: (126 MHz, $CDCl_3$)

177.6 (*C*(2) for diastereomer 1 or 2), 177.35 (*C*(2) for diastereomer 1 or 2), 68.3 (*C*(8) for diastereomer 1 or 2), 68.1 (*C*(8) for diastereomer 1 or 2), 58.9 (*C*(5) for diastereomer 1 or 2), 58.6 (*C*(5) for diastereomer 1 or 2), 35.4 (*C*(6), *C*(10) for diastereomer 1 or 2), 35.0 (*C*(6), *C*(10) for diastereomer 1 or 2)), 33.20 (*C*(7), *C*(9) for diastereomer 1 or 2), 33.19 (*C*(7), *C*(9) for diastereomer 1 or 2), 31.6 (*C*(4) for diastereomer 1 or 2), 31.4 (*C*(4) for diastereomer 1 or 2), 30.02 (*C*(3) for diastereomer 1 or 2), 29.99 (*C*(3) for diastereomer 1 or 2)

IR: (neat)

3285 (w), 3236 (m), 2983 (w), 2940 (m), 2857 (w), 1658 (s), 1452 (m), 1426 (m), 1368 (m), 1342 (m), 1317 (w), 1302 (m), 1285 (w), 1248 (w), 1210 (m), 1182 (w), 1153 (w), 1138 (w), 1115 (w), 1081 (m), 1047 (m), 1029 (m), 986 (w), 955 (m), 946 (w), 920 (m), 831 (w), 811 (w), 802 (w), 765 (m), 719 (m), 676, (m) 662 (m)

MS: (ESI^+)

362 (6%), 339 (34%), 192 ($[M+Na]^+$, 100%), 170 ($[M+H]^+$, 85%)

HRMS: (ESI^+)

calcd for $C_9H_{16}NO_2$: 170.1176, found: 170.1179

Preparation of 8-oxa-1-azaspiro[4.5]decan-2-one (17o)

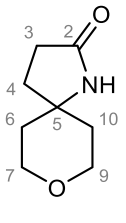

17o

Following *General Procedure 1*, 4-aminotetrahydrofuran **1o** (47 µL, 46 mg, 0.45 mmol, 1.0 equiv), methyl acrylate **14** (41 µL, 39 mg, 0.45 mmol, 1.0 equiv), 4CzIPN (2.28 mM in MeCN, 1.98 mL, 4.5 µmol, 1 mol%), tetrabutylammonium azide (70.3 mM in MeCN, 640 µL, 45 µmol, 10 mol%), and MeCN (390 µL) were reacted for 20 h with a 425 nm LED lamp. Following concentration *in vacuo*, the residue was dissolved in MeOH (5 mL) and Et_3N (0.25 mL, 0.18 g, 1.8 mmol, 4.0 equiv)

was added. The reaction was then heated at reflux for 2 h, followed by concentration *in vacuo*. Purification *via* flash column chromatography on silica gel (12 g) in CH_2Cl_2 (5 CV) then 100:0:0→90:9:1 CH_2Cl_2–MeOH–aq. NH_4OH (over 15 CV) then 90:9:1 CH_2Cl_2–MeOH–aq. NH_4OH (17 CV), followed by reversed-phase flash column chromatography on C_{18} silica gel (50 g, 20 mm Ø) in 80:20 MeOH–H_2O, gave **17o** as a cream solid (41 mg, 58%).

Data for **17o**:

mp: 168–170 °C

^{1}H NMR: (500 MHz, $CDCl_3$)
6.45 (s, 1H, N*H*), 3.78–3.64 (m, 4H, C(7)H_2, C(9)H_2), 2.42 (t, *J* = 8.0 Hz, 2H, C(3)H_2), 2.00 (t, *J* = 8.0 Hz, 2H, C(4)H_2), 1.71 (app t, *J* = 5.4 Hz, 4H, C(6)H_2, C(10)H_2)

^{13}C NMR: (126 MHz, $CDCl_3$)
177.0 (*C*(2)), 64.9 (*C*(7), *C*(9)), 56.8 (*C*(5)), 38.6 (*C*(6), *C*(10)), 32.55 (*C*(4)), 29.6 (*C*(3))

IR: (neat)
3178 (w), 3075 (w), 2960 (w), 2931 (w), 2917 (w), 2857 (m), 2759 (w), 2722 (w), 2699 (w), 1733 (w), 1670 (s), 1549 (w), 1475 (w), 1455 (m), 1440 (m), 1429 (m), 1374 (m), 1348 (w), 1311 (w), 1299 (w), 1271 (m), 1248 (w), 1219 (m), 1202 (w), 1184 (w), 1170 (m), 1121 (w), 1101 (s), 1090 (m), 1024 (m), 1012 (m), 978 (w), 957 (m), 926 (w), 909 (w), 843 (m), 820 (m), 768 (s), 659 (m)

MS: (ESI$^+$)
311 (13%), 242 (24%), 178 (M+Na]$^+$, 59%), 156 ([M+H]$^+$, 100%), 142 (15%)

HRMS: (ESI$^+$)
calcd for $C_8H_{14}NO_2$: 156.1019, found: 156.1014

Preparation of 2-oxa-5-azaspiro[3.4]octan-6-one (17p)

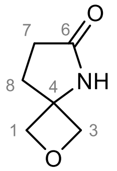

17p

Following *General Procedure 1*, oxetan-3-amine **1p** (32 μL, 33 mg, 0.45 mmol, 1.0 equiv), methyl acrylate **14** (41 μL, 39 mg, 0.45 mmol, 1.0 equiv), 4CzIPN (2.28 mM in MeCN, 1.98 mL, 4.5

µmol, 1 mol%), tetrabutylammonium azide (70.3 mM in MeCN, 640 µL, 45 µmol, 10 mol%), and MeCN (390 µL) were reacted for 20 h with a 425 nm LED lamp. Following concentration *in vacuo*, the residue was dissolved in MeCN (5 mL) and Et_3N (0.25 mL, 0.18 g, 1.8 mmol, 4.0 equiv) was added. The reaction was then heated at reflux for 12 h, followed by concentration *in vacuo*. Purification *via* flash column chromatography on silica gel (12 g) in CH_2Cl_2 (5 CV) then 100:0:0→90:9:1 CH_2Cl_2–MeOH–aq. NH_4OH (over 15 CV) then 90:9:1 CH_2Cl_2–MeOH–aq. NH_4OH (17 CV), followed by reversed-phase flash column chromatography on C_{18} silica gel (15.5 g) in H_2O (3 CV) then 100:0→0:100 H_2O–MeOH (over 15 CV) then MeOH (16 CV) to give **17p** as a white crystalline solid (14.8 mg, 26%).

Data for **17p**:

mp: 129–131 °C

^{1}H NMR: (500 MHz, $CDCl_3$)
7.24 (br s, 1H, N*H*), 4.76 (d, *J* = 7.3 Hz, 2H, C(1)H_A, C(3)H_A), 4.65 (d, *J* = 6.9 Hz, 2H, C(1)H_B, C(3)H_B), 2.49–2.43 (m, 2H, C(7)H_2), 2.41–2.35 (m, 2H, C(8)H_2)

^{13}C NMR: (126 MHz, $CDCl_3$)
177.3 (*C*(6)), 84.0 (*C*(1), *C*(3)), 60.6 (*C*(4)), 32.4 (*C*(7)), 30.1 (*C*(8))

IR: (neat)
3167 (w), 3095 (w), 2951 (w), 2870 (w), 2212 (w), 1702 (m), 1661 (s), 1460 (m), 1426 (w), 1370 (m), 1342 (w), 1298 (w), 1252 (m), 1146 (w), 972 (s), 935 (m), 837 (m), 756 (m)

MS: (ESI$^+$)
242 (38%), 128 ([M+H]$^+$, 100%)

HRMS: (ESI$^+$)
calcd for $C_6H_{10}NO_2$: 128.0706, found: 128.0707

Preparation of *tert*-butyl 2-oxo-1,8-diazaspiro[4.5]decane-8-carboxylate (17q)

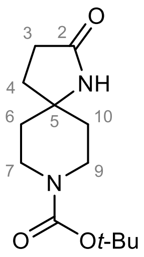

17q

Following *General Procedure 1*, *tert*-butyl 4-aminopiperidine-1-carboxylate **1q** (92 mg, 0.45 mmol, 1.0 equiv), methyl acrylate **14** (41 µL, 39 mg, 0.45 mmol, 1.0 equiv), 4CzIPN (2.28 mM in MeCN, 1.98 mL, 4.5 µmol, 1 mol%), tetrabutylammonium azide (70.3 mM in MeCN, 640 µL, 45 µmol, 10 mol%), and MeCN (390 µL) were reacted for 20 h with a 425 nm LED lamp. Following concentration *in vacuo*, the residue was dissolved in MeOH (5 mL) and Et_3N (0.25 mL, 0.18 g, 1.8 mmol, 4.0 equiv) was added. The reaction was then heated at reflux for 2 h, followed by concentration *in vacuo*. Purification *via* flash column chromatography on silica gel (12 g) in CH_2Cl_2 (5 CV) then 100:0:0→90:9:1 CH_2Cl_2–MeOH–aq. NH_4OH (over 15 CV) then 90:9:1 CH_2Cl_2–MeOH–aq. NH_4OH (17 CV), followed by reversed-phase flash column chromatography on C_{18} silica gel (50 g, 20 mm Ø) in 80:20 MeOH–H_2O, gave **17q** as a white crystalline solid (65 mg, 56%).

Data for **17q**:

mp: 156–158 °C

^{1}H NMR: (500 MHz, $CDCl_3$)
7.32 (s, 1H, N*H*), 3.54–3.34 (m, 4H, C(7)H_2, C(9)H_2), 2.38 (t, J = 8.1 Hz, 2H, C(3)H_2), 1.91 (t, J = 8.1 Hz, 2H, C(4)H_2), 1.73–1.49 (m, 4H, C(6)H_2, C(10)H_2), 1.42 (s, 9H, O*t-Bu*)

^{13}C NMR: (126 MHz, $CDCl_3$)
177.5 (*C*(2)), 154.65 (N*C*=O), 79.9 (O*C*Me$_3$), 57.9 (*C*(5)), 41.1 (*C*(7), *C*(9)), 37.7 (*C*(6), *C*(10)), 32.4 (*C*(4)), 29.8 (*C*(3)), 28.55 (OC*Me*$_3$)

IR: (neat)
3173 (w), 3075 (w), 2974 (w), 2957 (w), 2934 (w), 2903 (w), 2874 (w), 1736 (w), 1673 (s), 1684 (s), 1538 (w), 1477 (w), 1455 (w), 1423 (m), 1386 (m), 1363 (m), 1296 (w), 1274 (m), 1242 (m), 1179 (m), 1153 (s), 1081 (m), 1026 (m), 995 (w), 978 (w), 955 (m), 937 (w), 914 (w), 868 (w), 820 (w), 782 (m), 762 (m), 736 (w), 667

(w)

MS: (ESI$^+$)

509 (16%), 277 (22%), 276 (12%), 255 ([M+H]$^+$, 25%), 243 (17%), 242 (100%), 199 (64%), 155 (12%), 133 (19%)

HRMS: (ESI$^+$)

calcd for $C_{13}H_{23}N_2O_3$: 255.1703, found: 255.1711

Preparation of *tert*-butyl (*RS*)-2-oxo-1,7-diazaspiro[4.5]decane-7-carboxylate (17r)

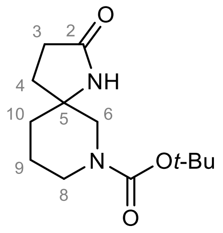

17r

Following *General Procedure 1*, *tert*-butyl (*RS*)-3-aminopiperidine-1-carboxylate **1r** (92 mg, 0.45 mmol, 1.0 equiv), methyl acrylate **14** (41 µL, 39 mg, 0.45 mmol, 1.0 equiv), 4CzIPN (2.28 mM in MeCN, 1.98 mL, 4.5 µmol, 1 mol%), tetrabutylammonium azide (70.3 mM in MeCN, 640 µL, 45 µmol, 10 mol%), and MeCN (390 µL) were reacted for 20 h with a 425 nm LED lamp. Following concentration *in vacuo*, the residue was dissolved in MeOH (5 mL) and Et_3N (0.25 mL, 0.18 g, 1.8 mmol, 4.0 equiv) was added. The reaction was then heated at reflux for 2 h, followed by concentration *in vacuo*. Purification *via* flash column chromatography on silica gel (12 g) in CH_2Cl_2 (5 CV) then 100:0:0→90:9:1 CH_2Cl_2–MeOH–aq. NH_4OH (over 15 CV) then 90:9:1 CH_2Cl_2–MeOH–aq. NH_4OH (17 CV), followed by reversed-phase flash column chromatography on C_{18} silica gel (50 g, 20 mm Ø) in 80:20 MeOH–H_2O, gave **17r** as a colourless oil (61 mg, 52%).

Data for **17r**:

^{1}H NMR: (500 MHz, $CDCl_3$)

6.03 (s, 1H, N*H*), 3.41 (d, *J* = 13.0 Hz, 1H, C(6)H_A), 3.22 (d, *J* = 13.2 Hz, 1H, C(6)H_B), 2.50–2.35 (m, 2H, C(8)H_2), 1.89–1.55 (m, 8H, C(3)H_2, C(4)H_2, C(9)H_2, C(10)H_2), 1.45 (s, 9H, O*tBu*)

^{13}C NMR: (126 MHz, $CDCl_3$)

177.5 (*C*(2)), 154.7 (N*C*=O), 80.0 (O*C*Me_3), 58.0 (*C*(5)), 36.9 (*C*(8)), 30.0 (*C*(6)), 29.8 (OCMe_3), 28.5 (*C*(4)), 22.3 (*C*(9)), 21.1 (*C*(3)), 14.3 (*C*(10))

IR: (neat)

3247 (w), 2974 (w), 2934 (w), 2859 (w), 1679 (s), 1532 (w), 1455 (w), 1420 (m), 1391 (w), 1363 (m), 1345 (w), 1271 (m), 1248 (m), 1213 (w), 1153 (s), 1078 (w), 1032 (w), 1003 (w), 966 (w), 926 (w), 897 (w), 868 (w), 825 (w), 762 (m), 699 (w), 662 (w)

MS: (ESI^+)

531 (43%), 278 (14%), 277 ($[M+Na]^+$, 100%), 199 (42%)

HRMS: (ESI^+)

calcd for $C_{13}H_{23}N_2O_3$: 255.1703, found: 255.1698

Preparation of *tert*-butyl 6-oxo-2,5-diazaspiro[3.4]octane-2-carboxylate (17s)

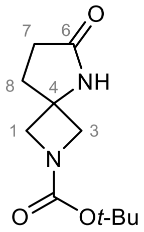

17s

Following *General Procedure 1*, *tert*-butyl 3-aminoazetidine-1-carboxylate **1s** (71 µL, 77 mg, 0.45 mmol, 1.0 equiv), methyl acrylate **14** (41 µL, 39 mg, 0.45 mmol, 1.0 equiv), 4CzIPN (2.28 mM in MeCN, 1.98 mL, 4.5 µmol, 1 mol%), tetrabutylammonium azide (70.3 mM in MeCN, 640 µL, 45 µmol, 10 mol%), and MeCN (390 µL) were reacted for 20 h with a 425 nm LED lamp. Following concentration *in vacuo*, the residue was dissolved in MeOH (5 mL) and Et_3N (0.25 mL, 0.18 g, 1.8 mmol, 4.0 equiv) was added. The reaction was then heated at reflux for 2 h, followed by concentration *in vacuo*. Purification *via* flash column chromatography on silica gel (12 g) in CH_2Cl_2 (5 CV) then 100:0:0→90:9:1 CH_2Cl_2–MeOH–aq. NH_4OH (over 15 CV) then 90:9:1 CH_2Cl_2–MeOH–aq. NH_4OH (17 CV), followed by reversed-phase flash column chromatography on C_{18} silica gel (15.5 g) in H_2O (3 CV) then 100:0→0:100 H_2O–MeOH (over 15 CV) then MeOH (16 CV), gave **17s** as a white crystalline solid (40.3 mg, 40%).

Data for **17s**:

mp: 179–181 °C

^{1}H NMR: (500 MHz, $CDCl_3$)

6.85 (br s, 1H, N*H*), 4.05–3.95 (m, 4H, C(1)H_2, C(3)H_2), 2.43–2.31 (m, 4H, C(8)H_2,

C(7)*H*$_2$), 1.43 (s, 9H, C(O)(O*t*-*Bu*))

^{13}C NMR: (126 MHz, $CDCl_3$)

177.3 (*C*(6)), 156.2 (*C*(O)(O*t*-Bu)), 80.3 (C(O)(O*t*-*Bu*)), 62.6 (*C*(1), *C*(3)) 55.5 (*C*(4)), 33.5 (*C*(8)), 30.2 (*C*(7)), 28.5 (C(O)(O*t*-*Bu*))

IR: (neat)

3179 (w), 3093 (w), 2976 (w), 2927 (w), 2884 (w), 1996 (w), 1687 (m), 1544 (w), 1464 (w), 1450 (w), 1397 (m), 1371 (m), 1361 (m), 1299 (w), 1252 (w), 1219 (w), 1156 (m), 1086 (m), 949 (w), 932 (w), 863 (w), 770 (m)

MS: (ESI^+)

543 (24%), 249 ($[M+Na]^+$, 32%), 171 (100%),

HRMS: (ESI^+)

calcd for $C_{11}H_{18}N_2O_3{}^{23}Na$: 249.1210, found: 249.1216

Preparation of 8-thia-1-azaspiro[4.5]decan-2-one (17t)

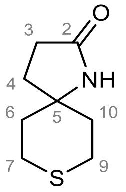

17t

Following *General Procedure 1*, tetrahydro-2*H*-thiopyran-4-amine hydrochloride **1t** (69.2 mg, 0.45 mmol, 1.0 equiv), methyl acrylate **14** (41 µL, 39 mg, 0.45 mmol, 1.0 equiv), 4CzIPN (2.28 mM in MeCN, 1.98 mL, 4.5 µmol, 1 mol%), tetrabutylammonium azide (70.3 mM in MeCN, 640 µL, 45 µmol, 10 mol%), Cs_2CO_3 (146 mg, 0.45 mmol, 1.0 equiv), and MeCN (780 µL) were reacted for 20 h with a 425 nm LED lamp. The reaction mixture was then filtered through a plug of cotton wool in a Pasteur pipette to remove Cs_2CO_3 residues, followed by rinsing with MeCN. Following concentration *in vacuo*, the residue was dissolved in MeOH (5 mL) and Et_3N (0.25 mL, 0.18 g, 1.8 mmol, 4.0 equiv) was added. The reaction was then heated at reflux for 2 h, followed by concentration *in vacuo*. Purification *via* flash column chromatography on silica gel (12 g) in CH_2Cl_2 (5 CV) then 100:0:0→90:9:1 CH_2Cl_2–MeOH–aq. NH_4OH (over 15 CV) then 90:9:1 CH_2Cl_2–MeOH–aq. NH_4OH (17 CV), followed by reversed-phase flash column chromatography on C_{18} silica gel (12 g) in H_2O (3 CV) then 100:0→0:100 H_2O–MeOH (over 15 CV) then MeOH (16 CV), gave **17t** as a white crystalline solid (50.0 mg, 65%).

<u>Data for **17t**:</u>

<u>mp:</u> 215–217 °C

<u>^{1}H NMR:</u> (500 MHz, $CDCl_3$)

6.93 (br s, 1H, N*H*), 2.75–2.59 (m, 4H, C(7)H_2, C(9)H_2), 2.43–2.37 (m, 2H, C(3)H_2), 1.94–1.86 (m, 6H, C(4)H_2, C(6)H_2, C(10)H_2)

<u>^{13}C NMR:</u> (126 MHz, $CDCl_3$)

177.2 (*C*(2)), 58.2 (*C*(5)), 39.4 (*C*(6), *C*(10)), 33.0 (*C*(4)), 29.65 (*C*(3)), 25.4 (*C*(7), *C*(9))

<u>IR:</u> (neat)

3185 (w), 2986 (w), 2959 (w), 2933 (w), 2901 (w), 2837 (w), 1687 (m), 1655 (m), 1459 (w), 1429 (m), 1413 (w), 1375 (m), 1343 (w), 1306 (w), 1271 (m), 1255 (m), 1238 (m), 1219 (m), 1193 (w), 1156 (w), 1143 (w), 1067 (w), 1027 (w), 985 (w), 956 (m), 938 (w), 926 (w), 906 (w), 822 (w), 803 (w), 755 (m), 696 (w)

<u>MS:</u> (ESI^+)

357 (11%), 194 ($[M+Na]^+$, 74%), 172 ($[M+H]^+$, 100%)

<u>HRMS:</u> (ESI^+)

calcd for $C_8H_{14}NOS$: 172.0791, found: 172.0793

Preparation of 8-thia-1-azaspiro[4.5]decan-2-one 8,8-dioxide (17u)

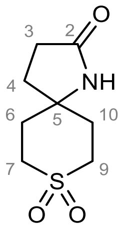

17u

Following *General Procedure 1*, 4-aminotetrahydro-2*H*-thiopyran 1,1-dioxide hydrochloride **1u** (83.5 mg, 0.45 mmol, 1.0 equiv), methyl acrylate **14** (41 µL, 39 mg, 0.45 mmol, 1.0 equiv), 4CzIPN (2.28 mM in MeCN, 1.98 mL, 4.5 µmol, 1 mol%), tetrabutylammonium azide (70.3 mM in MeCN, 640 µL, 45 µmol, 10 mol%), Cs_2CO_3 (146 mg, 0.45 mmol, 1.0 equiv), and MeCN (780 µL) were reacted for 20 h with a 425 nm LED lamp. The reaction mixture was then filtered through a plug of cotton wool in a Pasteur pipette to remove Cs_2CO_3 residues, followed by rinsing with MeCN. Following concentration *in vacuo*, the residue was dissolved in MeOH (5 mL) and Et_3N (0.25 mL, 0.18 g, 1.8 mmol, 4.0 equiv) was added. The reaction was then heated at reflux for 2 h, followed

by concentration *in vacuo*. Purification *via* flash column chromatography on silica gel (12 g) in CH_2Cl_2 (5 CV) then 100:0:0→90:9:1 CH_2Cl_2–MeOH–aq. NH_4OH (over 15 CV) then 90:9:1 CH_2Cl_2–MeOH–aq. NH_4OH (17 CV), followed by reversed-phase flash column chromatography on C_{18} silica gel (15.5 g) in H_2O (3 CV) then 100:0→0:100 H_2O–MeOH (over 15 CV) then MeOH (16 CV) to give **17u** as a white crystalline solid (53.1 mg, 58%).

Data for **17u**:

mp: 347–349 °C

^{1}H NMR: (500 MHz, DMSO-d_6)

8.26 (br s, 1H, N*H*), 3.27–3.01 (m, 4H, C(7)H_2, C(9)H_2), 2.22 (t, J = 8.0 Hz, 2H, C(3)H_2), 2.10–1.98 (m, 4H, C(6)H_2, C(10)H_2), 1.92 (t, J = 8.1 Hz, 2H, C(4)H_2)

^{13}C NMR: (126 MHz, DMSO-d_6)

175.9 (*C*(2)), 55.9 (*C*(5)), 47.3 (*C*(7), *C*(9)), 35.5 (*C*(6) *C*(10)), 31.6 (*C*(4)), 29.3 (*C*(3))

IR: (neat)

3180 (w), 2986 (w), 2950 (w), 2889 (w), 1699 (m), 1644 (m), 1464 (w), 1451 (w), 1435 (w), 1425 (w), 1401 (w), 1356 (w), 1330 (w), 1286 (m), 1273 (m), 1254 (m), 1244 (m), 1196 (w), 1161 (w), 1127 (s), 1100 (m), 1071 (m), 1031 (w), 991 (w), 983 (w), 944 (w), 927 (w), 849 (m), 825 (w), 790 (m), 748 (m), 709 (w), 682 (w), 660 (m)

MS: (ESI$^+$)

497 (74%), 441 (16%), 294 (35%), 204 ([M+H]$^+$, 100%)

HRMS: (ESI$^+$)

calcd for $C_8H_{14}NO_3S$: 204.0689, found: 204.0689

Preparation of (*RS*)-5-(hydroxymethyl)-5-methylpyrrolidin-2-one (17v)

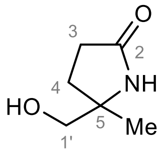

17v

Following *General Procedure 1*, (*RS*)-2-amino-1-propanol **1v** (36 µL, 34 mg, 0.45 mmol, 1.0 equiv), 2-methoxyethyl acrylate **15** (59 µL, 60 mg, 0.45 mmol, 1.0 equiv), 4CzIPN (2.28 mM in MeCN, 1.98 mL, 4.5 µmol, 1 mol%), tetrabutylammonium azide (70.3 mM in MeCN, 640 µL, 45

µmol, 10 mol%), and MeCN (390 µL) were reacted for 20 h with a 425 nm LED lamp. Following concentration *in vacuo*, the residue was dissolved in MeCN (5 mL) and Et_3N (0.25 mL, 0.18 g, 1.8 mmol, 4.0 equiv) was added. The reaction was then heated at reflux for 12 h, followed by concentration *in vacuo*. Purification *via* flash column chromatography on silica gel (12 g) in CH_2Cl_2 (5 CV) then 100:0:0→90:9:1 CH_2Cl_2–MeOH–aq. NH_4OH (over 15 CV) then 90:9:1 CH_2Cl_2–MeOH–aq. NH_4OH (17 CV), followed by reversed-phase flash column chromatography on C_{18} silica gel (15.5 g) in H_2O (3 CV) then 100:0→0:100 H_2O–MeOH (over 15 CV) then MeOH (16 CV), gave **17v** as a white solid (22.3 mg, 39%). The NMR spectroscopic data was in accordance with the literature.[16]

Data for **17v**:

mp: 94–96 °C {lit.[16] 82–83 °C}

^{1}H NMR: (500 MHz, $CDCl_3$)
6.91 (br s, 1H, N*H*), 3.52–3.41 (m, 2H, C(1′)H_2), 3.24 (br s, 1H, O*H*), 2.51–2.34 (m, 2H, C(3)H_2), 2.13–2.04 (m, 1H, C(4)H_A), 1.85–1.75 (m, 1H, C(4)H_B), 1.25 (s, 3H, C(5)*Me*)

^{13}C NMR: (126 MHz, $CDCl_3$)
178.7 (*C*(2)), 69.7 (*C*(1′)), 61.2 (*C*(5)), 31.1 (*C*(3)), 30.5 (*C*(4)), 24.3 (C(5)*Me*)

IR: (neat)
3246 (m), 2978 (w), 2970 (w), 2943 (w), 2100 (w), 1703 (m), 1674 (m), 1469 (w), 1454 (w), 1420 (m), 1392 (m), 1368 (m), 1288 (m), 1272 (m), 1244 (w), 1203 (w), 1145 (m), 1114 (w), 1052 (s), 1009 (w), 954 (w), 909 (w), 898 (w), 832 (w), 785 (w), 711 (m), 671 (s)

MS: (ESI^+)
281 (24%), 152 ($[M+Na]^+$, 100%), 130 ($[M+H]^+$, 28%)

HRMS: (ESI^+)
calcd for $C_6H_{11}NO_2{}^{23}Na$: 152. 0682, found: 152.0687

Preparation of (*RS*)-5-(methoxymethyl)-5-methylpyrrolidin-2-one (17w)

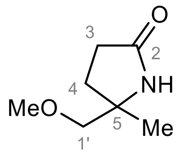

17w

Following *General Procedure 1*, (*RS*)-1-methoxy-2-propylamine **1w** (48 µL, 40 mg, 0.45 mmol, 1.0 equiv), 2-methoxyethyl acrylate **15** (59 µL, 60 mg, 0.45 mmol, 1.0 equiv), 4CzIPN (2.28 mM in MeCN, 1.98 mL, 4.5 µmol, 1 mol%), tetrabutylammonium azide (70.3 mM in MeCN, 640 µL, 45 µmol, 10 mol%), and MeCN (390 µL) were reacted for 20 h with a 425 nm LED lamp. Following concentration *in vacuo*, the residue was dissolved in MeCN (5 mL) and Et_3N (0.25 mL, 0.18 g, 1.8 mmol, 4.0 equiv) was added. The reaction was then heated at reflux for 12 h, followed by concentration *in vacuo*. Purification *via* flash column chromatography on silica gel (12 g) in CH_2Cl_2 (5 CV) then 100:0:0→90:9:1 CH_2Cl_2–MeOH–aq. NH_4OH (over 15 CV) then 90:9:1 CH_2Cl_2–MeOH–aq. NH_4OH (17 CV), followed by reversed-phase flash column chromatography on C_{18} silica gel (15.5 g) in H_2O (3 CV) then 100:0→0:100 H_2O–MeOH (over 15 CV) then MeOH (16 CV), gave **17w** as a pale yellow oil (29.6 mg, 46%).

Data for **17w**:

^{1}H NMR: (500 MHz, $CDCl_3$)
6.22 (br s, 1H, N*H*), 3.34 (s, 3H, O*Me*), 3.26–3.18 (m, 2H, C(1′)H_2), 2.45–2.32 (m, 2H, C(3)H_2), 2.02–1.93 (m, 1H, C(4)H_A), 1.85–1.76 (m, 1H, C(4)H_B), 1.26 (s, 3H, C(5)*Me*)

^{13}C NMR: (126 MHz, $CDCl_3$)
177.45 (*C*(2)), 80.1 (*C*(1′)), 59.5 (O*Me*), 59.3 (*C*(5)), 31.0 (*C*(4)), 30.4 (*C*(3)), 24.9 (C(5)*Me*)

IR: (neat)
3244 (w), 2973 (w), 2932 (w), 2876 (w), 2063 (w), 1683 (s), 1459 (w), 1419 (w), 1370 (w), 1271 (w), 1203 (w), 1184 (w), 1149 (w), 1104 (m), 973 (w), 669 (m)

MS: (ESI$^+$)
309 (35%), 287 (25%), 166 ([M+Na]$^+$, 100%), 144 ([M+H]$^+$, 96%)

HRMS: (ESI$^+$)
calcd for $C_7H_{13}NO_2{}^{23}Na$: 166.0839, found: 166.0845

Preparation of (*RS*)-ethyl 2-(2-methyl-5-oxopyrrolidin-2-yl)acetate (17x)

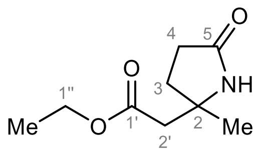

17x

Following *General Procedure 1*, ethyl (*RS*)-3-aminobutanoate **1x** (59 mg, 0.45 mmol, 1.0 equiv), methyl acrylate **14** (41 µL, 39 mg, 0.45 mmol, 1.0 equiv), 4CzIPN (2.28 mM in MeCN, 1.98 mL, 4.5 µmol, 1 mol%), tetrabutylammonium azide (70.3 mM in MeCN, 640 µL, 45 µmol, 10 mol%), and MeCN (390 µL) were reacted for 20 h with a 425 nm LED lamp. Following concentration *in vacuo*, the residue was dissolved in MeOH (5 mL) and Et_3N (0.25 mL, 0.18 g, 1.8 mmol, 4.0 equiv) was added. The reaction was then heated at reflux for 2 h, followed by concentration *in vacuo*. Purification *via* flash column chromatography on silica gel (12 g) in CH_2Cl_2 (5 CV) then 100:0:0→90:9:1 CH_2Cl_2–MeOH–aq. NH_4OH (over 15 CV) then 90:9:1 CH_2Cl_2–MeOH–aq. NH_4OH (17 CV), followed by reversed-phase flash column chromatography on C_{18} silica gel (15.5 g) in H_2O (3 CV) then 100:0→0:100 H_2O–MeOH (over 15 CV) then MeOH (16 CV), gave **17x** as a colourless oil (61.1 mg, 73%).

Data for **17x**:

^{1}H NMR: (500 MHz, $CDCl_3$)
6.39 (br s, 1H, N*H*), 4.15 (q, *J* = 7.1 Hz, 2H, C(1″)*H*$_2$), 2.53 (d, *J* = 1.5 Hz, 2H, C2′)*H*$_2$), 2.46–2.31 (m, 2H, C(4)*H*$_2$) 2.03–1.95 (m, 2H, C(3)*H*$_2$) 1.35 (s, 3H, C(2)*Me*) 1.26 (t, *J* = 7.1 Hz, 3H, C(1″)*Me*)

^{13}C NMR: (126 MHz, $CDCl_3$)
176.6 (*C*(5)), 171.0 (*C*(1′)), 60.9 (*C*(1″)), 57.1 (*C*(2)), 46.1 (*C*(2′)), 34.6 (*C*(3)), 29.7 (*C*(4)), 26.8 (C(2)*Me*) 14.3 (C(1″)*Me*)

IR: (neat)
3232 (w), 2976 (w), 2036 (w), 2010 (w), 1727 (m), 1690 (s), 1460 (w), 1369 (w), 1341 (w), 1302 (w), 1211 (m), 1189 (m), 1140 (m), 1095 (m), 1030 (m), 953 (w), 901 (w), 673 (w)

MS: (ESI$^+$)
208 ([M+Na]$^+$, 100%), 186 ([M+H]$^+$, 53%)

HRMS: (ESI$^+$)
calcd for $C_9H_{16}NO_3$: 186.1125, found: 186.1127

E.2. Challenges with α-Monosubstituted Primary Amines

E.2.1. Optimisation Studies for α-C–H Alkylation

Under the standard conditions for α-C–H alkylation, the use of α-monosubstituted primary amine **1f** results in a mixture of monoalkylated and dialkylated products: **17f** and **20f**, respectively. As generation of **20f** consumes two equivalents of acrylate, the use of a 1:1 amine:acrylate stoichiometry (as per the standard protocol), leads to incomplete conversion of amine **1f** (Entry 1). A similar result is obtained upon changing the photocatalyst to Ir[(dF(CF$_3$)ppy)$_2$(dtbbpy)]PF$_6$ (Entry 2). In order to increase the yield of α-C–H monoalkylated product **17f**, it proved necessary to increase the equivalents of amine **1f** to 2.0 equiv (Entry 3), and a further increase could be realised by switching the photocatalyst to Ir[(dF(CF$_3$)ppy)$_2$(dtbbpy)]PF$_6$ (Entry 4). The origin of the better performance of the latter catalyst over 4CzIPN is unclear, but it may be due to enhanced stability against photobleaching (deactivation), and a consequent reduction in the extent of telomerisation occurring (determined by the lifetime of the α-carboxy radical).

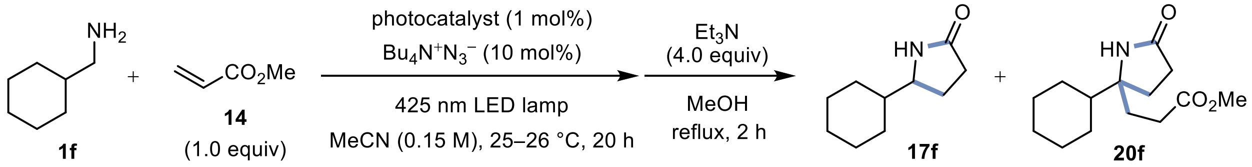

Entry	Amine **1f** Equiv.	Photocatalyst	Unreacted amine **1f**[a,b]	**17f** Yield[c]	**20f** Yield[c]	Mass Balance
1	1.0	4CzIPN	31%	40%	20%	91%
2	1.0	Ir[(dF(CF$_3$)ppy)$_2$(dtbbpy)]PF$_6$	~30%	41%	18%	~89%
3	2.0	4CzIPN	4%	45%	<2%	50%[d]
4	2.0	Ir[(dF(CF$_3$)ppy)$_2$(dtbbpy)]PF$_6$	0%	63%	7%	70%[d]

Table S7. Optimisation of reaction conditions. [a] Yield reported was measured by ^{1}H NMR in MeCN-*d*$_3$ against Bu_4N^+ as an internal standard. [b] Discounting the 1.0 equiv excess of amine **1f**. [c] Yield reported was measured by ^{1}H NMR in C_6D_6 (after concentration *in vacuo*) against Bu_4N^+ as an internal standard. [d] Complex mixture of unidentified methyl ester-containing species present.

The significantly lower mass balance observed on increasing the amine loading seems to be a consequence of the formation of a complex mixture of unidentified, methyl ester-containing by-products, as evidenced by a complex array of singlets in the -CO$_2$*Me* region of the crude ^{1}H NMR spectra. The origin of these by-products is suspected to be telomerisation arising from additions of α-carboxy radicals to further molecules of acrylate, but we were unable to confirm this. They do

however complicate chromatographic purification. We also found that increasing the amine loading still further, to 3.0 equiv, affords no measurable increase in the yield of **17f**.

E.2.2. Isolated Yields for α-C–H Monoalkylation of α-Monosubstituted Primary Amines

Prior to determining that 2.0 equiv of amine is necessary to mitigate α-C–H dialkylation (giving over-alkylated products **20**), we had conducted a brief survey of α-monosubstituted amines (**1y**–**ae**) in the α-C–H alkylation process. All of these results (with the exception of amine **1ae**) were performed with only 1.0 equiv of amine, and so the yields of monoalkylated lactams **17** are consequently low. Chromatographic purification was also challenging in these cases, which is why the more polar acrylate derivative **15** was employed. Nevertheless, we have summarised our findings in Fig. S6, followed by all the experimental procedures and characterisation data for these experiments.

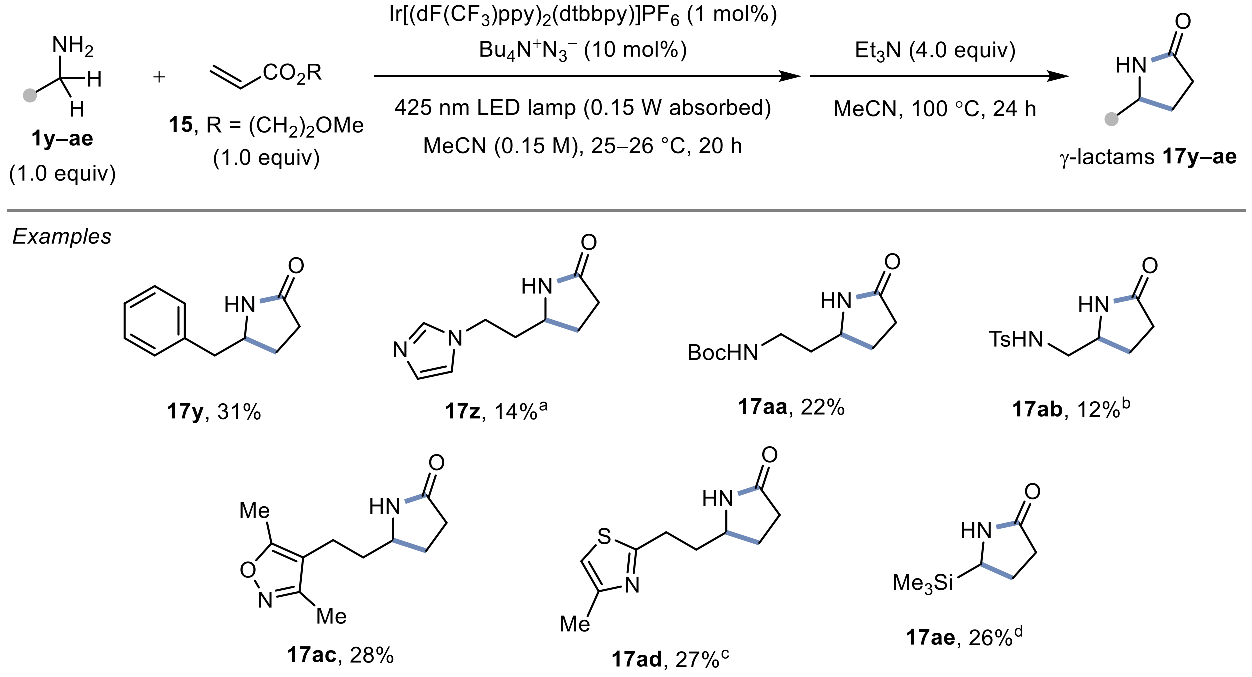

Fig. S6. Isolated yields for α-C–H monoalkylation of α-monosubstituted primary amines. [a] 16% of the α,α-dialkylated product **20z** was also isolated. [b] Gave 16% of $TsNH_2$ as a by-product. [c] 12% of the α,α-dialkylated product **20ad** was also isolated. [d] 2.0 equiv of amine **1ae** was used (16% yield with 1.0 equiv amine).

Preparation of 5-benzylpyrrolidin-2-one (17y)

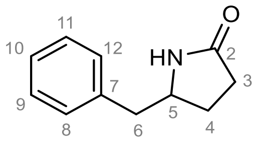
17y

Following *General Procedure 1*, 2-phenethylamine **1y** (57 µL, 55 mg, 0.45 mmol, 1.0 equiv), 2-methoxyethyl acrylate **15** (59 µL, 59 mg, 0.45 mmol, 1.0 equiv), $[Ir(dF(CF_3)ppy)_2(dtbbpy)]PF_6$ (2.23 mM in MeCN, 2.02 mL, 4.5 µmol, 1 mol%), tetrabutylammonium azide (70.3 mM in MeCN, 640 µL, 45 µmol, 10 mol%), and MeCN (340 µL) were reacted for 20 h with a 425 nm LED lamp. Following concentration *in vacuo*, the residue was dissolved in MeCN (5 mL) and Et_3N (0.25 mL, 0.18 g, 1.8 mmol, 4.0 equiv) was added. The reaction was then heated at 100 °C in a microwave vial for 24 h, followed by concentration *in vacuo*. Purification *via* flash column chromatography on silica gel (12 g) in CH_2Cl_2 (5 CV) then 100:0:0→90:9:1 CH_2Cl_2–MeOH–aq. NH_4OH (over 15 CV) then 90:9:1 CH_2Cl_2–MeOH–aq. NH_4OH (17 CV), followed by reversed-phase flash column chromatography on C_{18} silica gel (15.5 g) in H_2O (3 CV) then 100:0→0:100 H_2O–MeOH (over 15 CV) then MeOH (16 CV), gave **17y** as a colourless oil (25.0 mg, 32%).

Data for **17y**:

^{1}H NMR: (500 MHz, $CDCl_3$)
7.33 (m, 2H, C(8)*H*, C(12)*H*), 7.28–7.24 (m, 1H, C(10)*H*), 7.21–7.16 (m, 2H, C(9)*H*, C(11)*H*), 5.81–5.65 (br s, 1H, N*H*), 3.97–3.84 (m, 1H, C(5)*H*), 2.87 (dd, *J* = 13.4, 5.3 Hz, 1H, C(6)H_A), 2.72 (dd, *J* = 13.5, 8.3 Hz, 1H, C(6)H_B), 2.38–2.24 (m, 3H, C(3)H_2, C(4)H_A), 1.90–1.82 (m, 1H, C(4)H_B)

^{13}C NMR: (126 MHz, $CDCl_3$)
177.9 (*C*=O), 137.6 (*C*(7)), 129.1 (*C*(8), *C*(12)), 129.0 (*C*(9), *C*(11)), 127.0 (*C*(10)), 55.9 (*C*(5)), 43.2 (*C*(6)), 30.2 (*C*(4)), 27.2 (*C*(3))

IR: (neat)

MS: (ESI^+)
198 ($[M+Na]^+$, 18%), 177 ($[M+H]^+$, 11%), 176 (100%)

HRMS: (ESI^+)
calcd for $C_{11}H_{13}NO^{23}Na$: 198.0889, found: 198.0893

Preparation of (*RS*)-5-(2-(1*H*-imidazol-1-yl)ethyl)pyrrolidin-2-one (17z)

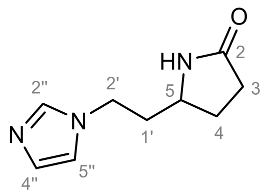

17z

Following *General Procedure 1*, 3-(1*H*-imidazol-1-yl)propan-1-amine **1z** (55 µL, 58 mg, 0.45 mmol, 1.0 equiv), 2-methoxyethyl acrylate **15** (59 µL, 59 mg, 0.45 mmol, 1.0 equiv), [Ir(dF(CF_3)ppy)$_2$(dtbbpy)]PF_6 (2.23 mM in MeCN, 2.02 mL, 4.5 µmol, 1 mol%), tetrabutylammonium azide (70.3 mM in MeCN, 640 µL, 45 µmol, 10 mol%), and MeCN (340 µL) were reacted for 20 h with a 425 nm LED lamp. Following concentration *in vacuo*, the residue was dissolved in MeCN (5 mL) and Et_3N (0.25 mL, 0.18 g, 1.8 mmol, 4.0 equiv) was added. The reaction was then heated at 100 °C in a microwave vial for 24 h, followed by concentration *in vacuo*. Purification *via* flash column chromatography on silica gel (12 g) in CH_2Cl_2 (5 CV) then 100:0:0→90:9:1 CH_2Cl_2–MeOH–aq. NH_4OH (over 15 CV) then 90:9:1 CH_2Cl_2–MeOH–aq. NH_4OH (17 CV), followed by reversed-phase flash column chromatography on C_{18} silica gel (15.5 g) in H_2O (3 CV) then 100:0→0:100 H_2O–MeOH (over 15 CV) then MeOH (16 CV), gave **17z** as an orange oil (11.4 mg, 14%).

Data for **17z**:

^{1}H NMR: (500 MHz, $CDCl_3$)
7.70 (s, 1H, C(2″)*H*), 7.12 (s, 1H, C(4″)*H*), 6.96 (s, 1H, C(5″)*H*), 6.61 (s, 1H, N*H*), 4.21–3.97 (m, 2H, C(2′)H_2), 3.65–3.53 (m, 1H, C(5)*H*), 2.44–2.22 (m, 3H, C(3)H_2, C(4)*H*), 2.02 (app q, J = 6.8 Hz, 2H, C(1′)H_2), 1.80–1.65 (m, 1H, C(4)*H*)

^{13}C NMR: (126 MHz, $CDCl_3$)
178.5 (*C*(2)), 137.2 (*C*(2″)), 128.8 (*C*(4″)), 119.0 (*C*(5″)), 51.65 (*C*(5)), 44.3 (*C*(2′)), 38.0 (*C*(1′)), 30.0 (*C*(3)), 27.2 (*C*(4))

IR: (neat)
3203 (w), 3110 (w), 2939 (w), 2384 (w), 2014 (w), 1670 (s), 1548 (w), 1511 (w), 1459 (w), 1441 (w), 1425 (w), 1392 (w), 1351 (w), 1317 (w), 1285 (m), 1266 (m), 1229 (m), 1106 (w), 1083 (m), 1031 (w), 979 (w), 917 (w), 823 (m), 748 (m), 671 (m), 664 (m), 655 (m)

MS: (ESI$^+$)

259 (14%), 181 (10%), 180 ([M+H]$^+$, 100%)

HRMS: (ESI$^+$)

calcd for $C_9H_{14}N_3O$: 180.1131, found: 180.1136

Preparation of *tert*-butyl *N*-[2-[(*RS*)-5′-oxopyrrolidin-2′-yl]ethyl]carbamate (17aa)

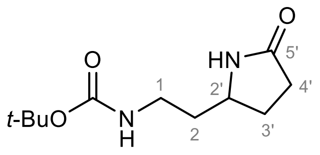

17aa

Following *General Procedure 1*, *tert*-butyl (3-aminopropyl)carbamate **1aa** (82 µL, 82 mg, 0.45 mmol, 1.0 equiv), 2-methoxyethyl acrylate **15** (59 µL, 59 mg, 0.45 mmol, 1.0 equiv), [Ir(dF(CF_3)ppy)$_2$(dtbbpy)]PF_6 (2.23 mM in MeCN, 2.02 mL, 4.5 µmol, 1 mol%), tetrabutylammonium azide (70.3 mM in MeCN, 640 µL, 45 µmol, 10 mol%), and MeCN (340 µL) were reacted for 20 h with a 425 nm LED lamp. Following concentration *in vacuo*, the residue was dissolved in MeCN (5 mL) and Et_3N (0.25 mL, 0.18 g, 1.8 mmol, 4.0 equiv) was added. The reaction was then heated at 100 °C in a microwave vial for 24 h, followed by concentration *in vacuo*. Purification *via* flash column chromatography on silica gel (12 g) in CH_2Cl_2 (5 CV) then 100:0:0→90:9:1 CH_2Cl_2–MeOH–aq. NH_4OH (over 15 CV) then 90:9:1 CH_2Cl_2–MeOH–aq. NH_4OH (17 CV), followed by reversed-phase flash column chromatography on C_{18} silica gel (15.5 g) in H_2O (3 CV) then 100:0→0:100 H_2O–MeOH (over 15 CV) then MeOH (16 CV), gave **17aa** as a white solid (22.9 mg, 22%).

Data for **17aa**:

mp: 117–118 °C

^{1}H NMR: (500 MHz, $CDCl_3$)

6.18 (s, 1H, C(O)N*H*), 4.58 (s, 1H, *t*-BuOC(O)N*H*), 3.72–3.64 (m, 1H, C(2′)*H*), 3.38–3.26 (m, 1H, C(2)H_A), 3.16–3.07 (m, 1H, C(2)H_B), 2.41–2.24 (m, 3H, C(3′)H_A, C(4)H_2), 1.78–1.59 (m, 3H, C(1)H_2, C(3′)H_B), 1.44 (s, 9H, O*t-Bu*)

^{13}C NMR: (126 MHz, $CDCl_3$)

178.3 (*C*(5′)), 156.4 (*t*-BuO*C*(O)NH), 79.7 (O*C*(CH_3)$_3$), 52.0 (*C*(2′)), 37.55 (*C*(2)), 37.50 (*C*(1)), 30.1 (*C*(4′)), 28.5 (OC(*C*H_3)$_3$), 27.3 (*C*(3′))

IR: (neat)

3323 (w), 3199 (w), 3083 (w), 2979 (w), 2968 (w), 2933 (w), 1703 (m), 1679 (s), 1536 (m), 1446 (w), 1386 (w), 1363 (m), 1310 (w), 1291 (m), 1271 (s), 1249 (m), 1211 (w), 1171 (m), 1105 (w), 1052 (m), 1022 (w), 1003 (w), 979 (w), 963 (m), 933 (w), 911 (w), 885 (w), 863 (w), 829 (w), 779 (m), 723 (w)

MS: (ESI^+)

479 (24%), 457 (34%), 251 ($[M+Na]^+$, 41%), 229 ($[M+H]^+$, 17%), 173 (100%), 129 (18%)

HRMS: (ESI^+)

calcd for $C_{11}H_{20}N_2O_3{}^{23}Na$: 251.1366, found: 251.1371

Preparation of (*RS*)-4-methyl-*N*-((5-oxopyrrolidin-2-yl)methyl)benzenesulfonamide (17ab)

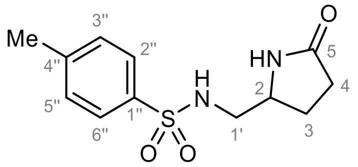

17ab

Following *General Procedure 1*, *N*-tosylethylenediamine **1ab** (99.5 mg, 0.45 mmol, 1.0 equiv), 2-methoxyethyl acrylate **15** (59 µL, 59 mg, 0.45 mmol, 1.0 equiv), $[Ir(dF(CF_3)ppy)_2(dtbbpy)]PF_6$ (2.23 mM in MeCN, 2.02 mL, 4.5 µmol, 1 mol%), tetrabutylammonium azide (70.3 mM in MeCN, 640 µL, 45 µmol, 10 mol%), and MeCN (340 µL) were reacted for 20 h with a 425 nm LED lamp. Following concentration *in vacuo*, purification *via* flash column chromatography on silica gel (12 g) in CH_2Cl_2 (5 CV) then 100:0:0→90:9:1 CH_2Cl_2–MeOH–aq. NH_4OH (over 15 CV) then 90:9:1 CH_2Cl_2–MeOH–aq. NH_4OH (17 CV), followed by reversed-phase flash column chromatography on C_{18} silica gel (15.5 g) in H_2O (3 CV) then 100:0→0:100 H_2O–MeOH (over 15 CV) then MeOH (16 CV), gave a slightly impure **17ab** (15 mg), in addition to $TsNH_2$ as a white crystalline solid (12.3 mg, 16%). The NMR spectroscopic data for $TsNH_2$ was in accordance with the literature.[17] Further purification of **6y** *via* flash column chromatography on high performance silica gel (4 g) in CH_2Cl_2 (5 CV) then 100:0:0→90:9:1 CH_2Cl_2–MeOH–aq. NH_4OH (over 15 CV) then 90:9:1 CH_2Cl_2–MeOH–aq. NH_4OH (17 CV) gave **17ab** as a white crystalline solid (13.4 mg, 11%).

Data for **17ab**:

mp: 167–169 °C

<u>^{1}H NMR:</u> (500 MHz, $CDCl_3$)

7.73 (d, *J* = 7.9 Hz, 2H, C(2″)*H*, C(6″)*H*), 7.30 (d, *J* = 8.0 Hz, 2H, C(3″)*H*, C(5″)*H*), 6.77 (s, 1H, C(O)N*H*), 6.14 (app t, *J* = 6.5 Hz, 1H, SO_2N*H*), 3.87–3.76 (m, 1H, C(2)*H*), 3.12–2.99 (m, 1H, C(1′)H_A), 2.94–2.83 (m, 1H, C(1′)H_B), 2.42 (s, 3H, C(4′)*Me*), 2.4 –2.15 (m, 3H, C(3)H_A, C(4)H_2), 1.87–1.75 (m, 1H, C(3)H_B)

<u>^{13}C NMR:</u> (126 MHz, $CDCl_3$)

178.6 (*C*(5)), 143.9 (*C*(1″)), 137.0 (*C*(4″)), 130.0 (*C*(3″), *C*(5″)), 127.1 (*C*(2″), *C*(6″)), 54.2 (*C*(2)), 48.1 (*C*(1′)), 29.9 (*C*(4)), 24.2 (*C*(3)), 21.7 (C(4′)*Me*)

<u>IR:</u> (neat)

3362 (m), 3094 (w), 2998 (w), 2919 (w), 2852 (w), 1668 (s), 1599 (w), 1493 (w), 1452 (m), 1438 (m), 1416 (w), 1358 (w), 1325 (m), 1288 (m), 1270 (m), 1211 (w), 1185 (w), 1162 (s), 1117 (m), 1091 (m), 1051 (m), 1018 (w), 996 (w), 966 (w), 919 (w), 890 (w), 810 (m), 802 (m), 770 (m), 713 (m), 702 (m), 672 (m), 657 (s)

<u>MS:</u> (ESI^+)

559 (35%), 538 (13%), 537 (46%), 393 (22%), 392 (93%), 391 (20%), 291 ($[M+Na]^+$, 29%), 270 (14%), 269 ($[M+H]^+$, 100%), 270 (14%)

<u>HRMS:</u> (ESI^+)

calcd for $C_{12}H_{17}N_2O_3S$: 269.0954, found: 269.0961

Preparation of (*RS*)-5-(2-(3,5-dimethylisoxazol-4-yl)ethyl)pyrrolidin-2-one (17ac)

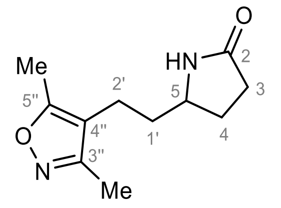

17ac

Following *General Procedure 1*, 3,5-dimethyl-4-isoxazolepropanamine **1ac** (73 mg, 0.45 mmol, 1.0 equiv), 2-methoxyethyl acrylate **15** (59 µL, 59 mg, 0.45 mmol, 1.0 equiv), $[Ir(dF(CF_3)ppy)_2(dtbbpy)]PF_6$ (2.23 mM in MeCN, 2.02 mL, 4.5 µmol, 1 mol%), tetrabutylammonium azide (70.3 mM in MeCN, 640 µL, 45 µmol, 10 mol%), and MeCN (340 µL) were reacted for 20 h with a 425 nm LED lamp. Following concentration *in vacuo*, the residue was dissolved in MeCN (5 mL) and Et_3N (0.25 mL, 0.18 g, 1.8 mmol, 4.0 equiv) was added. The reaction was then heated at 100 °C in a microwave vial for 24 h, followed by concentration *in*

vacuo. Purification *via* flash column chromatography on silica gel (12 g) in CH_2Cl_2 (5 CV) then 100:0:0→90:9:1 CH_2Cl_2–MeOH–aq. NH_4OH (over 15 CV) then 90:9:1 CH_2Cl_2–MeOH–aq. NH_4OH (17 CV), followed by reversed-phase flash column chromatography on C_{18} silica gel (15.5 g) in H_2O (3 CV) then 100:0→0:100 H_2O–MeOH (over 15 CV) then MeOH (16 CV), gave **17ac** as a white solid (26.5 mg, 28%).

Data for **17ac**:

mp: 99–101 °C

^{1}H NMR: (500 MHz, $CDCl_3$)

6.11 (s, 1H, N*H*), 3.69–3.61 (m, 1H, C(5)*H*), 2.42–2.26 (m, 8H, C(3)H_2, C(4)*H*, C(2′)H_2, C(5″)*Me*), 2.21 (s, 3H, C(3″)*Me*) 1.81–1.54 (m, 3H, C(4)*H*, C(1′)H_2)

^{13}C NMR: (126 MHz, $CDCl_3$)

178.1 (*C*(2)), 164.9 (*C*(5″)), 159.4 (*C*(3″)), 112.5 (*C*(4″)), 53.95 (*C*(5)), 36.9 (*C*(1′)), 30.1 (*C*(3)), 27.4 (*C*(4)), 19.0 (*C*(5)), 11.1 (*C*(5″)*Me*), 10.4 (*C*(3″)*Me*)

IR: (neat)

3182 (w), 3076 (w), 2990 (w), 2967 (w), 2941 (w), 2866 (w), 1693 (s), 1646 (m), 1634 (w), 1490 (w), 1442 (m), 1422 (m), 1388 (m), 1365 (w), 1341 (w), 1317 (m), 1283 (w), 1271 (m), 1216 (w), 1185 (m), 1107 (w), 1095 (w), 1035 (w), 1015 (w), 981 (w), 938 (w), 920 (w), 883 (w), 826 (w), 785 (m), 747 (m), 702 (w), 666 (w)

MS: (ESI^+)

417 (18%), 231 ($[M+Na]^+$, 32%), 210 (12%), 209 ($[M+H]^+$, 100%)

HRMS: (ESI^+)

calcd for $C_{11}H_{17}N_2O_2$: 209.1286, found: 209.1289

Preparation of (*RS*)-5-(2-(4-methylthiazol-2-yl)ethyl)pyrrolidin-2-one (17ad)

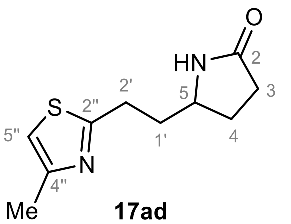

17ad

Following *General Procedure 1*, 3-(4-methylthiazol-2-yl)propan-1-amine **1ad** (70 mg, 0.45 mmol, 1.0 equiv), 2-methoxyethyl acrylate **15** (59 µL, 59 mg, 0.45 mmol, 1.0 equiv), $[Ir(dF(CF_3)ppy)_2(dtbbpy)]PF_6$ (2.23 mM in MeCN, 2.02 mL, 4.5 µmol, 1 mol%),

tetrabutylammonium azide (70.3 mM in MeCN, 640 µL, 45 µmol, 10 mol%), and MeCN (340 µL) were reacted for 20 h with a 425 nm LED lamp. Following concentration *in vacuo*, the residue was dissolved in MeCN (5 mL) and Et_3N (0.25 mL, 0.18 g, 1.8 mmol, 4.0 equiv) was added. The reaction was then heated at 100 °C in a microwave vial for 24 h, followed by concentration *in vacuo*. Purification *via* flash column chromatography on silica gel (12 g) in CH_2Cl_2 (5 CV) then 100:0:0→90:9:1 CH_2Cl_2–MeOH–aq. NH_4OH (over 15 CV) then 90:9:1 CH_2Cl_2–MeOH–aq. NH_4OH (17 CV), followed by followed by reversed-phase flash column chromatography on C_{18} silica gel (15.5 g) in H_2O (3 CV) then 100:0→0:100 H_2O–MeOH (over 15 CV) then MeOH (16 CV), gave **17ad** as a colourless oil (25.7 mg, 27%).

Data for **17ad**:

^{1}H NMR: (500 MHz, $CDCl_3$)
6.73–6.71 (m, 1H, C(5′)*H*), 6.71–6.58 (m, 1H, N*H*), 3.74–3.67 (m, 1H, C(5)*H*), 3.08–2.97 (m, 2H, C(2′)H_2), 2.41–2.39 (m, 3H, C(4)*Me*), 2.40–2.24 (m, 3H, C(3)H_2, C(4)*H*), 2.05–1.92 (m, 2H, C(1′)H_2), 1.80–1.70 (m, 1H, C(4)*H*)

^{13}C NMR: (126 MHz, $CDCl_3$)
178.2 (*C*(2)), 168.9 (*C*(4′)), 152.6 (*C*(2″)), 112.85 (*C*(5′)), 53.9 (*C*(5)), 36.7 (*C*(1′)), 30.2 (*C*(3)), 30.0 (*C*(2′)), 27.3 (*C*(4)), 17.1 (C(4)*Me*)

IR: (neat)
3183 (w), 3123 (w), 3088 (w), 2981 (w), 2969 (w), 2922 (w), 2854 (w), 1681 (s), 1526 (w), 1455 (m), 1445 (m), 1429 (w), 1388 (w), 1372 (w), 1352 (m), 1312 (w), 1285 (m), 1270 (m), 1256 (w), 1208 (w), 1165 (w), 1139 (w), 1116 (w), 1098 (w), 1080 (w), 1056 (w), 1017 (w), 993 (w), 974 (w), 966 (w), 954 (w), 929 (w), 865 (w), 793 (m), 776 (m), 757 (m), 737 (m), 693 (w)

MS: (ESI^+)
443 (15%), 233 ($[M+Na]^+$, 67%), 212 (10%), 211 ($[M+H]^+$, 100%), 142 (11%)

HRMS: (ESI^+)
calcd for $C_{10}H_{14}OS$: 210.0827, found: 210.0831

Preparation of (*RS*)-5-(trimethylsilyl)pyrrolidin-2-one (17ae)

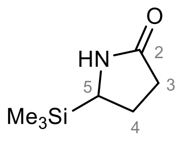

17ae

Following *General Procedure 1*, (trimethylsilyl)methanamine **1ae** (123 µL, 95 mg, 0.90 mmol, 2.0 equiv), 2-methoxyethyl acrylate **15** (59 µL, 59 mg, 0.45 mmol, 1.0 equiv), [Ir(dF(CF_3)ppy)$_2$(dtbbpy)]PF_6 (2.23 mM in MeCN, 2.02 mL, 4.5 µmol, 1 mol%), tetrabutylammonium azide (70.3 mM in MeCN, 640 µL, 45 µmol, 10 mol%), and MeCN (340 µL) were reacted for 20 h with a 425 nm LED lamp. Following concentration *in vacuo*, the residue was dissolved in MeCN (5 mL) and Et_3N (0.25 mL, 0.18 g, 1.8 mmol, 4.0 equiv) was added. The reaction was then heated at 100 °C in a microwave vial for 24 h, followed by concentration *in vacuo*. Purification *via* flash column chromatography on silica gel (12 g) in CH_2Cl_2 (5 CV) then 100:0:0→90:9:1 CH_2Cl_2–MeOH–aq. NH_4OH (over 15 CV) then 90:9:1 CH_2Cl_2–MeOH–aq. NH_4OH (17 CV), followed by reversed-phase flash column chromatography on C_{18} silica gel (15.5 g) in H_2O (3 CV) then 100:0→0:100 H_2O–MeOH (over 15 CV) then MeOH (16 CV), followed by flash column chromatography on high performance silica gel (4 g) in CH_2Cl_2 (5 CV) then 100:0:0→90:9:1 CH_2Cl_2–MeOH–aq. NH_4OH (over 15 CV) then 90:9:1 CH_2Cl_2–MeOH–aq. NH_4OH (17 CV), gave **17ae** as a white crystalline solid (18.6 mg, 26%).

Data for **17ae**:

mp: 113–114 °C

^{1}H NMR: (500 MHz, $CDCl_3$)

5.70 (br s, 1H, N*H*), 3.07 (dd, 1H, *J* = 9.3, 7.7 Hz, C(5)*H*), 2.42–2.33 (m, 1H, C(3)H_A), 2.32–2.24 (m, 1H, C(3)H_B), 2.24–2.16 (m, 1H, C(4)H_A), 2.01–1.90 (m, 1H, C(4)H_B), 0.06 (s, 9H, SiMe_3)

^{13}C NMR: (126 MHz, $CDCl_3$)

179.7 (*C*(2)), 44.6 (*C*(5)), 31.4 (*C*(3)), 23.4 (*C*(4)), –4.15 (SiMe_3)

IR: (neat)

2962 (m), 2931 (m), 2872 (m), 1650 (s), 1587 (w), 1462 (w), 1423 (w), 1384 (w), 1361 (m), 1283 (m), 1257 (s), 1173 (w), 1157 (w), 1103 (w), 1069 (w), 1057 (w), 996 (m), 921 (w), 876 (w), 736 (w), 664 (m), 612 (w), 492 (w), 408 (w)

<u>MS:</u> (ESI^+)

181 (11%), 180 ($[M+Na]^+$, 100%), 158 ($[M+H]^+$, 34%)

<u>HRMS:</u> (ESI^+)

calcd for $C_7H_{15}NOSi$: 157.0923, found: 157.0925

E.3. Robustness Screen with Functional Group Additives

additive	product yield (**8** + **17b**)	additive remaining	amine **5** remaining	acrylate **14** remaining
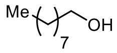	✓ 69%	✓ 82%	34%	0%
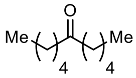	✓ 70%	✓ 71%	17%	0%
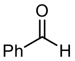	× 13%	× 0%	0%	45%
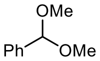	✓ 80%	✓ 81%	0%	0%
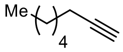	✓ 77%	✓ >80%	5%	0%
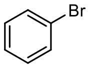	✓ 69%	✓ 76%	0%	1%
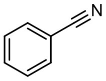	✓ 81%	✓ 76%	0%	0%
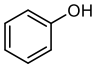	× <10%	- 68%	-	1%

additive	product yield (**8** + **17b**)	additive remaining	amine **5** remaining	acrylate **14** remaining
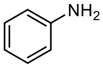	× 23%	- 61%	86%	88%
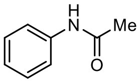	✓ 70%	✓ 80%	9%	0%
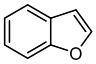	✓ 65%	✓ 71%	9%	0%
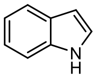	× <5%	✓ 81%	99%	5%
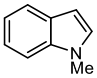	- 44%	✓ 70%	44%	19%
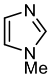	✓ 77%	✓ 80%	0%	1%
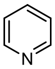	✓ 73%	✓ >80%	4%	0%

Fig. S7. Table shows the yield of total product (unlactamised **8** + lactamised **17b**) when the standard reaction (cyclohexylamine **5** and methyl acrylate **14**) is carried out in the presence of one molar equivalent of the given additive. All yields were measured by ^{1}H NMR in MeCN-d_3 against Bu_4N^+ as an internal standard.

E.4. Scope of α-C–H Dialkylation of α-Monosubstituted Primary Amines

Preparation of (*RS*)-methyl 3-(2-cyclohexyl-5-oxopyrrolidin-2-yl)propanoate (20f)

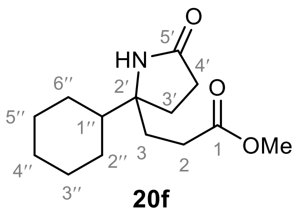

20f

Following *General Procedure 2*, cyclohexanemethylamine **1f** (60 µL, 52 mg, 0.45 mmol, 1.0 equiv), methyl acrylate **14** (124 µL, 118 mg, 1.35 mmol, 3.0 equiv), 4CzIPN (2.28 mM in MeCN, 1.98 mL, 4.5 µmol, 1 mol%), tetrabutylammonium azide (70.3 mM in MeCN, 640 µL, 45 µmol, 10 mol%), and MeCN (390 µL) were reacted for 20 h with a 425 nm LED lamp. Following concentration *in vacuo*, the residue was dissolved in MeOH (5 mL) and Et_3N (0.25 mL, 0.18 g, 1.8 mmol, 4.0 equiv) was added. The reaction was then heated at reflux for 2 h, followed by concentration *in vacuo*. Purification *via* flash column chromatography on silica gel (12 g) in CH_2Cl_2 (5 CV) then 100:0:0→90:9:1 CH_2Cl_2–MeOH–aq. NH_4OH (over 15 CV) then 90:9:1 CH_2Cl_2–MeOH–aq. NH_4OH (17 CV), followed by reversed-phase flash column chromatography on C_{18} silica gel (50 g, 20 mm Ø) in 80:20 MeOH–H_2O, gave **20f** as a yellow oil (41 mg, 36%).

Data for **20f**:

^{1}H NMR: (500 MHz, $CDCl_3$)
7.09–6.89 (m, 1H, N*H*), 3.64 (s, 3H, O*Me*), 2.37–2.22 (m, 4H, C(4′)H_2, C(2)H_2), 2.04–1.58 (m, 9H, C(3′)H_2, C(3)H_2, C(2″)H_A, C(3″)H_A, C(4″)H_A, C(5″)H_A, C(6″)H_A), 1.39–1.31 (m, 1H, C(1″)*H*), 1.26–0.88 (m, 5H, C(2″)H_B, C(3″)H_B, C(4″)H_B, C(5″)H_B, C(6″)H_B)

^{13}C NMR: (126 MHz, $CDCl_3$)
177.8 (*C*(5′)), 174.0 (*C*(1)), 64.0 (*C*(2′)), 51.9 (O*Me*), 46.6 (*C*(1″)), 32.7 (*C*(3)), 30.8 (*C*(4′)), 28.8 (*C*(2)), 28.1 (*C*(3′)), 27.1 (*C*(2″)), 27.0 (*C*(6″)), 26.5 (*C*(3″), *C*(5″)), 26.4 (*C*(4″))

IR: (neat)
3196 (w), 2926 (w), 2853 (w), 2359 (w), 2175 (w), 2058 (w), 1735 (m), 1684 (s), 1436 (w), 1367 (w), 1255 (w), 1196 (m), 1170 (m), 1108 (w), 1016 (w), 993 (w), 892 (w), 780 (w)

MS: (ESI$^+$)

276 ([M+Na]$^+$, 100%), 254 ([M+H]$^+$, 31%)

HRMS: (ESI$^+$)

calcd for $C_{14}H_{23}NO_3$: 253.1678, found: 253.1682

Preparation of (*RS*)-2-methoxyethyl 3-(2-benzyl-5-oxopyrrolidin-2-yl)propanoate (20y)

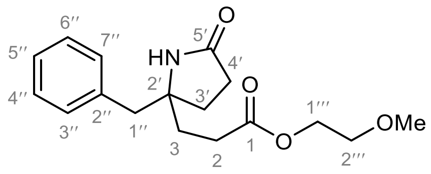

20y

Following *General Procedure 2*, 2-phenethylamine **1y** (57.5 µL, 55.3 mg, 0.45 mmol, 1.0 equiv), 2-methoxyethyl acrylate **15** (178 µL, 179.4 mg, 1.35 mmol, 3.0 equiv), Ir[dF(CF_3)ppy]$_2$(dtbbpy))PF_6 (2.23 mM in MeCN, 2.02 mL, 4.5 µmol, 1 mol%), tetrabutylammonium azide (70.3 mM in MeCN, 640 µL, 45 µmol, 10 mol%), and MeCN (340 µL) were reacted for 20 h with a 425 nm LED lamp. Following concentration *in vacuo*, the residue was dissolved in MeCN (5 mL) and Et_3N (0.25 mL, 0.18 g, 1.8 mmol, 4.0 equiv) was added. The reaction was then heated at reflux for 24 h, followed by concentration *in vacuo*. Purification *via* flash column chromatography on silica gel (12 g) in CH_2Cl_2 (5 CV) then 100:0:0→90:9:1 CH_2Cl_2–MeOH–aq. NH_4OH (over 15 CV) then 90:9:1 CH_2Cl_2–MeOH–aq. NH_4OH (17 CV), followed by reversed-phase flash column chromatography on C_{18} silica gel (50 g, 20 mm Ø) in 80:20 MeOH–H_2O, gave **20y** as a colourless oil (75 mg, 55%).

Data for **20y**:

^{1}H NMR: (500 MHz, $CDCl_3$)

7.34–7.14 (m, 5H, C(3″)*H*, C(4″)*H*, C(5″)*H*, C(6″)*H*, C(7″)*H*)), 6.40–6.18 (m, 1H, N*H*), 4.31–4.15 (m, 2H, C(1‴)H_2), 3.61–3.54 (m, 2H, C(2‴)H_2), 3.38 (s, 3H, O*Me*), 2.85–2.71 (m, 2H, C(1″)H_2), 2.48–2.41 (m, 2H, C(3)H_2), 2.24–2.14 (m, 1H, C(4′)H_A), 2.05–1.81 (m, 5H, C(4′)H_B, C(3′)H_2, C(2)H_2)

^{13}C NMR: (126 MHz, $CDCl_3$)

177.4 (*C*(5′)), 173.2 (*C*(1)), 136.1 (*C*(2″)), 130.5 (*C*(3″), *C*(7″)), 128.6 (*C*(4″), *C*(6″)), 127.1 (*C*(5″)), 70.4 (*C*(2‴)), 63.8 (*C*(1‴)), 61.6 (*C*(2′)), 59.0 (O*Me*), 46.8 (*C*(1″)), 34.6 (*C*(3′)), 30.4 (*C*(2)), 30.1 (*C*(4′)), 29.1 (*C*(3))

IR: (neat)

3285 (w), 2972 (w), 2172 (w), 2146 (w), 1681 (s), 1519 (w), 1438 (w), 1391 (w), 1365 (m), 1270 (m), 1249 (m), 1166 (s), 1044 (w), 1015 (w), 868 (w), 780 (w)

MS: (ESI^+)

653 (100%), 441 (18%), 328 ($[M+Na]^+$, 27%), 242 (29%), 221 (21%)

HRMS: (ESI^+)

calcd for $C_{17}H_{23}NO_4$: 305.1631, found: 305.1631

Preparation of (*RS*)-methyl 3-(2-(2-(1*H*-imidazol-1-yl)ethyl)-5-oxopyrrolidin-2-yl)propanoate (20z)

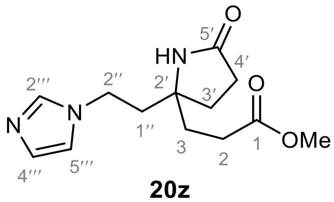

20z

Following *General Procedure 2*, 1-(3-aminopropyl)imidazole **1z** (55 µL, 57.5 mg, 0.45 mmol, 1.0 equiv), methyl acrylate **14** (124 µL, 118 mg, 1.35 mmol, 3.0 equiv), $Ir[dF(CF_3)ppy]_2(dtbbpy))PF_6$ (2.23 mM in MeCN, 2.02 mL, 4.5 µmol, 1 mol%), tetrabutylammonium azide (70.3 mM in MeCN, 640 µL, 45 µmol, 10 mol%), and MeCN (340 µL) were reacted for 20 h with a 425 nm LED lamp. Following concentration *in vacuo*, the residue was dissolved in MeCN (5 mL) and Et_3N (0.25 mL, 0.18 g, 1.8 mmol, 4.0 equiv) was added. The reaction was then heated at reflux for 24 h, followed by concentration *in vacuo*. Purification *via* flash column chromatography on silica gel (12 g) in CH_2Cl_2 (5 CV) then 100:0:0→90:9:1 CH_2Cl_2–MeOH–aq. NH_4OH (over 15 CV) then 90:9:1 CH_2Cl_2–MeOH–aq. NH_4OH (17 CV), followed by reversed-phase flash column chromatography on C_{18} silica gel (50 g, 20 mm Ø) in 80:20 MeOH–H_2O, gave **20z** as a white crystalline solid (56 mg, 47%, >95% purity).

Data for **20z**:

mp: 132–133 °C

^{1}H NMR: (500 MHz, $CDCl_3$)

7.84–7.69 (m, 1H, N*H*), 7.51–7.46 (m, 1H, C(5′′′)*H*), 7.04–6.98 (m, 1H, C(4′′′)*H*), 6.93–6.86 (m, 1H, C(2′′′)*H*), 4.05–3.98 (m, 2H, C(2′′)H_2), 3.68–3.64 (s, 3H, O*Me*), 2.43–2.31 (m, 4H, C(3′)H_2, C(3)H_2), 2.07–1.83 (m, 6H, C(1′′)H_2, C(4′)H_2, C(2)H_2)

^{13}C NMR: (126 MHz, $CDCl_3$)

177.7 (*C*(5′)), 173.4 (*C*(1)), 137.0 (*C*(5‴)), 129.7 (*C*(4‴)), 118.8 (*C*(2‴)), 60.2 (*C*(2′)), 52.1 (O*Me*), 42.5 (*C*(2″)), 41.3 (*C*(1″)), 34.6, 30.7 (*C*(2), *C*(4′)), 30.4 (*C*(3′)), 28.8 (*C*(3))

IR: (neat)

3165 (w), 3124 (w), 3104 (w), 3072 (w), 2948 (w), 2886 (w), 2172 (w), 2040 (w), 2025 (w), 1977 (w), 1732 (m), 1686 (s), 1507 (m), 1464 (w), 1449 (m), 1436 (m), 1418 (w), 1389 (m), 1337 (w), 1316 (m), 1284 (m), 1232 (m), 1220 (w), 1195 (m), 1181 (m), 1170 (m), 1123 (m), 1113 (m), 1102 (m), 1085 (m), 1032 (w), 991 (m), 941 (w), 906 (m), 895 (w), 798 (s), 766 (m), 733 (m), 709 (m), 657 (m)

MS: (ESI$^+$)

553 (16%), 328 (14%), 288 (30%), 267 (13%), 266 ([M+H]$^+$,100%)

HRMS: (ESI$^+$)

calcd for $C_{13}H_{19}N_3O_3$: 265.1426, found: 265.1432

Preparation of (*RS*)-methyl 3-(2-(2-((*tert*-butoxycarbonyl)amino)ethyl)-5-oxopyrrolidin-2-yl)propanoate (20aa)

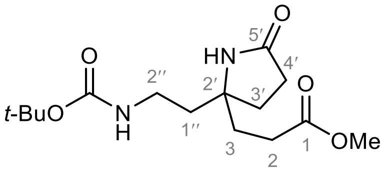

20aa

Following *General Procedure 2*, *N*-Boc-1,3-propanediamine **1aa** (82 µL, 82 mg, 0.45 mmol, 1.0 equiv), methyl acrylate **14** (124 µL, 118 mg, 1.35 mmol, 3.0 equiv), Ir[dF(CF_3)ppy]$_2$(dtbbpy))PF_6 (2.23 mM in MeCN, 2.02 mL, 4.5 µmol, 1 mol%), tetrabutylammonium azide (70.3 mM in MeCN, 640 µL, 45 µmol, 10 mol%), and MeCN (340 µL) were reacted for 20 h with a 425 nm LED lamp. Following concentration *in vacuo*, the residue was dissolved in MeCN (5 mL) and Et_3N (0.25 mL, 0.18 g, 1.8 mmol, 4.0 equiv) was added. The reaction was then heated at reflux for 24 h, followed by concentration *in vacuo*. Purification *via* flash column chromatography on silica gel (12 g) in CH_2Cl_2 (5 CV) then 100:0:0→90:9:1 CH_2Cl_2–MeOH–aq. NH_4OH (over 15 CV) then 90:9:1 CH_2Cl_2–MeOH–aq. NH_4OH (17 CV), followed by reversed-phase flash column chromatography on C_{18} silica gel (50 g, 20 mm Ø) in 80:20 MeOH–H_2O, gave **20aa** as a colourless oil (69 mg, 49%).

<u>Data for **20aa**:</u>

<u>^{1}H NMR:</u> (500 MHz, $CDCl_3$)

7.18–6.88 (m, 1H, N*H*), 4.82 (s, 1H, N*H*), 3.65 (s, 3H, O*Me*), 3.24–3.04 (m, 2H, C(2″)*H*$_2$), 2.41–2.27 (m, 4H, C(3)*H*$_2$, C(3′)*H*$_2$), 2.03–1.81 (m, 4H, C(2)*H*$_2$, C(4′)*H*$_2$), 1.77–1.69 (m, 2H, C(1″)*H*$_2$), 1.41 (s, 9H, O*t*-*Bu*)

<u>^{13}C NMR:</u> (126 MHz, $CDCl_3$)

177.6 (*C*(5′)), 173.7 (*C*(1)), 156.1 (*C*O$_2$*t*-Bu), 79.6 (O*C*Me$_3$), 60.4 (*C*(2′)), 52.0 (O*Me*)), 40.0 (*C*(1″)), 36.3 (*C*(2″)), 34.8 (*C*(4′)), 30.9 (*C*(2)), 30.3 (*C*(3′)), 28.9 (*C*(3)), 28.5 (OC*Me*$_3$)

<u>IR:</u> (neat)

3285 (w), 2972 (w), 2172 (w), 2146 (w), 1681 (s), 1519 (w), 1438 (w), 1391 (w), 1365 (m), 1270 (m), 1249 (m), 1166 (s), 1044 (w), 1015 (w), 868 (w), 780 (w)

<u>MS:</u> (ESI^+)

652 (16%), 651 (48%), 338 (17%), 337 ($[M+Na]^+$,100%), 259 (25%), 242 (20%)

<u>HRMS:</u> (ESI^+)

calcd for $C_{15}H_{26}N_2O_5$: 314.1842, found: 314.1847

Preparation of (*RS*)-methyl 3-(2-(((4-methylphenyl)sulfonamido)methyl)-5-oxopyrrolidin-2-yl)propanoate (20ab)

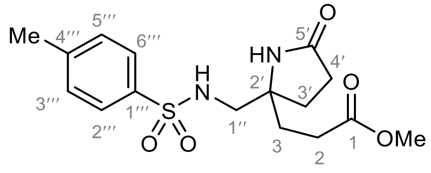

20ab

Following *General Procedure 2*, *N*-tosylethylenediamine **1ab** (99.5 mg, 0.45 mmol, 1.0 equiv), methyl acrylate **14** (124 µL, 118 mg, 1.35 mmol, 3.0 equiv), Ir[dF(CF_3)ppy]$_2$(dtbbpy))PF_6 (2.23 mM in MeCN, 2.02 mL, 4.5 µmol, 1 mol%), tetrabutylammonium azide (70.3 mM in MeCN, 640 µL, 45 µmol, 10 mol%), and MeCN (340 µL) were reacted for 20 h with a 425 nm LED lamp. Following concentration *in vacuo*, the residue was dissolved in MeCN (5 mL) and Et_3N (0.25 mL, 0.18 g, 1.8 mmol, 4.0 equiv) was added. The reaction was then heated at reflux for 24 h, followed by concentration *in vacuo*. Purification *via* flash column chromatography on silica gel (12 g) in CH_2Cl_2 (5 CV) then 100:0:0→90:9:1 CH_2Cl_2–MeOH–aq. NH_4OH (over 15 CV) then 90:9:1 CH_2Cl_2–MeOH–aq. NH_4OH (17 CV), followed by reversed-phase flash column chromatography

on C_{18} silica gel (50 g, 20 mm Ø) in 80:20 MeOH–H_2O, gave **20ab** as a colourless oil (41 mg, 27%).

Data for **20ab**:

^{1}H NMR: (500 MHz, $CDCl_3$)
7.72 (d, J = 8.1 Hz, 2H, C(2‴)*H*, C(6‴)*H*), 7.29 (d, J = 8.1 Hz, 2H, C(3‴)*H*, C(5‴)*H*), 6.83 (s, 1H, N*H*), 3.65 (s, 3H, O*Me*), 2.94–2.83 (m, 2H, C(1″)H_2), 2.52–2.29 (m, 8H, C(2)H_2, C(4′)H_2, C(4‴)*Me*), 2.15–2.04 (m, 1H, C(3′)H_A), 2.00–1.80 (m, 3H, C(3′)H_B, C(3)H_2)

^{13}C NMR: (126 MHz, $CDCl_3$)
178.5 (*C*(5′)), 173.7 (*C*(1)), 143.6 (*C*(4‴)), 137.2 (*C*(1‴)), 129.9 (*C*(3‴), *C*(5‴)), 127.0 (*C*(2‴), *C*(6‴)), 61.9 (*C*(2′)), 52.1 (O*Me*)), 50.9 (*C*(1″)), 32.6 (*C*(3)), 30.7 (*C*(4′)), 28.6 (*C*(2)), 28.5 (*C*(3′)), 21.7 (C(4‴)*Me*)

IR: (neat)
3265 (w), 2950 (w), 2370 (w), 2358 (w), 2326 (w), 2035 (w), 1977 (w), 1735 (w), 1686 (w), 1599 (w), 1437 (w), 1328 (w), 1200 (w), 1159 (m), 1092 (w), 1020 (w), 914 (w), 815 (w), 729 (w), 672 (w), 662 (w), 655 (w)

MS: (ESI$^+$)
387.1 ($[M+CH_3OH+H]^+$, 27%), 377 ($[M+Na]^+$, 25%), 355.1 ($[M+H]^+$, 13%), 264.1 (64%), 248.1 (11%), 247.1 (75%), 243.2 (20%), 242.3 (100%)

HRMS: (ESI$^+$)
calcd for $C_{16}H_{23}N_2O_5S$: 355.1322, found: 355.1327

E.5. Scope of Michael Acceptors

Preparation of (*RS*)-3-methyl-1-azaspiro[4.5]decan-2-one (23a)

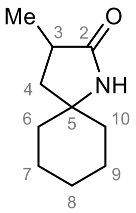

23a

Following *General Procedure 1*, cyclohexylamine **5** (52 µL, 45 mg, 0.45 mmol, 1.0 equiv), methyl methacrylate **21a** (45.1 mg, 0.45 mmol, 1.0 equiv), 4CzIPN (2.28 mM in MeCN, 1.98 mL, 4.5 µmol, 1 mol%), tetrabutylammonium azide (70.3 mM in MeCN, 640 µL, 45 µmol, 10 mol%), and MeCN (390 µL) were reacted for 20 h with a 425 nm LED lamp. Following concentration *in vacuo*, the residue was dissolved in MeOH (5 mL) and Et_3N (0.25 mL, 0.18 g, 1.8 mmol, 4.0 equiv) was added. The reaction was then heated at reflux for 2 h, followed by concentration *in vacuo*. Purification *via* flash column chromatography on silica gel (12 g) in CH_2Cl_2 (3 CV) then 100:0:0→90:9:1 CH_2Cl_2–MeOH–aq. NH_4OH (over 15 CV) then 90:9:1 CH_2Cl_2–MeOH–aq. NH_4OH (17 CV), followed by reversed-phase flash column chromatography on C_{18} silica gel (15.5 g) in H_2O (3 CV) then 100:0→0:100 H_2O–MeOH (over 15 CV) then MeOH (16 CV), gave **23a** as a white solid (58.7 mg, 78%). The NMR spectroscopic data was in accordance with the literature.[6]

Data for **23a**:

mp: 111–113 °C {lit.[6] 95–97 °C}

^{1}H NMR: (500 MHz, $CDCl_3$)
6.28 (br s, 1H, N*H*), 2.61–2.51 (m, 1H, C(3)*H*), 2.24 (dd, *J* = 12.7, 8.9 Hz, 1H, C(4)H_A), 1.59–1.47 (m, 8H, C(6)H_2, C(7)H_2, C(9)H_2, C(10)H_2), 1.46–1.35 (m, (m, 3H, C(4)H_B, C(8)H_2), 1.19 (d, *J* = 7.2 Hz, 3H, C(3)*Me*)

^{13}C NMR: (126 MHz, $CDCl_3$)
179.3 (*C*(2)), 57.0 (*C*(5)), 41.9 (*C*(4)), 39.8 (*C*(6) or *C*(10)), 37.8 (*C*(10) or *C*(6)), 35.5 (*C*(3)), 25.3 (*C*(7) or *C*(9)), 23.3 (*C*(9) or *C*(7)), 23.2 (*C*(8)), 16.7 (C(3)*Me*)

IR: (neat)
3169 (w), 3080 (w), 2964 (w), 2925 (m), 2854 (m), 1981 (w), 1690 (s), 1453 (m), 1379 (w), 1357 (w), 1342 (w), 1328 (w), 1316 (w), 1286 (w), 1256 (w), 1236 (w),

1180 (w), 1148 (w), 1100 (w), 1066 (w), 988 (w), 963 (w), 933 (w), 912 (w), 889 (w), 785 (m), 708 (w)

MS: (ESI$^+$)

357 (27%), 335 (13%), 190 ([M+Na]$^+$, 100%), 168 ([M+H]$^+$, 84%)

HRMS: (ESI$^+$)

calcd for $C_{10}H_{17}ON^{23}Na$: 190.1202, found: 190.1210

Preparation of (*RS*)-3-phenyl-1-azaspiro[4.5]decan-2-one (23b)

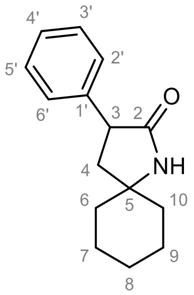

23b

Following *General Procedure 1*, cyclohexylamine **5** (52 µL, 45 mg, 0.45 mmol, 1.0 equiv), methyl 2-phenylacrylate **21b** (73 mg, 0.45 mmol, 1.0 equiv), 4CzIPN (2.28 mM in MeCN, 1.98 mL, 4.5 µmol, 1 mol%), tetrabutylammonium azide (70.3 mM in MeCN, 640 µL, 45 µmol, 10 mol%), and MeCN (390 µL) were reacted for 20 h with a 425 nm LED lamp. Following concentration *in vacuo*, the residue was dissolved in MeOH (5 mL) and Et_3N (0.25 mL, 0.18 g, 1.8 mmol, 4.0 equiv) was added. The reaction was then heated at reflux for 2 h, followed by concentration *in vacuo*. Purification *via* flash column chromatography on silica gel (12 g) in CH_2Cl_2 (3 CV) then 100:0:0→90:9:1 CH_2Cl_2–MeOH–aq. NH_4OH (over 15 CV) then 90:9:1 CH_2Cl_2–MeOH–aq. NH_4OH (17 CV), followed by reversed-phase flash column chromatography on C_{18} silica gel (15.5 g) in H_2O (3 CV) then 100:0→0:100 H_2O–MeOH (over 15 CV) then MeOH (16 CV), gave **23b** as a white crystalline solid (72.4 mg, 70%).

Data for **23b**:

mp: 185–187 °C

^{1}H NMR: (500 MHz, $CDCl_3$)

7.37–7.31 (m, 2H, C(3′)*H*, C(5′)*H*), 7.30–7.23 (m, 3H, C(2′)*H*, C(4′)*H*, C(6′)*H*), 6.08 (s, 1H, N*H*), 3.80 (app t, *J* = 9.8 Hz, 1H, C(3)*H*), 2.54 (dd, *J* = 13.0, 9.4 Hz, 1H, C(4)H_A), 1.94 (dd, *J* = 13.0, 10.4 Hz, 1H, C(4)H_B), 1.78–1.37 (m, 10H, C(6)H_2,

C(7)H_2, C(8)H_2, C(9)H_2, C(10)H_2)

<u>^{13}C NMR:</u> (126 MHz, $CDCl_3$)

176.45 (*C*(2)), 139.4 (*C*(1′)), 128.7 (*C*(3′), *C*(5′)), 128.15 (*C*(2′), *C*(6′)), 127.0 (*C*(4′)), 56.8 (*C*(5)), 47.05 (*C*(3)), 42.85 (*C*(4)), 39.55, 37.6 (*C*(6), *C*(10)), 25.1 (*C*(8)), 23.15, 23.12 (*C*(7), *C*(9))

<u>IR:</u> (neat)

3745 (w), 3176 (w), 3077 (w), 3030 (w), 2972 (w), 2924 (w), 2853 (w), 2117 (w), 1738 (w), 1694 (s), 1603 (w), 1497 (w), 1451 (m), 1369 (m), 1338 (w), 1306 (w), 1275 (w), 1252 (w), 1230 (w), 1217 (w), 1176 (w), 1160 (w), 1129 (w), 1101 (w), 1067 (w), 1030 (w), 989 (w), 967 (w), 920 (w), 891 (w), 855 (w), 810 (m), 788 (w), 755 (m), 710 (m), 694 (m), 657 (w)

<u>MS:</u> (ESI^+)

481 (33%), 259 (16%), 252 ($[M+Na]^+$, 94%), 230 ($[M+H]^+$, 100%)

<u>HRMS:</u> (ESI^+)

calcd for $C_{15}H_{20}NO$: 230.1539, found: 230.1546

Preparation of (*RS*)-3-[bis[(1,1-dimethylethoxy)carbonyl]amino]-2-oxo-1-azaspiro[4.5]decane (23c)

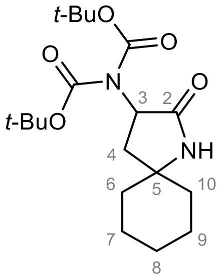

23c

Following *General Procedure 1*, cyclohexylamine **5** (52 µL, 45 mg, 0.45 mmol, 1.0 equiv), methyl 2-[di(*tert*-butoxycarbonyl)amino]prop-2-enoate **21c** (136 mg, 0.45 mmol, 1.0 equiv), 4CzIPN (2.28 mM in MeCN, 1.98 mL, 4.5 µmol, 1 mol%), tetrabutylammonium azide (70.3 mM in MeCN, 640 µL, 45 µmol, 10 mol%), and MeCN (390 µL) were reacted for 20 h with a 425 nm LED lamp. Following concentration *in vacuo*, the residue was dissolved in MeOH (5 mL) and Et_3N (0.25 mL, 0.18 g, 1.8 mmol, 4.0 equiv) was added. The reaction was then heated at reflux for 2 h, followed by concentration *in vacuo*. Purification *via* flash column chromatography on silica gel (12 g) in CH_2Cl_2 (3 CV) then 100:0:0→90:9:1 CH_2Cl_2–MeOH–aq. NH_4OH (over 15 CV) then 90:9:1

CH_2Cl_2–MeOH–aq. NH_4OH (17 CV), followed by reversed-phase flash column chromatography on C_{18} silica gel (15.5 g) in H_2O (3 CV) then 100:0→0:100 H_2O–MeOH (over 15 CV) then MeOH (16 CV), gave **23c** as a white crystalline solid (34.2 mg, 20%).

Data for **23c**:

mp: 162–164 °C

^{1}H NMR: (500 MHz, $CDCl_3$)
6.43 (s, 1H, N*H*), 4.99 (dd, *J* = 10.4, 9.1 Hz, 1H, C(3)*H*), 2.35 (dd, *J* = 12.0, 9.1 Hz, 1H, C(4)H_A), 2.03 (dd, *J* = 11.8, 10.9 Hz, 1H, C(4)H_B), 1.72–1.30 (m, 28H, C(6)H_2, C(7)H_2, C(8)H_2, C(9)H_2, C(10)H_2, 2 × O*t*-*Bu*)

^{13}C NMR: (126 MHz, $CDCl_3$)
172.75 (2 × *C*O_2*t*-Bu), 152.2 (*C*(2)), 83.3 (2 × O*C*(CH_3)$_3$), 56.1 (*C*(3)), 55.9 (*C*(5)), 39.2 (*C*(6) or *C*(10)), 38.2 (*C*(10) or *C*(6)), 37.7 (*C*(4)), 28.2 (OC(*C*H_3)$_3$), 25.15 (*C*(8)), 23.2 (*C*(7) or *C*(9)), 23.0 (*C*(9) or *C*(7))

IR: (neat)
3183 (w), 3093 (w), 2983 (w), 2926 (w), 2851 (w), 2165 (w), 2025 (w), 1976 (w), 1733 (w), 1697 (m), 1479 (w), 1454 (w), 1384 (w), 1364 (m), 1355 (m), 1342 (m), 1305 (w), 1260 (w), 1244 (m), 1229 (w), 1172 (m), 1148 (m), 1139 (m), 1123 (m), 1033 (w), 1001 (w), 985 (w), 963 (w), 945 (w), 930 (w), 894 (w), 870 (w), 854 (w), 835 (w), 814 (w), 761 (w), 701 (w), 681 (w), 673 (w), 659 (w)

MS: (ESI^+)
428 (12%), 392 (20%), 391 ($[M+Na]^+$, 100%), 269 (10%), 213 (36%)

HRMS: (ESI^+)
calcd for $C_{19}H_{32}N_2O_5{}^{23}Na$: 391.2203, found: 391.2210

Preparation of (*RS*)-4-methyl-1-azaspiro[4.5]decan-2-one (23d)

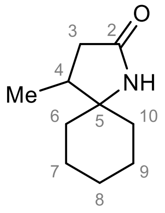

23d

Following *General Procedure 1*, cyclohexylamine **5** (52 µL, 45 mg, 0.45 mmol, 1.0 equiv), methyl crotonate **21d** (45 mg, 0.45 mmol, 1.0 equiv), 4CzIPN (2.28 mM in MeCN, 1.98 mL, 4.5 µmol, 1

mol%), tetrabutylammonium azide (70.3 mM in MeCN, 640 µL, 45 µmol, 10 mol%), and MeCN (390 µL) were reacted for 20 h with a 425 nm LED lamp. Following concentration *in vacuo*, the residue was dissolved in MeOH (5 mL) and Et_3N (0.25 mL, 0.18 g, 1.8 mmol, 4.0 equiv) was added. The reaction was then heated at reflux for 2 h, followed by concentration *in vacuo*. Purification *via* flash column chromatography on silica gel (12 g) in CH_2Cl_2 (3 CV) then 100:0:0→90:9:1 CH_2Cl_2–MeOH–aq. NH_4OH (over 15 CV) then 90:9:1 CH_2Cl_2–MeOH–aq. NH_4OH (17 CV), followed by reversed-phase flash column chromatography on C_{18} silica gel (15.5 g) in H_2O (3 CV) then 100:0→0:100 H_2O–MeOH (over 15 CV) then MeOH (16 CV), gave **23d** as a white crystalline solid (47.4 mg, 63%). The NMR spectroscopic data was in accordance with the literature.[6]

Data for **23d**:

mp: 196–198°C {lit.[18] 199–200 °C}

^{1}H NMR: (500 MHz, $CDCl_3$)
6.57 (br s, 1H, N*H*), 2.52–2.42 (dd, *J* = 16.3, 7.8 Hz, 2H, C(3)H_A), 2.17–2.08 (m, 1H, C(4)*H*), 2.07–2.01 (m, 1H, C(3)H_B), 1.73–1.18 (m, 10H, C(6)H_2, C(7)H_2, C(8)H_2, C(9)H_2, C(10)H_2), 1.01 (d, *J* = 6.9 Hz, 3H, C(4)*Me*)

^{13}C NMR: (126 MHz, $CDCl_3$)
176.8 (*C*(2)), 61.2 (*C*(5)), 39.9 (*C*(4)), 38.3 (*C*(3)), 37.1, 31.9 (*C*(6), *C*(10)), 25.6, 23.5, 22.4 (*C*(7), *C*(8), *C*(9)), 14.6 (C(4)*Me*)

IR: (neat)
3172 (w), 3079 (w), 2927 (m), 2912 (m), 2866 (w), 2850 (w), 2070 (w), 1671 (s), 1449 (w), 1437 (w), 1374 (m), 1343 (w), 1274 (w), 1259 (w), 1171 (w), 1149 (w), 1139 (w), 1108 (w), 1081 (w), 1040 (w), 1005 (w), 950 (w), 937 (w), 926 (w), 906 (w), 894 (w), 849 (w), 804 (m), 770 (m), 747 (m)

MS: (ESI^+)
610 (11%), 357 (12%), 190 ($[M+Na]^+$, 100%), 168 ($[M+H]^+$, 27%)

HRMS: (ESI^+)
calcd for $C_{10}H_{17}NO^{23}Na$: 190.1202, found: 190.1208

Preparation of (*RS*)-methyl 3-(1-aminocyclohexyl)-3-phenylpropanoate (22e)

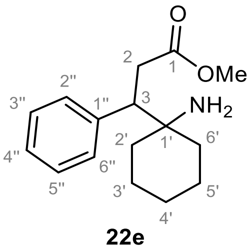

22e

Following *General Procedure 1*, cyclohexylamine **5** (52 µL, 45 mg, 0.45 mmol, 1.0 equiv), methyl cinnamate **21e** (73 mg, 0.45 mmol, 1.0 equiv), 4CzIPN (2.28 mM in MeCN, 1.98 mL, 4.5 µmol, 1 mol%), tetrabutylammonium azide (70.3 mM in MeCN, 640 µL, 45 µmol, 10 mol%), and MeCN (390 µL) were reacted for 20 h with a 425 nm LED lamp. Following concentration *in vacuo*,* purification *via* flash column chromatography on silica gel (12 g) in CH_2Cl_2 (5 CV) then 100:0:0→90:9:1 CH_2Cl_2–MeOH–aq. NH_4OH (over 15 CV) then 90:9:1 CH_2Cl_2–MeOH–aq. NH_4OH (17 CV), followed by reversed-phase flash column chromatography on C_{18} silica gel (15.5 g) in H_2O (3 CV) then 100:0→0:100 H_2O–MeOH (over 15 CV) then MeOH (16 CV), gave **22e** as a pale yellow oil (39.1 mg, 33%).

*All attempts to lactamise **22e** by refluxing with Et_3N in MeCN for 12 h, or by subjection to NaH in THF at room temperature, failed.

Data for **22e**:

^{1}H NMR: (500 MHz, $CDCl_3$)
7.27–7.12 (m, 5H, C(2″)*H*, C(3″)*H*, C(4″)*H*, C(5″)*H*, C(6″)*H*), 3.48 (s, 3H, O*Me*), 2.98–2.88 (m, 2H, C(2)*H*$_2$), 2.73–2.67 (m, 1H, C(3)*H*), 1.67–1.28 (m, 12H, N*H*$_2$, C(2′)*H*$_2$, C(3′)*H*$_2$, C(4′)*H*$_2$, C(5′)*H*$_2$, C(6′)*H*$_2$)

^{13}C NMR: (126 MHz, $CDCl_3$)
175.3 (*C*(1)), 140.0, 128.9, 128.5, 126.3 (*C*(1″), *C*(2″), *C*(3″), *C*(4″), *C*(5″), *C*(6″)), 58.1 (*C*(3)), 52.9 (*C*(1′)), 51.2 (O*Me*) , 38.1, 36.4, 33.1, 25.9, 21.9 (*C*(2′), *C*(3′), *C*(4′), *C*(5′)), *C*(6′))

IR: (neat)
3028 (w), 2925 (w), 2854 (w), 1723 (m), 1603 (w), 1495 (w), 1447 (w), 1358 (w), 1199 (w), 1153 (m), 1070 (w), 1030 (w), 998 (w), 931 (w), 910 (w), 871 (w), 838 (w), 792 (w), 747 (m), 699 (m)

MS: (ESI^+)
413 (10%), 262 ($[M+H]^+$, 100%)

HRMS: (ESI^+)

calcd for $C_{16}H_{24}NO_2$: 262.1801, found: 262.1810

Preparation of (*RS*)-4-(hydroxymethyl)-1-azaspiro[4.5]decan-2-one (23f)

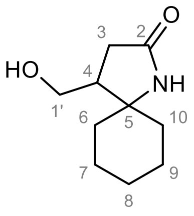

23f

Following *General Procedure 1*, cyclohexylamine **5** (52 µL, 45 mg, 0.45 mmol, 1.0 equiv), furan-2(5*H*)-one **21f** (38 mg, 0.45 mmol, 1.0 equiv), 4CzIPN (2.28 mM in MeCN, 1.98 mL, 4.5 µmol, 1 mol%), tetrabutylammonium azide (70.3 mM in MeCN, 640 µL, 45 µmol, 10 mol%), and MeCN (390 µL) were reacted for 20 h with a 425 nm LED lamp. Following concentration *in vacuo*, the residue was dissolved in MeOH (5 mL) and Et_3N (0.25 mL, 0.18 g, 1.8 mmol, 4.0 equiv) was added. The reaction was then heated at reflux for 2 h, followed by concentration *in vacuo*. Purification *via* flash column chromatography on silica gel (12 g) in CH_2Cl_2 (5 CV) then 100:0:0→90:9:1 CH_2Cl_2–MeOH–aq. NH_4OH (over 15 CV) then 90:9:1 CH_2Cl_2–MeOH–aq. NH_4OH (17 CV), followed by reversed-phase flash column chromatography on C_{18} silica gel (15.5 g) in H_2O (3 CV) then 100:0→0:100 H_2O–MeOH (over 15 CV) then MeOH (16 CV), gave **23f** as a white crystalline solid (58.9 mg, 71%).

Data for **23f**:

mp: 190–192 °C

^{1}H NMR: (500 MHz, MeOH-d_4)

3.76 (dd, J = 10.8, 5.8 Hz, 1H, C(1′)H_A), 3.57 (dd, J = 11.0, 7.1 Hz, 1H, C(1′)H_B), 2.52–2.42 (m, 1H, C(3)H_A), 2.32–2.17 (m, 2H, (C(3)H_B, C(4)*H*), 1.83–1.26 (m, 10H, C(6)H_2, C(7)H_2, C(8)H_2, C(9)H_2, C(10)H_2)

^{13}C NMR: (126 MHz, MeOH-d_4)

178.7 (*C*(2)), 62.4 (*C*(5)), 62.1 (*C*(1′)), 47.9 (*C*(4)), 39.3 (*C*(3)), 34.9, 33.0, 26.5, 23.9, 23.1 (*C*(6), *C*(7), *C*(8), *C*(9), *C*(10))

IR: (neat)

3344 (w), 3210 (w), 2923 (w), 2099 (w), 1667 (w), 1439 (w), 1392 (w), 1256 (w), 1169 (w), 1070 (w), 1032 (w), 888 (w), 800 (w), 740 (w)

MS: (ESI$^+$)

389 (27%), 206 ([M+Na]$^+$, 100%), 184 ([M+H]$^+$, 18%)

HRMS: (ESI$^+$)

calcd for $C_{10}H_{17}NO_2{}^{23}Na$: 206.1152, found: 206.1158

Preparation of *tert*-butyl 3-(1-aminocyclohexyl)propanoate (22g)

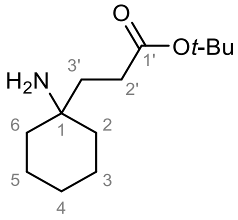

22g

Following *General Procedure 1*, cyclohexylamine **5** (52 µL, 45 mg, 0.45 mmol, 1.0 equiv), *tert*-butyl acrylate **21g** (58 mg, 66 µL, 0.45 mmol, 1.0 equiv), 4CzIPN (2.28 mM in MeCN, 1.98 mL, 4.5 µmol, 1 mol%), tetrabutylammonium azide (70.3 mM in MeCN, 640 µL, 45 µmol, 10 mol%), and MeCN (390 µL) were reacted for 20 h with a 425 nm LED lamp. Following concentration *in vacuo*, purification *via* flash column chromatography on silica gel (12 g) in CH_2Cl_2 (5 CV) then 100:0:0→90:9:1 CH_2Cl_2–MeOH–aq. NH_4OH (over 15 CV) then 90:9:1 CH_2Cl_2–MeOH–aq. NH_4OH (17 CV) gave **22g** as a light brown oil (69.1 mg, 68%).

Data for **22g**:

^{1}H NMR: (500 MHz, $CDCl_3$)

2.28 (m, 2H, C(2′)*H*$_2$), 1.68–1.61 (m, 2H, C(3′)*H*$_2$), 1.54–1.20 (m, 21H, N*H*$_2$, C(2)*H*$_2$, C(3)*H*$_2$, C(4)*H*$_2$, C(5)*H*$_2$, C(6)*H*$_2$, O*t-Bu*)

^{13}C NMR: (126 MHz, $CDCl_3$)

173.9 (*C*(1′)), 80.2 (O*C*(CH$_3$)$_3$)), 50.4 (*C*(1)), 38.7 (*C*(2), *C*(6)), 37.3 (*C*(3′)), 29.9 (*C*(2′)), 28.2 (OC(*C*H$_3$)$_3$), 26.1 (*C*(3), *C*(5)), 22.3 (*C*(4))

IR: (neat)

3268 (w), 3125 (w), 2929 (w), 2857 (w), 1735 (m), 1641 (m), 1571 (w), 1538 (m), 1475 (w), 1450 (w), 1436 (w), 1421 (w), 1366 (w), 1331 (w), 1311 (m), 1292 (m), 1246 (w), 1219 (w), 1191 (m), 1169 (m), 1147 (m), 1137 (m), 1082 (w), 1030 (w), 991 (w), 972 (w), 950 (w), 932 (w), 896 (w), 886 (w), 846 (w), 779 (w), 756 (m), 682 (w)

MS: (ESI$^+$)

229 (13%), 228 ([M+H]$^+$, 100%)

HRMS: (ESI$^+$)

calcd for $C_{13}H_{26}NO_2$: 228.1958, found: 228.1969

Preparation of 1-[2-(pyridin-4-yl)ethyl]cyclohexan-1-amine (22h)

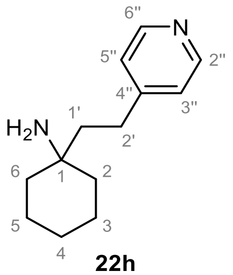

22h

Following *General Procedure 1*, cyclohexylamine **5** (52 µL, 45 mg, 0.45 mmol, 1.0 equiv), 4-vinylpyridine **21h** (47 mg, 0.45 mmol, 1.0 equiv), 4CzIPN (2.28 mM in MeCN, 1.98 mL, 4.5 µmol, 1 mol%), tetrabutylammonium azide (70.3 mM in MeCN, 640 µL, 45 µmol, 10 mol%), and MeCN (390 µL) were reacted for 20 h with a 425 nm LED lamp. Following concentration *in vacuo*, purification *via* flash column chromatography on silica gel (12 g) in CH_2Cl_2 (5 CV) then 100:0:0→90:9:1 CH_2Cl_2–MeOH–aq. NH_4OH (over 15 CV) then 90:9:1 CH_2Cl_2–MeOH–aq. NH_4OH (17 CV), followed by reversed-phase flash column chromatography on C_{18} silica gel (15.5 g) in H_2O (3 CV) then 100:0→0:100 H_2O–MeOH (over 15 CV) then MeOH (16 CV), gave **22h** as a yellow oil (54.0 mg, 59%).

Data for **22h**:

^{1}H NMR: (500 MHz, $CDCl_3$)

8.51–8.44 (m, 2H, C(2″)*H*, C(6″)*H*), 7.16–7.10 (m, 2H, C(3″)*H*, C(5″)*H*), 2.71–2.63 (m, 2H, C(2′)H_2), 1.64–1.22 (m, 14H, NH_2, C(1′)H_2, C(2)H_2, C(3)H_2, C(4)H_2, C(5)H_2, C(6)H_2)

^{13}C NMR: (126 MHz, $CDCl_3$)

152.2 (*C*(4″)), 149.85 (*C*(2″), C(6″)), 124.0 (*C*(3″), *C*(5″)), 51.05 (*C*(1)), 43.4 (*C*(1′)), 38.7 (*C*(2), *C*(6)), 29.1 (*C*(2′)), 26.0 (*C*(4)), 22.3 (*C*(3), *C*(5))

IR: (neat)

3349 (w), 3025 (w), 2924 (w), 2852 (w), 2021 (w), 1682 (w), 1600 (m), 1559 (w), 1497 (w), 1449 (w), 1417 (w), 1335 (w), 1220 (w), 1168 (w), 1098 (w), 1056 (w), 993 (w), 954 (w), 897 (w), 844 (w), 805 (m), 685 (w)

MS: (ESI$^+$)

206 (14%), 205 ([M+H]$^+$, 100%)

HRMS: (ESI$^+$)

calcd for $C_{13}H_{21}N_2$: 205.1699 found: 205.1705

Preparation of 1-[2-(pyridin-2-yl)ethyl]cyclohexan-1-amine (22i)

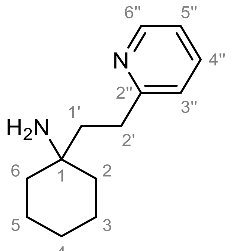

22i

Following *General Procedure 1*, cyclohexylamine **5** (52 µL, 45 mg, 0.45 mmol, 1.0 equiv), 2-vinylpyridine **21i** (47 mg, 0.45 mmol, 1.0 equiv), 4CzIPN (2.28 mM in MeCN, 1.98 mL, 4.5 µmol, 1 mol%), tetrabutylammonium azide (70.3 mM in MeCN, 640 µL, 45 µmol, 10 mol%), and MeCN (390 µL) were reacted for 20 h with a 425 nm LED lamp. Following concentration *in vacuo*, purification *via* flash column chromatography on Biotage® KP-NH silica gel (10 g, 10 mm Ø) in 90:9:1 CH_2Cl_2–MeOH–aq. NH_4OH (over 15 CV), followed by re-purification of mixed fractions under the same conditions (with 8 g KP-NH silica), gave **22i** as a yellow oil (54.9 mg, 59%).

Data for **22i**:

^{1}H NMR: (500 MHz, $CDCl_3$)

8.48 (d, J = 5.0 Hz, 1H, C(6″)H), 7.54 (t, J = 7.7 Hz, 1H, C(4″)H), 7.13 (d, J = 7.8 Hz, 1H, C(3″)H), 7.05 (dd, J = 7.4, 5.1 Hz, 1H, C(5″)H), 2.85–2.77 (m, 2H, C(2′)H_2), 1.79–1.71 (m, 2H, C(1′)H_2), 1.56–1.24 (m, 12H, NH_2, C(1)H_2, C(2)H_2, C(3)H_2, C(4)H_2, C(5)H_2, C(6)H_2)

^{13}C NMR: (126 MHz, $CDCl_3$)

162.9 (C(2″)), 149.3 (C(6″)), 136.4 (C(4″)), 122.8 (C(3″)), 120.9 (C(5″)), 50.6 (C(1)), 42.9 (C(1′)), 38.8 (C(2), C(6)), 32.2 (C(2′)), 26.1 (C(3), C(5)), 22.3 (C(4))

IR: (neat)

3290 (w), 2922 (m), 2851 (w), 2163 (w), 2123 (w), 1837 (w), 1590 (m), 1568 (w), 1474 (w), 1450 (w), 1434 (m), 1149 (w), 1051 (w), 993 (w), 904 (w), 750 (m), 664 (m)

MS: (ESI$^+$)

206 (13%), 205 ([M+H]$^+$, 100%), 188 (33%)

HRMS: (ESI$^+$)

calcd for $C_{13}H_{21}N_2$: 205.1699, found: 205.1707

E.6. Unsuccessful Substrates

The following amines were not successful in the α-C–H alkylation process:

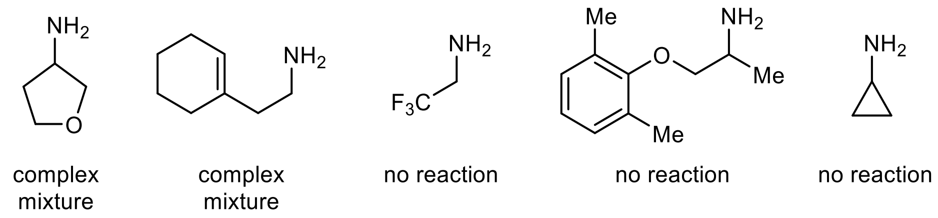

The following Michael acceptors were not successful in the α-C–H alkylation process:

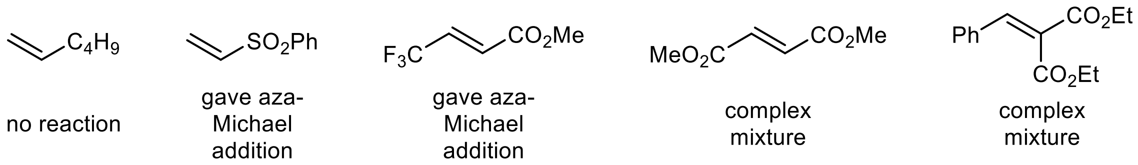

Fig. S8. Unsuccessful substrates in our α-C–H alkylation reaction.

F. Synthesis of α-Tertiary Amine Derivatives 24a–f in Flow

F.1. Optimisation on Vapourtec UV-150 Photoreactor

F.1.1. Optimising the Lamp Power and Residence Time

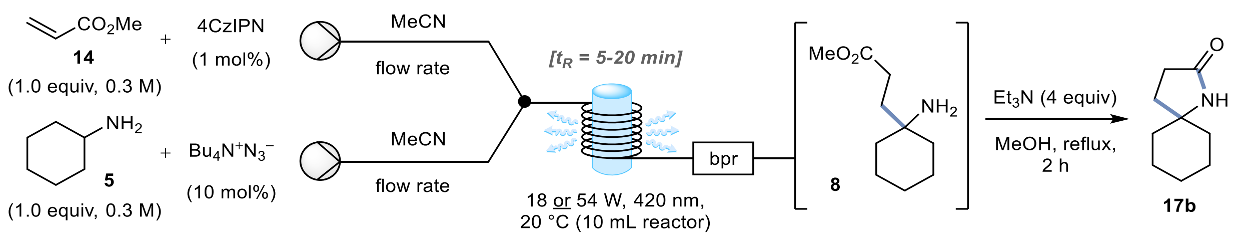

The following solutions were prepared in 100-mL volumetric flasks under N_2, and transferred to 250-mL, round-bottomed flasks capped with rubber septa under an N_2 atmosphere:

Reagent feed A: A 100-mL solution of methyl acrylate **14** (2.70 mL, 2.58 g, 30.0 mmol, 1.0 equiv) and 4CzIPN (237 mg, 0.30 mmol, 1 mol%) in dry MeCN.

Reagent feed B: A 100-mL solution of cyclohexylamine **5** (3.43 mL, 2.98 g, 30.0 mmol, 1.0 equiv) and tetrabutylammonium azide (854 mg, 3.00 mmol, 10 mol%) in dry MeCN.

A Vapourtec E-series flow reactor equipped with a UV-150 10-mL photoreactor and dry-ice cooling module was used. After priming the reagent lines for feeds A and B, and flushing the system with dry MeCN, 5.0 mL portions of feeds A and B were injected simultaneously into the photoreactor at various flow rates (residence time: 5–20 min[19]), mixed in a T-mixer and passed through a 10 mL coil (0.8 mm inner diameter, fluoropolymer tube), irradiated with a 420 nm LED array (18 W or 54 W radiant output power) at 20 °C. The pressure was kept around 1 bar by using the third pump as a back-pressure regulator (BPR). After the entire 10-mL mixture had entered the reactor, it was followed with dry MeCN at the same flow rate. A 3.00-mL aliquot of the steady-state product mixture was collected and transferred to a microwave vial, then concentrated *in vacuo* on a spiral evaporator. Et_3N (0.42 mL, 304 mg, 3.00 mmol) and MeOH (3.0 mL) were added, and the vial was crimp-sealed and heated at 70 °C in an aluminium block for 2 h. After cooling to rt, the vial was de-crimped and the mixture was concentrated *in vacuo* on a spiral evaporator, before subjection to ^{1}H NMR analysis. The NMR yield of **17b** was calculated using the tetrabutylammonium ion as an internal standard, using the resonance at δ_H = 3.35 (8H, m) as a reference peak.

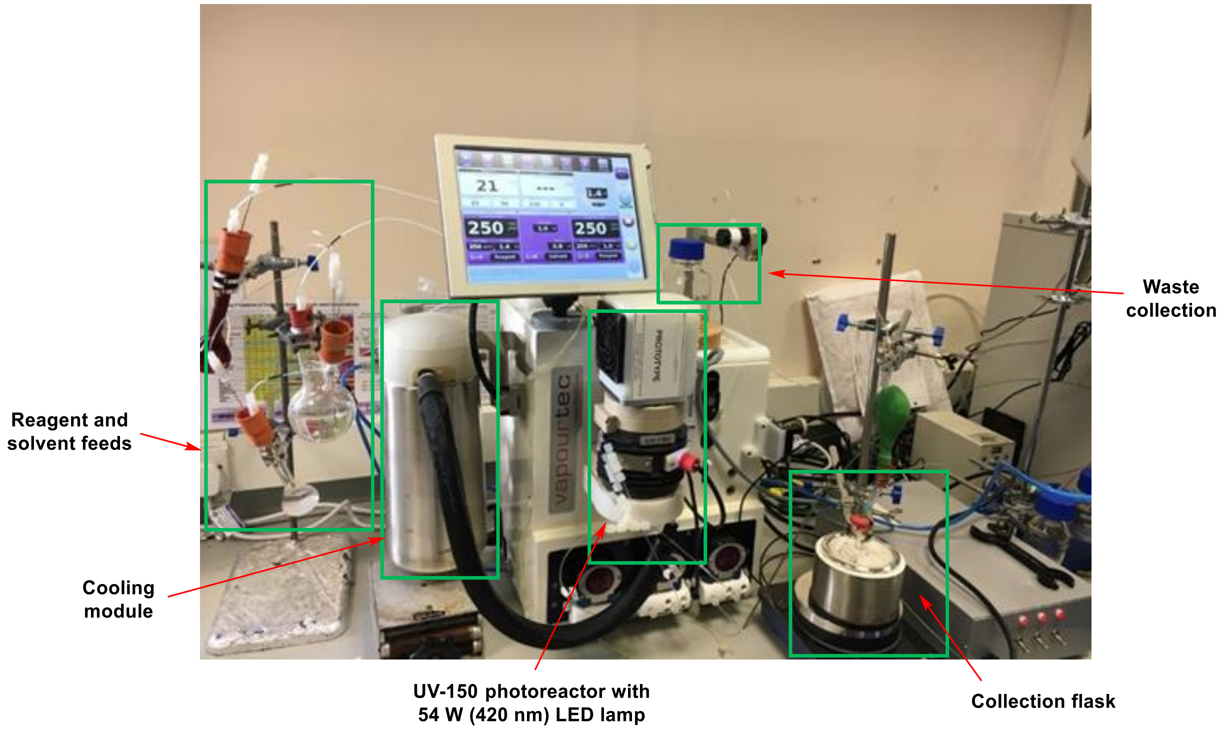

Fig. S9. Photograph of the set-up for flow photochemistry using a Vapourtec E-series.

^{1}H NMR spectroscopic data for methyl 3-(1-aminocyclohexyl)-propanoate **8** is given below:

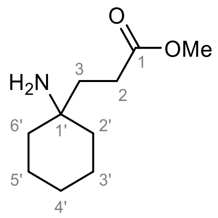

8

<u>^{1}H NMR:</u> (300 MHz, $CDCl_3$)

3.65 (s, 3H, O*Me*), 2.44–2.30 (m, 2H, C(2)H_2), 1.72–1.35 (m, 14H, NH_2, C(3)H_2, C(2′)H_2, C(3′)H_2, C(4′)H_2, C(5′)H_2, C(6′)H_2)

<u>^{1}H NMR:</u> (300 MHz, MeCN-d_6)

3.57 (s, 3H, O*Me*), 2.38–2.26 (m, 2H, C(2)H_2), 1.66–1.22 (m, 14H, NH_2, C(3)H_2, C(2′)H_2, C(3′)H_2, C(4′)H_2, C(5′)H_2, C(6′)H_2)

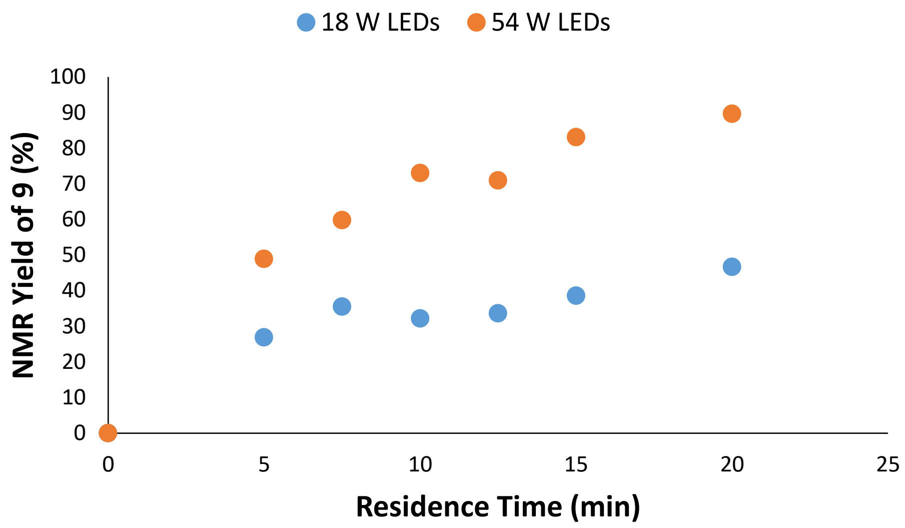

Fig. S10. NMR yield of **17b** as a function of both residence time and LED radiant output power.

F.1.2. Attempts to Perform Lactamisation Step in Continuous Flow by Heating

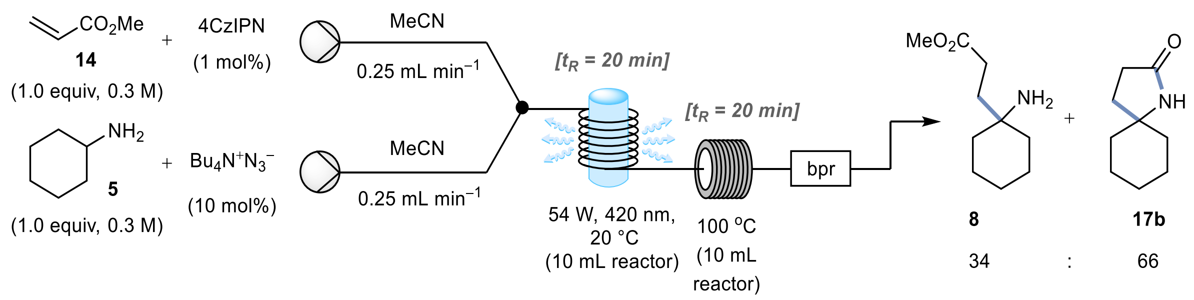

We also attempted to perform lactamisation of the intermediate **8** in continuous flow by passing through a heated reactor after the UV-150 photoreactor, but even at 100 °C (20 min residence time) the lactamisation remained incomplete (66% conversion). Switching the reaction solvent to 90:10 MeCN/*t*-AmOH and subsequent heating at 100 °C also proved ineffective, with the lactamisation conversion only 47%.

F.2. Synthesis of α-Tertiary Amine Derivatives

*F.2.1. Determining the Concentration of γ-Amino Ester Intermediate **8** Produced In Flow*

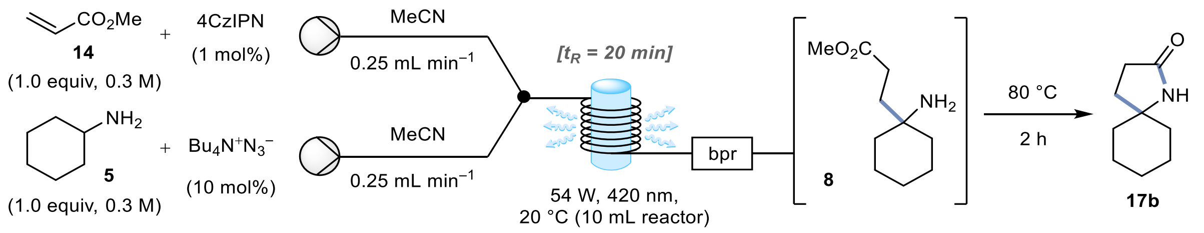

The following solutions were prepared in 50-mL volumetric flasks under N_2:

Reagent feed A: A 50-mL solution of methyl acrylate **14** (1.36 mL, 1.30 g, 15.0 mmol, 1.0 equiv) and 4CzIPN (118 mg, 0.15 mmol, 1 mol%) in dry MeCN.

Reagent feed B: A 50-mL solution of cyclohexylamine **5** (1.72 mL, 1.50 g, 15.0 mmol, 1.0 equiv) and tetrabutylammonium azide (427 mg, 1.50 mmol, 10 mol%) in dry MeCN.

A Vapourtec E-series flow reactor equipped with a UV-150 10-mL photoreactor and dry-ice cooling module was used. After priming the reagent lines for feeds A and B, and flushing the system with dry MeCN, 5.0 mL portions of feeds A and B were injected simultaneously into the photoreactor at a flow rate of 0.25 mL min^{-1} (residence time: 20 min), mixed in a T-mixer and passed through a 10 mL coil (0.8 mm inner diameter, fluoropolymer tube), irradiated with a 420

nm LED array (54 W radiant output power) at 20 °C. The pressure was kept around 1 bar by using the third pump as a back-pressure regulator (BPR). After the entire 10-mL mixture had entered the reactor, it was followed with dry MeCN at the same flow rate. A 5-mL aliquot of the central part of the steady-state product mixture was collected over the time period 28–38 min, as a dark orange solution. From this solution, 3 × 1 mL aliquots were taken and transferred to microwave vials, then crimped and heated at 80 °C for 2 h. After allowing to cool to rt, the vials were de-crimped and the mixtures were each concentrated *in vacuo* on a spiral evaporator. Mesitylene (2.0 µL, 0.019 mmol) was added to each sample as an internal standard, followed by subjection to ^{1}H NMR analysis. The NMR yield for **17b** (80% across all three aliquots) was calculated using the tetrabutylammonium ion as an internal standard, using the resonance at δ_H = 3.35 (8H, m) as a reference peak. Assuming the lactamisation of **17b** is quantitative, the concentration of **8** in the steady-state product mixture was calculated as 0.08 M by reference to the mesitylene internal standard, using the resonance at δ_H = 6.77 (3H, s). The concentration of tetrabutylammonium azide in the steady-state product mixture was also calculated as 0.0088 M.

*F.2.2. Determining the Quantity of Electrophile Needed to Trap Flow-Generated **8***

For carrying out *N*-functionalisations of flow-generated **8** with electrophiles, we first needed to calculate the quantity of electrophile needed. An excess of electrophile is necessary due to the presence of unreacted cyclohexylamine **5** in the product flow stream, as well as the potential for reaction of the azide ion catalyst with the electrophilic reagent. Thus, in a 5-mL aliquot of the steady-state product mixture, the total quantity of reactive amine present (i.e., **5** + **8**) is given by:

$$\text{mmol of } \mathbf{8} + \mathbf{5} = \frac{\text{mmol of } \mathbf{8} \text{ produced in flow}}{\text{fractional yield of } \mathbf{8}} = \frac{0.40}{0.80} = 0.50$$

Assuming that azide ion is also capable of quenching the electrophile, we can calculate the minimum quantity of electrophile required by:

$$\text{mmol of electrophile} = \text{mmol of } \mathbf{8} + \mathbf{5} + \text{azide ion} = 0.50 + 0.044 = 0.544$$

Thus, for a 20% excess of electrophile (i.e., 1.2 equiv), we need to use 0.544 × 1.2 = 0.65 mmol of electrophile (i.e., 1.63 equiv w.r.t. **8**).

*F.2.3. N-Functionalisation of Flow-Generated γ-Amino Ester Intermediates **8** or **22g***

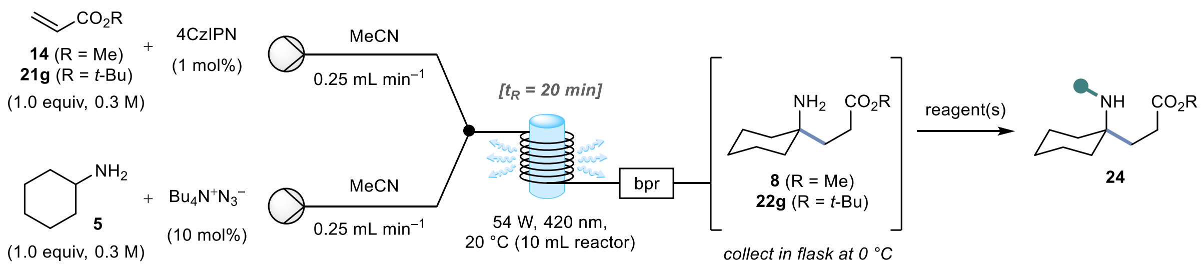

The flow reaction was set up and run as per the procedure in section F.2.1. For generation of γ-amino ester **22g** (R = *t*-Bu), methyl acrylate **14** was substituted for *tert*-butyl acrylate **21g**, but all other parameters were kept unchanged (and the calculated concentration and NMR yield of **22g** was found to be identical to **8**). As before, a 5-mL aliquot of the central part of the steady-state product mixture was collected over the time period 28–38 min, as a dark orange solution. This was collected in a nitrogen-flushed, round-bottomed flask equipped with a stirrer bar, sealed with a rubber septum, and immersed in an ice-water bath. The requisite electrophile (and any other reagents) needed for *N*-functionalisation were then added dropwise to the reaction mixture (or, if stated, the inverse of this addition was performed).

Preparation of methyl *N*-[(9*H*-fluoren-9-ylmethoxy)carbonyl]-3-(1-aminocyclohexyl)-propanoate (24a)

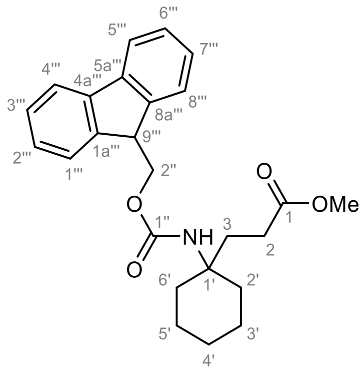

24a

Following the general procedure described above, 5.0 mL of a steady-state aliquot of crude γ-amino ester **8** (0.40 mmol) was added dropwise to a stirred solution of 9-fluorenylmethoxycarbonyl chloride (170 mg, 0.65 mmol, 1.63 equiv) in MeCN (1.0 mL) at 0 °C under an N_2 atmosphere. The reaction mixture was stirred at 0 °C for 3 h, then was allowed to

warm to rt and was partitioned between CH_2Cl_2 (25 mL) and H_2O (25 mL). The layers were separated, and the aqueous phase was extracted with CH_2Cl_2 (2 × 25 mL). The combined organic layers were dried ($MgSO_4$), filtered, and concentrated *in vacuo*. Purification *via* flash column chromatography on silica gel (12 g) in CH_2Cl_2 (5 CV) then 100:0:0→90:9:1 CH_2Cl_2–MeOH–aq. NH_4OH (over 15 CV) then 90:9:1 CH_2Cl_2–MeOH–aq. NH_4OH (17 CV), followed by reversed-phase flash column chromatography on C_{18} silica gel (15.5 g) in H_2O (3 CV) then 100:0→0:100 H_2O–MeOH (over 15 CV) then MeOH (16 CV), gave **24a** as an orange gum (102 mg, 63%).

Data for **24a**:

^{1}H NMR: (500 MHz, $CDCl_3$)
7.76 (d, *J* = 7.5 Hz, 2H, C(4‴)*H*, C(5‴)*H*), 7.59 (d, *J* = 7.5 Hz, 2H, C(1‴)*H*, C(8‴)*H*), 7.40 (app t, *J* = 7.5 Hz, 2H, C(3‴)*H*, C(6‴)*H*), 7.32 (app td, *J* = 7.5, 1.0 Hz, 2H, C(2‴)*H*, C(7‴)*H*), 4.51–4.34 (m, 3H, N*H*, C(2″)H_2), 4.20 (t, *J* = 6.5 Hz, 1H, C(9‴)*H*), 3.64 (s, 3H, O*Me*), 2.34–2.20 (m, 2H, C(2)H_2), 2.13–1.87 (m, 4H, C(3)H_2, C(2′)H_A, C(6′)H_A), 1.65–1.20 (m, 8H, C(2′)H_B, C(3′)H_2, C(4′)H_2, C(5′)H_2, C(6′)H_B)

^{13}C NMR: (126 MHz, $CDCl_3$)
174.3 (*C*(1)), 154.3 (*C*(1″)), 144.0 (*C*(4a‴), *C*(5a‴)), 141.4 (*C*(1a‴), *C*(8a‴)), 127.6 (*C*(3‴), *C*(6‴)), 127.0 (*C*(2‴), *C*(7‴)), 124.95 (*C*(1‴), *C*(8‴)), 119.9 (*C*(4‴), *C*(5‴)), 65.7 (*C*(2″)), 54.3 (*C*(1′)), 51.6 (O*Me*), 47.5 (*C*(9‴)), 34.7 (*C*(2′), *C*(6′)), 33.4 (*C*(3)), 28.5 (*C*(2)), 25.6 (*C*(3′), *C*(5′)), 21.6 (*C*(4′))

IR: (neat)
3357 (w), 2932 (w), 2856 (w), 2253 (w), 2160 (w), 2025 (w), 1982 (w), 1707 (m), 1505 (m), 1478 (w), 1449 (m), 1328 (w), 1305 (w), 1239 (m), 1172 (m), 1093 (m), 1041 (w), 970 (w), 909 (w), 854 (w), 759 (m), 736 (m)

MS: (ESI^+)
430 ($[M+Na]^+$, 100%), 408 ($[M+H]^+$, 67%)

HRMS: (ESI^+)
calcd for $C_{25}H_{29}NO_4{}^{23}Na$: 430.1989, found: 430.1995

Preparation of methyl *N*-(2,2,2-trifluoroacetyl)-3-(1-aminocyclohexyl)propanoate (24b)

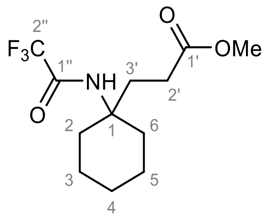

24b

Following the general procedure described above, trifluoroacetic anhydride (92 µL, 125 mg, 0.65 mmol, 1.63 equiv) and Et_3N (91 µL, 66 mg, 0.65 mmol, 1.63 equiv) were added dropwise to 5.0 mL of a steady-state aliquot of crude γ-amino ester **8** (0.40 mmol) at 0 °C under an N_2 atmosphere. The reaction mixture was stirred at 0 °C for 3 h, then was allowed to warm to rt and was partitioned between CH_2Cl_2 (25 mL) and sat. $NaHCO_3$ (25 mL). The layers were separated, and the aqueous phase was extracted with CH_2Cl_2 (2 × 25 mL). The combined organic layers were dried ($MgSO_4$), filtered, and concentrated *in vacuo*. Purification *via* flash column chromatography on silica gel (12 g) in CH_2Cl_2 (5 CV) then 100:0:0→90:9:1 CH_2Cl_2–MeOH–aq. NH_4OH (over 15 CV) then 90:9:1 CH_2Cl_2–MeOH–aq. NH_4OH (17 CV), followed by reversed-phase flash column chromatography on C_{18} silica gel (15.5 g) in H_2O (3 CV) then 100:0→0:100 H_2O–MeOH (over 15 CV) then MeOH (16 CV), gave **24b** as a white solid (53.2 mg, 47%).

Data for **24b**:

mp: 58–60 °C

^{1}H NMR: (500 MHz, $CDCl_3$)
5.96 (s, 1H, N*H*), 3.65 (s, 3H, O*Me*), 2.34–2.25 (m, 2H, C(2′)H_2), 2.18–2.04 (m, 4H, C(2)H_A, C(6)H_A, C(3′)H_2), 1.65–1.51 (3H, C(3)H_A, C(5)H_A, C(4)H_A), 1.49–1.22 (m, 5H, C(2)H_B, C(3)H_B, C(5)H_B, C(4)H_B, C(6)H_B)

^{13}C NMR: (126 MHz, $CDCl_3$)
173.8 (*C*(1′)), 156.1 (q, *J* = 35.9 Hz, (*C*(1″))), 115.6 (q, *J* = 289 Hz, (*C*(2″))), 57.0 (*C*(1)), 51.8 (O*Me*), 34.0 (*C*(2), *C*(6)), 32.4 (*C*(3′)), 28.3 (*C*(2′)), 25.2 (*C*(3), *C*(5)), 21.7 (*C*(4))

IR: (neat)
3299 (w), 3101 (w), 2930 (w), 2861 (w), 1738 (m), 1724 (m), 1698 (m), 1562 (m), 1455 (w), 1438 (w), 1416 (w), 1373 (w), 1359 (w), 1326 (w), 1299 (w), 1238 (w), 1187 (s), 1150 (s), 1056 (w), 999 (w), 988 (w), 966 (w), 936 (w), 908 (w), 888 (w), 875 (w), 854 (w), 836 (w), 806 (w), 789 (w), 770 (w), 748 (w), 723 (w), 707 (m),

667 (m)

MS: (ESI$^+$)

304 ([M+Na]$^+$, 100%), 305 (13%), 282 ([M+H]$^+$, 17%)

HRMS: (ESI$^+$)

calcd for $C_{12}H_{18}F_3NO_3{}^{23}Na$: 304.1131, found: 304.1139

Preparation of methyl *N*-benzoyl-3-(1-aminocyclohexyl)propanoate (24c)

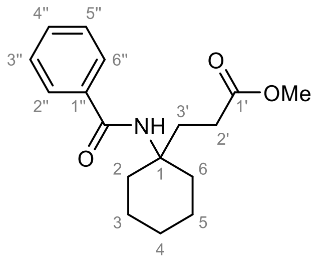

24c

Following the general procedure described above, benzoyl chloride (76 µL, 92 mg, 0.65 mmol, 1.63 equiv) and Et_3N (91 µL, 66 mg, 0.65 mmol, 1.63 equiv) were added dropwise to 5.0 mL of a steady-state aliquot of crude γ-amino ester **8** (0.40 mmol) at 0 °C under an N_2 atmosphere. The reaction mixture was stirred at 0 °C for 3 h, then was allowed to warm to rt and was partitioned between CH_2Cl_2 (25 mL) and H_2O (25 mL). The layers were separated, and the aqueous phase was extracted with CH_2Cl_2 (3 × 25 mL). The combined organic layers were dried ($MgSO_4$), filtered, and concentrated *in vacuo*. Purification *via* flash column chromatography on silica gel (12 g) in CH_2Cl_2 (5 CV) then 100:0:0→90:9:1 CH_2Cl_2–MeOH–aq. NH_4OH (over 15 CV) then 90:9:1 CH_2Cl_2–MeOH–aq. NH_4OH (17 CV), followed by reversed-phase flash column chromatography on C_{18} silica gel (15.5 g) in H_2O (3 CV) then 100:0→0:100 H_2O–MeOH (over 15 CV) then MeOH (16 CV), gave **24c** as a white solid (66.9 mg, 58%).

Data for **24c**:

mp: 95–96 °C

^{1}H NMR: (500 MHz, $CDCl_3$)

7.75–7.69 (m, 2H, C(2″)*H*, C(6″)*H*), 7.49–7.44 (m, 1H, C(4″)*H*), 7.43–7.37 (m, 2H, C(3″)*H*, C(5″)*H*), 5.78 (s, 1H, N*H*), 3.59 (s, 3H, O*Me*), 2.37–2.29 (m, 2H, C(2′)H_2), 2.29–2.22 (m, 2H, C(2)H_A, C(6)H_A), 2.22–2.17 (m, 2H, C(3′)H_2), 1.66–1.19 (m, 8H, C(2)H_B, C(3)H_2, C(4)H_2, C(5)H_2, C(6)H_B)

<u>^{13}C NMR:</u> (126 MHz, $CDCl_3$)

174.4 (*C*(1′)), 166.9 (NH*C*=O), 135.8 (*C*(1″)), 131.3 (*C*(4″)), 128.65 (*C*(3″), *C*(5″)), 126.8 (*C*(2″) *C*(6″)), 55.9 (*C*(1)), 51.7 (O*Me*)), 34.8 (*C*(2), *C*(6)), 33.3 (*C*(3′)), 28.8 (*C*(2′)), 25.7 (*C*(3), *C*(5)), 21.9 (*C*(4))

<u>IR:</u> (neat)

3312 (w), 2928 (w), 2858 (w), 2116 (w), 1732 (w), 1633 (w), 1579 (w), 1530 (w), 1490 (w), 1447 (w), 1435 (w), 1378 (w), 1333 (w), 1312 (w), 1288 (w), 1216 (w), 1194 (w), 1169 (w), 1077 (w), 1028 (w), 995 (w), 970 (w), 929 (w), 896 (w), 870 (w), 804 (w), 783 (w), 715 (w), 690 (w), 663 (w)

<u>MS:</u> (ESI^+)

313 (18%), 312 ($[M+Na]^+$, 100%), 291 (10%), 290 ($[M+H]^+$, 82%)

<u>HRMS:</u> (ESI^+)

calcd for $C_{17}H_{23}NO_3Na$: 312.3648, found: 312.1579

Preparation of methyl *N*-(2-furoyl)-3-(1-aminocyclohexyl)propanoate (24d)

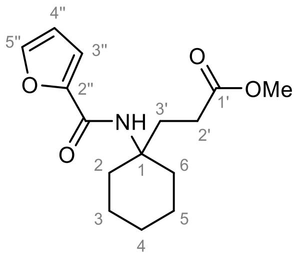

24d

Following the general procedure described above, furoyl chloride (64 μL, 85 mg, 0.65 mmol, 1.63 equiv) and Et_3N (91 μL, 66 mg, 0.65 mmol, 1.63 equiv) were added dropwise to 5.0 mL of a steady-state aliquot of crude γ-amino ester **8** (0.40 mmol) at 0 °C under an N_2 atmosphere. The reaction mixture was stirred at 0 °C for 3 h, then was allowed to warm to rt and was partitioned between CH_2Cl_2 (25 mL) and H_2O (25 mL). The layers were separated, and the aqueous phase was extracted with CH_2Cl_2 (3 × 25 mL). The combined organic layers were dried ($MgSO_4$), filtered, and concentrated *in vacuo*. Purification *via* flash column chromatography on silica gel (12 g) in CH_2Cl_2 (5 CV) then 100:0:0→90:9:1 CH_2Cl_2–MeOH–aq. NH_4OH (over 15 CV) then 90:9:1 CH_2Cl_2–MeOH–aq. NH_4OH (17 CV), followed by reversed-phase flash column chromatography on C_{18} silica gel (15.5 g) in H_2O (3 CV) then 100:0→0:100 H_2O–MeOH (over 15 CV) then MeOH (16 CV), gave **24d** as a cream solid (58.1 mg, 52%).

Data for **24d**:

mp: 86–88 °C

^{1}H NMR: (500 MHz, $CDCl_3$)
7.41 (dd, *J* = 1.8, 0.8 Hz, 1H, C(3″)*H*), 7.04 (dd, *J* = 3.5, 0.9 Hz, 1H, C(5″)*H*), 6.48 (dd, *J* = 3.5, 1.8 Hz, 1H, C(4″)*H*), 5.97 (s, 1H, N*H*), 3.60 (s, 3H, O*Me*), 2.40–2.29 (m, 2H, C(2′)H_2), 2.25–2.19 (m, 2H, C(3′)H_2), 2.19–2.12 (m, 2H, C(2)H_A, C(6)H_A), 1.59–1.25 (m, 8H, C(2)H_B, C(3)H_2, C(4)H_2, C(5)H_2, C(6)H_B)

^{13}C NMR: (126 MHz, $CDCl_3$)
174.4 (*C*(1′)), 157.8 (NH*C*=O), 148.6 (*C*(2″)), 143.6 (*C*(3″)), 113.8 (*C*(5″)), 112.3 (*C*(4″)), 55.8 (*C*(1)), 51.7 (O*Me*), 35.0 (*C*(2), *C*(6)), 33.6 (*C*(3′)), 28.8 (*C*(2′)), 25.7 (*C*(3), *C*(5)), 21.8 (*C*(4))

IR: (neat)
3270 (w), 3124 (w), 2929 (w), 2855 (w), 1737 (m), 1640 (m), 1570 (m), 1535 (m), 1475 (w), 1450 (w), 1435 (m), 1420 (w), 1378 (w), 1331 (w), 1308 (m), 1245 (m), 1219 (w), 1191 (m), 1169 (m), 1145 (m), 1135 (m), 1081 (w), 1053 (w), 1029 (w), 989 (w), 971 (w), 949 (w), 931 (w), 896 (w), 885 (w), 844 (w), 779 (w), 764 (m), 756 (m), 674 (w)

MS: (ESI^+)
303 (15%), 302 ($[M+Na]^+$, 100%), 280 ($[M+H]^+$, 45%)

HRMS: (ESI^+)
calcd for $C_{15}H_{21}NO_4{}^{23}Na$: 302.1363, found: 302.1373

Preparation of methyl 3-(1-(pyridine-3-sulfonamido)cyclohexyl)propanoate (24e)

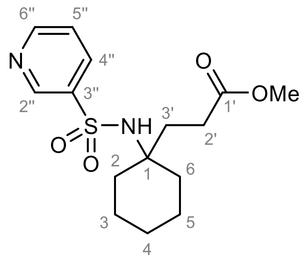

24e

Following the general procedure described above, pyridine-3-sulfonyl chloride (80 µL, 116 mg, 0.65 mmol, 1.63 equiv) and Et_3N (91 µL, 66 mg, 0.65 mmol, 1.63 equiv) were added dropwise to 5.0 mL of a steady-state aliquot of crude γ-amino ester **8** (0.40 mmol) at 0 °C under an N_2

atmosphere. The reaction mixture was stirred at 0 °C for 3 h, then was allowed to warm to rt and was partitioned between CH_2Cl_2 (25 mL) and H_2O (25 mL). The layers were separated, and the aqueous phase was extracted with CH_2Cl_2 (3 × 25 mL). The combined organic layers were dried ($MgSO_4$), filtered, and concentrated *in vacuo*. Purification *via* flash column chromatography on silica gel (12 g) in CH_2Cl_2 (5 CV) then 100:0:0→90:9:1 CH_2Cl_2–MeOH–aq. NH_4OH (over 15 CV) then 90:9:1 CH_2Cl_2–MeOH–aq. NH_4OH (17 CV), followed by reversed-phase flash column chromatography on C_{18} silica gel (15.5 g) in H_2O (3 CV) then 100:0→0:100 H_2O–MeOH (over 15 CV) then MeOH (16 CV), gave **24e** as a colourless oil (65.0 mg, 50%).

Data for **24e**:

^{1}H NMR: (500 MHz, $CDCl_3$)
9.11 (s, 1H, C(2″)*H*), 8.77 (d, *J* = 4.7 Hz, 1H, C(6″)*H*), 8.18 (d, *J* = 8.1 Hz, 1H, C(4″)*H*), 7.44 (dd, *J* = 7.3, 5.1, 1.6 Hz, 1H, C(5″)*H*), 5.05 (s, 1H, N*H*), 3.64 (s, 3H, O*Me*), 2.30 (t, *J* = 7.8 Hz, 2H, C(2′)H_2), 2.00 (t, *J* = 7.9 Hz, 2H, C(3′)H_2), 1.70 (m, 2H, C(2)H_A, C(6)H_A), 1.40–1.23 (m, 8H, C(2)H_B, C(3)H_2, C(4)H_2, C(5)H_2, C(6)H_B)

^{13}C NMR: (126 MHz, $CDCl_3$)
174.0 (*C*(1′)), 152.8 (*C*(6″)), 148.0 (*C*(2″)), 140.05 (*C*(3″)), 134.6 (*C*(4″)), 123.7 (*C*(5″)), 60.0 (*C*(1)), 51.9 (O*Me*), 35.9 (*C*(2), *C*(6)), 33.4 (*C*(3′)), 28.25 (*C*(2′)), 25.2 (*C*(3), *C*(5)), 21.7 (*C*(4))

IR: (neat)
3318 (w), 2948 (w), 2856 (w), 2162 (w), 2041 (w), 1732 (m), 1598 (w), 1498 (w), 1461 (w), 1436 (w), 1414 (w), 1379 (w), 1322 (w), 1301 (m), 1219 (w), 1186 (w), 1158 (m), 1147 (m), 1093 (m), 1045 (w), 1021 (w), 991 (m), 954 (w), 931 (w), 888 (w), 850 (w), 821 (m), 784 (w), 709 (w), 696 (w), 666 (m)

MS: ($ESI^–$)
325 ($[M–H]^–$, 100%), 326 (16%)

HRMS: ($ESI^–$)
calcd for $C_{15}H_{21}N_2O_4S$: 325.1217, found: 325.1227

Preparation of *tert*-butyl 3-(1-((4-fluorobenzyl)amino)cyclohexyl)propanoate (24f)

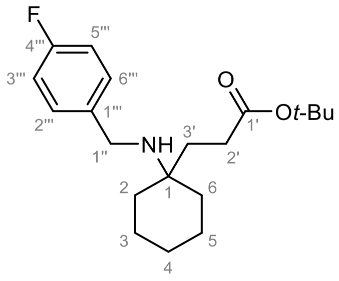

24f

Following the general procedure described above, 5.0 mL of a steady-state aliquot of crude γ-amino ester **22g** (0.40 mmol) was added dropwise to a stirred solution of 4-fluorobenzaldehyde (180 μL, 204 mg, 1.64 mmol, 4.1 equiv) in MeCN (1.0 mL) at 0 °C under an N_2 atmosphere. The reaction mixture was stirred at 0 °C for 15 min, then AcOH (300 μL, 5.25 mmol, 13 equiv) and sodium triacetoxyborohydride (458 mg, 2.16 mmol, 5.5 equiv) were added and the mixture was allowed to warm to rt over 18 h, with stirring. The resulting mixture was partitioned between CH_2Cl_2 (25 mL) and H_2O (25 mL). The layers were separated, and the aqueous phase was extracted with CH_2Cl_2 (2 × 25 mL). The combined organic layers were dried ($MgSO_4$), filtered, and concentrated *in vacuo*. Purification *via* flash column chromatography on silica gel (12 g) in CH_2Cl_2 (5 CV) then 100:0:0→90:9:1 CH_2Cl_2–MeOH–aq. NH_4OH (over 15 CV) then 90:9:1 CH_2Cl_2–MeOH–aq. NH_4OH (17 CV), followed by reversed-phase flash column chromatography on C_{18} silica gel (15.5 g) in H_2O (3 CV) then 100:0→0:100 H_2O–MeOH (over 15 CV) then MeOH (16 CV), gave **24f** as a brown oil (61.2 mg, 41%).

Data for **24f**:

^{1}H NMR: (500 MHz, $CDCl_3$)

7.36–7.30 (m, 2H, C(2‴)*H*, C(6‴)*H*), 7.02–6.96 (m, 2H, C(3‴)*H*, C(5‴)*H*), 3.57 (s, 2H, C(2‴)*H*$_2$), 2.32–2.24 (m, 2H, C(2′)*H*$_2$), 1.77–1.70 (m, 2H, C(3′)*H*$_2$), 1.56–1.29 (m, 20H, N*H*, C(O)(O*t-Bu*), C(2)*H*$_2$, C(3)*H*$_2$, C(4)*H*$_2$¸ C(5)*H*$_2$, C(6)*H*$_2$)

^{13}C NMR: (126 MHz, $CDCl_3$)

174.2 (*C*(1′)), 162 (d, *J* = 244 Hz, *C*(4‴)), 137.25 (*C*(4‴)), 129.9 (d, *J* = 7.9 Hz, *C*(2‴), *C*(6‴)), 115.2 (d, *J* = 21.1 Hz, *C*(3‴), *C*(5‴)), 80.2 (O*C*Me$_3$), 53.5 (*C*(1)), 44.6 (*C*(1″)), 35.5 (*C*(2), *C*(6)), 32.4 (*C*(3′)), 29.55 (*C*(2′)), 28.2 (OC*Me*$_3$), 26.2, (*C*(3), *C*(5)), 21.9 (*C*(4))

IR: (neat)

3783 (w), 2928 (m), 2853 (w), 2140 (w), 1725 (m), 1603 (w), 1509 (m), 1457 (w),

1392 (w), 1367 (m), 1298 (w), 1252 (w), 1220 (m), 1150 (m), 1015 (w), 958 (w), 918 (w), 845 (w), 825 (m), 755 (w), 733 (w), 666 (w)

MS: (ESI$^+$)

337 ([M+H]$^+$, 100%)

HRMS: (ESI$^+$)

calcd for $C_{20}H_{31}FNO_2$: 336.2333, found: 336.2343

G. Scale-up of α-C–H Alkylation in Flow

G.1. Gram-Scale Reaction on Vapourtec UV-150 Reactor

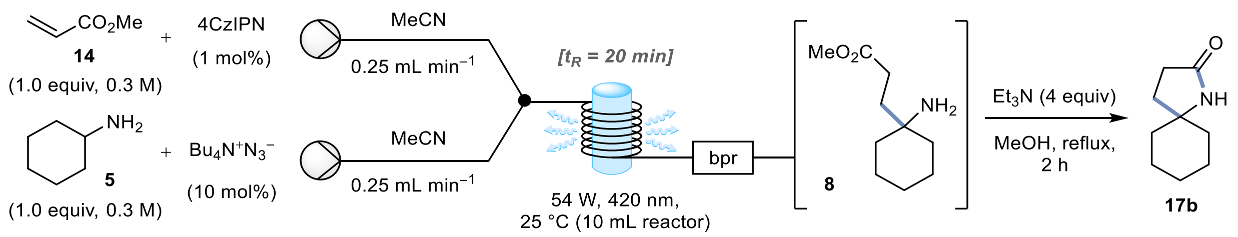

The following solutions were prepared in 50-mL volumetric flasks under N_2:

Reagent feed A: A 50-mL solution of methyl acrylate **14** (1.36 mL, 1.30 g, 15.0 mmol, 1.0 equiv) and 4CzIPN (118 mg, 0.15 mmol, 1 mol%) in dry MeCN.

Reagent feed B: A 50-mL solution of cyclohexylamine **5** (1.72 mL, 1.50 g, 15.0 mmol, 1.0 equiv) and tetrabutylammonium azide (427 mg, 1.50 mmol, 10 mol%) in dry MeCN.

A Vapourtec E-series flow reactor equipped with a UV-150 10-mL photoreactor and dry-ice cooling module was used. After priming the reagent lines for feeds A and B, and flushing the system with dry MeCN, 44-mL portions of feeds A and B were injected simultaneously into the photoreactor at a flow rate of 0.25 mL min^{-1} (residence time: 20 min), mixed in a T-mixer and passed through a 10 mL coil (0.8 mm inner diameter, fluoropolymer tube), irradiated with a 420 nm LED array (54 W radiant output power) at 25 °C. The pressure was kept around 1 bar by using the third pump as a back-pressure regulator (BPR). It was necessary to replenish the dry ice in the cooling module at intervals of ca. 30 min to ensure that reactor overheating did not occur, and the set point was temporarily increased to 30 °C during each re-filling process (to account for the brief temperature rise on removing the cooling module lid, and prevent the run terminating due to 'high' temperature). After the entire 88-mL mixture had entered the reactor, it was followed with dry MeCN at the same flow rate. The steady-state product mixture (from 25–196 min) was collected in a 250-mL, round-bottomed flask as a dark orange solution (85.5 mL), and was then concentrated *in vacuo*. Et_3N (7.36 mL, 5.34 g, 52.8 mmol) and MeOH (88 mL) were added, and the flask was fitted with a water-jacketed reflux condenser and heated at reflux in an oil bath for 2 h. After cooling to rt, the mixture was concentrated *in vacuo*. Purification *via* flash column chromatography on silica gel (80 g) in CH_2Cl_2 (3 CV) then 100:0:0→90:9:1 CH_2Cl_2–MeOH–aq. NH_4OH (over 11 CV) then 90:9:1 CH_2Cl_2–MeOH–aq. NH_4OH (10 CV) followed by reversed-phase flash column chromatography on C_{18} silica gel (20 g) in H_2O (3 CV) then 100:0→0:100 H_2O–MeOH (over 15

CV) then MeOH (16 CV) gave **17b** as a white crystalline solid (1.38 g, steady-state STY[20] = 0.32 mol L^{-1} h^{-1}).

Safety Note: It is known that inorganic azide reacts with CH_2Cl_2 to generate $CH_2(N_3)_2$, diazidomethane, and that this material presents a serious explosion risk when concentrated.[21] In the above procedure, we performed chromatographic purification using CH_2Cl_2 as an eluent, so there is the possibility of forming some $CH_2(N_3)_2$ during this step, by reaction of the tetrabutylammonium azide residues. The maximum theoretical yield would have been 74 mg of $CH_2(N_3)_2$ and, if formed at all, this would likely have eluted very early in the run. We did not experience any issues in our own laboratory but, for future reference, it may be advisable when working on gram-scale to replace CH_2Cl_2 with an alternative chromatography solvent.

G.2. Optimisation on Uniqsis PhotoSyn Reactor

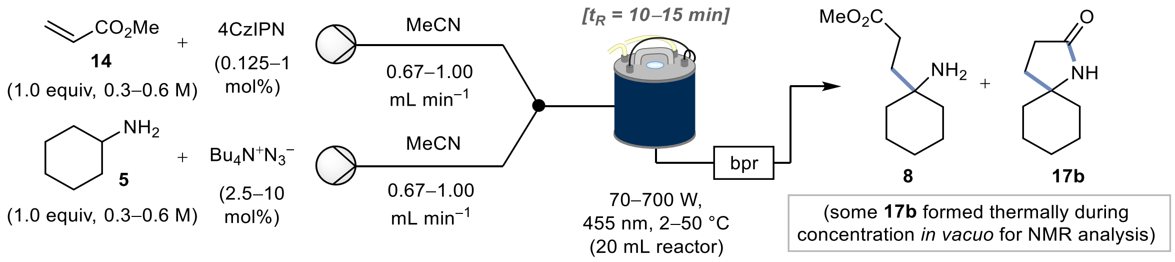

The following solutions were prepared in 100-mL volumetric flasks under N_2:

Reagent feed A: A 100-mL solution of methyl acrylate **14** (2.70 mL, 2.58 g, 30.0 mmol, 1.0 equiv) and 4CzIPN (237 mg, 0.30 mmol, 1 mol%) in dry MeCN. A suitable volume of this solution was then transferred to a 10-mL volumetric flask and additional **14** or 4CzIPN was added in order to adjust to the desired concentrations.

Reagent feed B: A 100-mL solution of cyclohexylamine **5** (3.43 mL, 2.98 g, 30.0 mmol, 1.0 equiv) and tetrabutylammonium azide (854 mg, 3.00 mmol, 10 mol%) in dry MeCN. A suitable volume of this solution was then transferred to a 10-mL volumetric flask and additional **5** or tetrabutylammonium azide was added in order to adjust to the desired concentrations.

A Vapourtec E-series flow reactor equipped with a Uniqsis cold coil tubing module and a PhotoSyn Blue HP LED photoreactor with a water-cooled 455 nm LED array (70–700 W radiant output power, with analog control) was used. After priming the reagent lines for feeds A and B, and flushing the system with dry MeCN, 5.0 mL portions of feeds A and B were injected

simultaneously into the photoreactor at various flow rates (residence time: 10–15 min), mixed in a T-mixer and passed through a 20 mL coil (1.0 mm inner diameter, fluoropolymer tube), irradiated with a 455 nm LED array (70–700 W radiant output power) at various temperatures (2–50 °C). The pressure was kept around 1 bar by using the third pump as a back-pressure regulator (BPR). After the entire 10-mL mixture had entered the reactor, it was followed with dry MeCN at the same flow rate. A 3.00-mL aliquot of the steady-state product mixture was collected and transferred to a round-bottomed flask, then concentrated *in vacuo* before subjection to ^{1}H NMR analysis. The combined NMR yield of **8** + **17b** (with the latter as a minor component) was calculated using the tetrabutylammonium ion as an internal standard, using the resonance at δ_H = 3.35 (8H, m) as a reference peak.

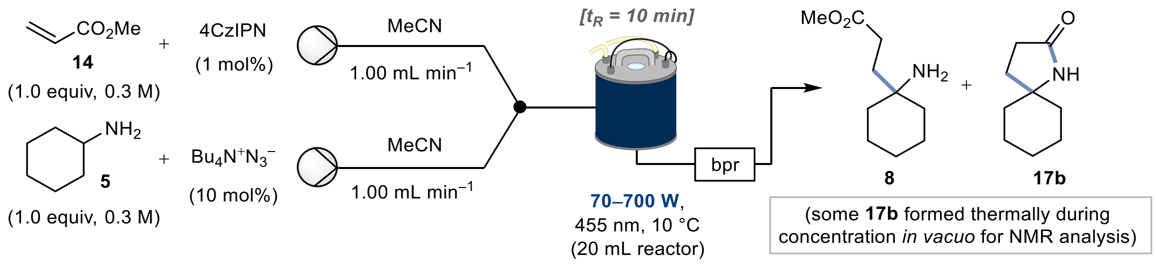

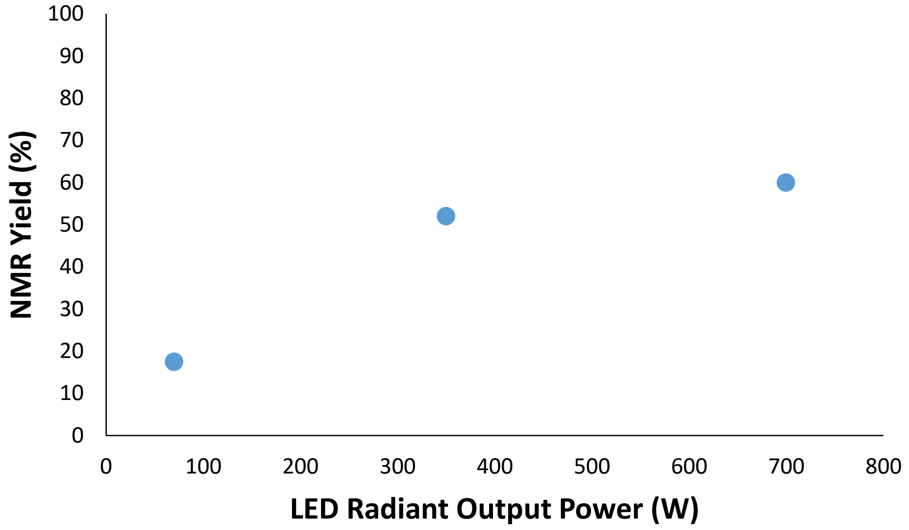

Fig. S11. NMR yield of **8** + **17b** as a function of LED radiant output power.

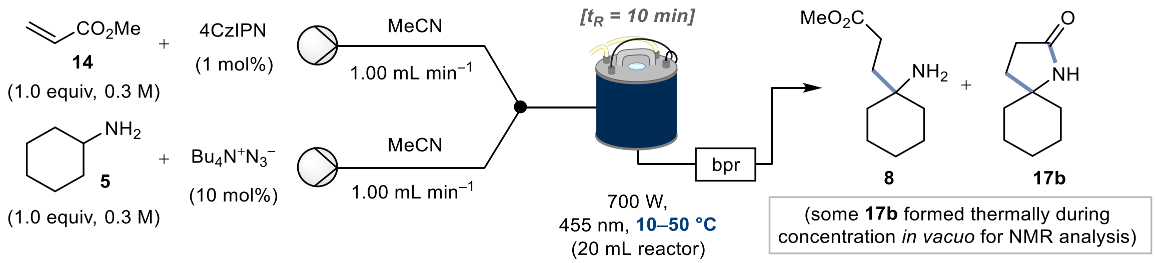

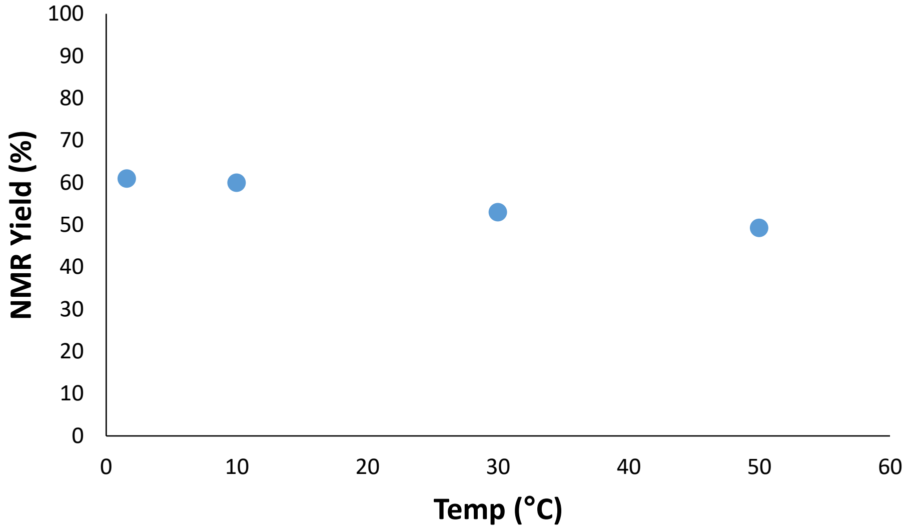

Fig. S12. NMR yield of **8** + **17b** as a function of reactor temperature.

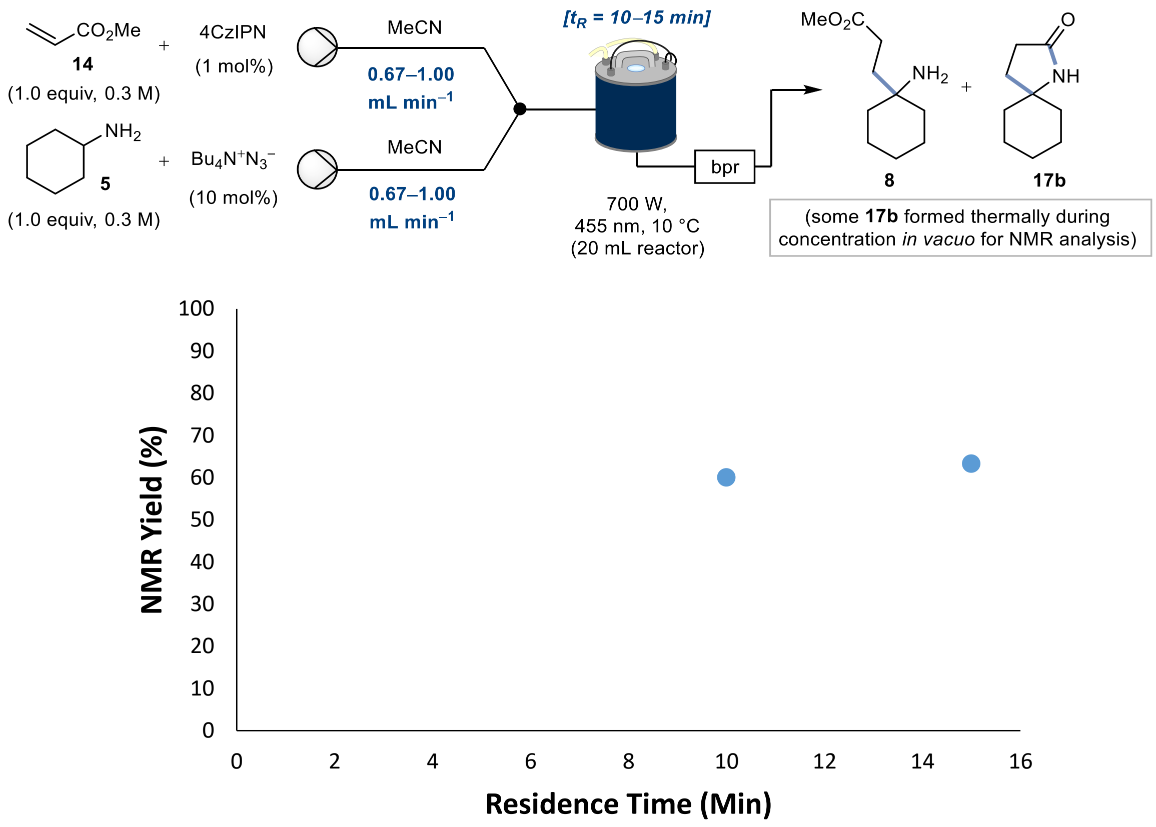

Fig. S13. NMR yield of **8** + **17b** as a function of residence time.

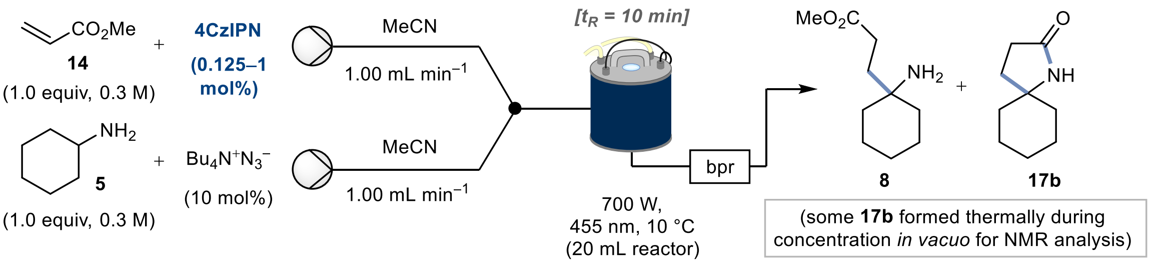

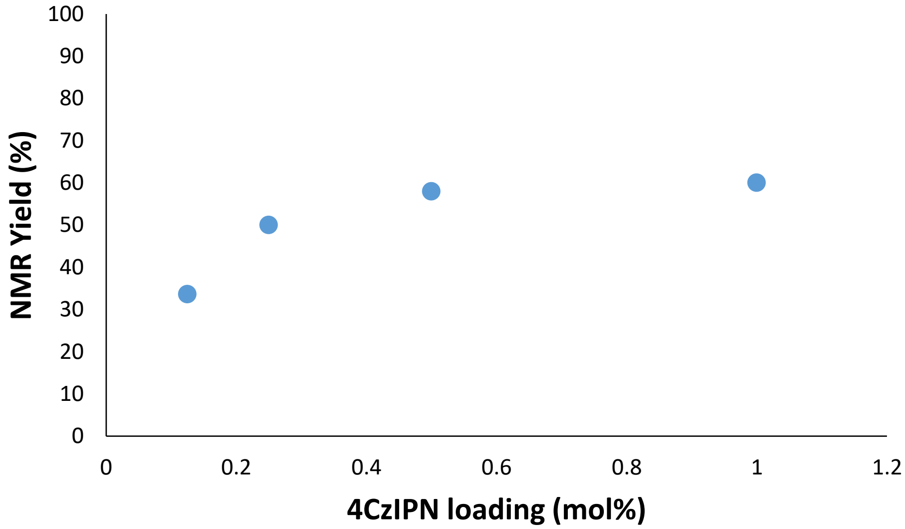

Fig. S14. **N**MR yield of **8** + **17b** as a function of 4CzIPN loading.

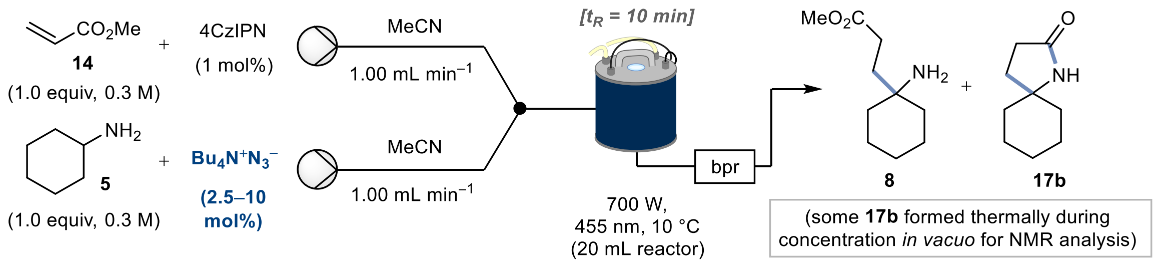

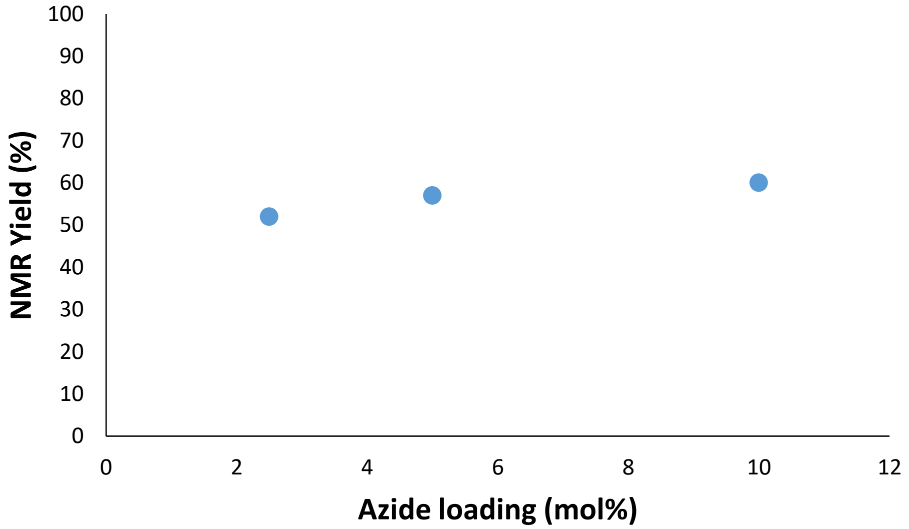

Fig. S15. NMR yield of **8** + **17b** as a function of tetrabutylammonium azide loading.

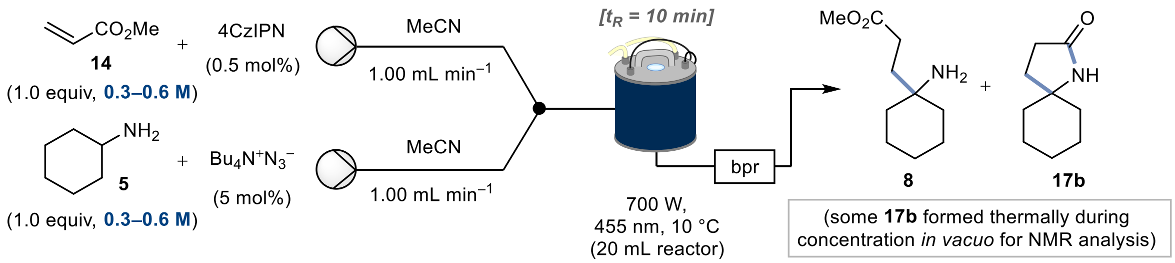

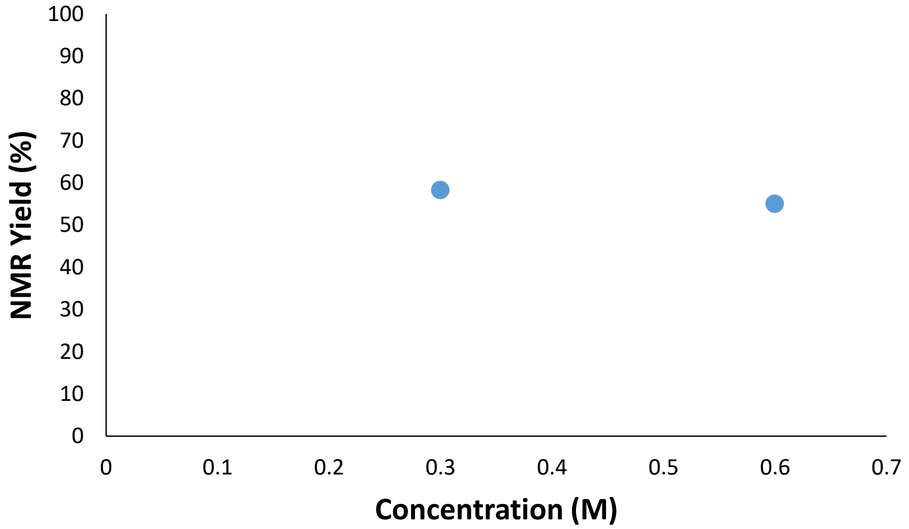

Fig. S16. NMR yield of **8** + **17b** as a function of reaction concentration (w.r.t. **5**).

Run	Residence time (min)	Power (W)	Temperature (°C)	4CzIPN catalyst (mol%)	Azide catalyst (mol%)	Effective Reaction Concentration (M, w.r.t. 5)	NMR Yield of 8 + 17b
1	10	700	10	1	10	0.15	60%
2	10	350	10	1	10	0.15	52%
3	10	70	10	1	10	0.15	18%
4	15	700	10	1	10	0.15	63%
5	10	700	30	1	10	0.15	53%
6	10	700	50	1	10	0.15	49%
7	10	700	10	0.5	10	0.15	58%
8	10	700	10	0.25	10	0.15	50%
9	10	700	10	0.125	10	0.15	34%
10	10	700	2	1	10	0.15	61%
11	10	700	10	1	5	0.15	57%
12	10	700	10	1	2.5	0.15	52%
13	10	700	10	0.5	5	0.15	58%
14	10	700	10	0.5	5	0.30	55%

Table S8. Summary of the above Optimisation experiments. Although the NMR yields of **8** + **17b** are all appreciably lower than that for **17b** under the optimised conditions on the Vapourtec UV-150 reactor (i.e., 90% NMR yield of **17b** with 54 W LED radiant output power at 420 nm), we reasoned that increasing the residence time to 20 min with the Uniqsis reactor would be counterproductive in terms of material throughput (STY). It should be noted that the Uniqsis reactor wavelength is centred on 455 nm (with a half-height bandwidth of 25 nm), and we already know that our reaction is appreciably slower at 450 nm than 425 nm (see section D.3).

G.3. Decagram-Scale Reaction on Uniqsis PhotoSyn Reactor

G.3.1. Preparation of Tetrabutylammonium Azide In Situ

Although tetrabutylammonium azide (CAS# 993-22-6) is commercially available, it is extremely hygroscopic and is difficult to handle on the open bench. To circumvent this issue, we sought to demonstrate that it can be prepared *in situ* without isolation.

Tetrabutylammonium chloride (2.92 g, 10.5 mmol, 1.0 equiv) and sodium azide (683 mg, 10.5 mmol, 1.0 equiv) – both weighed on the open bench – were added to a 500-mL round-bottomed flask containing a large stirrer bar. The flask was purged with N_2 for 10 min, then dry MeCN (100 mL) was added. The heterogeneous mixture was vigorously stirred for 18 h, then was cooled in an ice-water bath for 2 h to encourage complete precipitation of NaCl. The NaCl precipitate was removed by vacuum filtration through a sinter funnel packed with Celite (ca. 12 g, 1 cm thick layer). The NaCl precipitate was rinsed with chilled, dry MeCN (3 × 15 mL), and the filtrate was transferred to a 350-mL volumetric flask. Cyclohexylamine **5** (24.0 mL, 20.9 g, 210 mmol, 1.0 equiv) and additional MeCN (ca. 180 mL) were added to prepare the stock solution for reagent feed B (see below).

G.3.2. Performing a Decagram-Scale Reaction

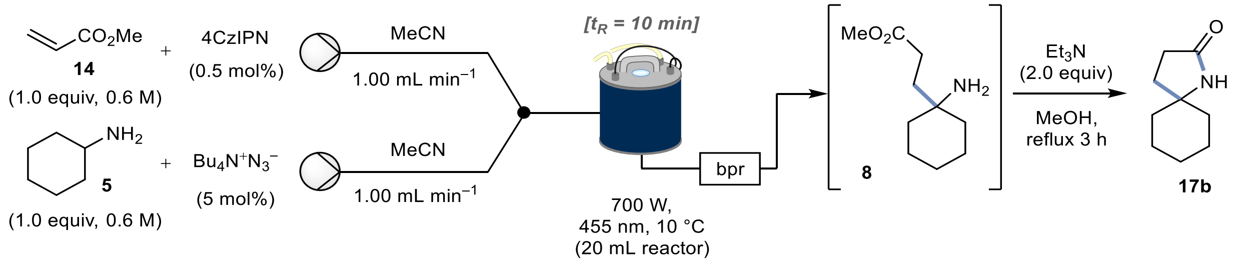

The following solutions were prepared under N_2:

Reagent feed A: A 350-mL solution of methyl acrylate **14** (18.9 mL, 18.1 g, 210 mmol, 1.0 equiv) and 4CzIPN (830 mg, 1.05 mmol, 0.5 mol%) in dry MeCN.

Reagent feed B: A 350-mL solution of cyclohexylamine **5** (24.0 mL, 20.9 g, 210 mmol, 1.0 equiv) and tetrabutylammonium azide (2.99 g, 10.5 mmol, 5 mol%) in dry MeCN.

A Vapourtec E-series flow reactor equipped with a Uniqsis cold coil tubing module and a PhotoSyn Blue HP LED photoreactor with a water-cooled 455 nm LED array (700 W radiant

output power) was used. After priming the reagent lines for feeds A and B, and flushing the system with dry MeCN, 320 mL portions of feeds A and B were injected simultaneously into the photoreactor at flow rates of 1.00 mL min^{-1} (residence time: 10 min), mixed in a T-mixer and passed through a 20 mL coil (1.0 mm inner diameter, fluoropolymer tube), irradiated with a 455 nm LED array (700 W radiant output power) at 10 °C. The pressure was kept around 1 bar by using the third pump as a back-pressure regulator (BPR). After the entire 640-mL mixture had entered the reactor, it was followed with dry MeCN at the same flow rate. The steady-state product mixture (collected over 5 h 17 min) was collected in a 1-L round-bottomed flask as a dark orange solution (634 mL), and was then concentrated *in vacuo* before subjection to ^{1}H NMR analysis. An NMR yield for **8** of 41% was calculated using the tetrabutylammonium ion as an internal standard, using the resonance at δ_H = 3.35 (8H, m) as a reference peak. Et_3N (52.9 mL, 38.4 g, 380 mmol, 2.0 equiv) and MeOH (250 mL) were added, and the flask was fitted with a water-jacketed reflux condenser and heated at reflux in an oil bath for 3 h. After cooling to rt, the mixture was concentrated *in vacuo*, followed by dilution with EtOAc (500 mL). The unreacted cyclohexylamine **5** was removed by washing with 2 M aq. HCl (500 mL), and the aqueous layer was back-extracted with EtOAc (3 × 100 mL). The combined organic layers were then washed with sat. aq. $NaHCO_3$ (500 mL), then the layers were separated and the organic phase was dried ($MgSO_4$), filtered and concentrated *in vacuo.* Purification by recrystallisation was performed by dissolution in hot MeCN (ca. 150 mL) and gravity filtration through filter paper (whilst still hot) to remove small quantities of insoluble material. The filtrate was left to cool to rt over 1 h, and was then cooled in a refrigerator (5 °C) overnight. Vacuum filtration, followed by rinsing with cold (–5 °C) MeCN and drying under high vacuum gave a first crop of **17b** as off-white crystals (2.43 g). The mother liquor was then concentrated *in vacuo* and a second recrystallisation was performed by dissolution in hot MeCN (ca. 100 mL), followed by cooling to rt and then in a refrigerator (5 °C) overnight. Vacuum filtration, as above, gave a second crop of **17b** as off-white crystals (2.10 g). The mother liquor was again concentrated *in vacuo* and the residue was dissolved in CH_2Cl_2 (50 mL) and concentrated *in vacuo* onto Celite (ca. 6 g). Purification *via* flash column chromatography on silica gel (120 g) in CH_2Cl_2 (3 CV) then 100:0:0→90:9:1 CH_2Cl_2–MeOH–aq. NH_4OH (over 11 CV) then 90:9:1 CH_2Cl_2–MeOH–aq. NH_4OH (10 CV) gave additional **17b** as an off-white solid (5.61 g). When combined with the two crops of recrystallised material, this led to an overall isolated yield of 10.1 g of **17b**.

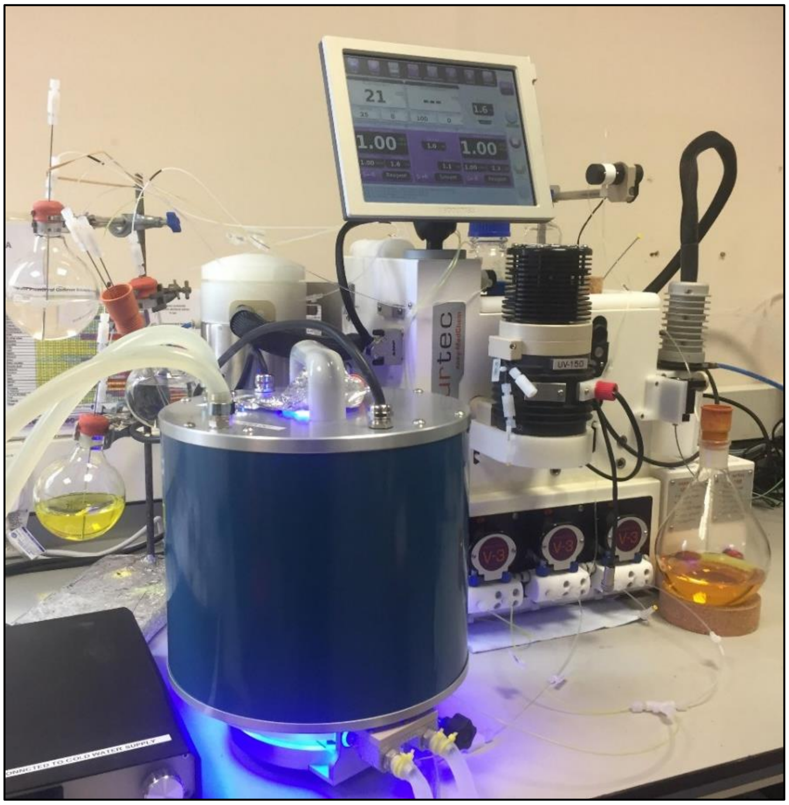

Fig. S17. Photograph of the set-up for flow photochemistry using the Uniqsis PhotoSyn reactor.

H. Mechanistic Studies

H.1. Reactivity of Cyclohexanol 18 towards α-C–H Alkylation

H.1.1. Absolute Reactivity of Cyclohexanol **18** *under α-C–H Alkylation Conditions*

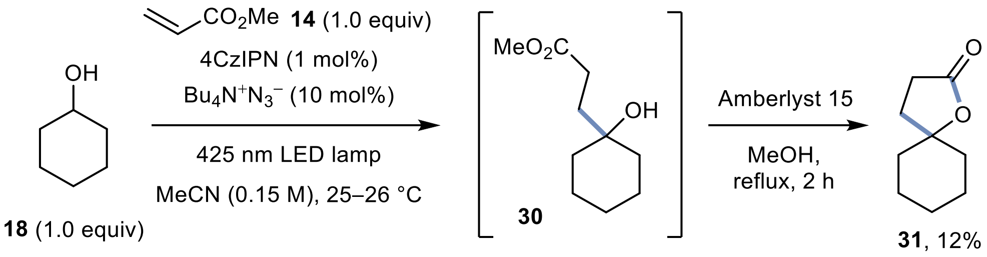

Cyclohexanol **18** (46.5 µL, 46 mg, 0.45 mmol, 1.0 equiv), methyl acrylate **14** (41.5 µL, 39 mg, 0.45 mmol, 1.0 equiv), 4CzIPN (2.28 mM in MeCN, 1.98 mL, 4.5 µmol, 1 mol%), tetrabutylammonium azide (70.3 mM in MeCN, 640 µL, 45 µmol, 10 mol%), and MeCN (390 µL) were reacted for 20 h with a 425 nm LED lamp. Following concentration *in vacuo*, the residue was dissolved in MeOH (5 mL) and Amberlyst®15 hydrogen form (118 mg) was added. The reaction was then heated at reflux for 2 h, followed by concentration *in vacuo*. Mesitylene (21.5 µL, 1.86 mg, 0.15 mmol, 0.33 equiv) was added and an NMR sample made up in $CDCl_3$. Quantitative ^{1}H NMR analysis showed that **31** had been generated in 12% yield (with the remaining mass balance being almost exclusively unreacted cyclohexanol **18**). The NMR spectroscopic data was in accordance with the literature.[22]

H.1.2. Relative Reactivity of Cyclohexanol **18** *and Cyclohexylamine* **5** *under α-C–H Alkylation Conditions*

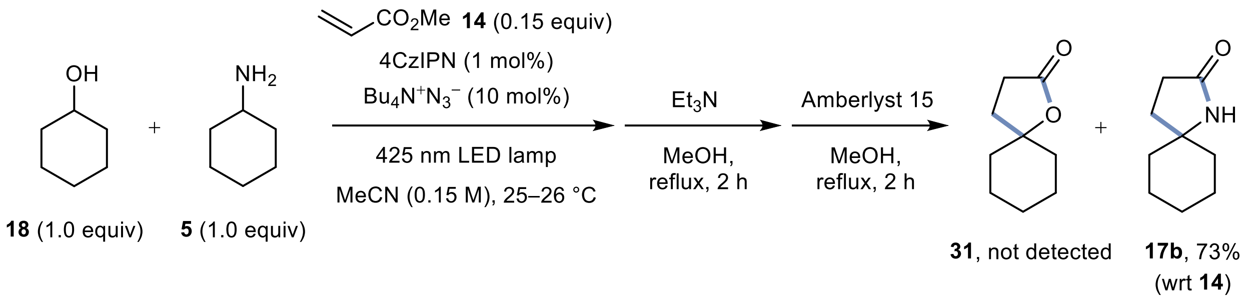

Cyclohexanol **18** (46.5 µL, 46 mg, 0.45 mmol, 1.0 equiv), cyclohexylamine **5** (52 µL, 45 mg, 0.45 mmol, 1.0 equiv), methyl acrylate **14** (6.0 µL, 5.9 mg, 0.068 mmol, 0.15 equiv), 4CzIPN (2.28 mM in MeCN, 1.98 mL, 4.5 µmol, 1 mol%), tetrabutylammonium azide (70.3 mM in MeCN, 640 µL, 45 µmol, 10 mol%), and MeCN (390 µL) were reacted for 20 h with a 425 nm LED lamp.

Following concentration *in vacuo*, the residue was dissolved in MeOH (5 mL) and Et_3N (0.25 mL, 0.18 g, 1.8 mmol, 4.0 equiv) was added. The reaction was then heated at reflux for 2 h, followed by concentration *in vacuo*. The residue was then dissolved in MeOH (5 mL) and Amberlyst®15 hydrogen form (118 mg) was added. The reaction was then heated at reflux for 2 h, followed by concentration *in vacuo*. Mesitylene (21.5 μL, 1.86 mg, 0.15 mmol, 0.33 equiv) was added and an NMR sample made up in $CDCl_3$. Quantitative ^{1}H NMR analysis showed that γ-lactam **17b** had been generated in 73% yield (wrt **14** as the limiting reactant), but no γ-lactone **31** was detectable. Based on the typical detection limits of ^{1}H NMR (i.e., detection of peaks with ~0.5% intensity), we estimated a lower limit for the relative reactivity ratio of **5**:**18** of 20:1.

H.2. Cyclic Voltammetry (CV) Measurements

Cyclic voltammetry analyses were performed using an IKA ElectraSyn. In a nitrogen purge box, tetrabutylammonium tetrafluoroborate (329 mg, 1.0 mmol) was measured into a 10 mL volumetric flask with either cyclohexylamine **5** (57 μL, 49.6 mg, 0.5 mmol) or tetrabutylammonium azide (142 mg, 0.5 mmol) and made up to the mark with MeCN.

The anodic/cathodic peak potentials were measured using a glassy carbon working electrode, a platinum plated counter electrode, and a 3 M KCl Ag/AgCl reference electrode using a 100 mV s^{-1} sweep rate for the cyclohexylamine **5** and a 500 mV s^{-1} sweep rate for tetrabutylammonium azide. The measurements are reported in graphs vs SCE, by subtraction of 30 mV from all voltages relative to the Ag|AgCl electrode.[23]

E_p were obtained from the graphs as the potential corresponding to the maximum current observed. To better evaluate compounds' redox potential, $E_{p/2}$ were used,[48] and these were measured as the potential corresponding to the half value of the maximum current intensity observed. Cyclic voltammetry of both cyclohexylamine **5** and tetrabutylammonium azide both show completely irreversible oxidation waves with E_p^A and $E_{p/2}$ (vs SCE) (Fig. S18).

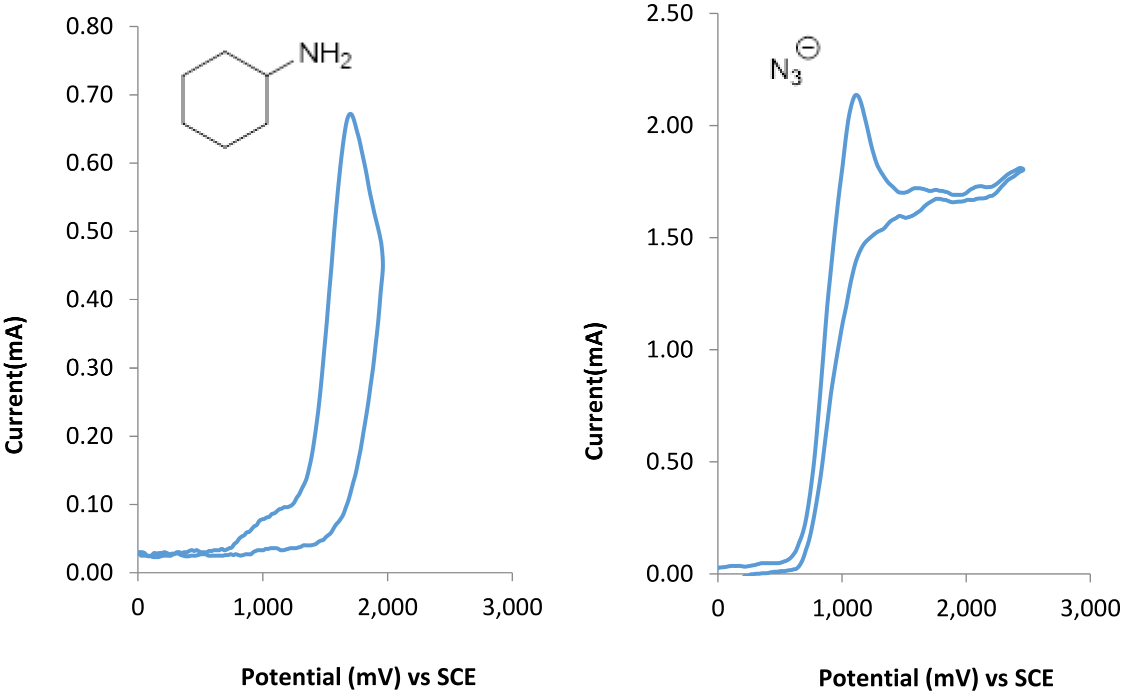

Fig. S18. Cyclic voltammograms of a 50 mM solution of cyclohexylamine **5** (left) and of a 50 mM solution of tetrabutylammonium azide (right).

	E_p^A (vs SCE)	$E_{p/2}$ (vs SCE)
Cyclohexylamine **5**	+1.69 V	+1.53 V
Tetrabutylammonium azide	+1.11 V	+0.87 V

Table S9. Redox potentials for cyclohexylamine **5** and tetrabutylammonium azide in MeCN.

H.3. Stern-Volmer Luminescence Quenching Analysis

In a nitrogen-filled purge box, stock solutions of 4CzIPN (0.02 mM in MeCN), tetrabutylammonium azide (0.70 mM in MeCN), and cyclohexylamine **5** (108 mM in MeCN) were prepared. Different solutions, with different concentration of quencher were prepared and analysed in a 10 × 10 mm quartz cuvette, equipped with a PTFE lid. The concentration of the 4CzIPN photocatalyst was maintained constant (0.045 μmol, 0.015 mM), varying only the quencher concentration. The emission spectra were recorded using an Agilent Cary Eclipse fluorimeter, equipped with a Xenon pulse lamp, pulse width of approximately 2–3 μs, peak power equivalent to 60–75 kW. The excitation wavelength was fixed at 425 nm while the emission light was acquired from 440 nm to 900 nm (emission light slit regulated to 5 nm). For quenching data the emission wavelength was fixed to 549 nm.

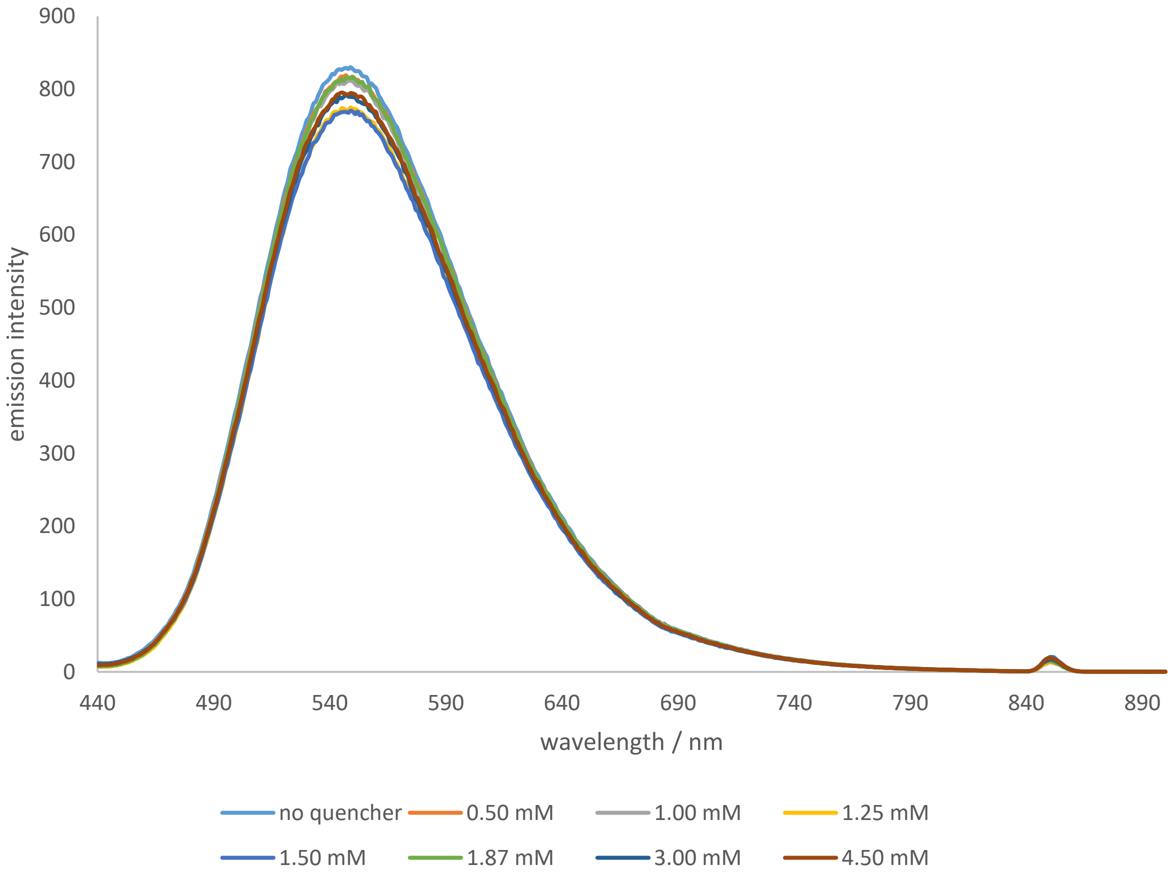

Fig. S19. Stacked emission spectra of 4CzIPN (0.015 mM in MeCN) at different concentrations of cyclohexylamine **5**.

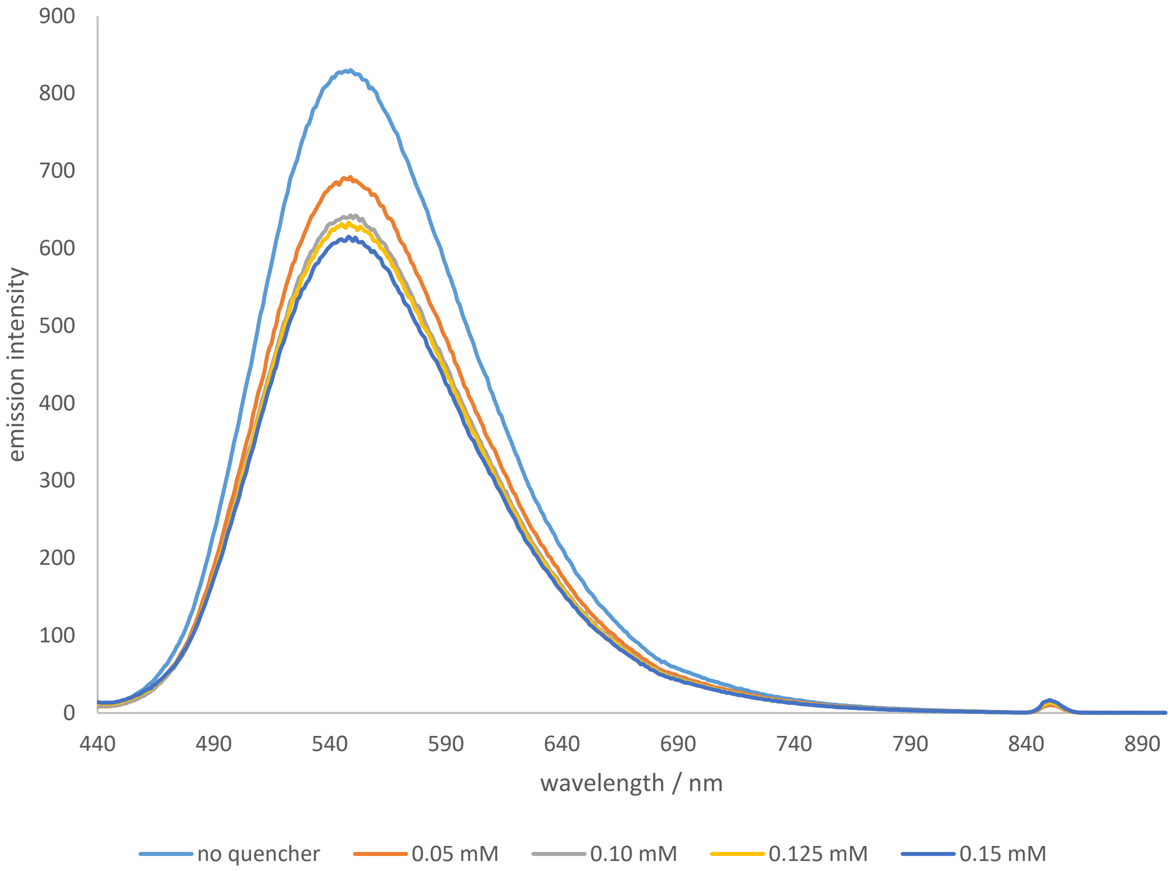

Fig. S20. Stacked emission spectra of 4CzIPN (0.015mM in MeCN) at different concentrations of tetrabutylammonium azide.

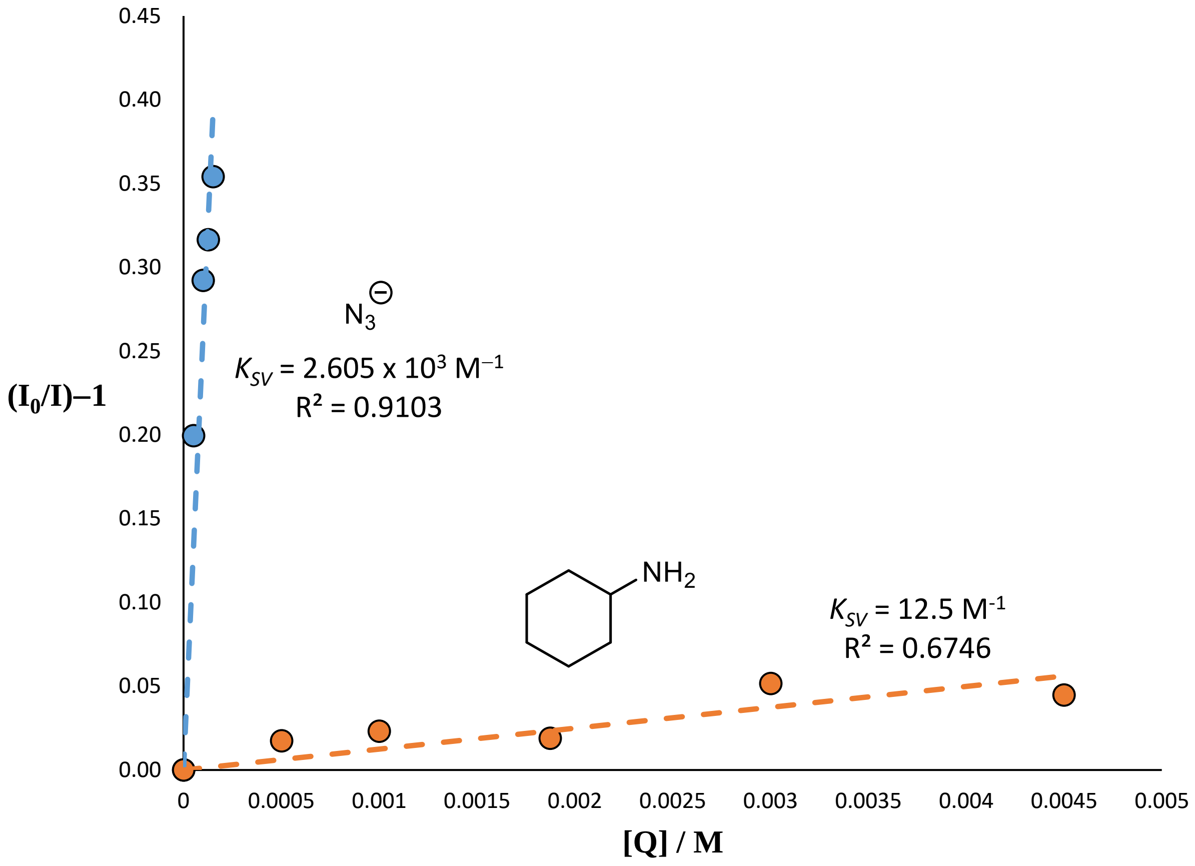

Fig. S21. Stern-Volmer plot for the quenching studies above. Emission wavelength fixed at 549 nm. Cyclohexylamine **5** is only weakly able to quench the fluorescence of 4CzIPN, whilst tetrabutylammonium azide is a much more effective quencher. The Stern-Volmer constant (K_{SV}) for the quenching of N_3^- is K_{SV} = 2.605 × 10^3 M^{-1}.

H.4. Quantum Yield Measurement

The quantum yield was measured for the reaction of cyclohexylamine **5** with methyl acrylate **14**. The reaction was performed in a 1-dram pressure relief reaction vial (15 × 45 mm, GPE) in an EvoluChem PhotoRedOx Box reactor with an EvoluChem 18 W LED lamp (λ_{max} = 425 nm).

H.4.1. Determination of the Photon Flux

The photon flux of the LED setup was determined using standard ferrioxalate actinometry.[24,25] A 0.15 M ferrioxalate solution was prepared by dissolving potassium ferrioxalate trihydrate (2.21 g) in 0.05 M aq. H_2SO_4 (30 mL). A buffered phenanthroline solution was prepared by dissolving 1,10-phenanthroline (50 mg) and NaOAc•3H_2O (11.25 g) in 0.5 M aq. H_2SO_4 (50 mL). Both solutions were stored in amber bottles in the dark.

Whilst working in a darkened fumehood, 1.0 mL of the 0.15 M ferrioxalate solution was added to a 1-dram pressure relief reaction vial (15 × 45 mm, GPE) positioned in an EvoluChem PhotoRedOx Box reactor equipped with a (switched-off) EvoluChem 18 W LED lamp (λ_{max} = 425 nm). The vial was then irradiated for 10 s, and 0.50 mL of the 1,10-phenanthroline solution was quickly added. This sequence was repeated for a second and third vial of ferrioxalate solution, irradiated for 20 and 30 s, respectively. A fourth vial of ferrioxalate solution was also prepared, to serve as a t = 0 s sample (no irradiation). Each mixture was wrapped in tin foil and left to stand for approximately 30 min before the absorbance at λ = 510 nm was measured by UV/Vis spectroscopy using a Perkin Elmer Lambda 25 UV/Vis Spectrophotometer.

The number of moles of Fe^{2+} formed was calculated using:

$$\text{mol Fe}^{2+} = \frac{V \times \Delta A}{l \times \varepsilon}$$

where V is the total volume of the solution after the addition of 1,10-phenanthroline (0.0015 L), ΔA is the difference in absorbance at λ = 510 nm between the irradiated and non-irradiated ferrioxalate solutions, l is the optical path length of the irradiation cell (1.0 cm), and ε is the molar absorptivity of the $Fe(phen)_3^{2+}$ complex at λ = 510 nm (11,100 L mol^{-1} cm^{-1}).

The moles of Fe^{2+} were plotted as a function of time (Fig. S22).

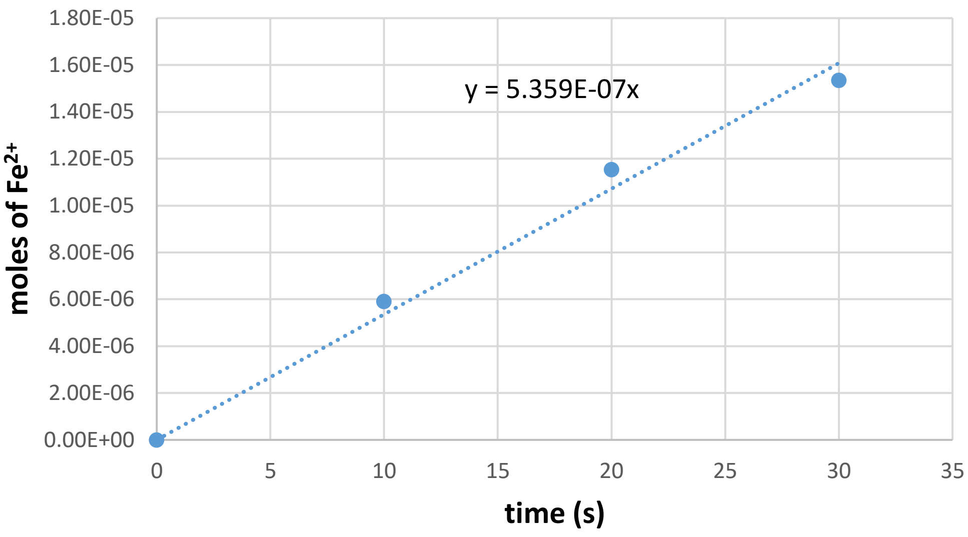

Fig. S22. The moles of Fe^{2+} produced in the ferrioxalate actinometry measurements, plotted as a function of time.

The photon flux was then calculated using:

$$\text{photon flux} = \frac{\text{mol Fe}^{2+}}{\Phi \times t \times f}$$

where Φ is the quantum yield of the ferrioxalate actinometer (1.1 at λ = 425 nm, by interpolation between 416 and 436 nm values),[49a] t is the time (s), and f is the fraction of absorbed light at λ = 425 nm,[26] where $f = 1 - 10^{-A}$. The absorbance (A) of the ferrioxalate solution at λ = 425 nm was measured by UV/Vis spectroscopy to be 1.174, so f = 0.9330.

$$\text{photon flux} = \frac{5.359 \times 10^{-7}}{1.1 \times 0.9330} = 5.22 \times 10^{-7}\ \text{mol s}^{-1}$$

This equates to 3.14×10^{17} photons s^{-1} being absorbed by the 1.0 mL solution (in this particular size and type of vial). According to $E = h\nu$ (by using an online calculator[27]) the energy of a single photon of wavelength λ = 425 nm is 4.67×10^{-19} J, so the photon power being absorbed by the solution can be calculated as 0.15 W.

H.4.2. Determination of the Quantum Yield

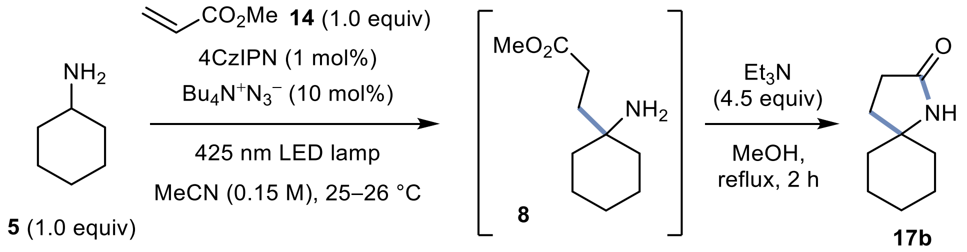

A 1-dram pressure relief reaction vial (15 × 45 mm, GPE) equipped with a stirrer bar was flame-dried and transferred to a nitrogen-filled purge box whilst still hot, then was allowed to cool under the N_2 atmosphere. The vial was then charged with stock solutions of 4CzIPN (2.28 mM in MeCN, 1.32 mL, 3.0 µmol, 1 mol%) and tetrabutylammonium azide (70.3 mM in MeCN, 430 µL, 30 µmol, 10 mol%), and made up to a total volume of 2.0 mL by addition of MeCN (260 µL). Cyclohexylamine **5** (35.0 µL, 30.3 mg, 0.30 mmol, 1.0 equiv) and methyl acrylate **14** (27.5 µL, 26.1 mg, 0.30 mmol, 1.0 equiv) were added, and the vial was sealed with a screw cap. It was then removed from the purge box and transferred to the same position in the same photoreactor used for the quantum yield measurement, and irradiated for 2 h at 425 nm (using the same lamp). Following irradiation, the reaction mixture was concentrated *in vacuo*. The residue was dissolved in MeOH (3 mL) and Et_3N (0.2 mL, 0.14 g, 1.35 mmol, 4.5 equiv) was added. The reaction was then heated at reflux for 2 h, followed by concentration *in vacuo*. The yield of **17b** was determined by ^{1}H NMR using tetrabutylammonium azide as an internal standard to be 45%. The reaction was repeated a second time, giving a yield of 50%. Average yield = 47%.

The quantum yield (Φ) was then calculated using:

$$\Phi = \frac{\text{mol product}}{\text{photon flux} \times t \ \times f}$$

where t is the time (7200 s) and f is the fraction of light absorbed by the 4CzIPN catalyst at λ = 425 nm, where $f = 1 - 10^{-A}$ (for a 1.5 × 10^{-3} M solution in MeCN, this was determined by UV/Vis spectroscopy to be 1.000).

$$\Phi = \frac{1.42 \times 10^{-4}}{5.22 \times 10^{-7} \times 7200 \ \times 1.000} = 0.04$$

H.5. Computational Details

Quantum mechanical calculations were performed using Gaussian 16 (Revision A.03).[28] All geometries were optimised using the M06-2X density functional and the 6-31G(d) basis set within the IEFPCM (acetonitrile) model. Single-point energies were calculated using M06-2X and the 6-311G(d,p) basis set within the IEFPCM (acetonitrile) model. The resulting energies were used to correct the energies obtained from the M06-2X Optimisations.[29] All temperature (298.15 K) and concentration-corrected (0.15 mol/l) quasiharmonic (Grimme approximation) free energies were calculated with GoodVibes[30] with a vibrational scaling factor of 1.0. Computed structures are illustrated with CYLView.[31]

Computational Data

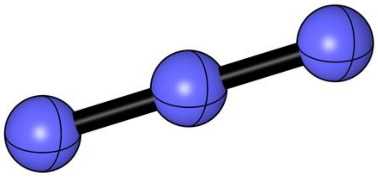

M06-2X/6-311G(d,p)-IEFPCM(acetonitrile) Energy	-164.110069
M06-2X/6-31G(d)-IEFPCM(acetonitrile) Energy	-164.066528
M06-2X/6-311G(d,p)-IEFPCM(acetonitrile) Derived Quasiharmonic Free Energy =	-164.119945
Imaginary Frequencies =	0

M06-2X/6-31G(d)-IEFPCM(acetonitrile) Geometry

N	1.160342	-0.001874	0.000000
N	0.000000	0.003721	0.000000
N	-1.160342	-0.001846	0.000000

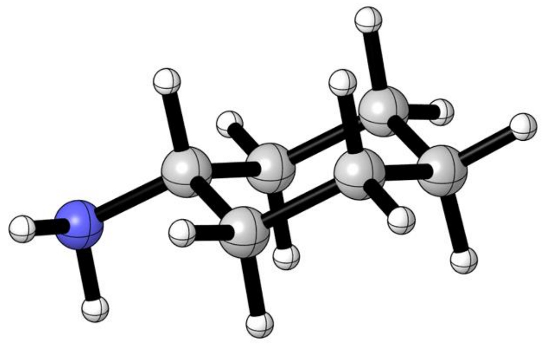

M06-2X/6-311G(d,p)-IEFPCM(acetonitrile) Energy	-291.165733
M06-2X/6-31G(d)-IEFPCM(acetonitrile) Energy	-291.081241
M06-2X/6-311G(d,p)-IEFPCM(acetonitrile) Derived Quasiharmonic Free Energy =	-291.004949
Imaginary Frequencies =	0

M06-2X/6-31G(d)-IEFPCM(acetonitrile) Geometry

C	1.153943	1.274174	-0.208432
C	-0.327091	1.245967	0.179191
C	-1.027382	-0.018467	-0.333940
C	-0.297209	-1.266550	0.162291
C	1.184870	-1.246513	-0.219665
C	1.873112	0.020367	0.294658
H	-0.419323	1.268330	1.275769
H	-0.844921	2.134233	-0.203854
H	1.238684	1.322190	-1.303112
H	1.632991	2.176778	0.186187
H	-0.389366	-1.308868	1.258628
H	-0.793494	-2.158273	-0.235468
H	1.686281	-2.139399	0.169199
H	1.273861	-1.283628	-1.314316
H	1.860628	0.015703	1.393567
H	2.924595	0.033928	-0.012578
H	-0.978564	-0.012646	-1.433140
N	-2.445929	-0.108417	0.030226
H	-2.512284	-0.081452	1.049027
H	-2.919044	0.738160	-0.286115

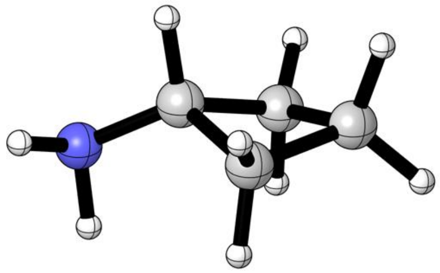

M06-2X/6-311G(d,p)-IEFPCM(acetonitrile) Energy	-212.518652
M06-2X/6-31G(d)-IEFPCM(acetonitrile) Energy	-212.454662
M06-2X/6-311G(d,p)-IEFPCM(acetonitrile) Derived Quasiharmonic Free Energy =	-212.415264
Imaginary Frequencies =	0

M06-2X/6-31G(d)-IEFPCM(acetonitrile) Geometry

C	0.524314	-0.005708	0.398108
C	-0.480728	-1.070489	-0.081380
C	-1.579024	0.015149	-0.018352
C	-0.462273	1.079049	-0.109499
H	0.551627	0.009083	1.493458
H	-0.598323	-1.979957	0.513150
H	-0.265428	-1.344859	-1.120677
H	-2.064386	0.031298	0.961474
H	-2.345571	0.010687	-0.795922
H	-0.566437	2.003283	0.465197
H	-0.240946	1.324834	-1.154539
N	1.886636	-0.115444	-0.090521
H	1.876082	-0.030720	-1.106787
H	2.433196	0.676444	0.245028

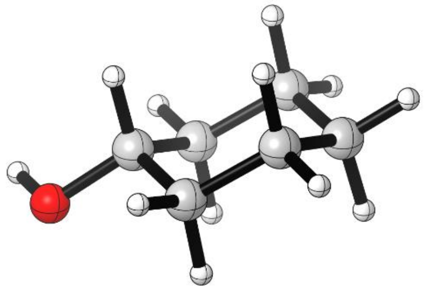

M06-2X/6-311G(d,p)-IEFPCM(acetonitrile) Energy	-311.038252
M06-2X/6-31G(d)-IEFPCM(acetonitrile) Energy	-310.944859
M06-2X/6-311G(d,p)-IEFPCM(acetonitrile) Derived Quasiharmonic Free Energy =	-310.890471
Imaginary Frequencies =	0

M06-2X/6-31G(d)-IEFPCM(acetonitrile) Geometry

C	1.165262	-1.248484	0.221847
C	-0.311728	-1.264478	-0.180357
C	-1.029659	-0.018179	0.319590
C	-0.341698	1.245962	-0.191885
C	1.134563	1.274134	0.213791
C	1.859904	0.019989	-0.281001
H	-0.400228	-1.289588	-1.275271
H	-0.816860	-2.155447	0.208264
H	1.242497	-1.290691	1.317025
H	1.668471	-2.140543	-0.165405
H	-0.430155	1.259084	-1.286874
H	-0.864439	2.132864	0.188383
H	1.615358	2.176772	-0.177335
H	1.207986	1.324204	1.308967
H	1.862932	0.016802	-1.379721
H	2.906516	0.033647	0.041787
H	-0.996617	-0.014340	1.422702
O	-2.377476	-0.100437	-0.124347
H	-2.835526	0.697067	0.180353

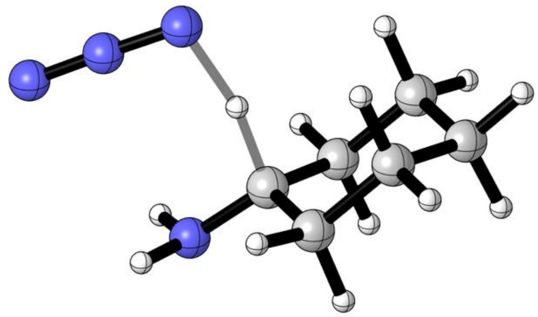

M06-2X/6-311G(d,p)-IEFPCM(acetonitrile) Energy	-455.279371
M06-2X/6-31G(d)-IEFPCM(acetonitrile) Energy	-455.149628
M06-2X/6-311G(d,p)-IEFPCM(acetonitrile) Derived Quasiharmonic Free Energy =	-455.117427
Imaginary Frequencies =	-328.12

M06-2X/6-31G(d)-IEFPCM(acetonitrile) Geometry

C	1.772588	-0.536593	-1.263358
C	0.825089	0.665340	-1.278359
C	0.002962	0.723849	0.000979
C	0.824488	0.661040	1.280497
C	1.772030	-0.540812	1.261912
C	2.637898	-0.540593	-0.000536
H	1.403204	1.597962	-1.352192
H	0.156427	0.626156	-2.146373
H	1.181979	-1.461461	-1.302014
H	2.400266	-0.519767	-2.159668
H	1.402532	1.593424	1.357791
H	0.155376	0.618894	2.148029
H	2.399307	-0.526948	2.158553
H	1.181431	-1.465816	1.297238
H	3.278888	0.351790	0.001094
H	3.301603	-1.411366	-0.001844
H	-0.595937	-0.397092	-0.000969
N	-0.974707	1.716546	0.002452
H	-1.517675	1.843290	-0.844695
H	-1.517932	1.840483	0.849849

N	-1.530900	-1.442998	-0.002199
N	-2.562837	-0.823191	-0.001370
N	-3.537242	-0.205062	-0.000541

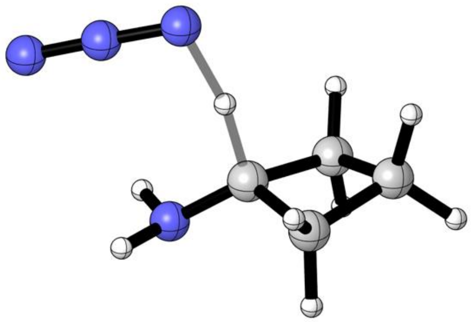

M06-2X/6-311G(d,p)-IEFPCM(acetonitrile) Energy	-376.628894
M06-2X/6-31G(d)-IEFPCM(acetonitrile) Energy	-376.519498
M06-2X/6-311G(d,p)-IEFPCM(acetonitrile) Derived Quasiharmonic Free Energy =	-376.525990
Imaginary Frequencies =	-483.40

M06-2X/6-31G(d)-IEFPCM(acetonitrile) Geometry

C	-0.591790	0.415224	-0.000028
C	-1.590552	0.024732	1.093181
C	-1.590732	0.024842	-1.093112
H	0.261530	-0.543095	-0.000133
H	-2.136649	0.905474	1.446145
H	-1.208559	-0.536581	1.948740
H	-2.136887	0.905621	-1.445894
H	-1.208884	-0.536382	-1.948794
N	0.087321	1.621550	-0.000017
H	0.578550	1.882574	0.848126
H	0.578440	1.882654	-0.848200
N	1.374533	-1.341953	-0.000160
N	2.259358	-0.523216	-0.000027
N	3.090495	0.274972	0.000100
C	-2.367432	-0.752419	0.000059
H	-2.114423	-1.814976	-0.000015
H	-3.452027	-0.639034	0.000154

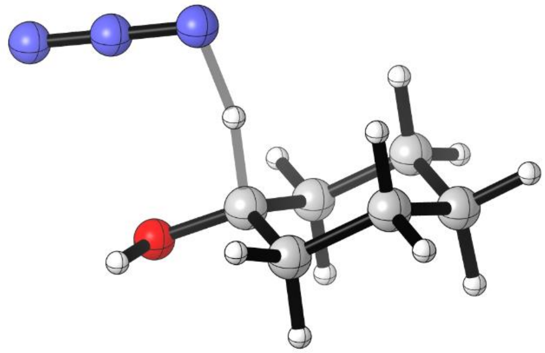

M06-2X/6-311G(d,p)-IEFPCM(acetonitrile) Energy	-475.142855
M06-2X/6-31G(d)-IEFPCM(acetonitrile) Energy	-475.004998
M06-2X/6-311G(d,p)-IEFPCM(acetonitrile) Derived Quasiharmonic Free Energy =	-474.994601
Imaginary Frequencies =	-938.94

M06-2X/6-31G(d)-IEFPCM(acetonitrile) Geometry

C	1.840715	0.108493	-1.336198
C	0.864393	1.174129	-0.829214
C	0.007339	0.625971	0.293127
C	0.756072	-0.042602	1.424119
C	1.733148	-1.090243	0.883653
C	2.651679	-0.489510	-0.183552
H	1.419055	2.038228	-0.432726
H	0.224469	1.539225	-1.641805
H	1.274105	-0.689295	-1.834353
H	2.502580	0.547959	-2.088199
H	1.303583	0.741558	1.968323
H	0.036709	-0.483482	2.122100
H	2.319678	-1.503793	1.709425
H	1.164005	-1.921711	0.447062
H	3.269416	0.297035	0.270863
H	3.335726	-1.254361	-0.564729
H	-0.625516	-0.350533	-0.252729
N	-1.568212	-1.210855	-0.683162
N	-2.601859	-0.676727	-0.346814
N	-3.586559	-0.175986	-0.033407

O	-0.973495	1.462468	0.770449
H	-1.259520	2.066967	0.065247

I. ^{1}H and $^{13}C\{^{1}H\}$ NMR Spectra

^{1}H NMR (500 MHz, $CDCl_3$)

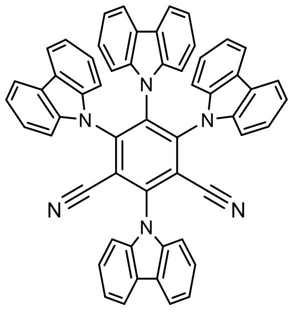

4CzIPN

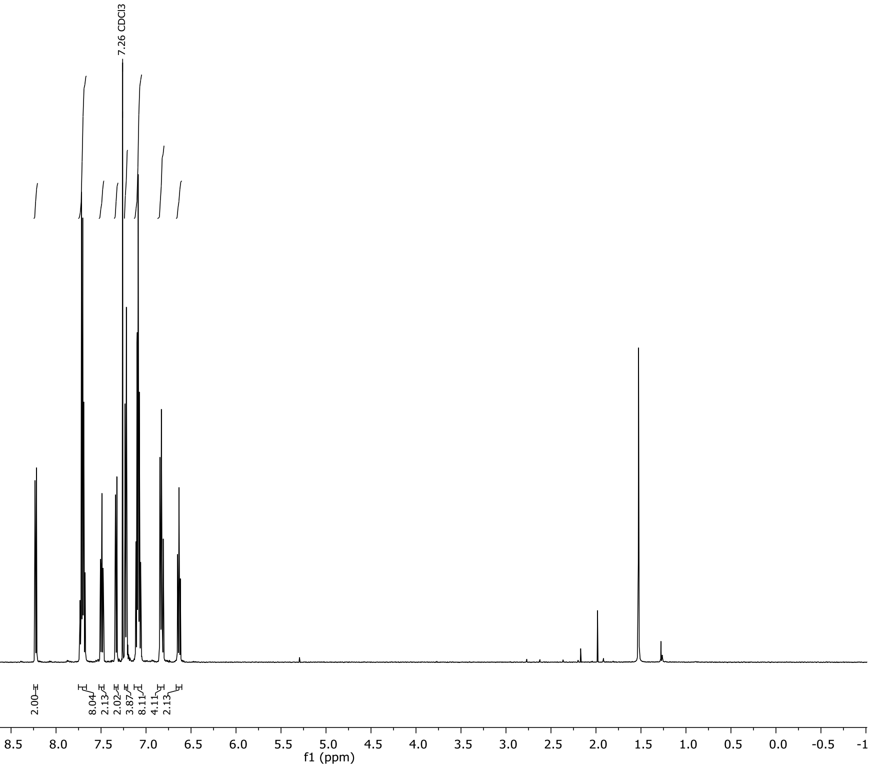

$^{13}C\{^{1}H\}$ NMR (126 MHz, $CDCl_3$)

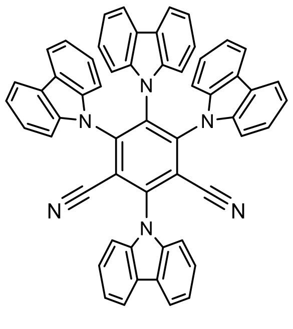

4CzIPN

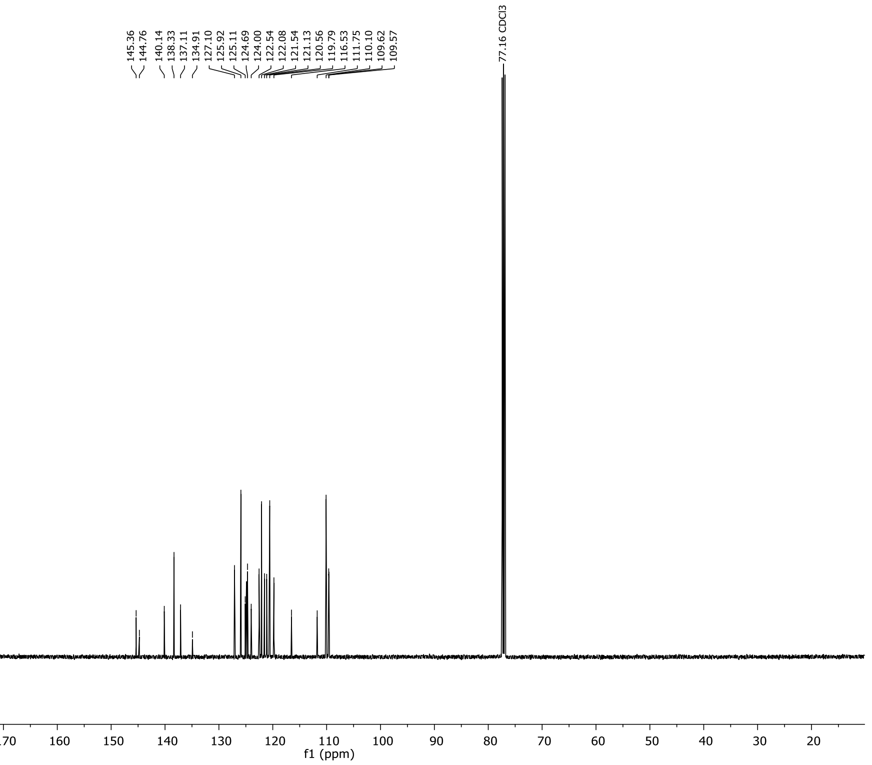

^{1}H NMR (500 MHz, DMSO-*d*$_6$)

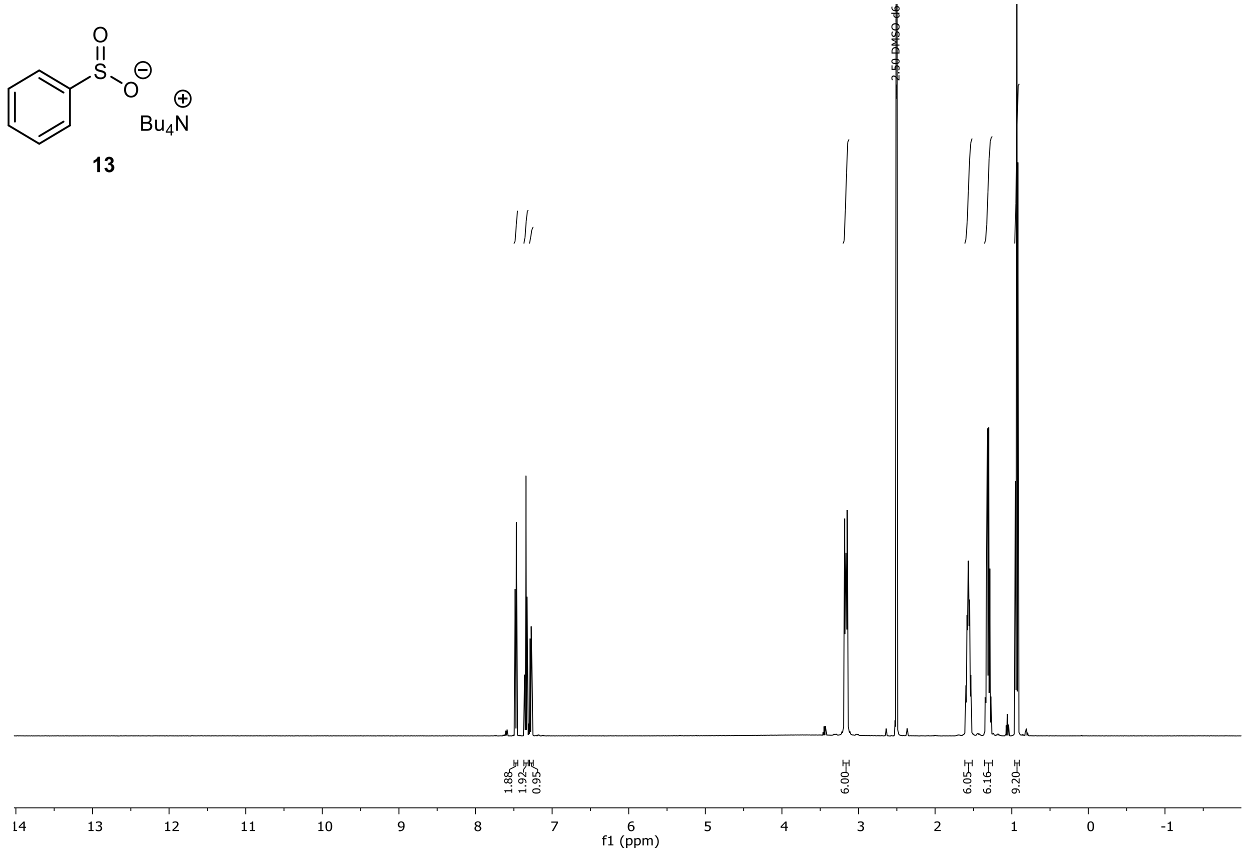

^{13}C{^{1}H} NMR (126 MHz, DMSO-*d*$_6$)

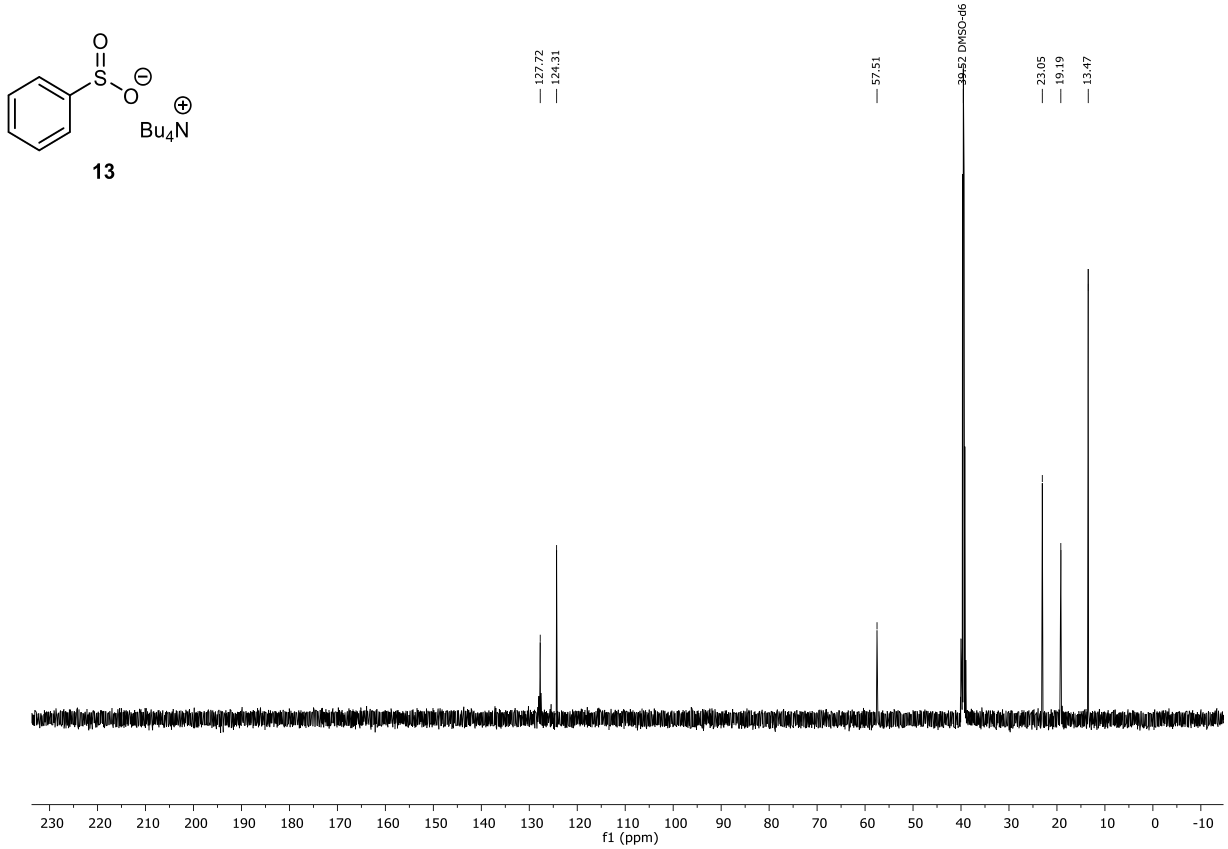

^{1}H NMR (500 MHz, $CDCl_3$)

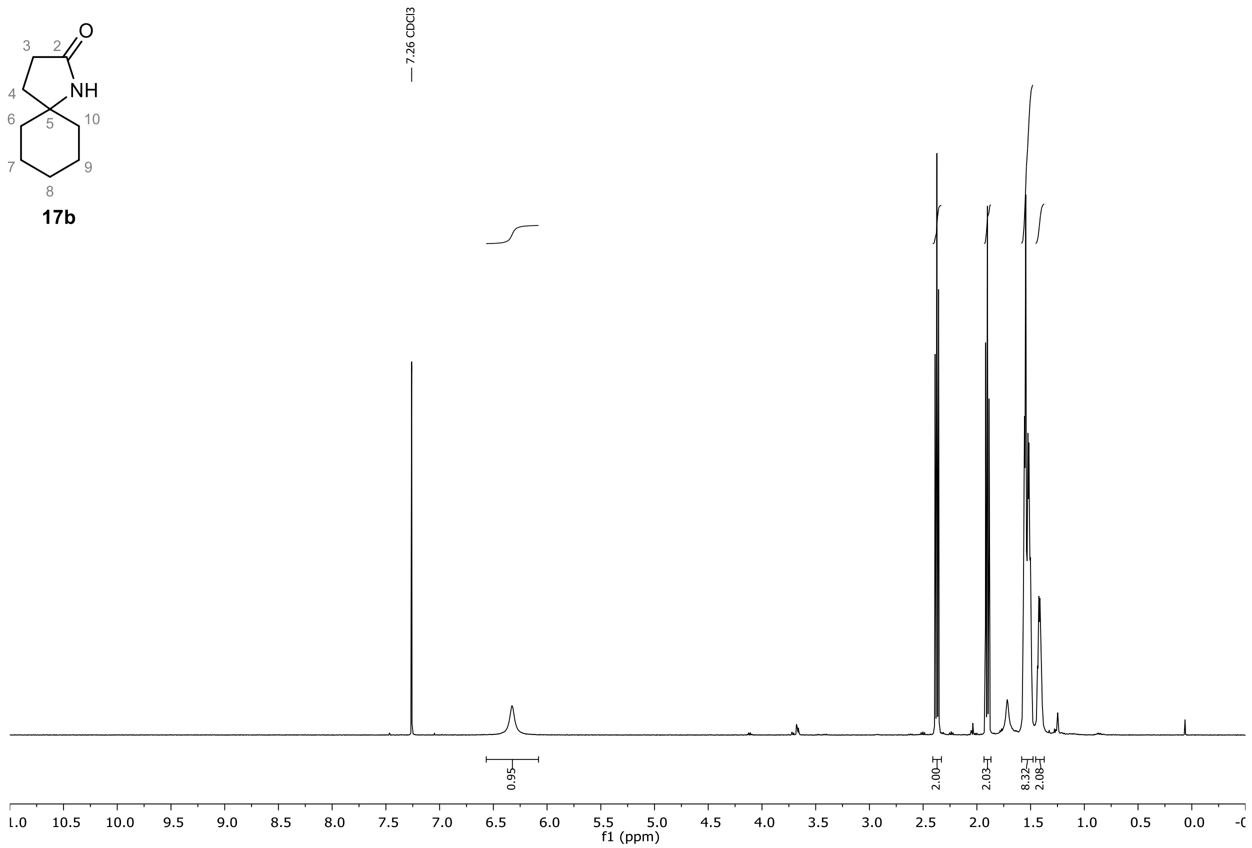

^{13}C{^{1}H} NMR (126 MHz, $CDCl_3$)

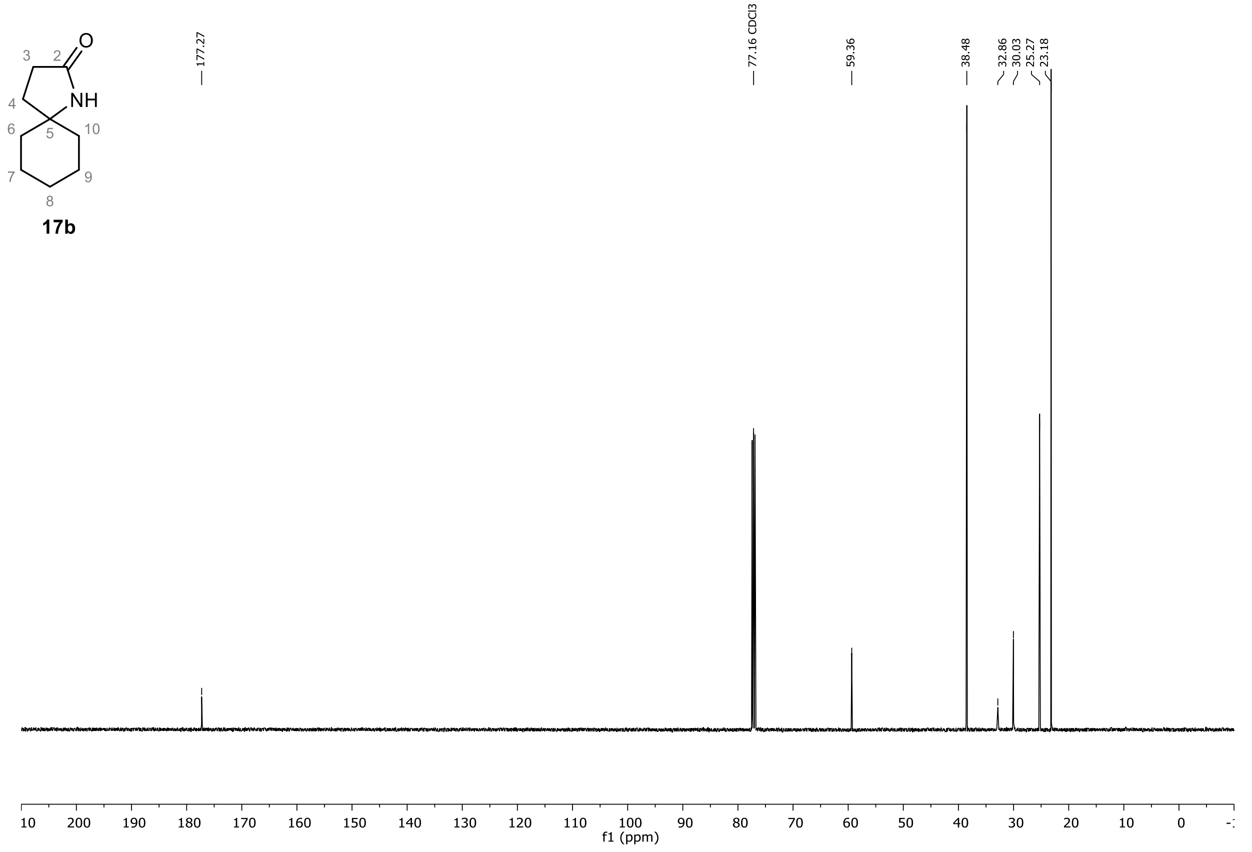

^{1}H NMR (500 MHz, $CDCl_3$)

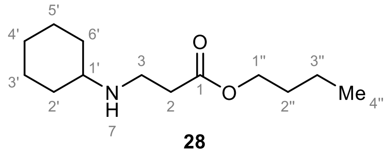

28

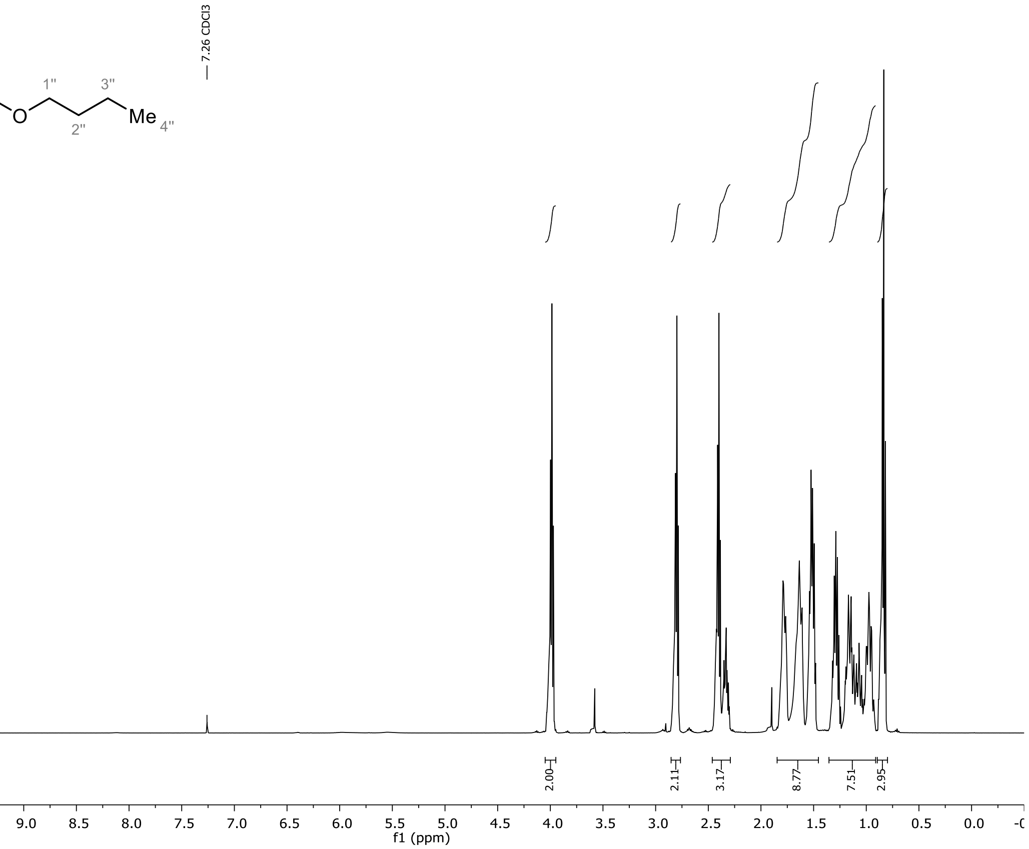

^{13}C{^{1}H} NMR (126 MHz, $CDCl_3$)

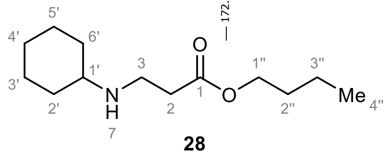

28

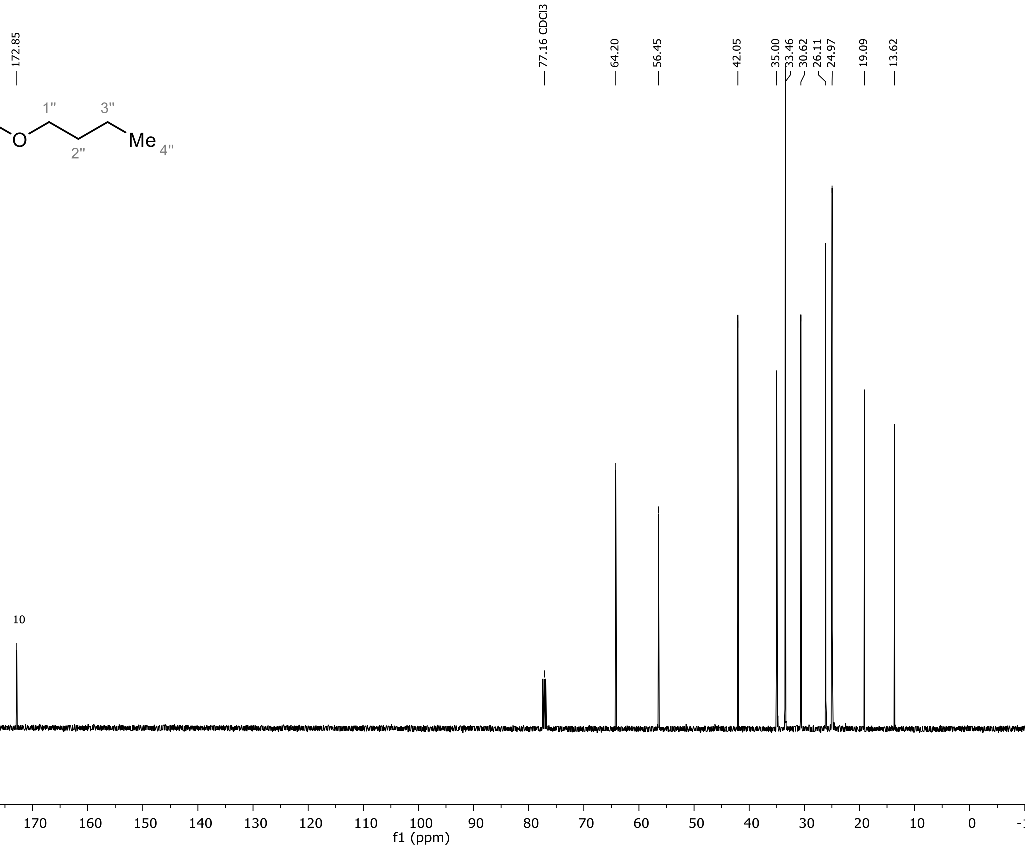

^{1}H NMR (500 MHz, $CDCl_3$)

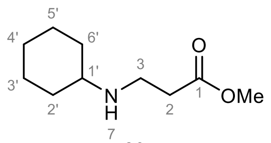

29

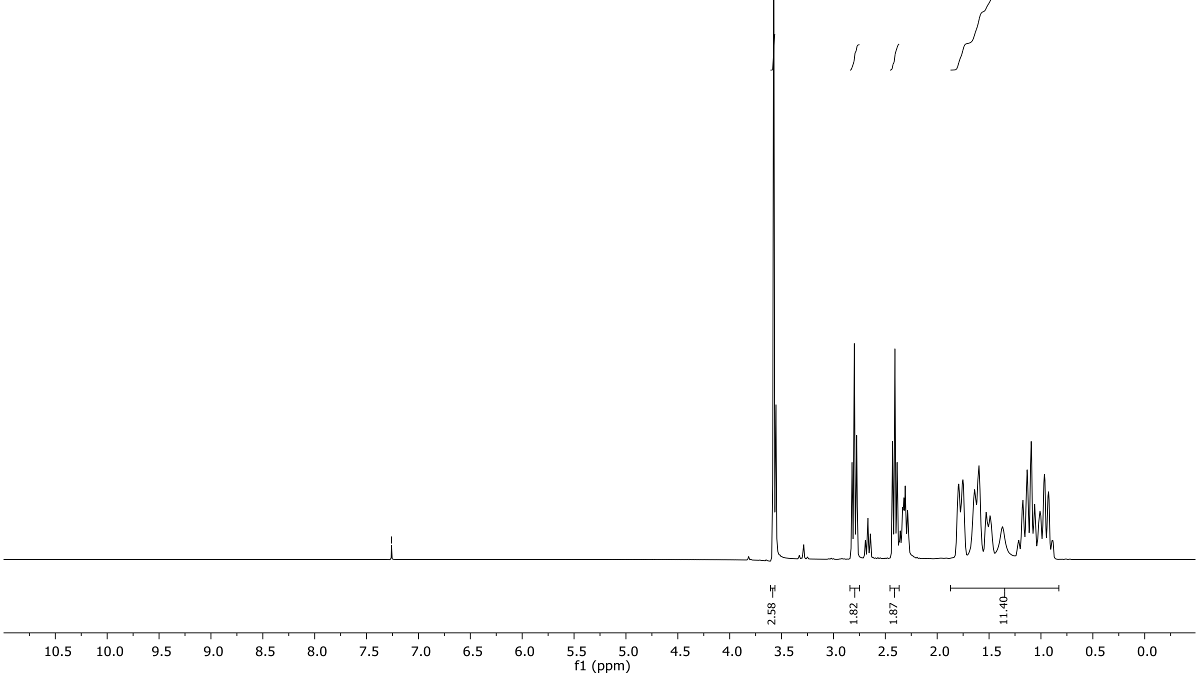

^{13}C{^{1}H} NMR (126 MHz, $CDCl_3$)

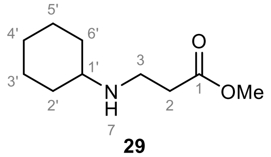

29

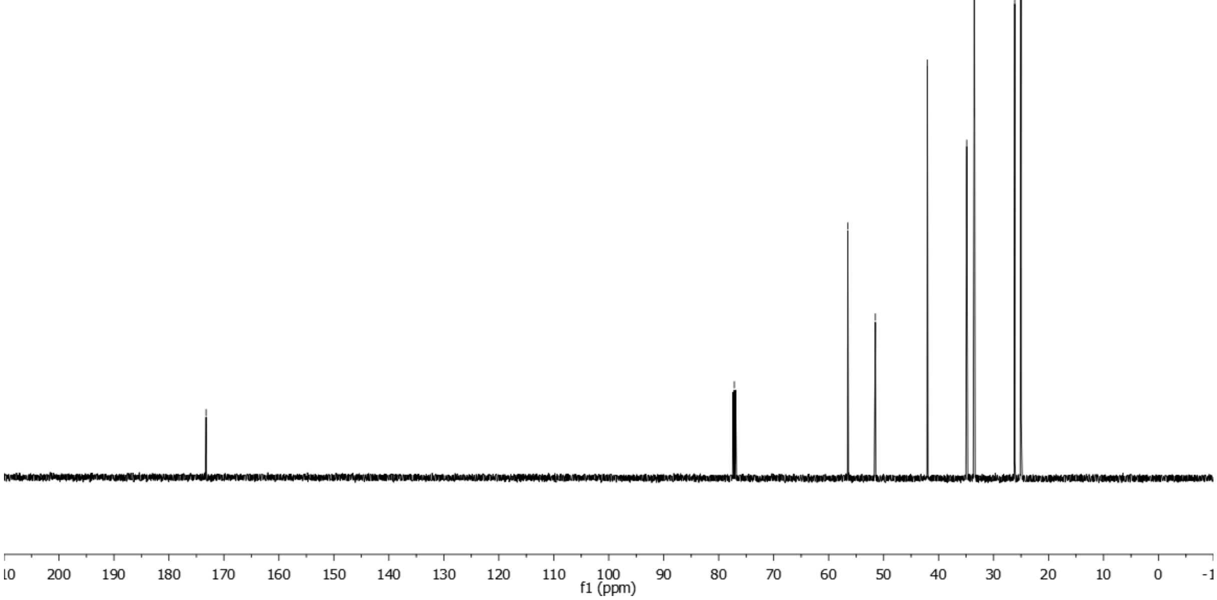

^{1}H NMR (500 MHz, $CDCl_3$)

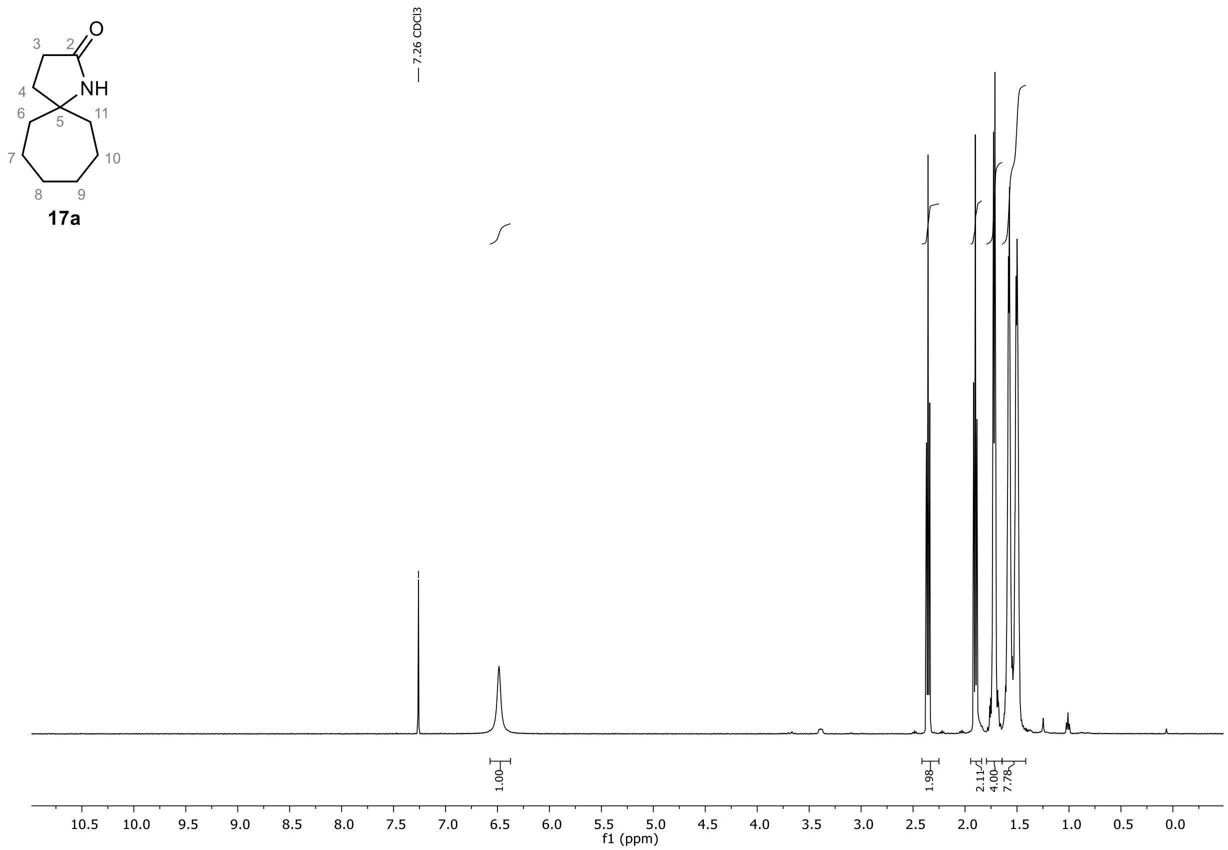

^{13}C{^{1}H} NMR (126 MHz, $CDCl_3$)

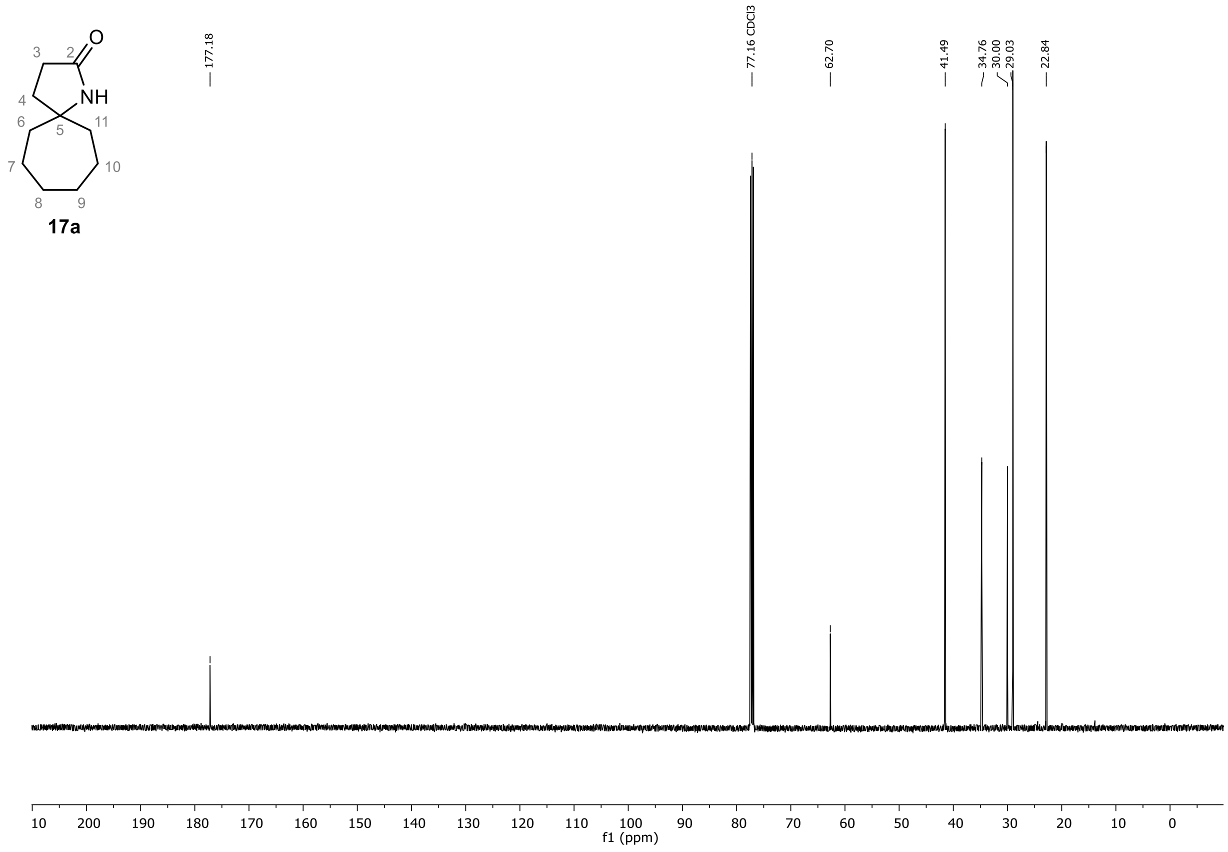

^{1}H NMR (500 MHz, $CDCl_3$)

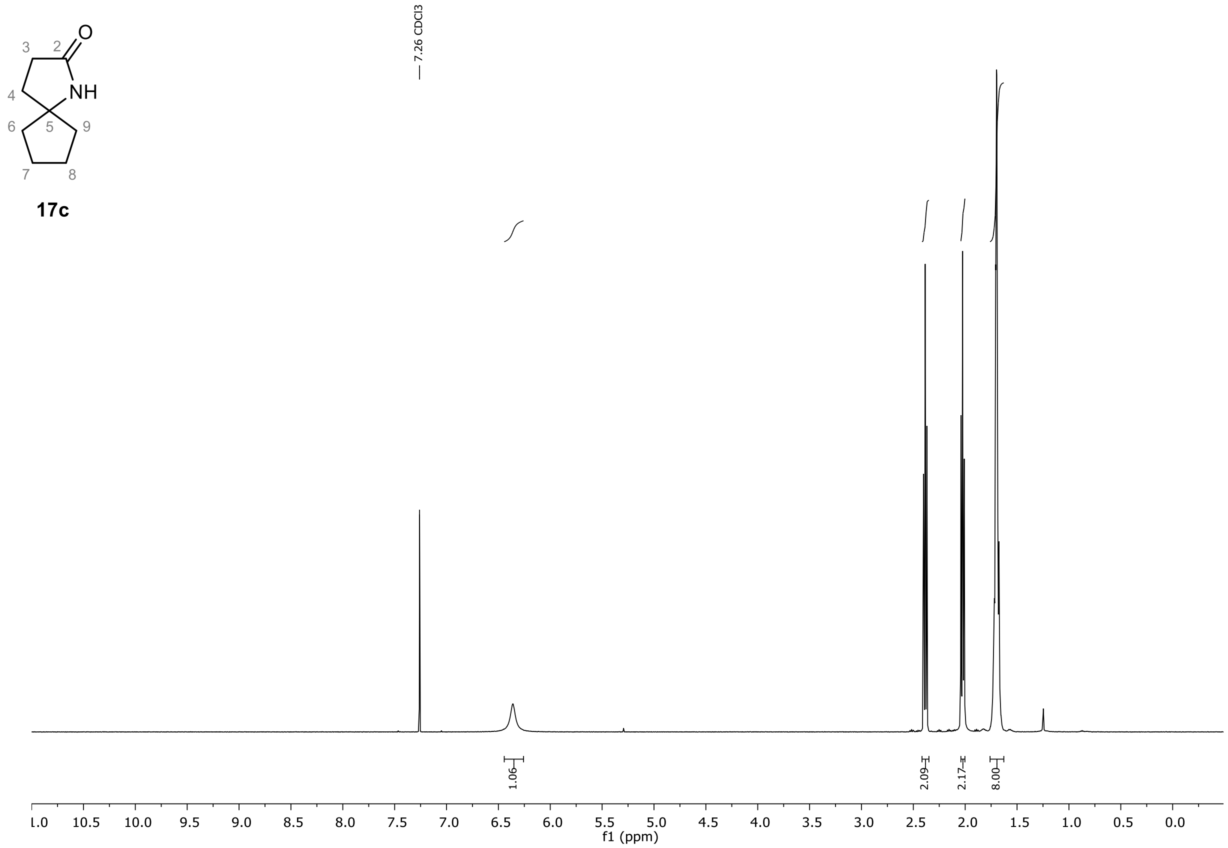

^{13}C{^{1}H} NMR (126 MHz, $CDCl_3$)

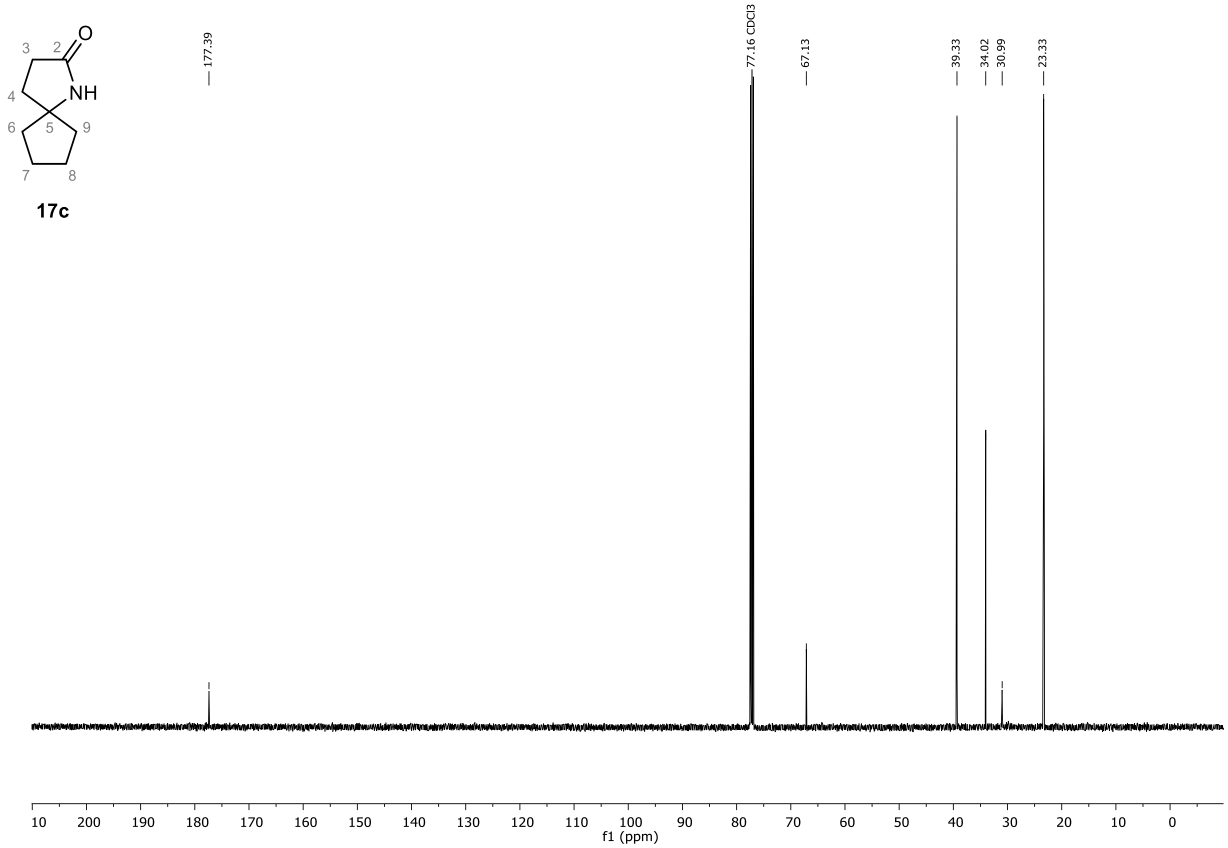

^{1}H NMR (500 MHz, $CDCl_3$)

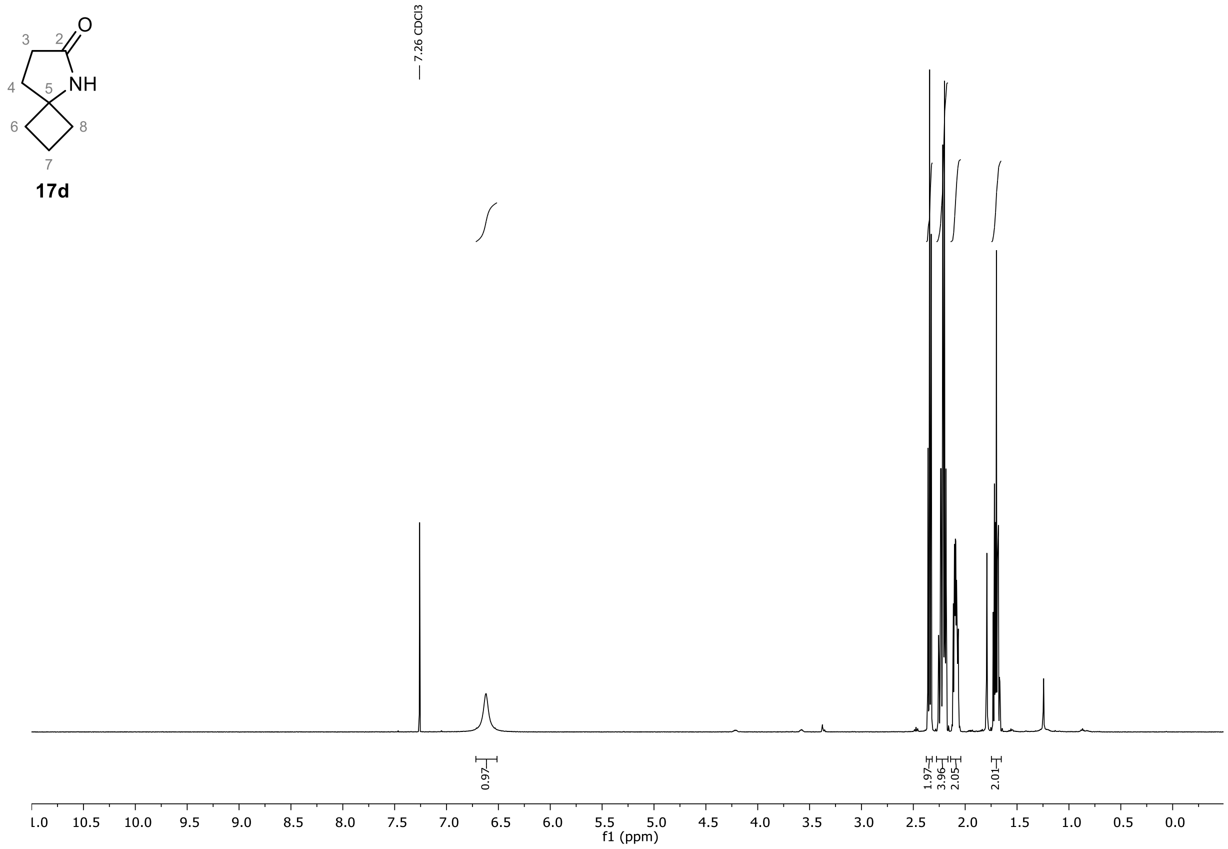

$^{13}C\{^{1}H\}$ NMR (126 MHz, $CDCl_3$)

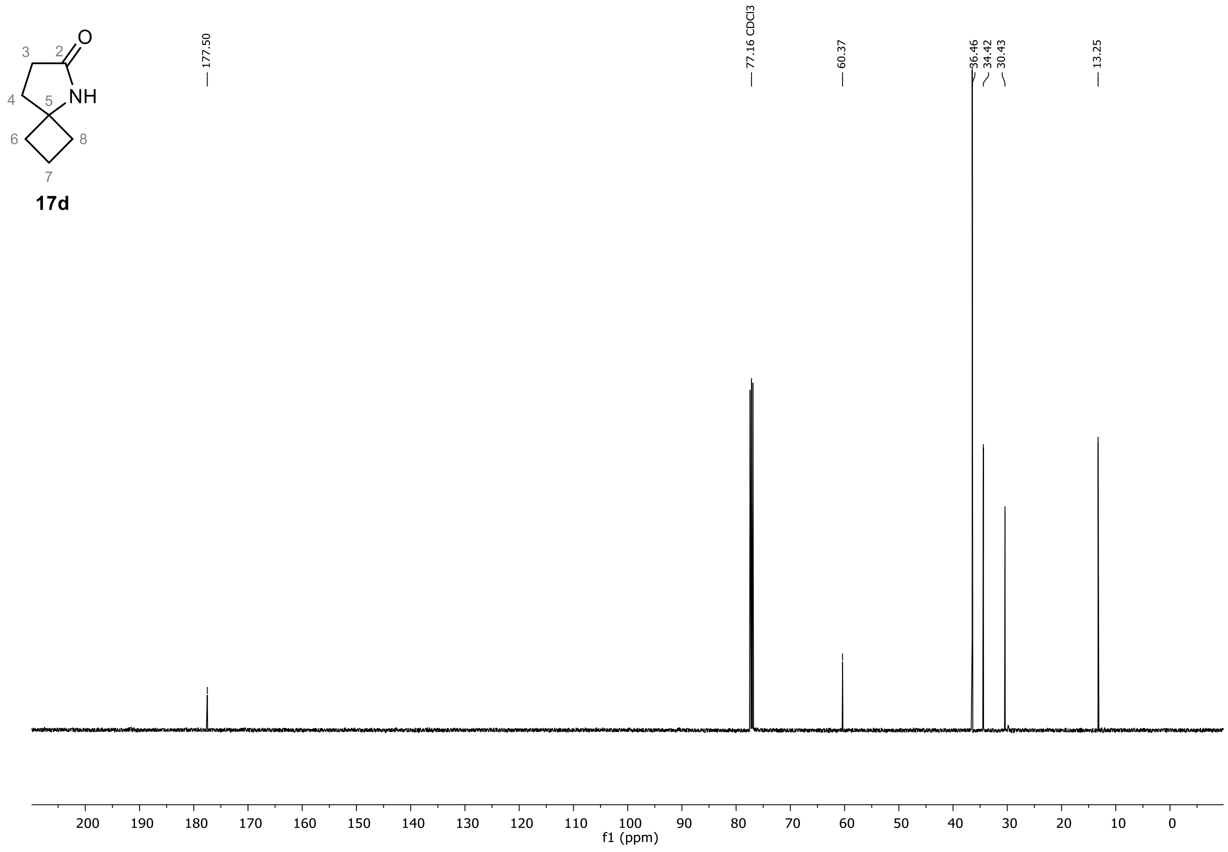

^{1}H NMR (500 MHz, CDCl$_3$)

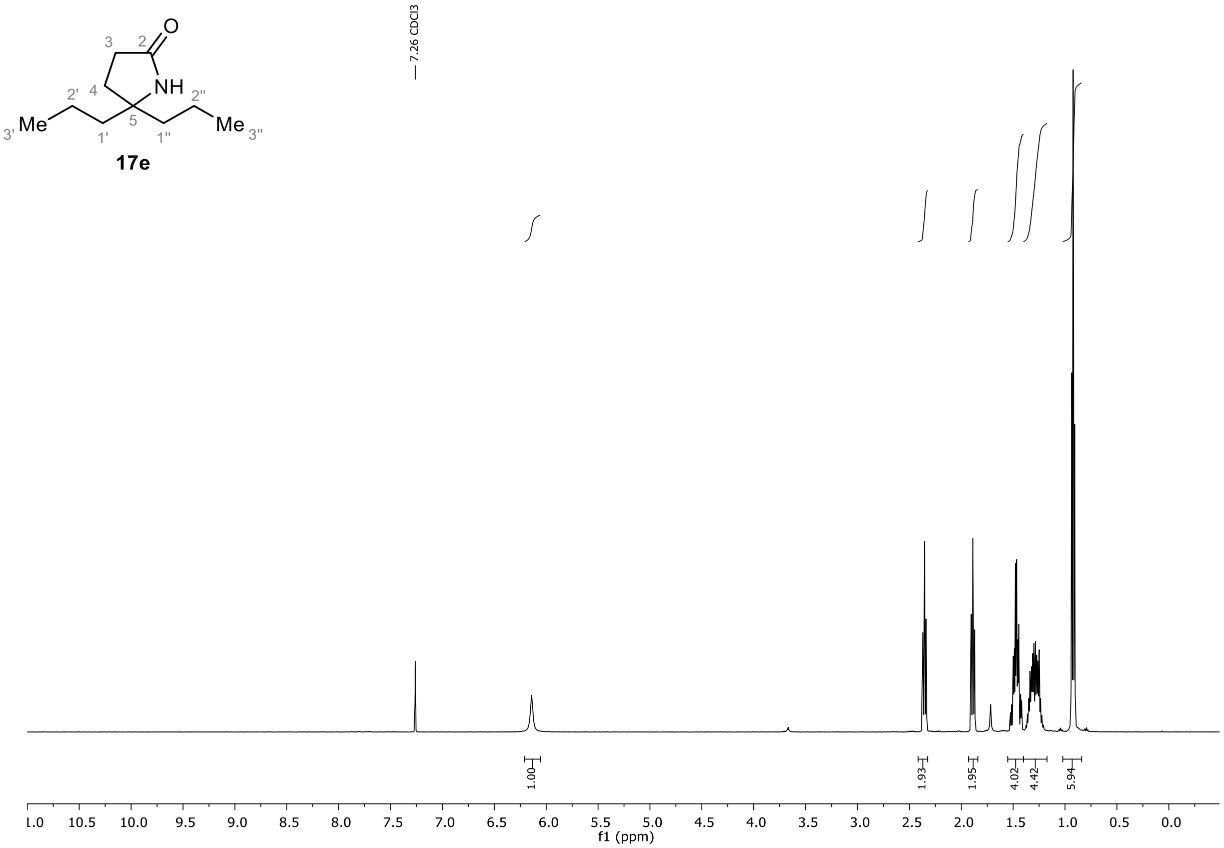

17e

^{13}C{^{1}H} NMR (126 MHz, CDCl$_3$)

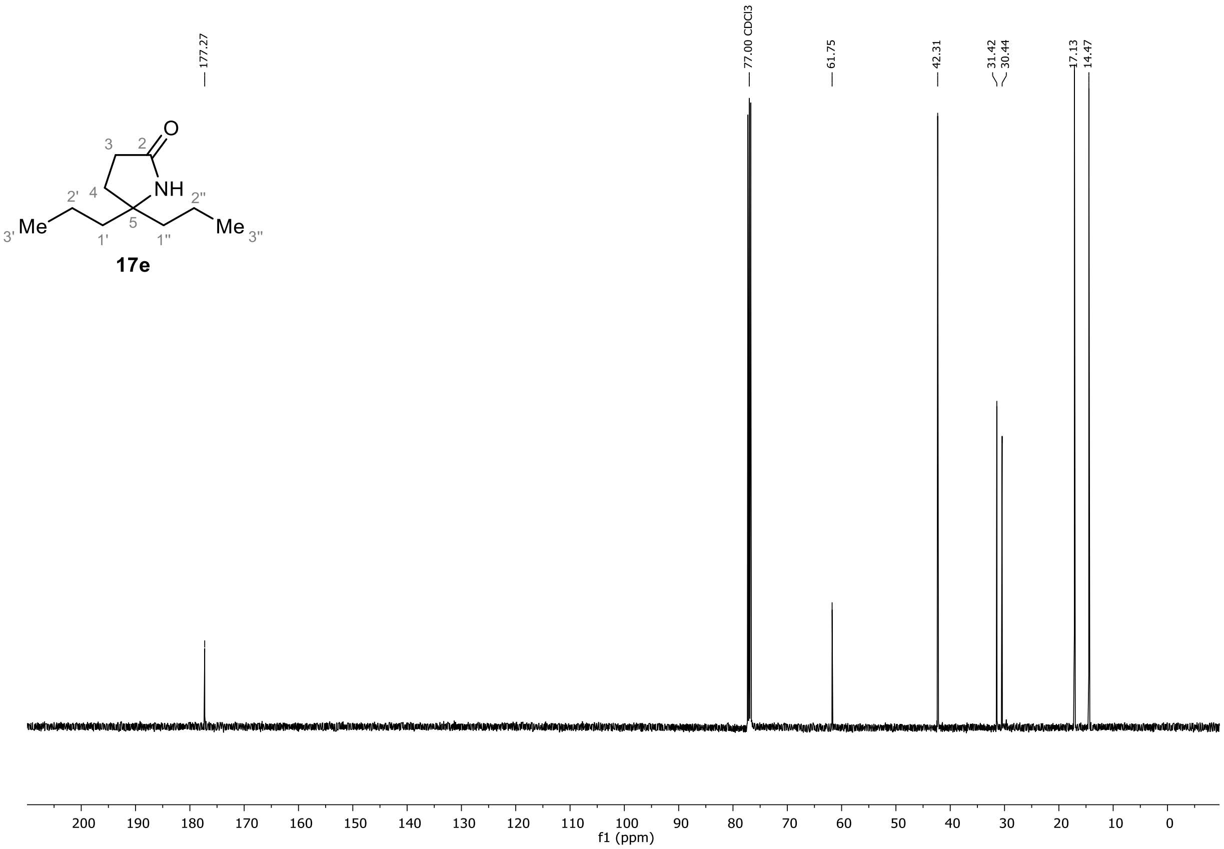

17e

^{1}H NMR (500 MHz, $CDCl_3$)

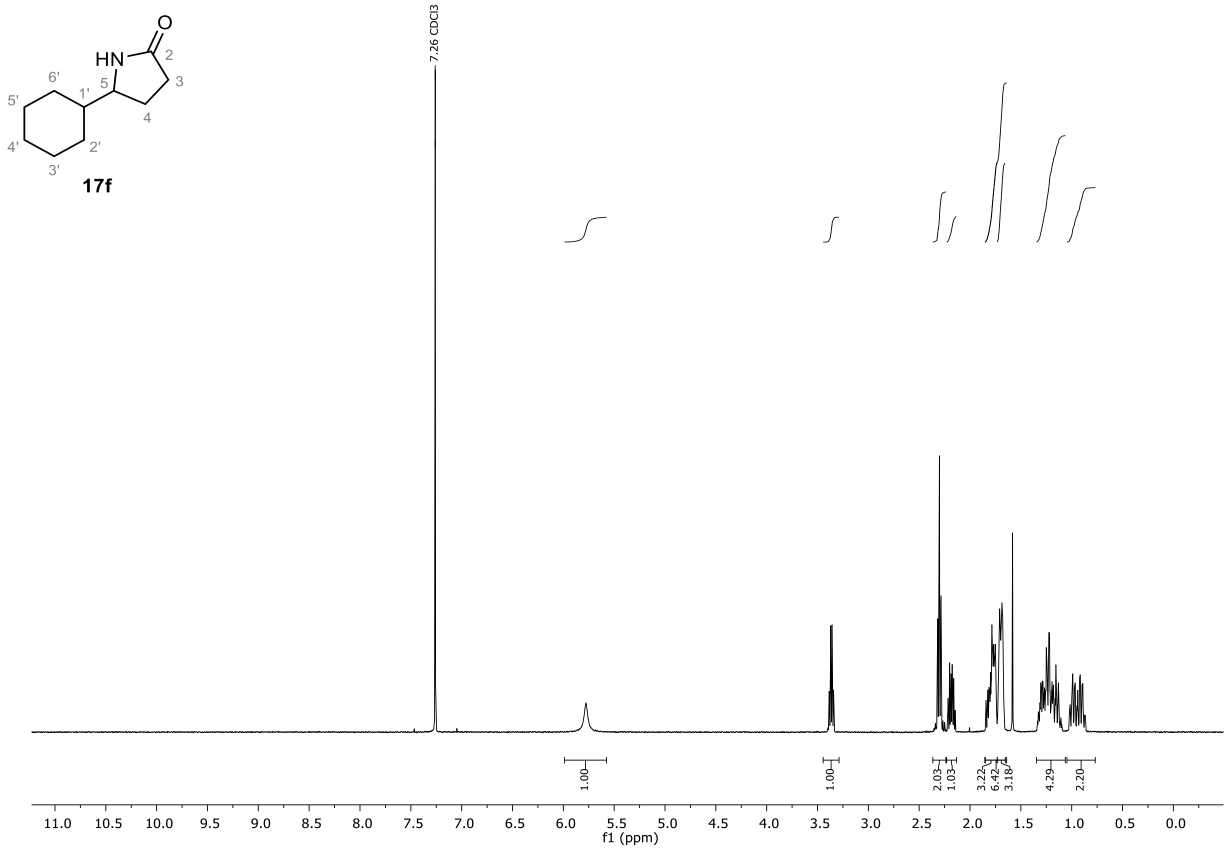

$^{13}C\{^{1}H\}$ NMR (126 MHz, $CDCl_3$)

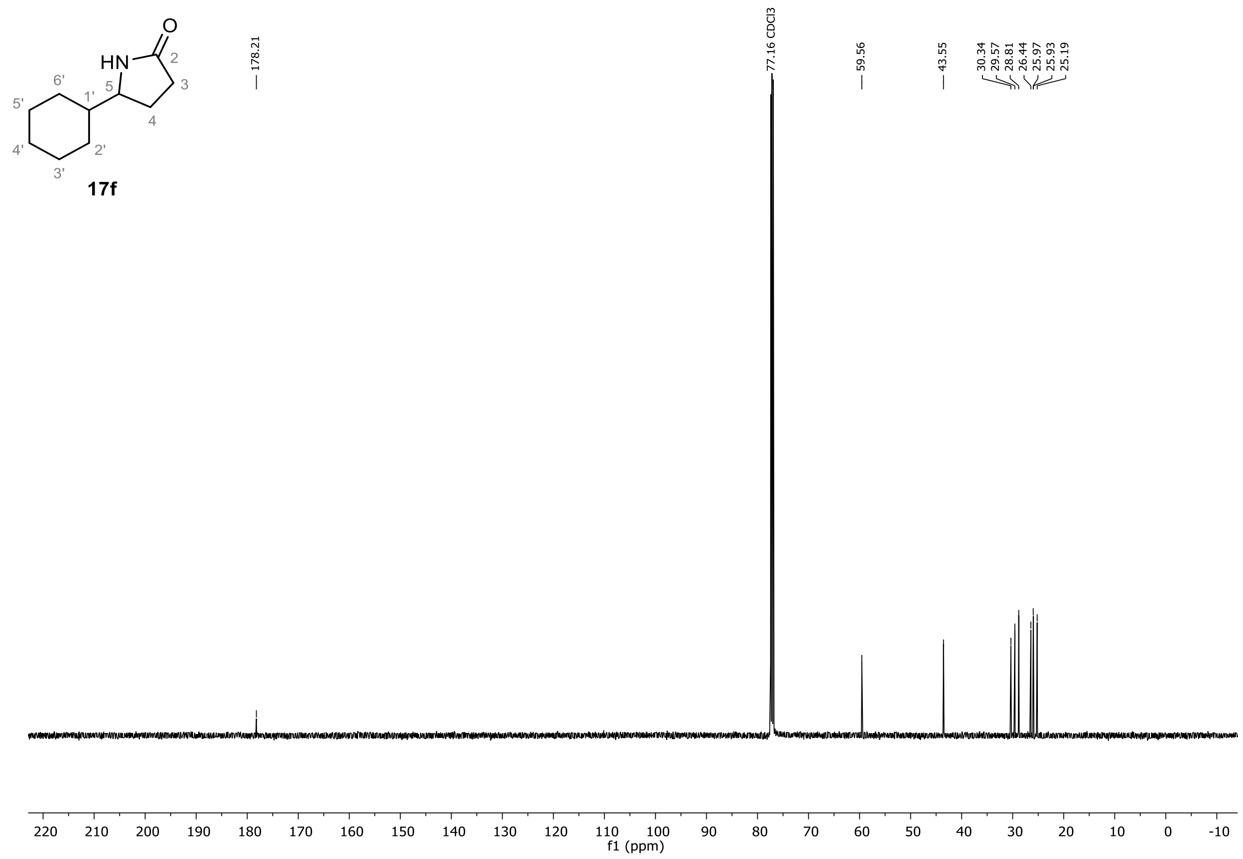

^{1}H NMR (500 MHz, $CDCl_3$)

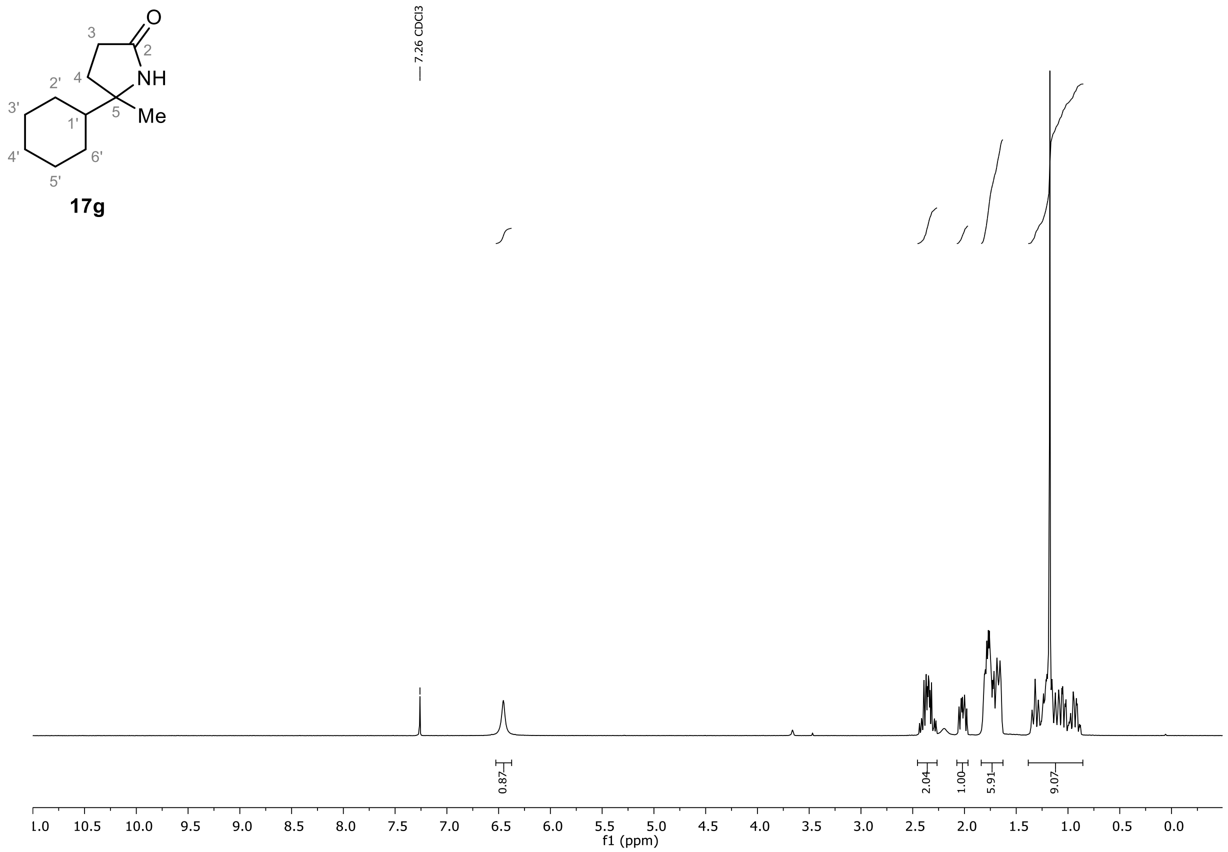

$^{13}C\{^1H\}$ NMR (126 MHz, $CDCl_3$)

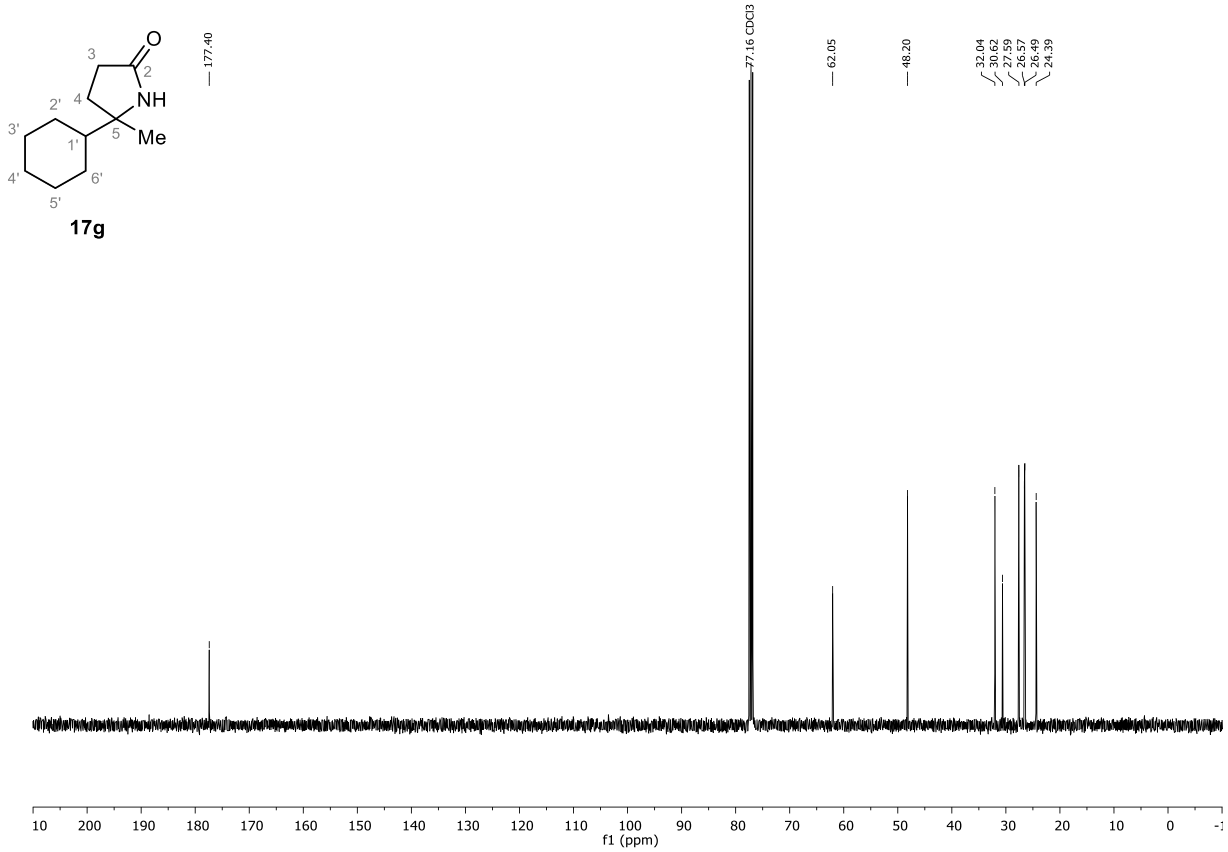

^1H NMR (500 MHz, $CDCl_3$)

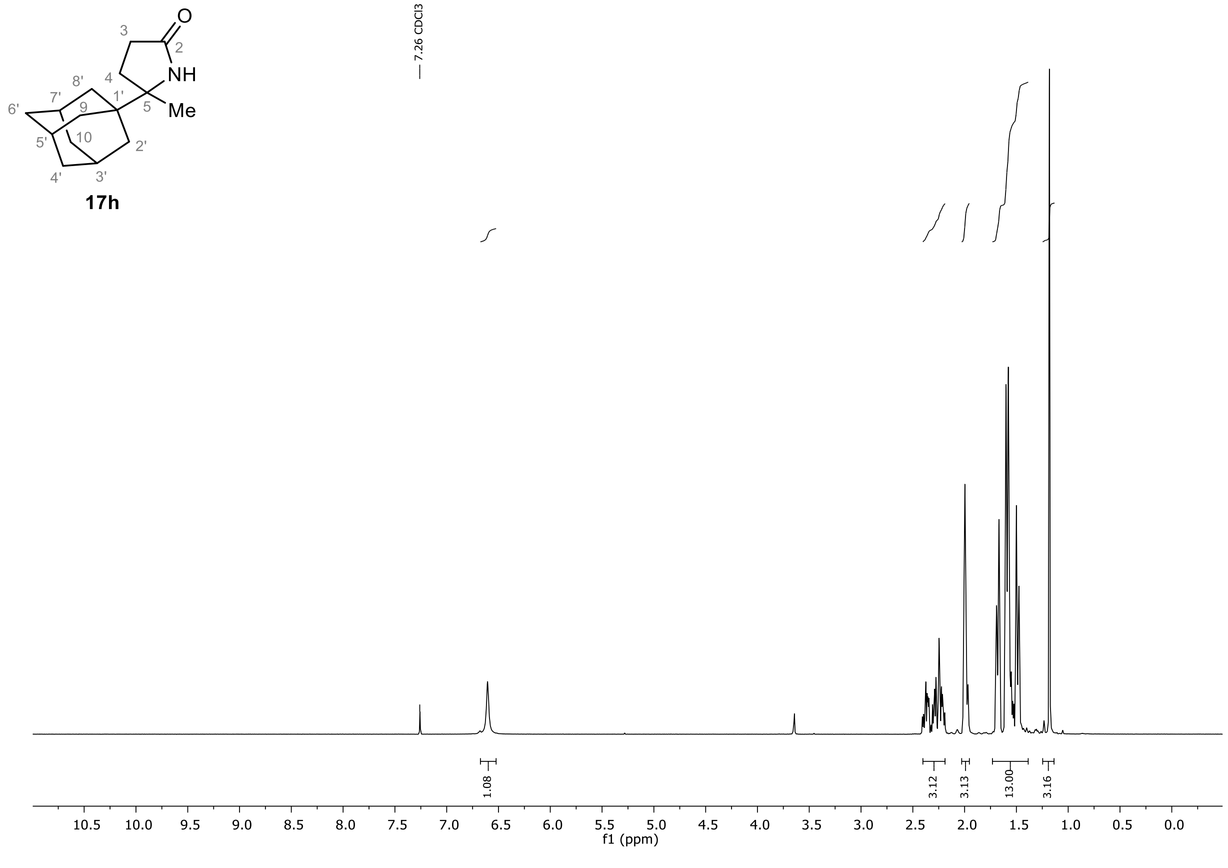

$^{13}C\{^1H\}$ NMR (126 MHz, $CDCl_3$)

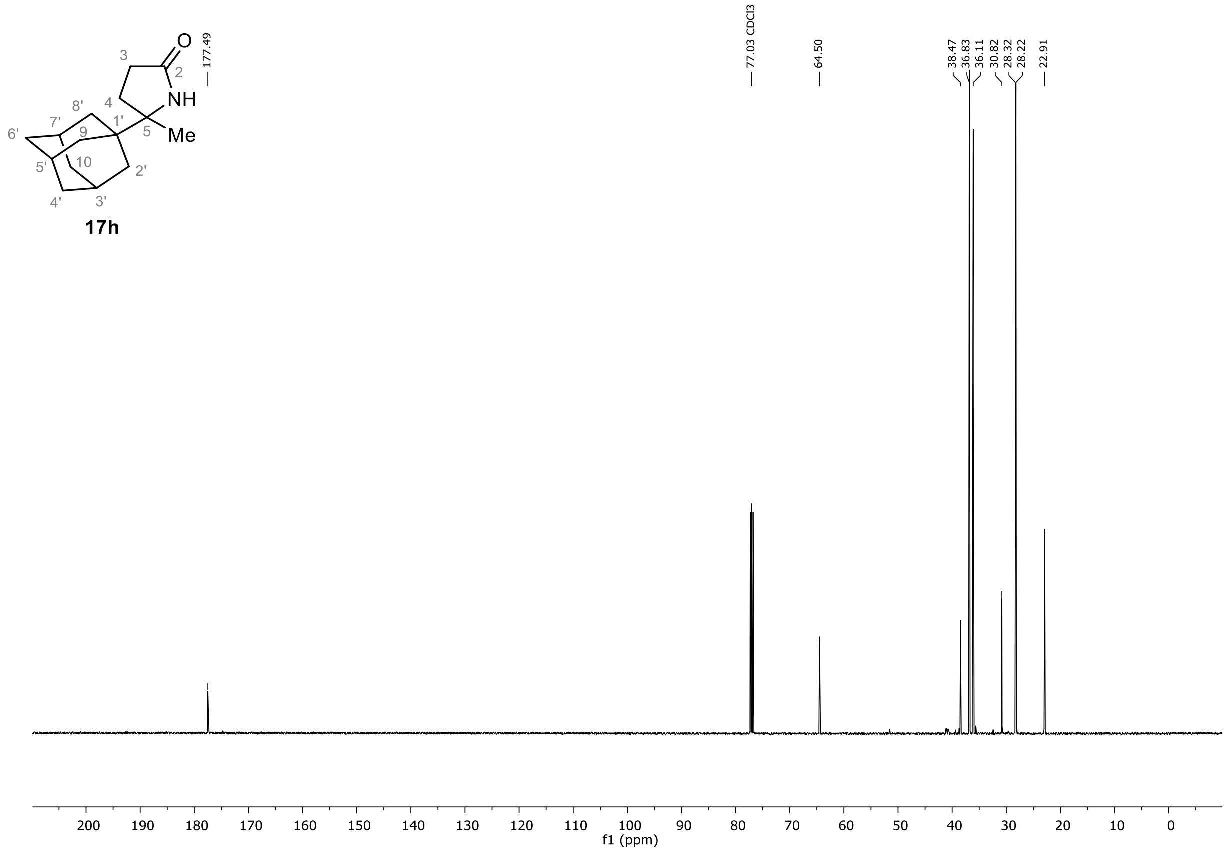

17h

^1H NMR (500 MHz, $CDCl_3$)

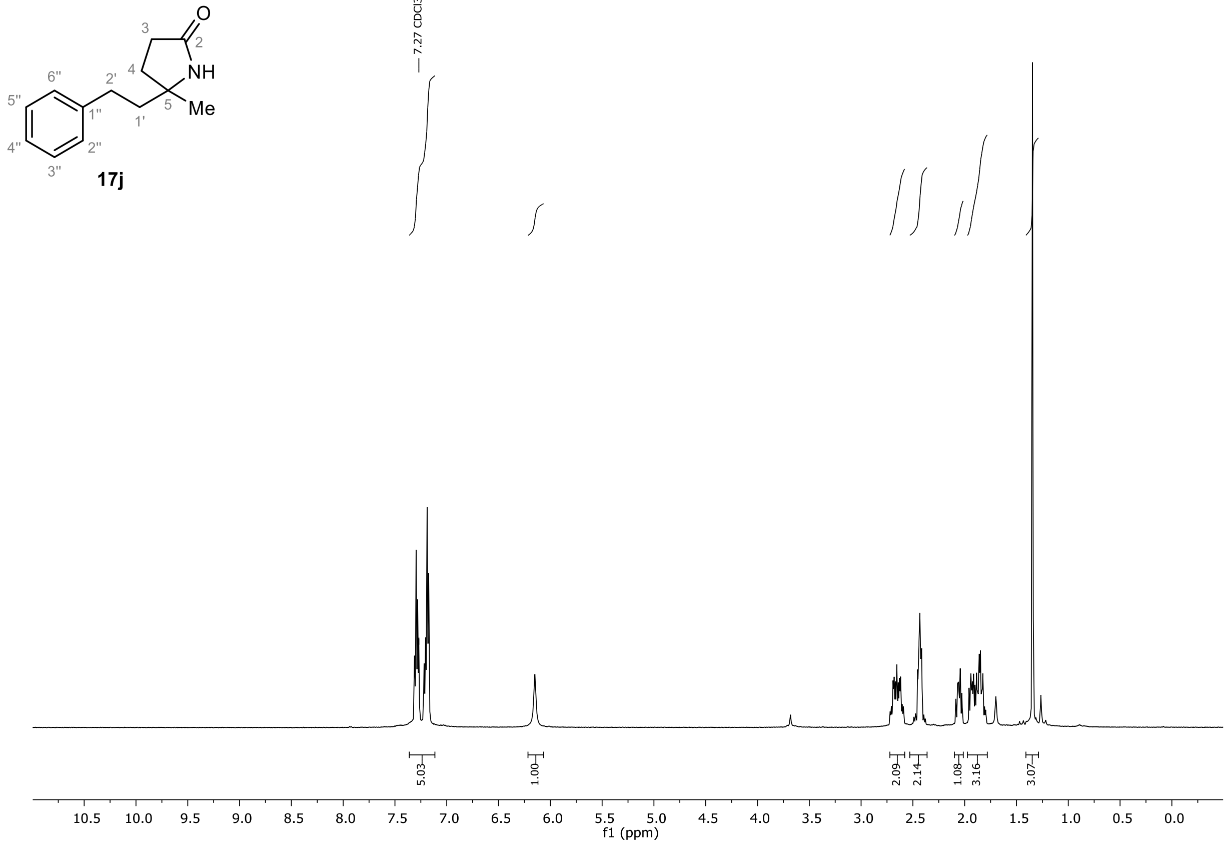

17j

$^{13}C\{^{1}H\}$ NMR (126 MHz, $CDCl_3$)

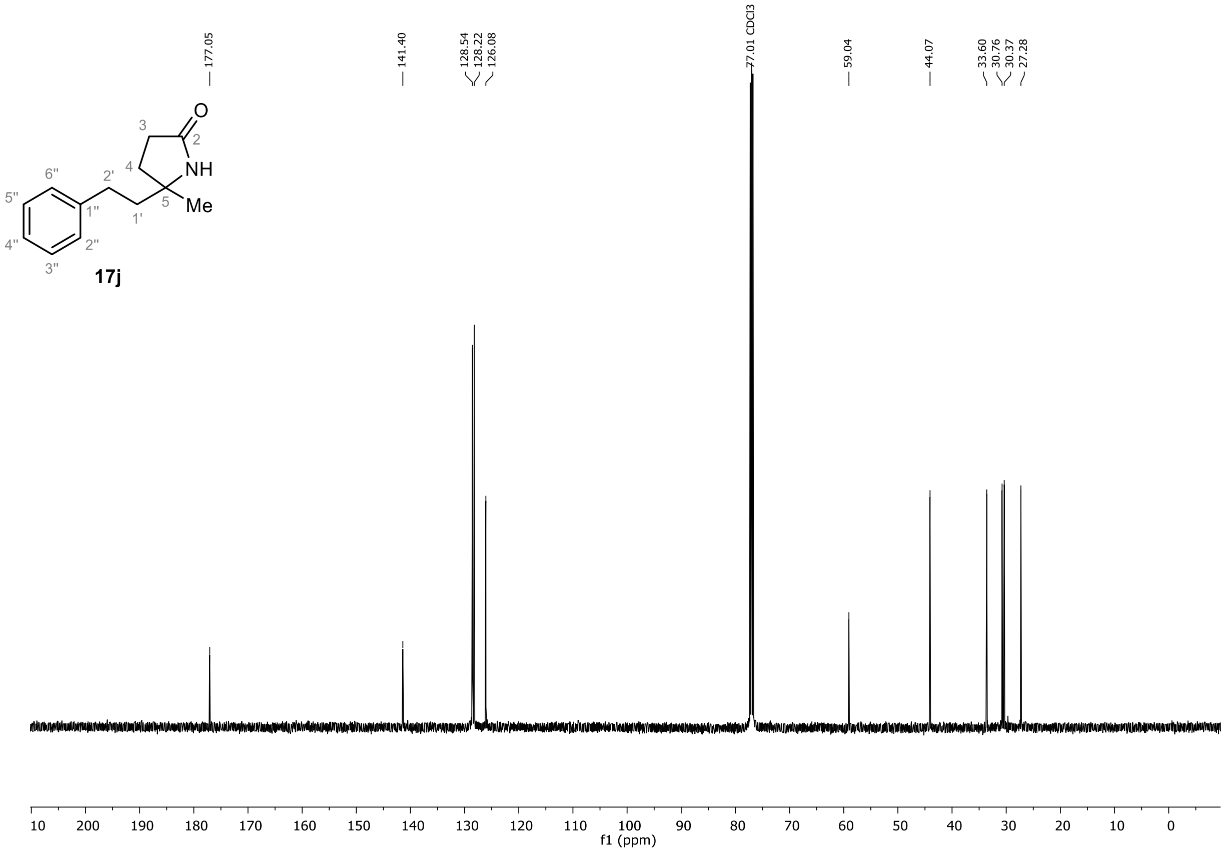

^{1}H NMR (500 MHz, $CDCl_3$)

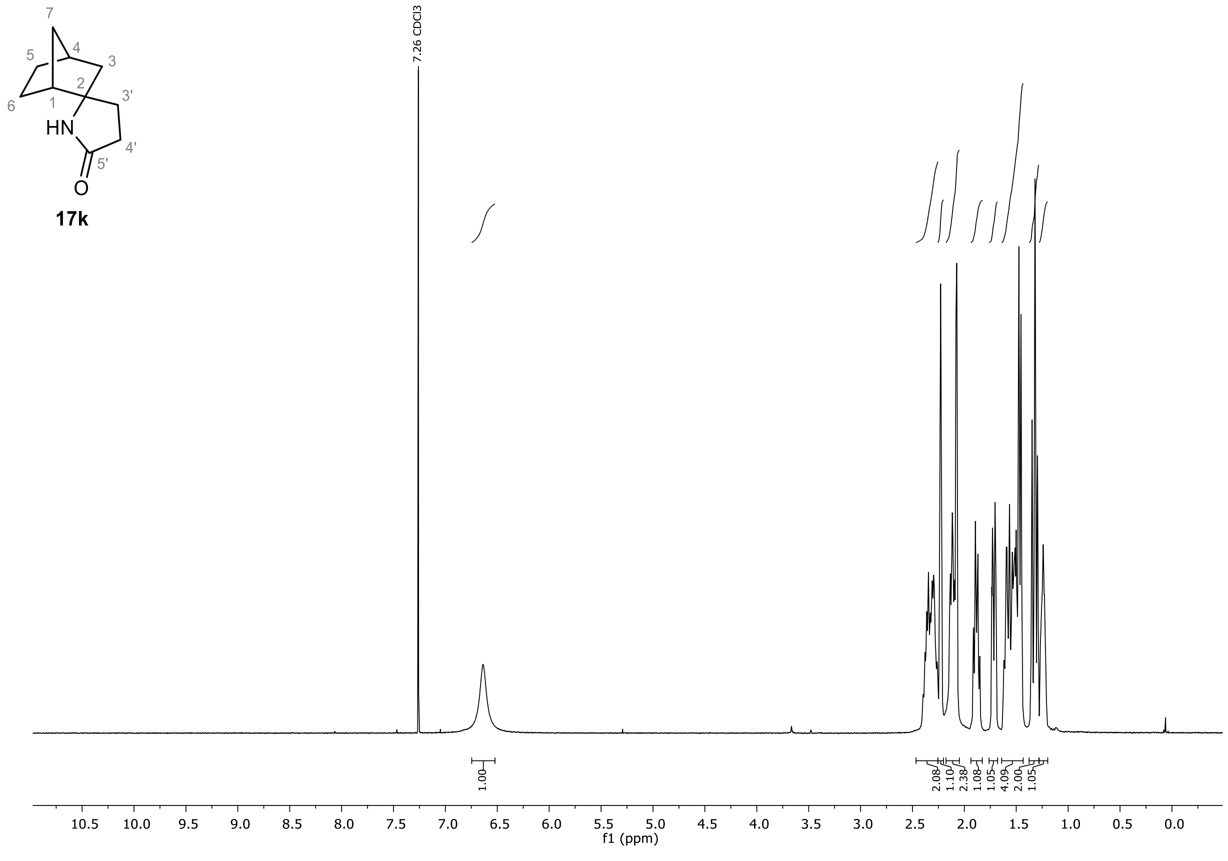

$^{13}C\{^1H\}$ NMR (126 MHz, $CDCl_3$)

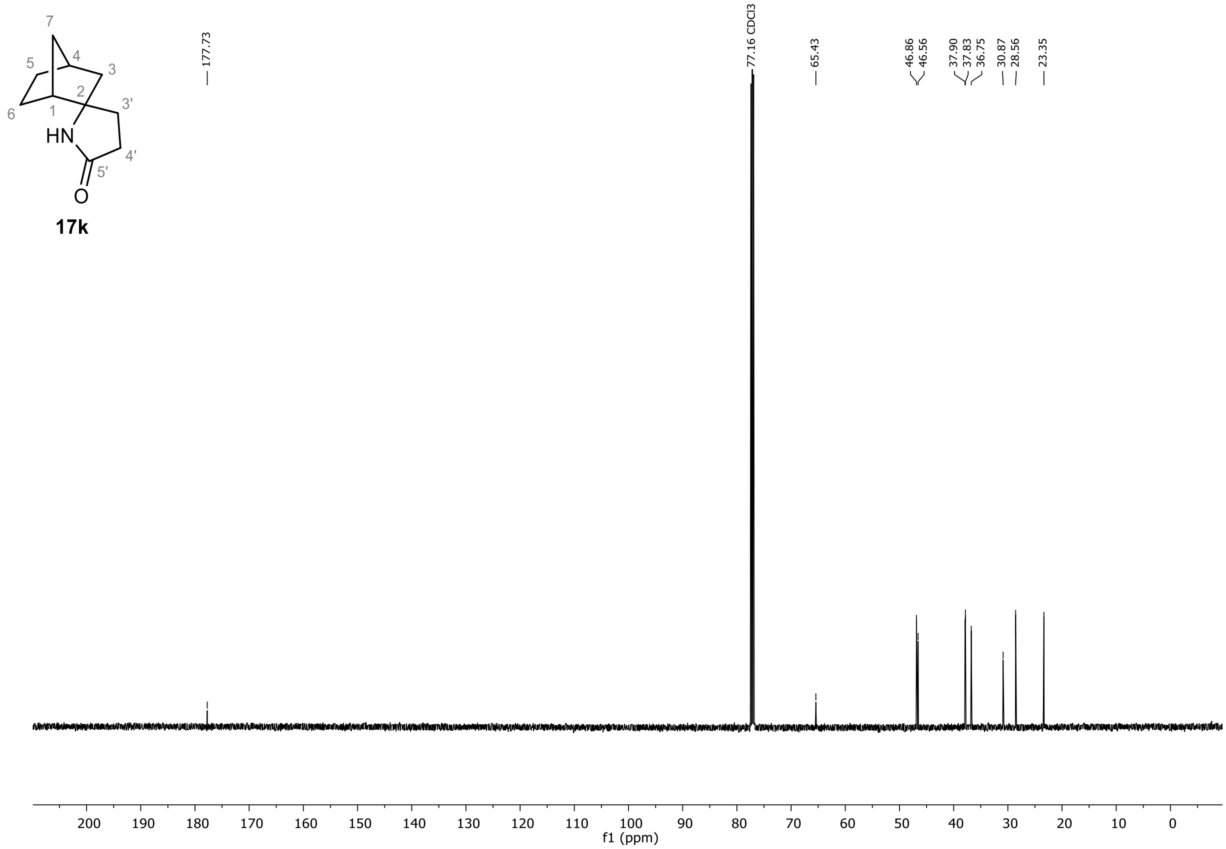

^{1}H NMR (500 MHz, $CDCl_3$)

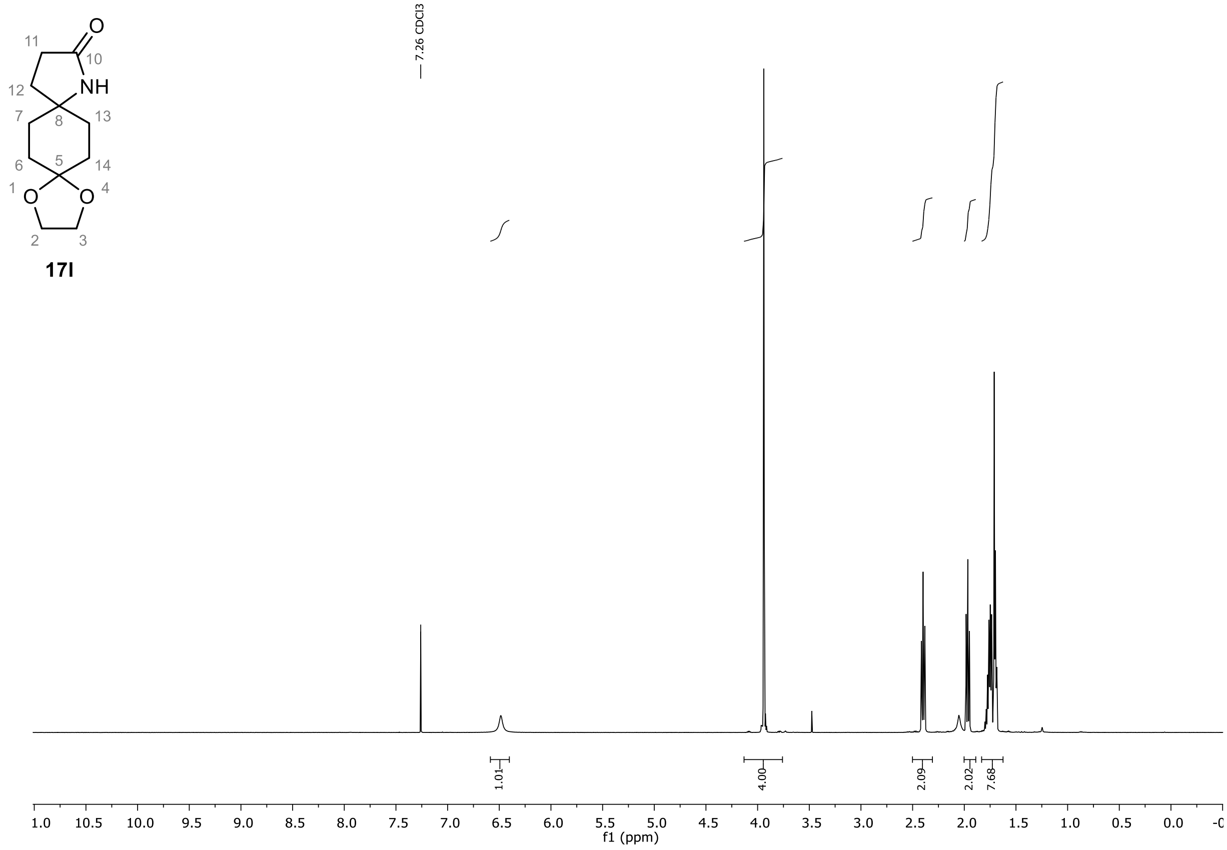

$^{13}C\{^1H\}$ NMR (126 MHz, $CDCl_3$)

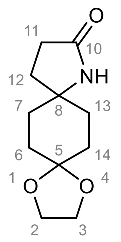

17l

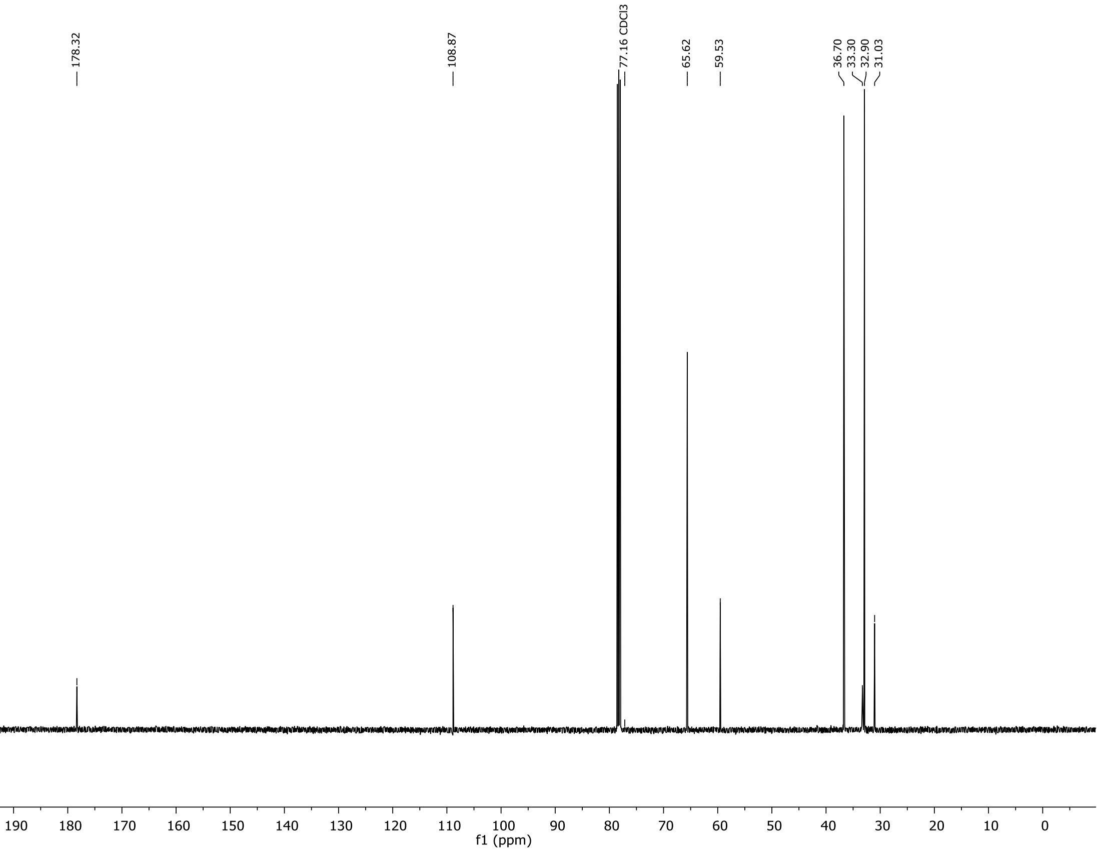

^1H NMR (500 MHz, $CDCl_3$)

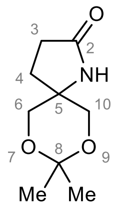

17m

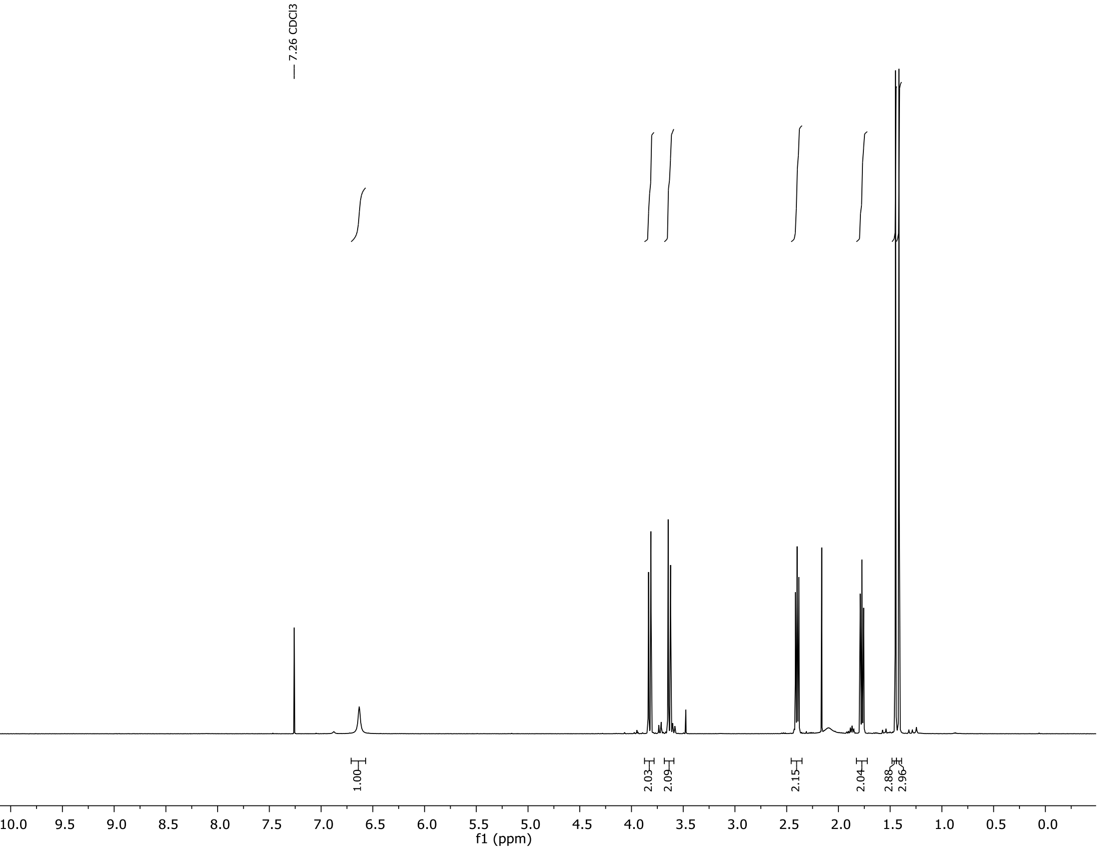

$^{13}C\{^1H\}$ NMR (126 MHz, $CDCl_3$)

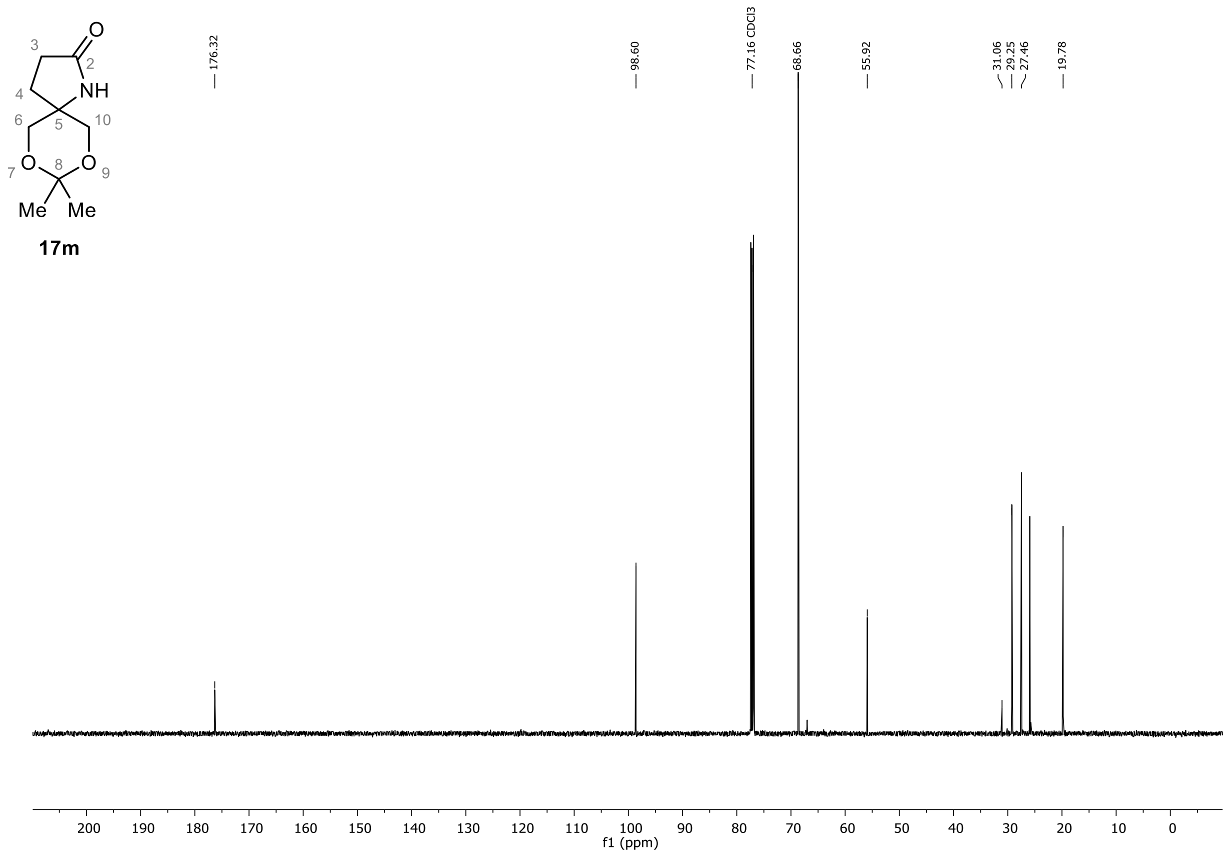

17m

^1H NMR (500 MHz, $CDCl_3$)

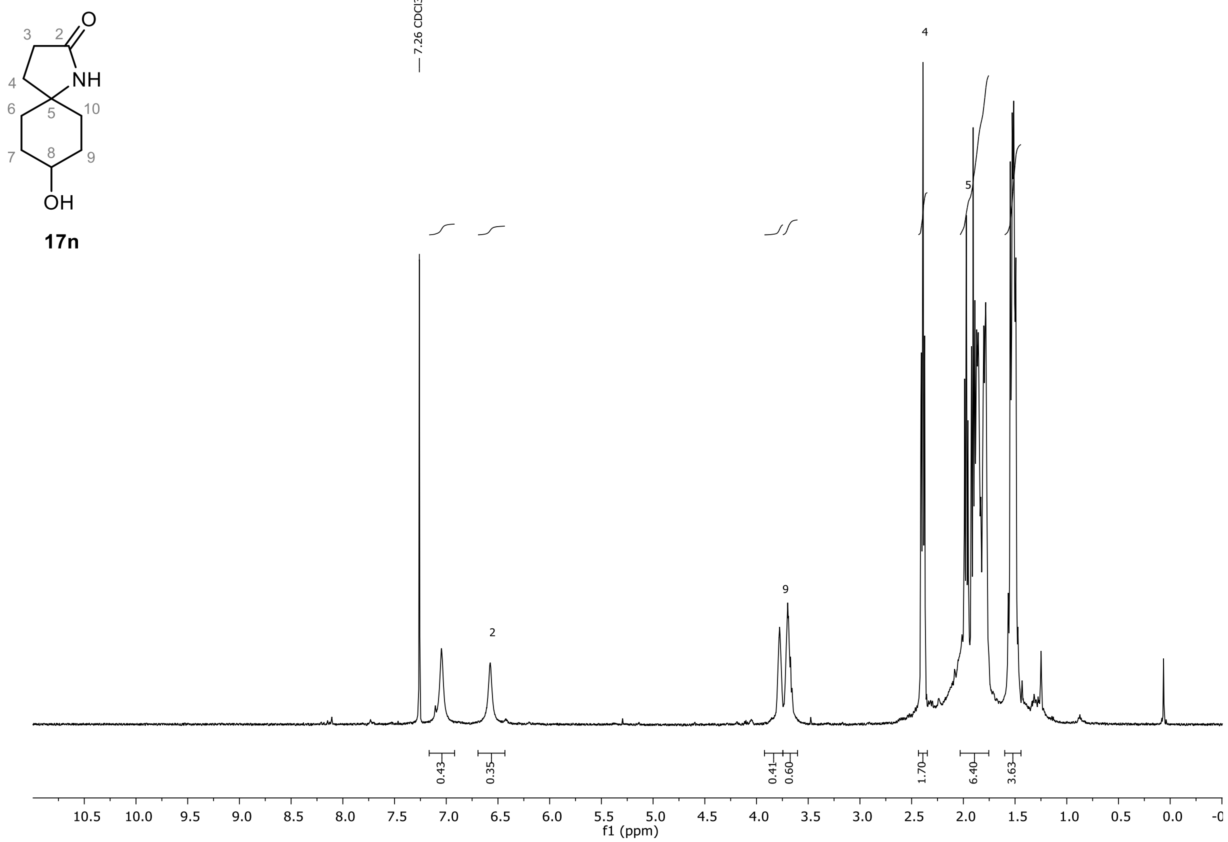

17n

$^{13}C\{^1H\}$ NMR (126 MHz, $CDCl_3$)

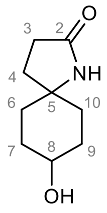

17n

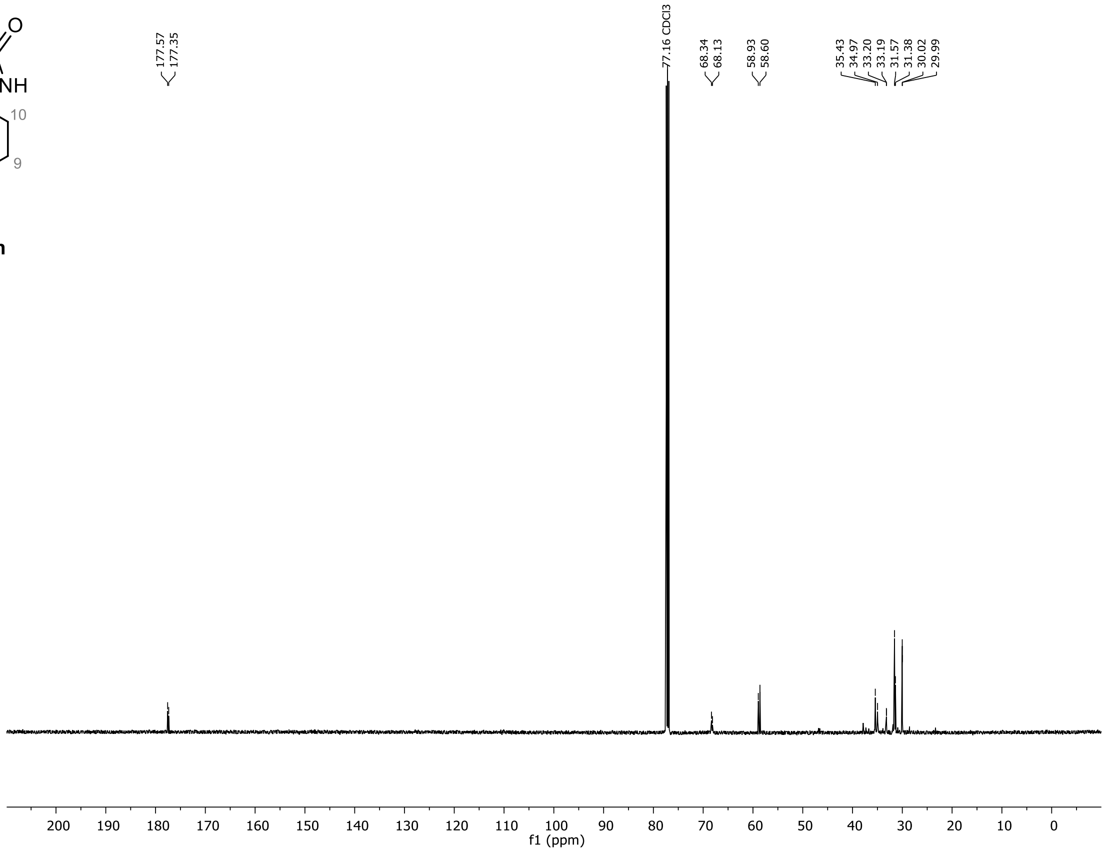

^1H NMR (500 MHz, $CDCl_3$)

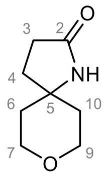

17o

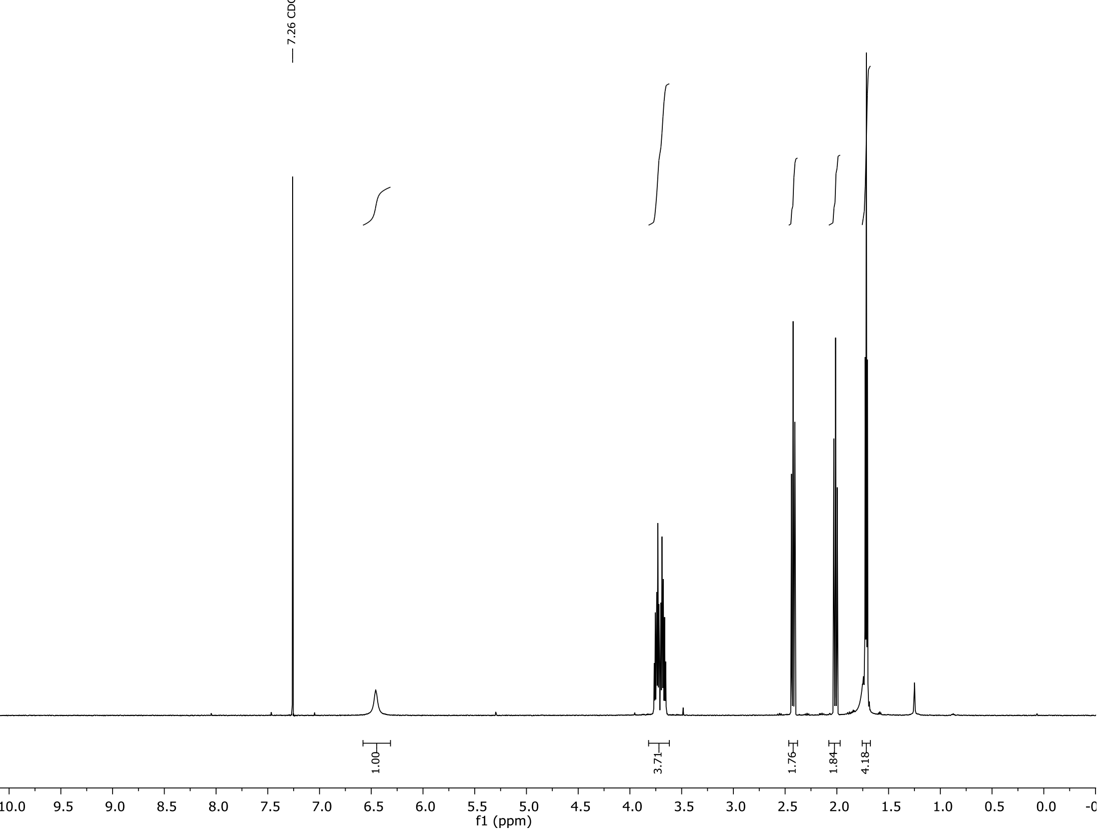

$^{13}C\{^{1}H\}$ NMR (126 MHz, $CDCl_3$)

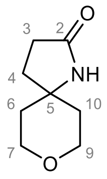

17o

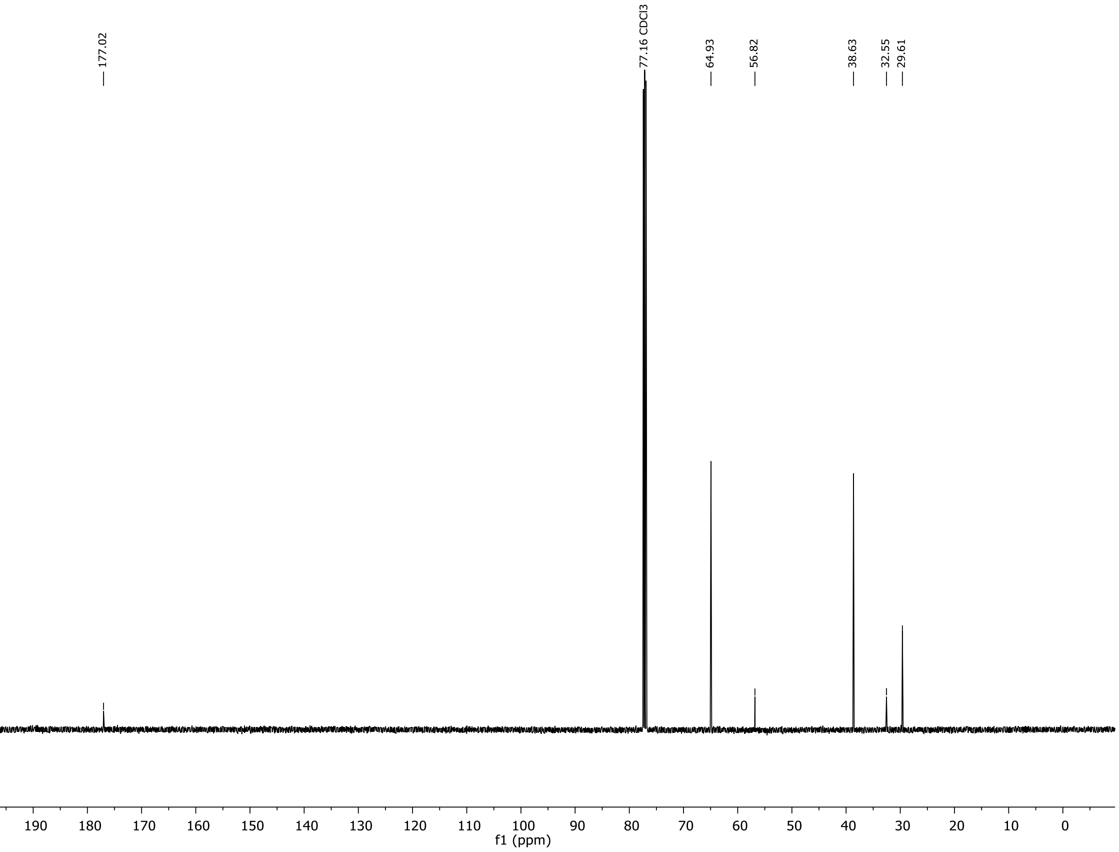

^{1}H NMR (500 MHz, $CDCl_3$)

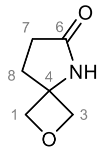

17p

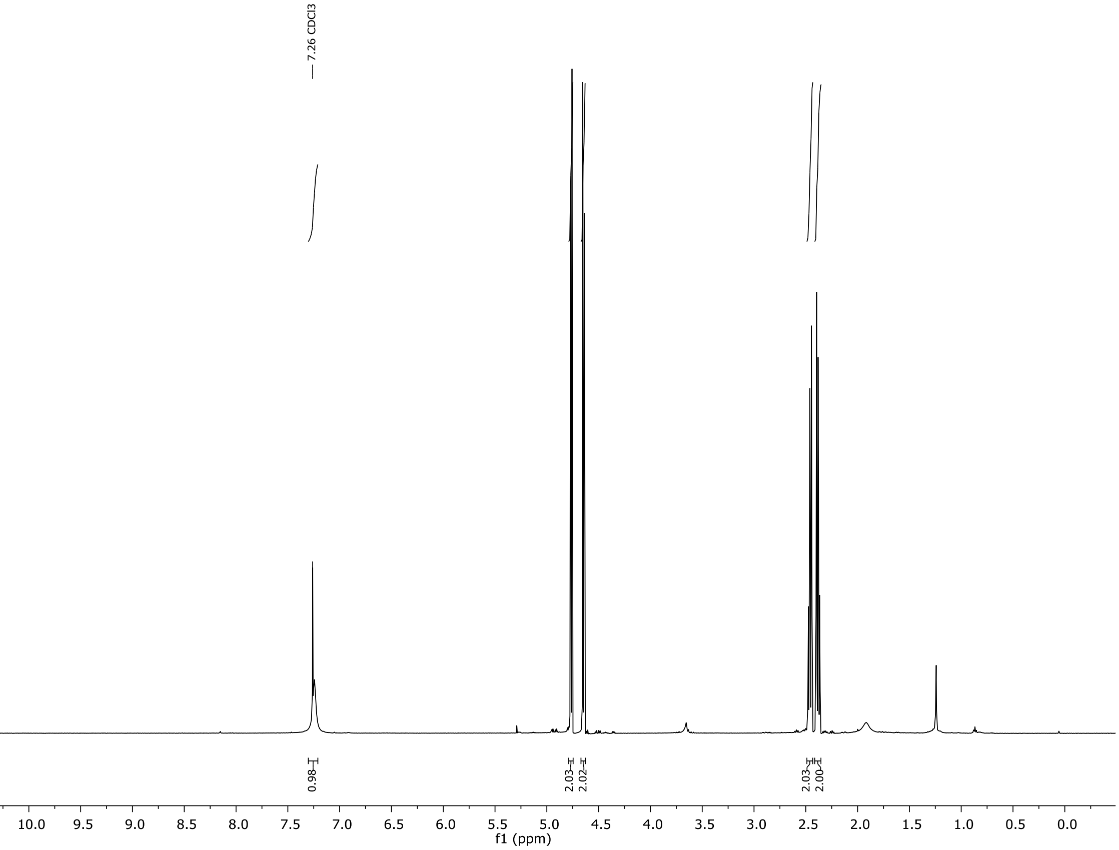

$^{13}C\{^{1}H\}$ NMR (126 MHz, $CDCl_3$)

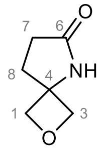

17p

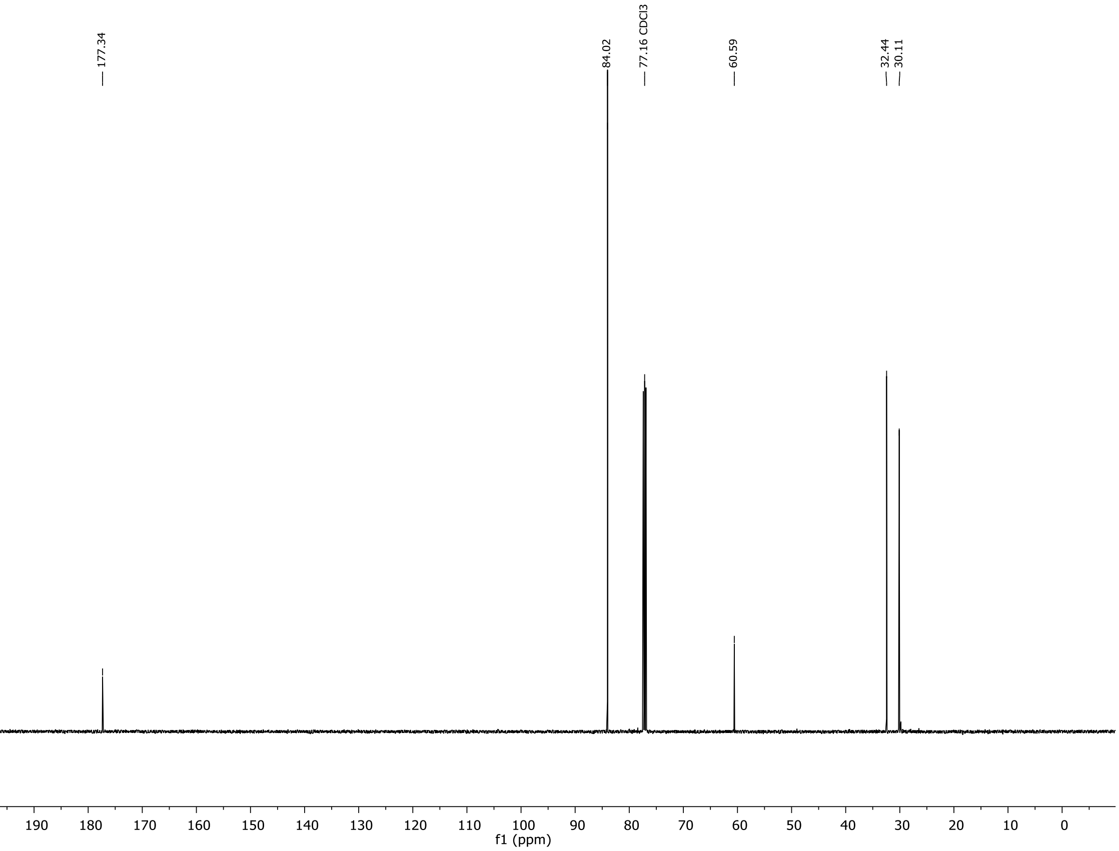

^{1}H NMR (500 MHz, $CDCl_3$)

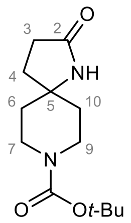

17q

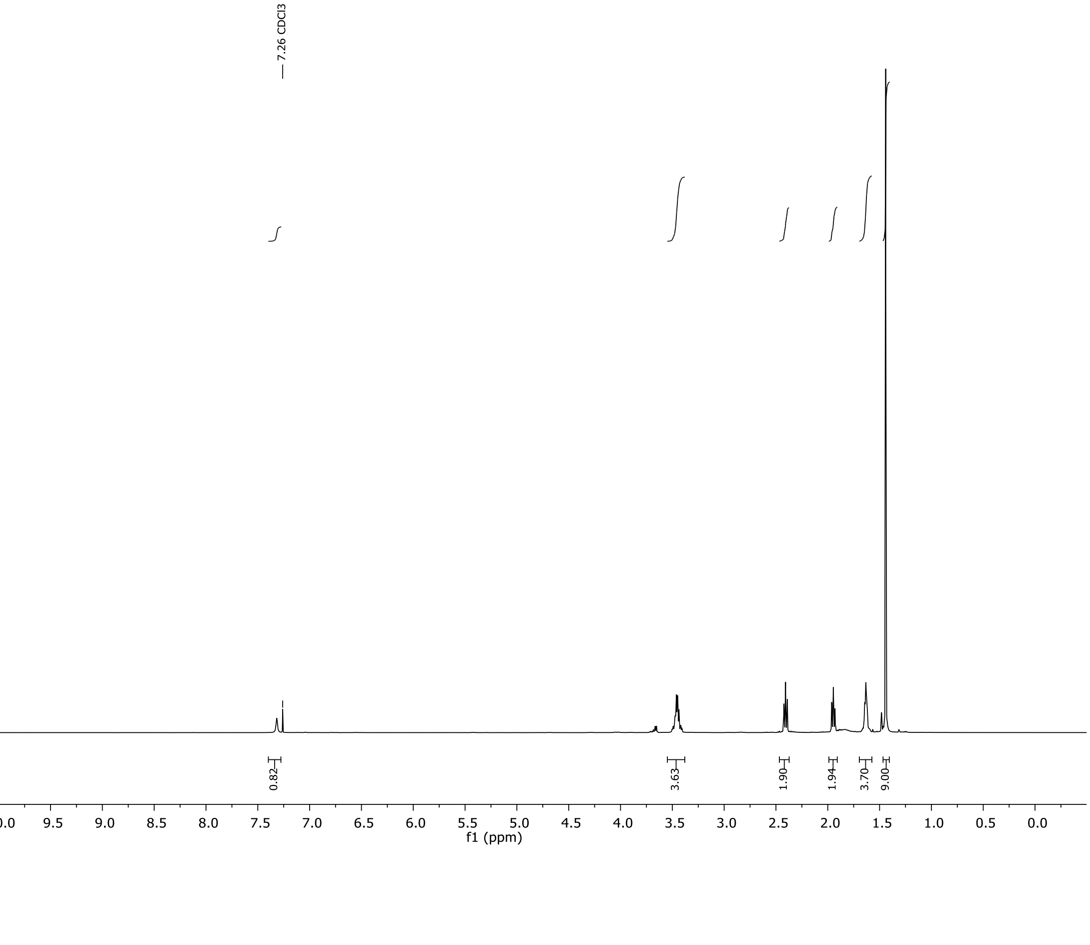

$^{13}C\{^{1}H\}$ NMR (126 MHz, $CDCl_3$)

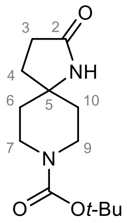

17q

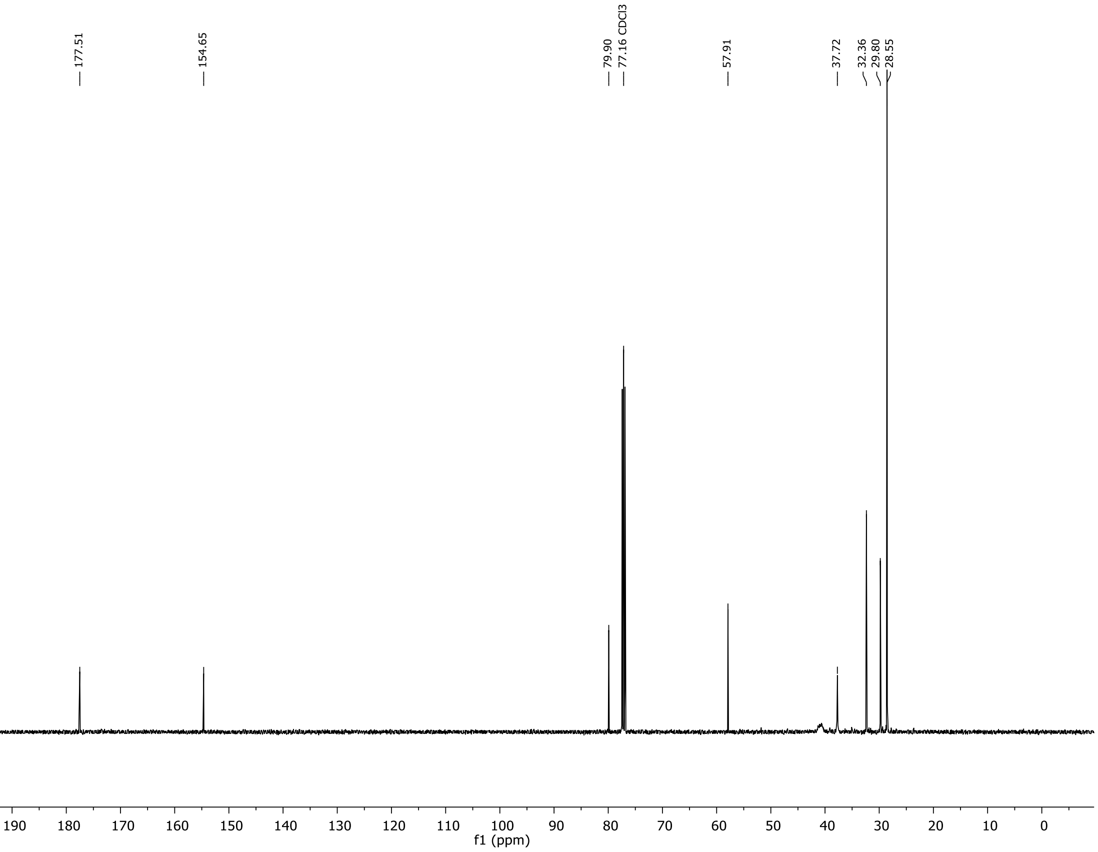

^{1}H NMR (500 MHz, $CDCl_3$)

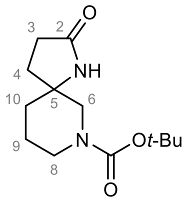

17r

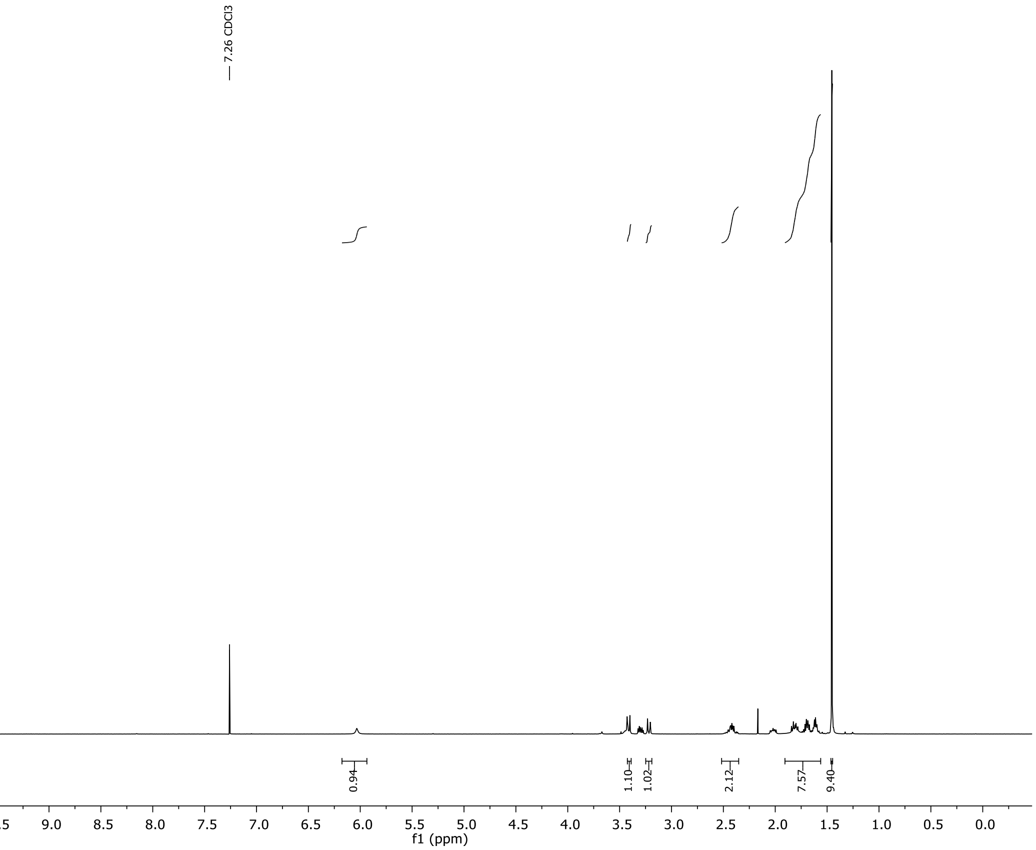

$^{13}C\{^1H\}$ NMR (126 MHz, $CDCl_3$)

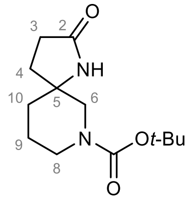

17r

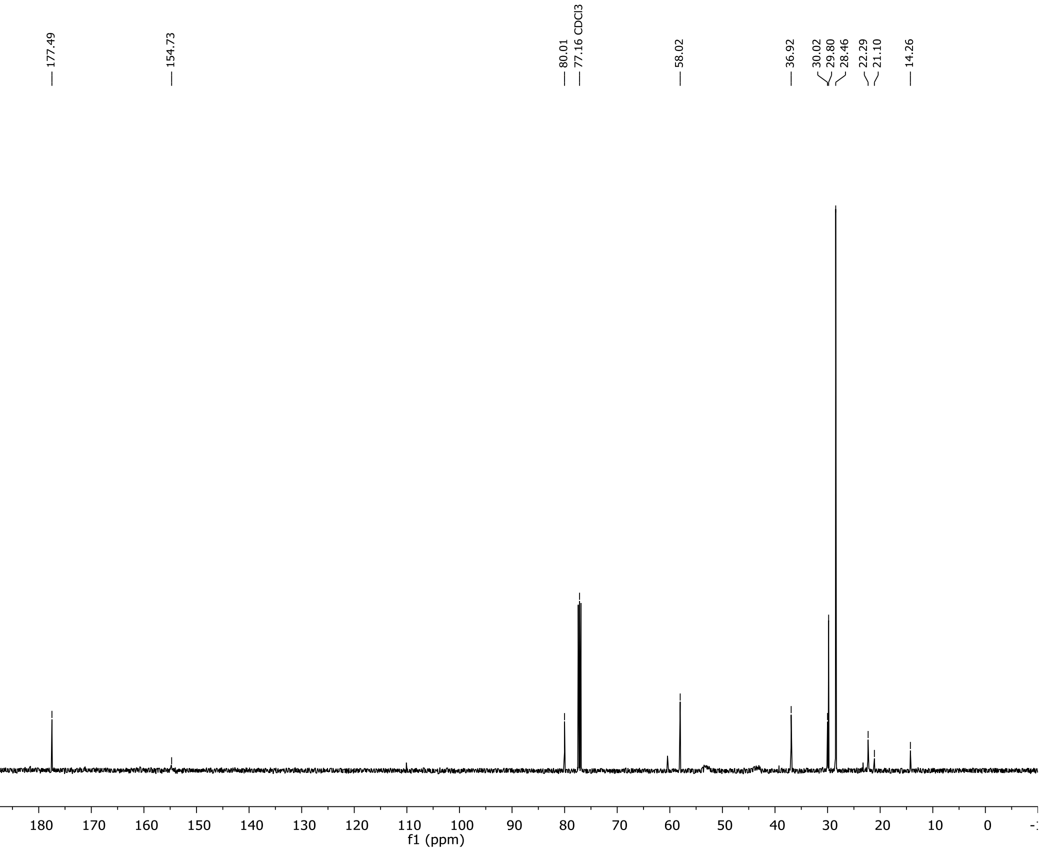

^1H NMR (500 MHz, $CDCl_3$)

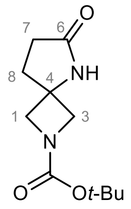

17s

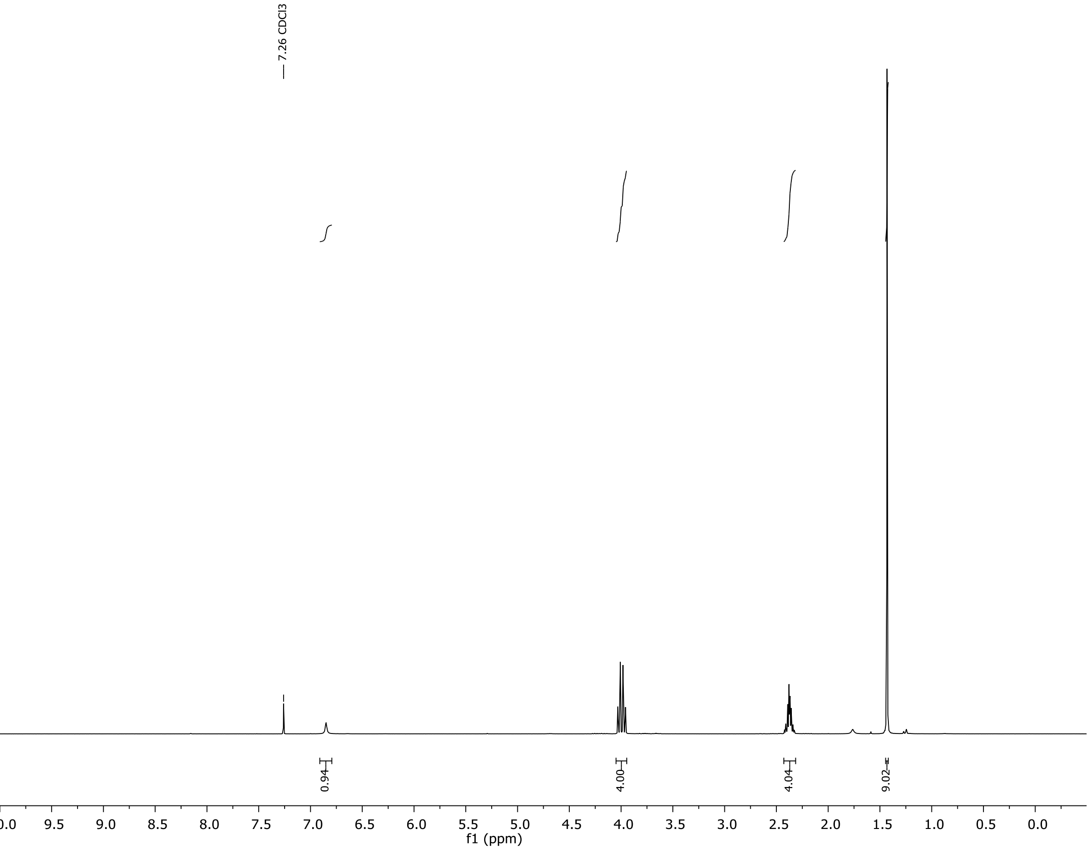

$^{13}C\{^{1}H\}$ NMR (126 MHz, $CDCl_3$)

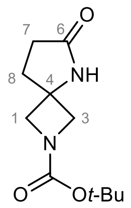

17s

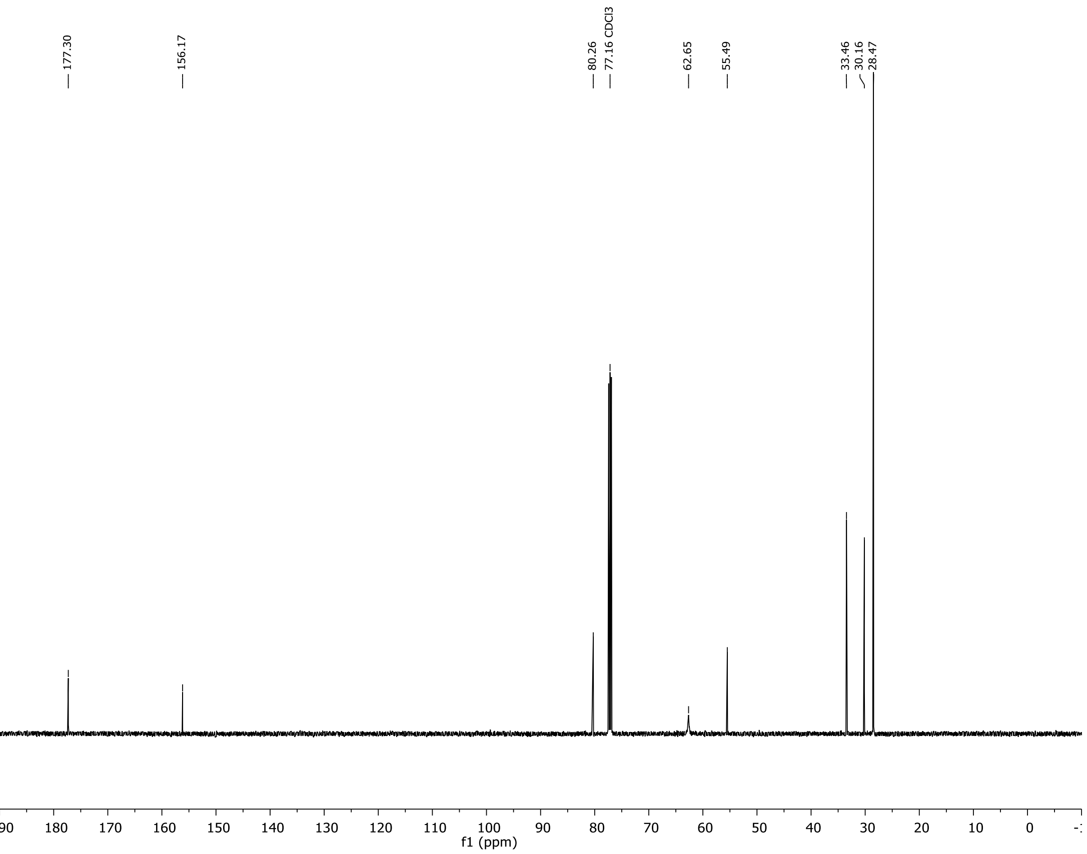

^{1}H NMR (500 MHz, $CDCl_3$)

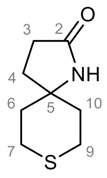

17t

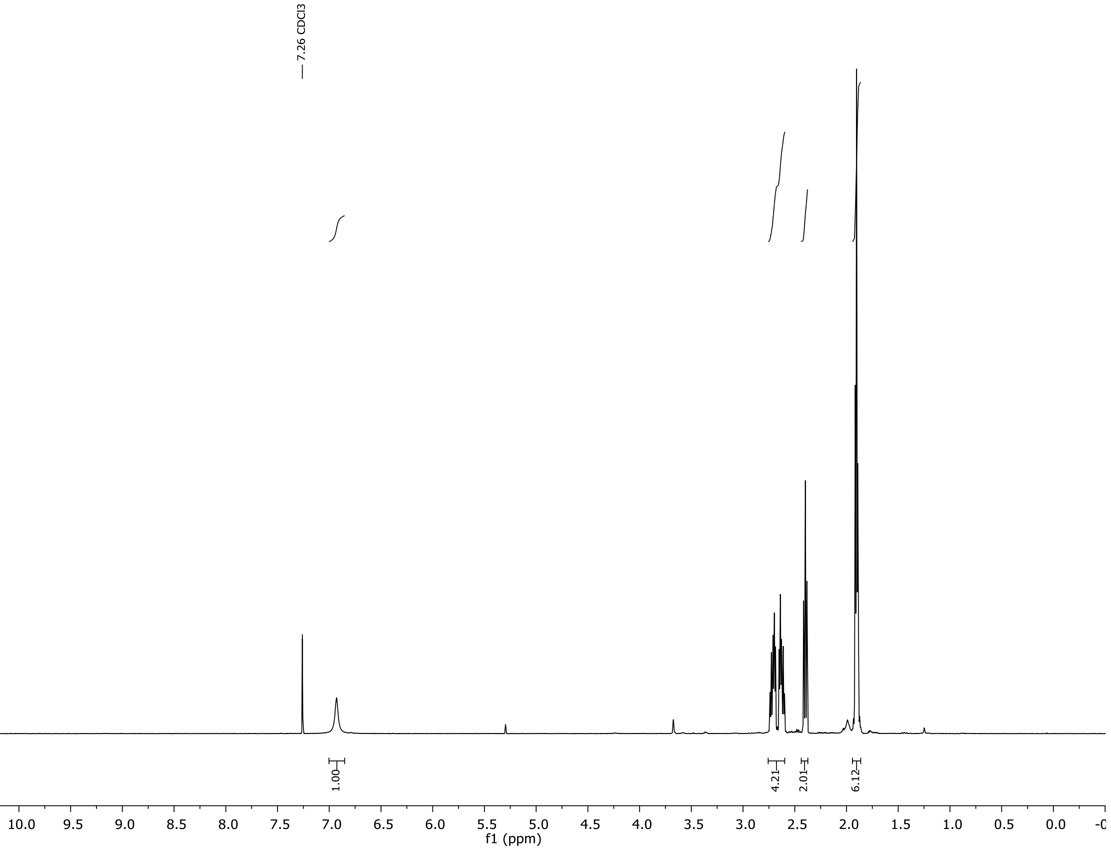

$^{13}C\{^1H\}$ NMR (126 MHz, $CDCl_3$)

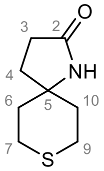

17t

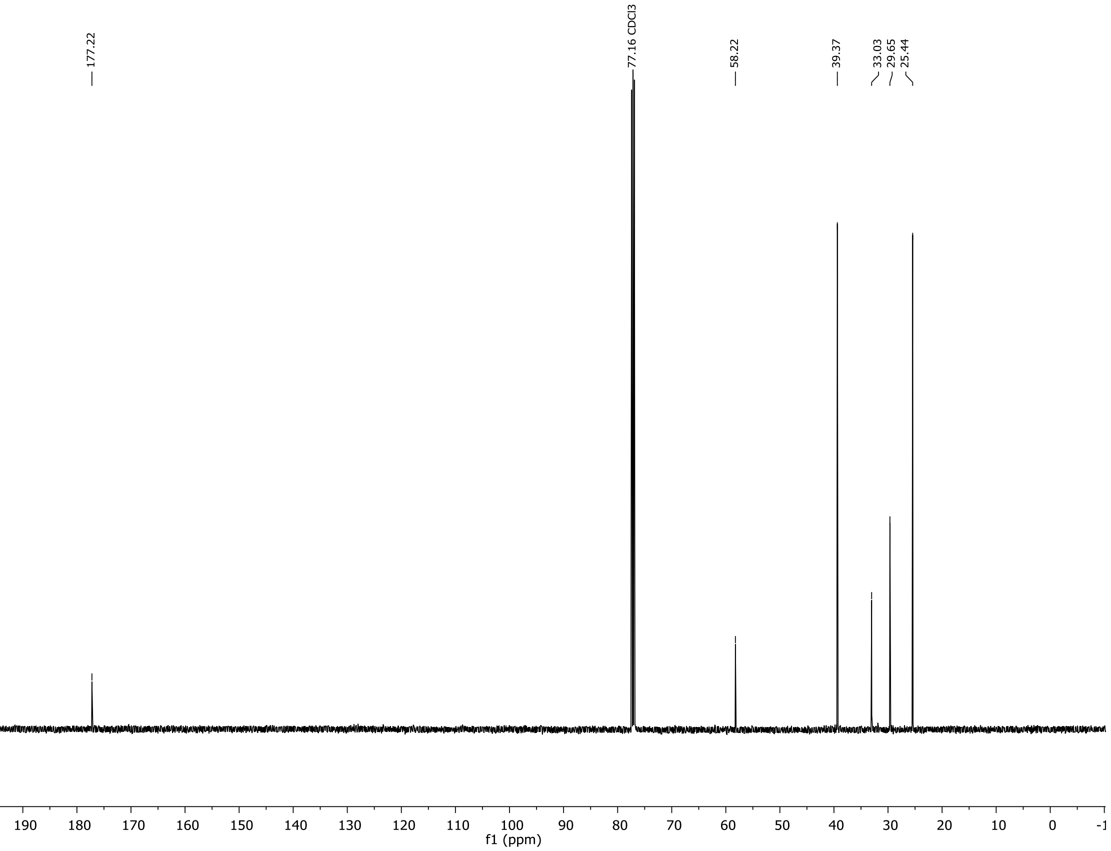

^1H NMR (500 MHz, DMSO-d_6)

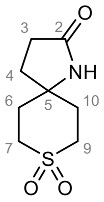

17u

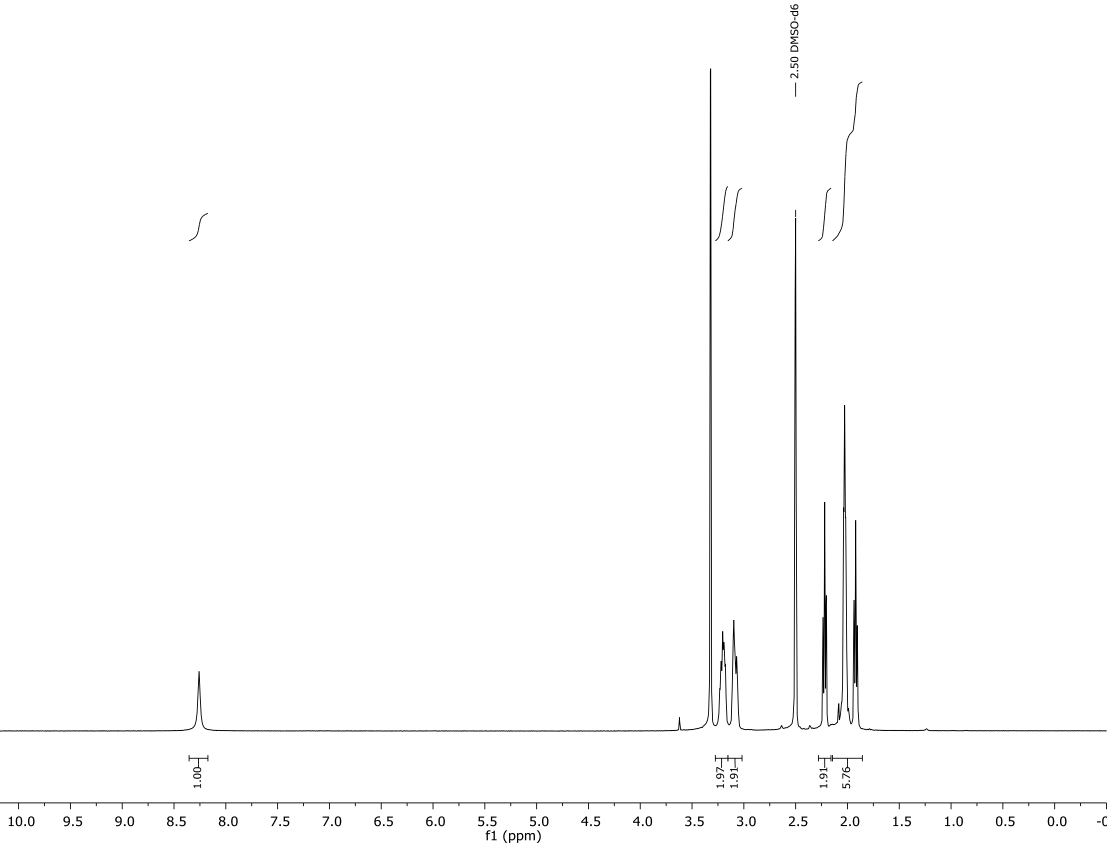

$^{13}C\{^{1}H\}$ NMR (126 MHz, DMSO-d_6)

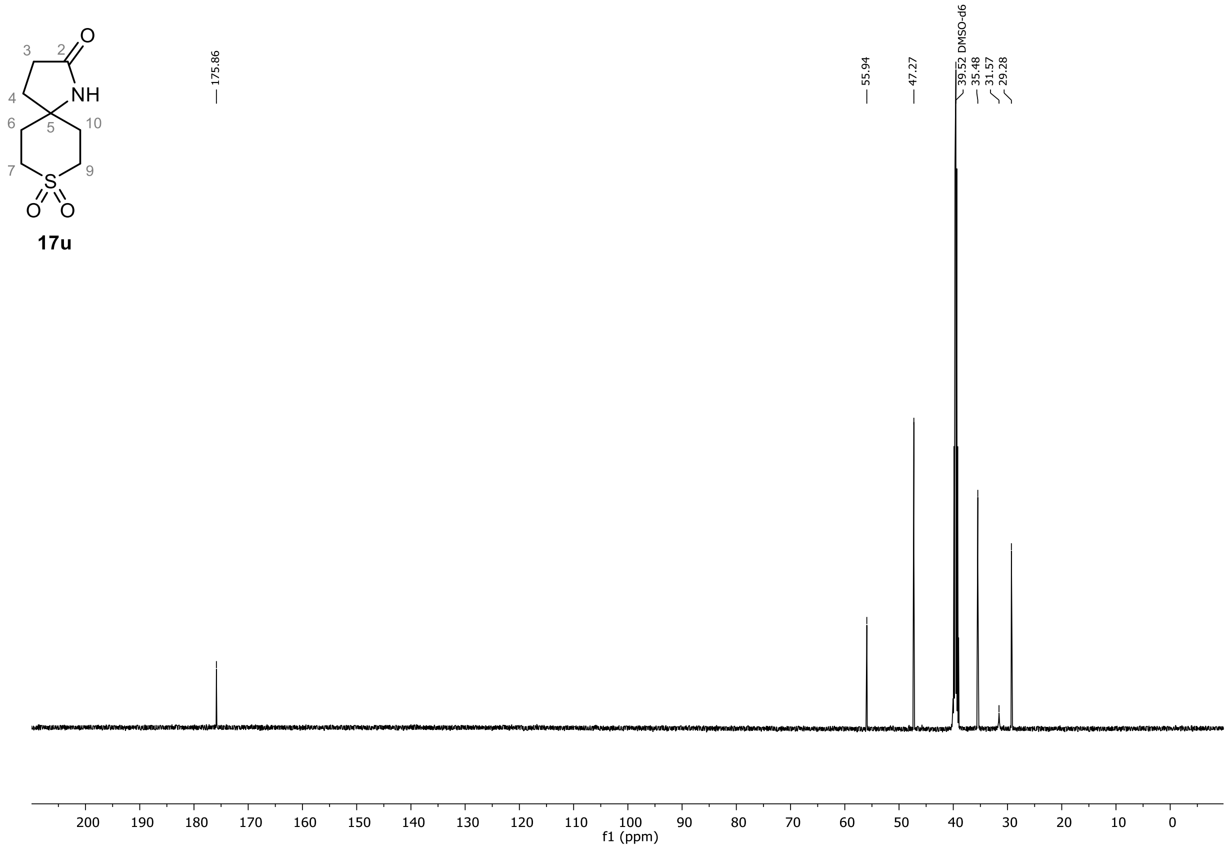

17u

^{1}H NMR (500 MHz, $CDCl_3$)

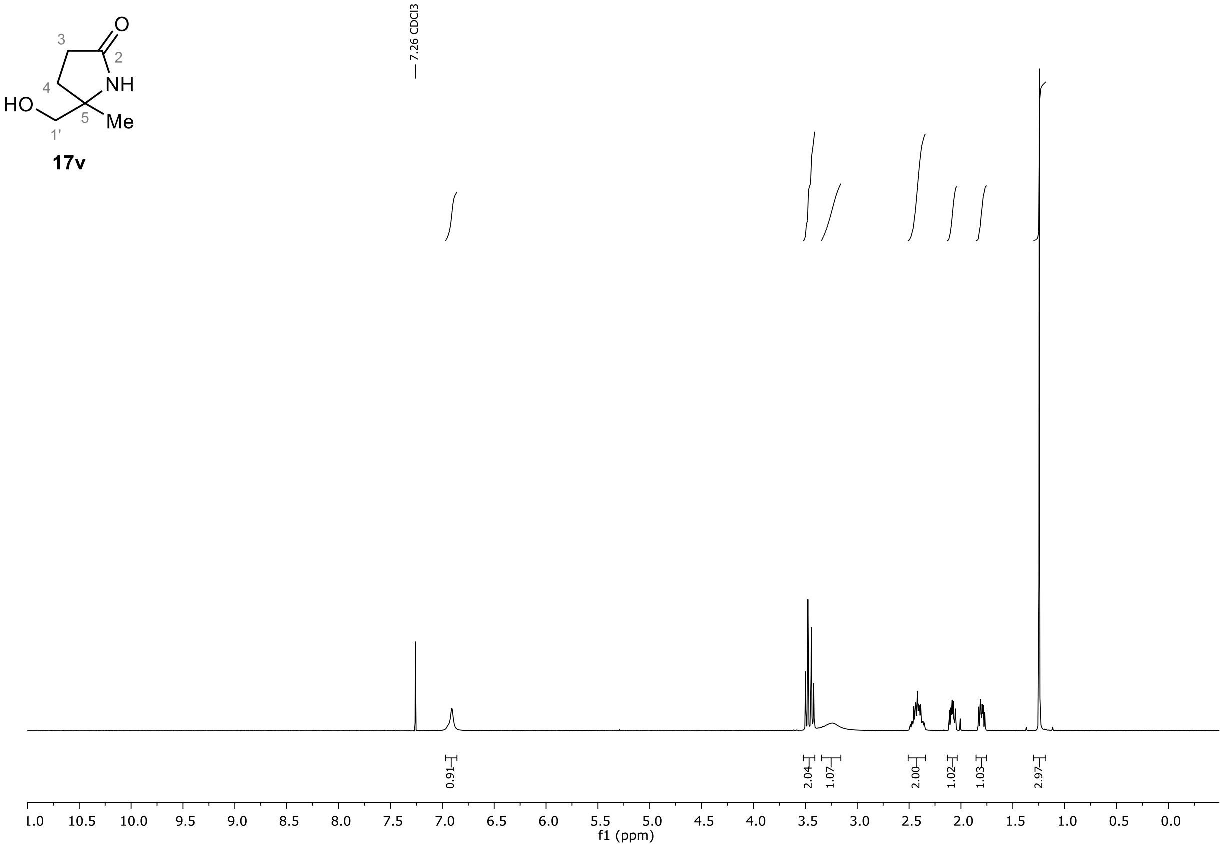

17v

$^{13}C\{^1H\}$ NMR (126 MHz, CDCl$_3$)

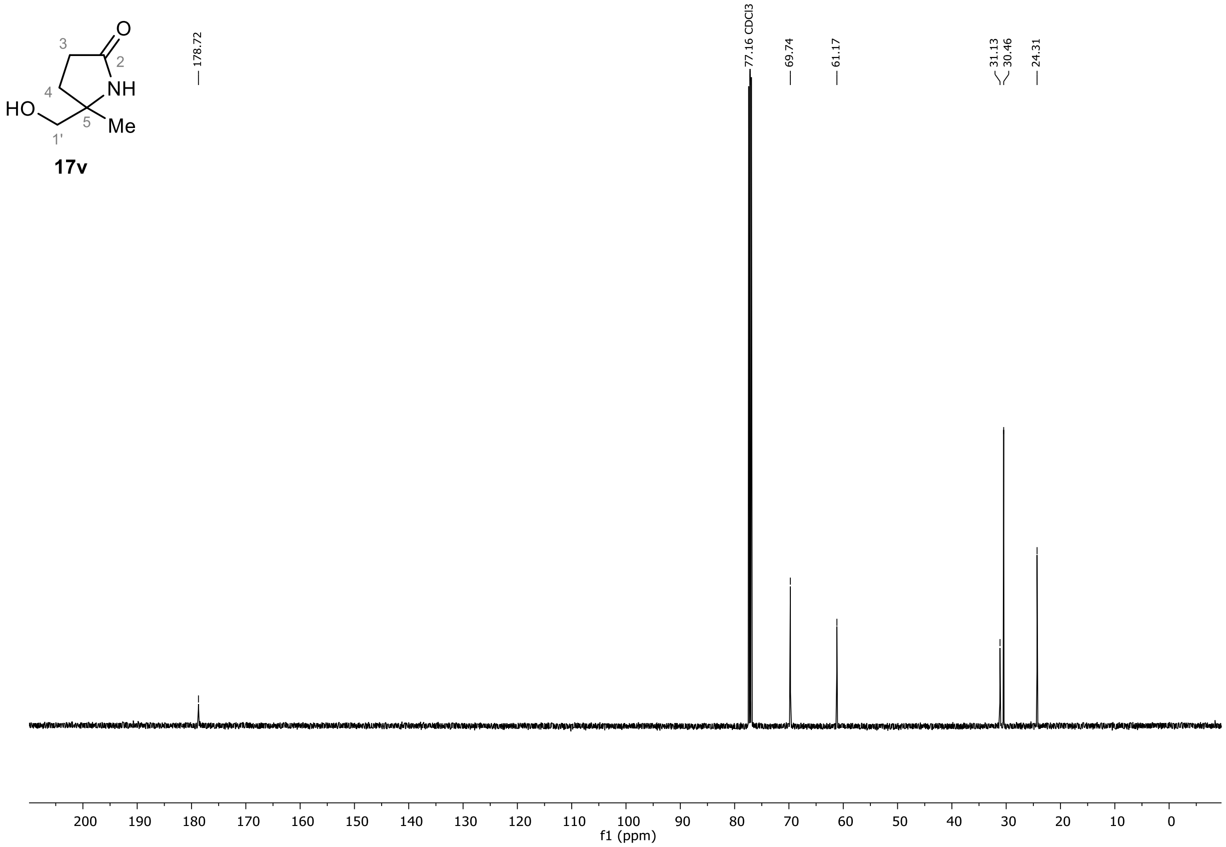

^1H NMR (500 MHz, CDCl$_3$)

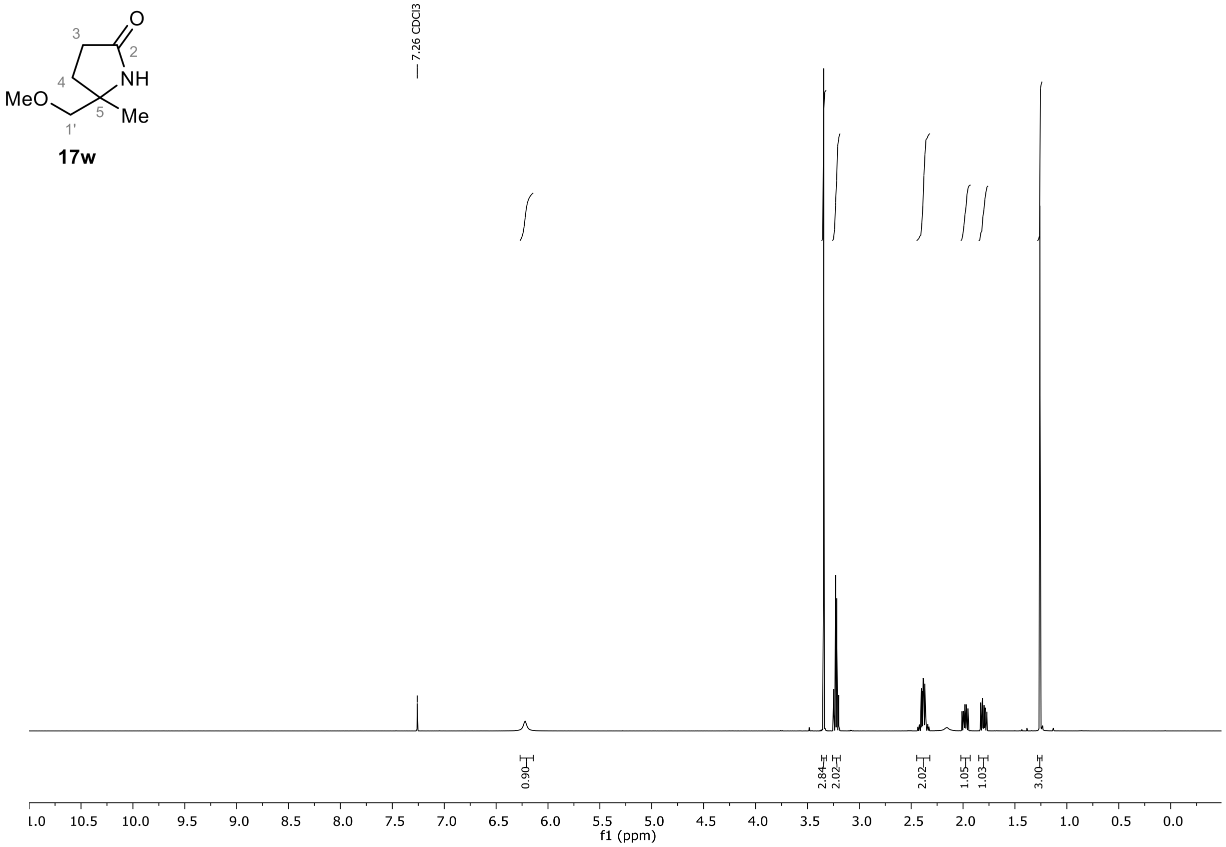

$^{13}C\{^1H\}$ NMR (126 MHz, $CDCl_3$)

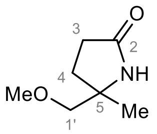

17w

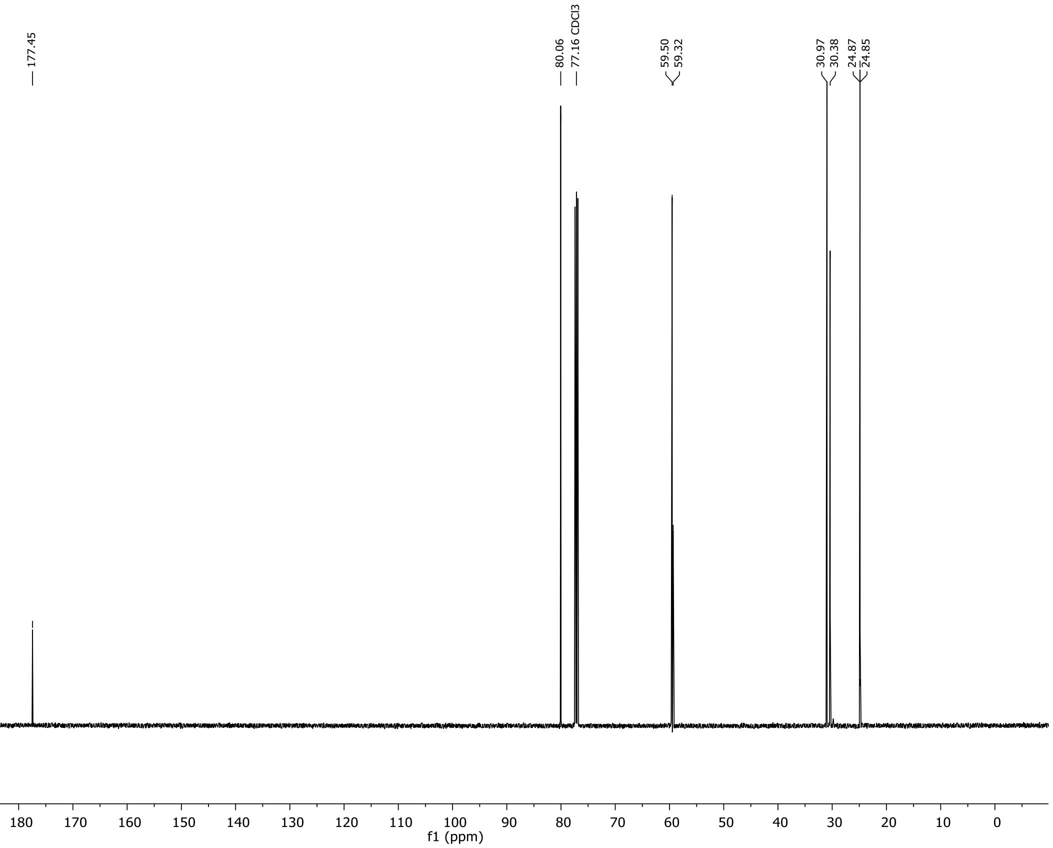

^1H NMR (500 MHz, $CDCl_3$)

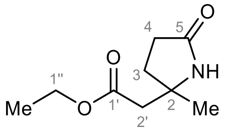

17x

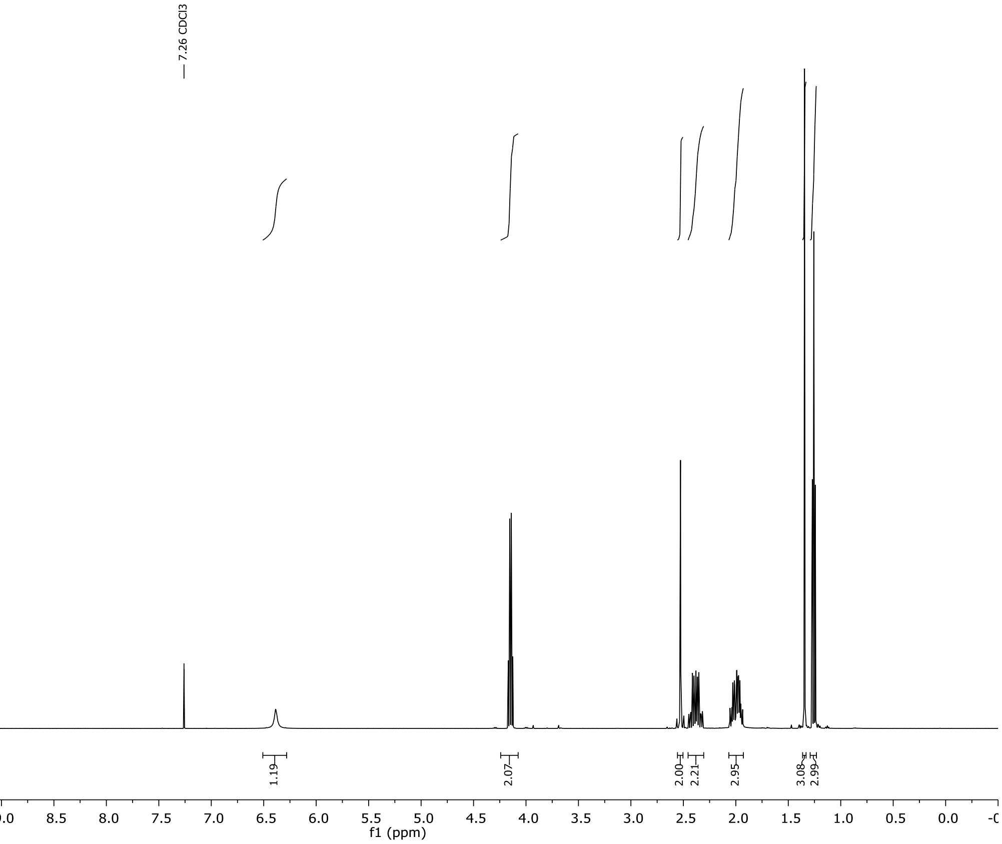

$^{13}C\{^1H\}$ NMR (126 MHz, $CDCl_3$)

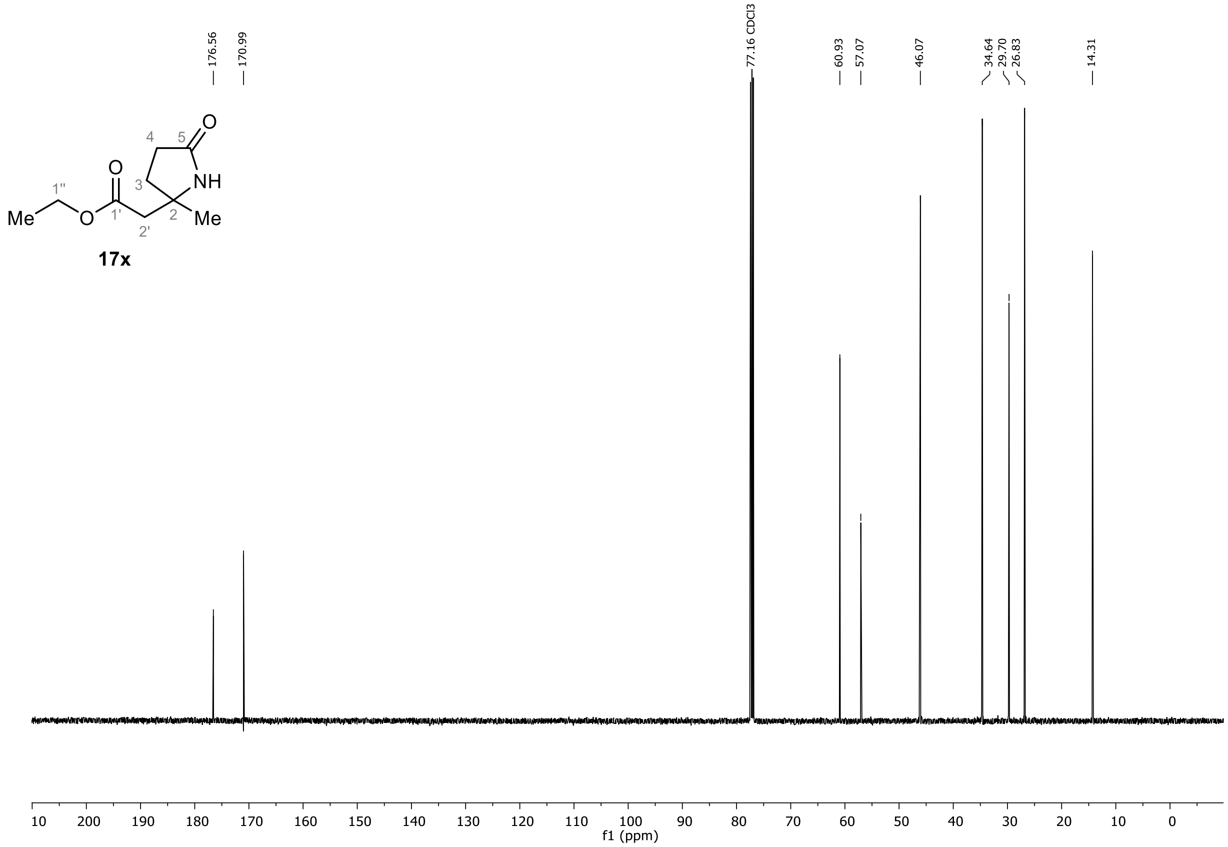

17x

^1H NMR (500 MHz, $CDCl_3$)

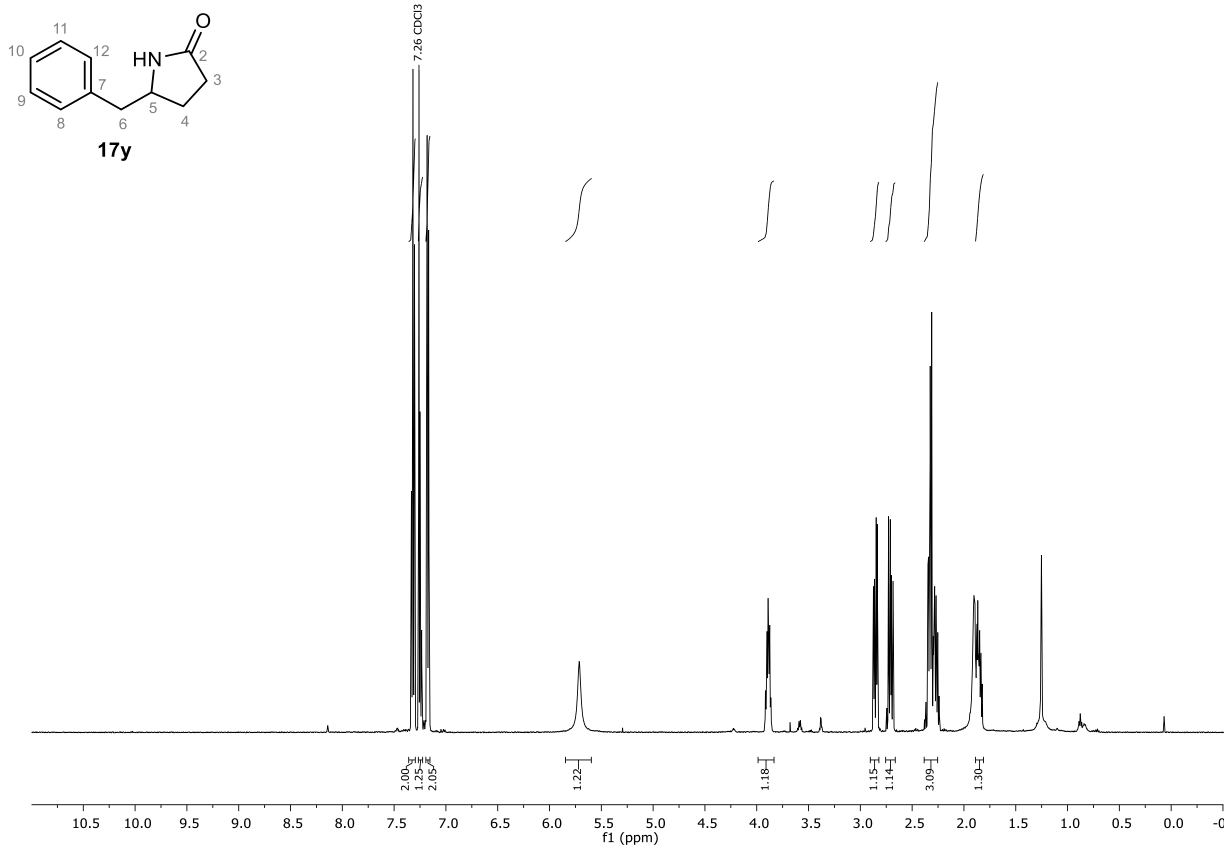

17y

$^{13}C\{^{1}H\}$ NMR (126 MHz, $CDCl_3$)

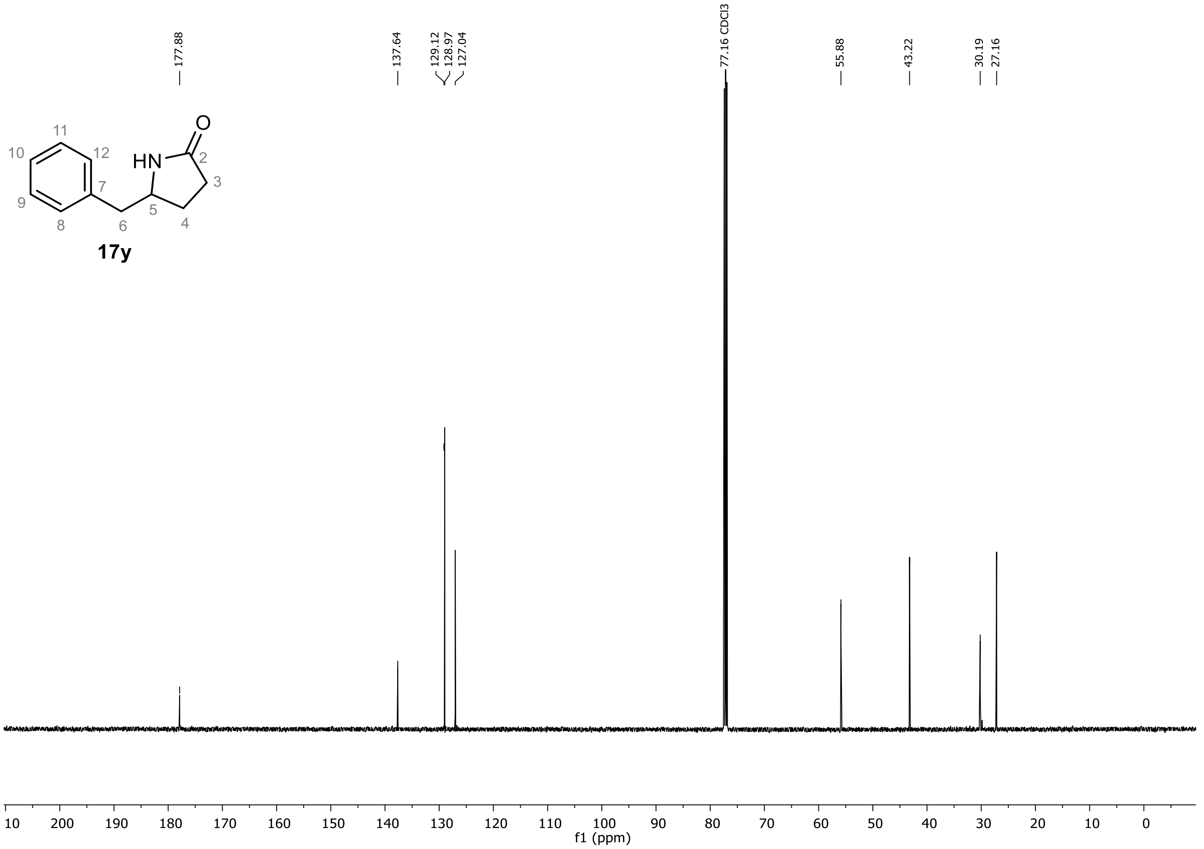

^{1}H NMR (500 MHz, $CDCl_3$)

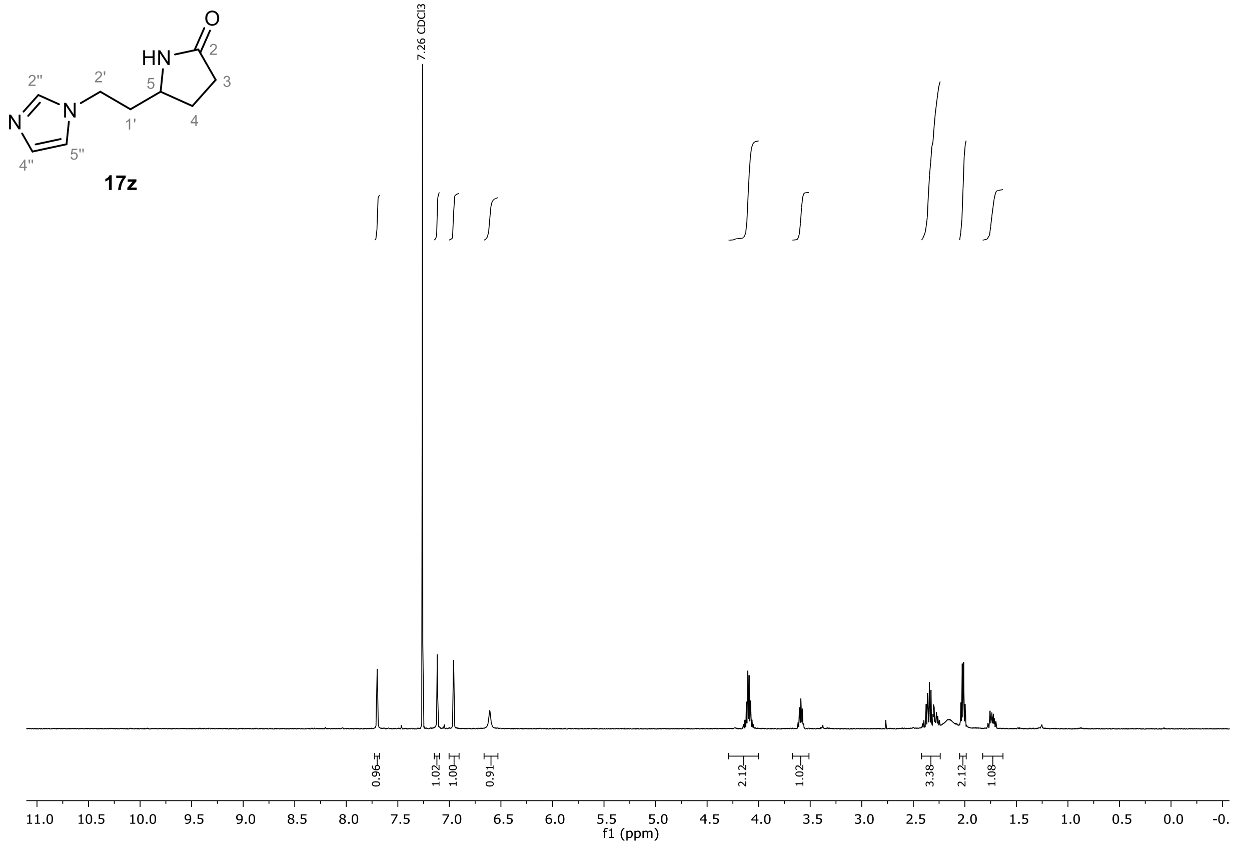

$^{13}C\{^1H\}$ NMR (126 MHz, $CDCl_3$)

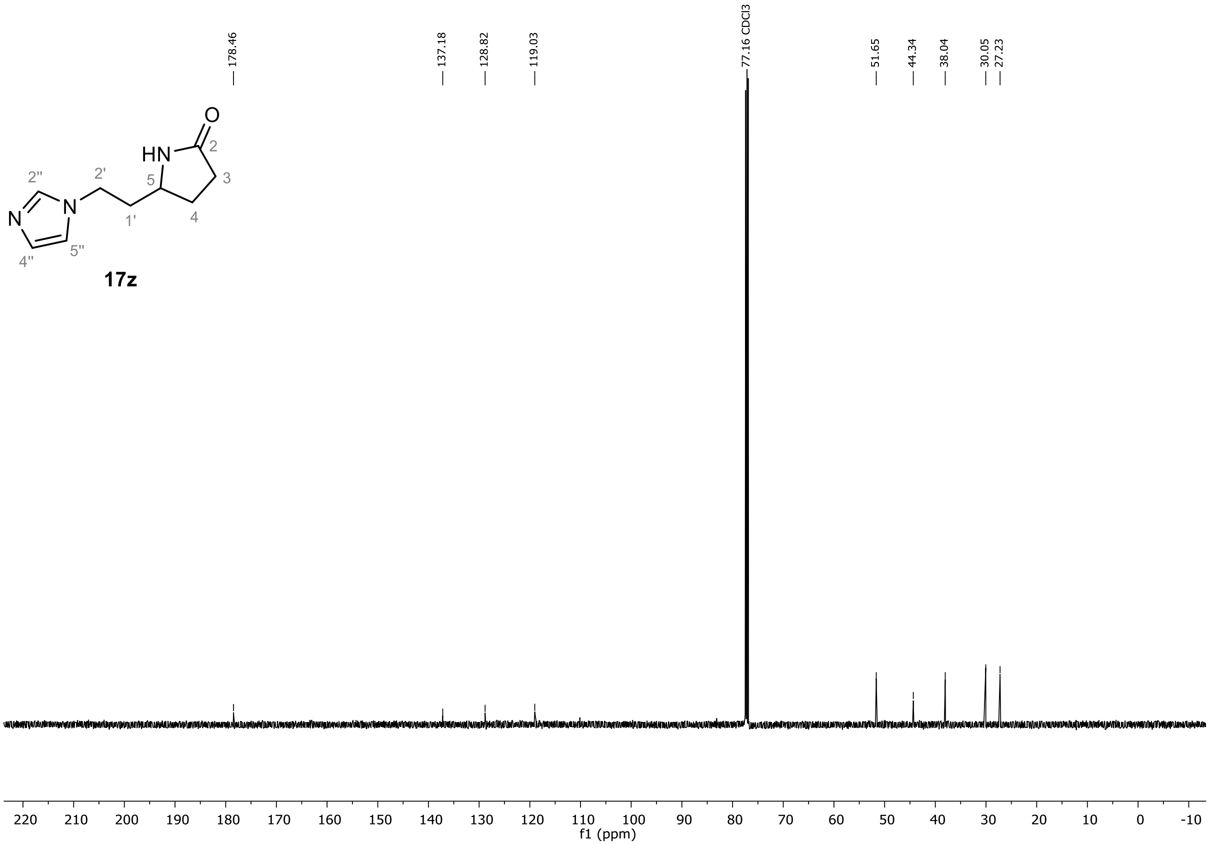

17z

^1H NMR (500 MHz, $CDCl_3$)

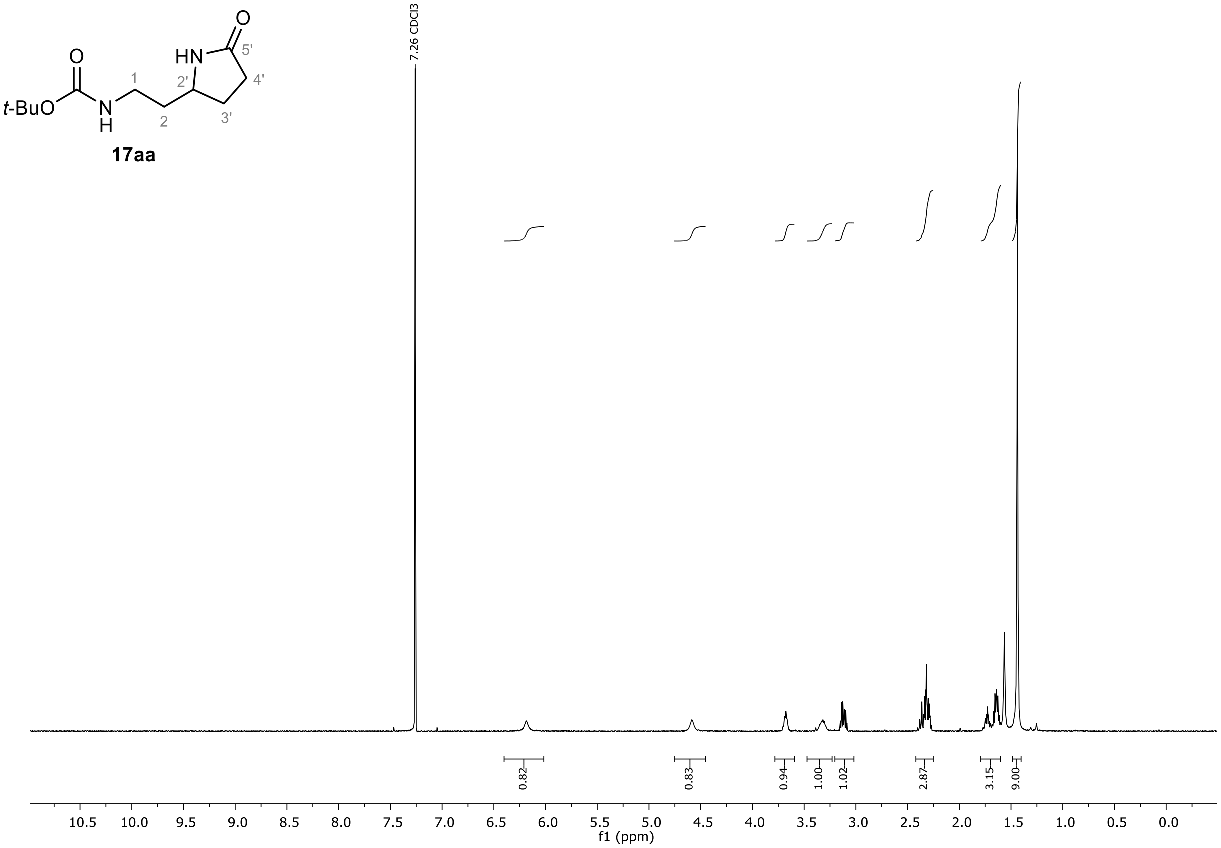

17aa

$^{13}C\{^1H\}$ NMR (126 MHz, $CDCl_3$)

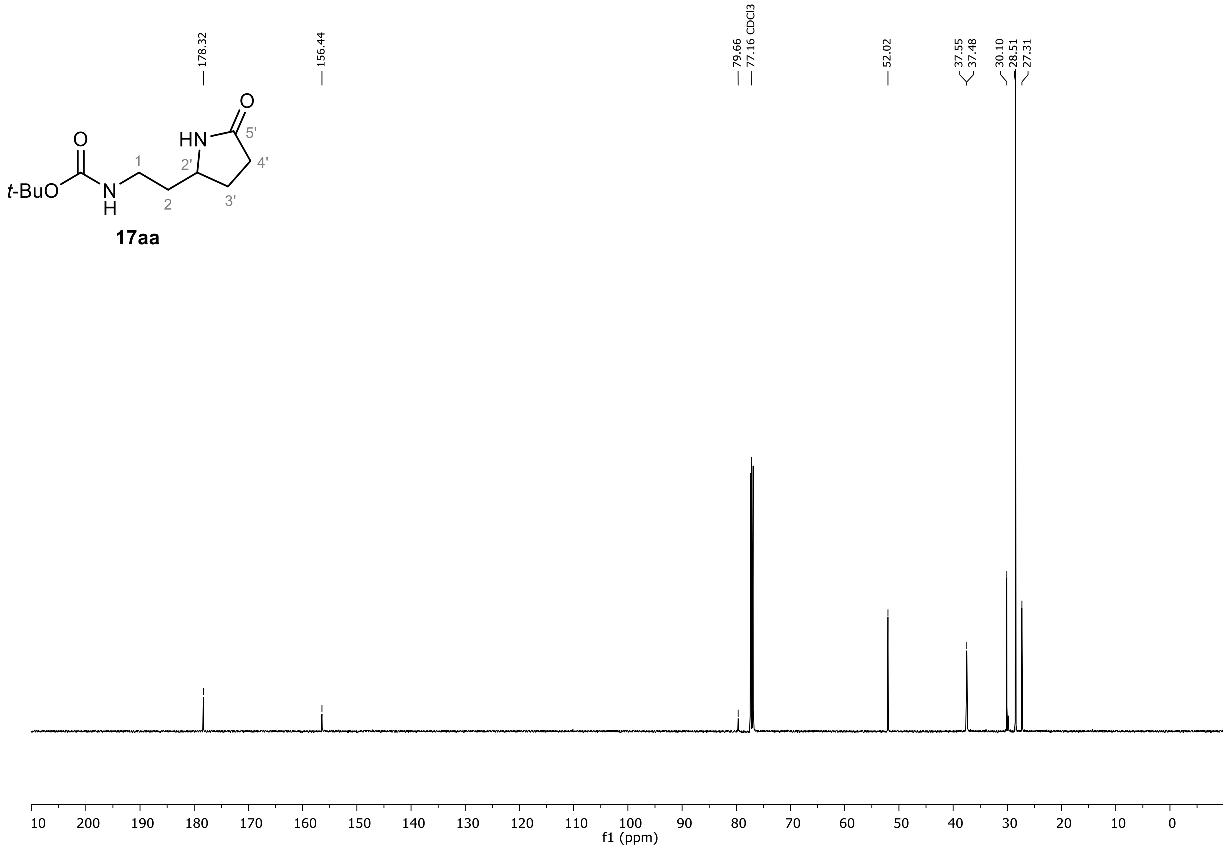

17aa

^1H NMR (500 MHz, $CDCl_3$)

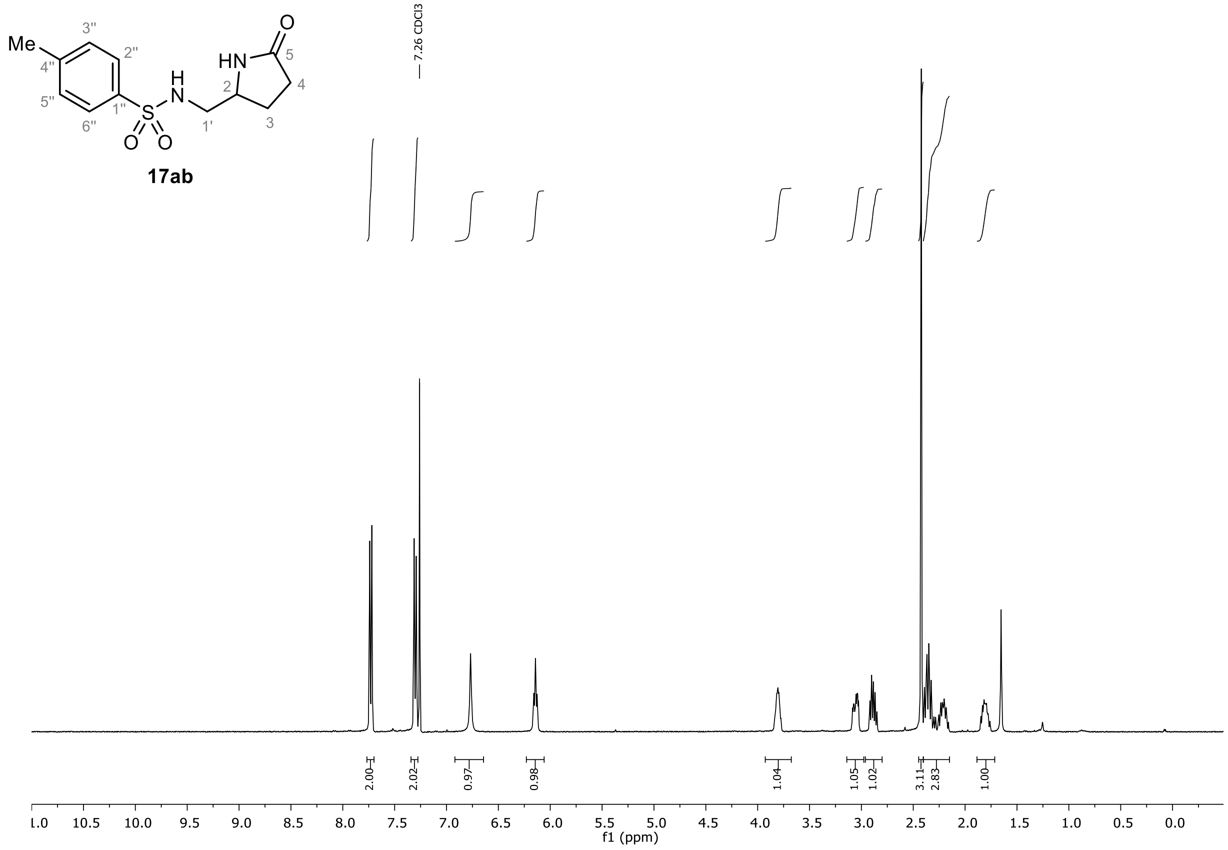

17ab

$^{13}C\{^{1}H\}$ NMR (126 MHz, $CDCl_3$)

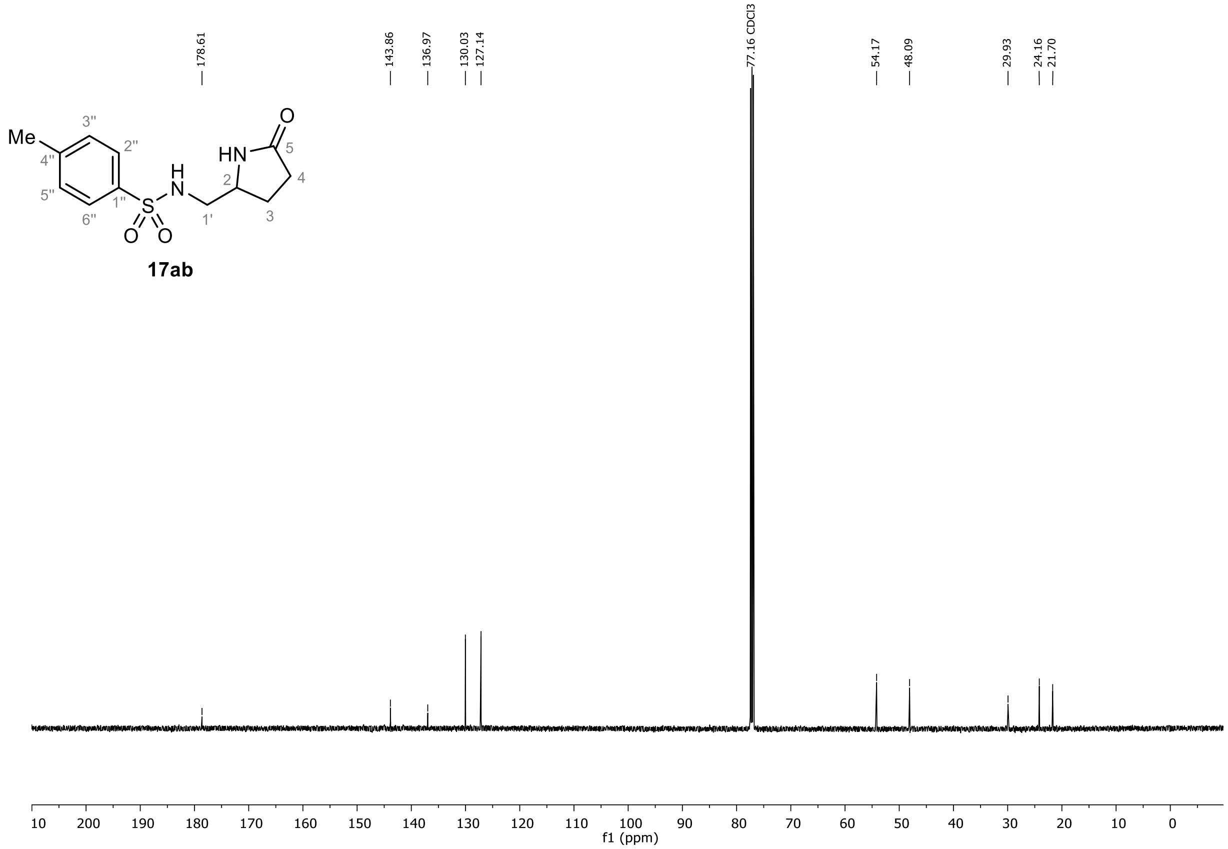

^{1}H NMR (500 MHz, $CDCl_3$)

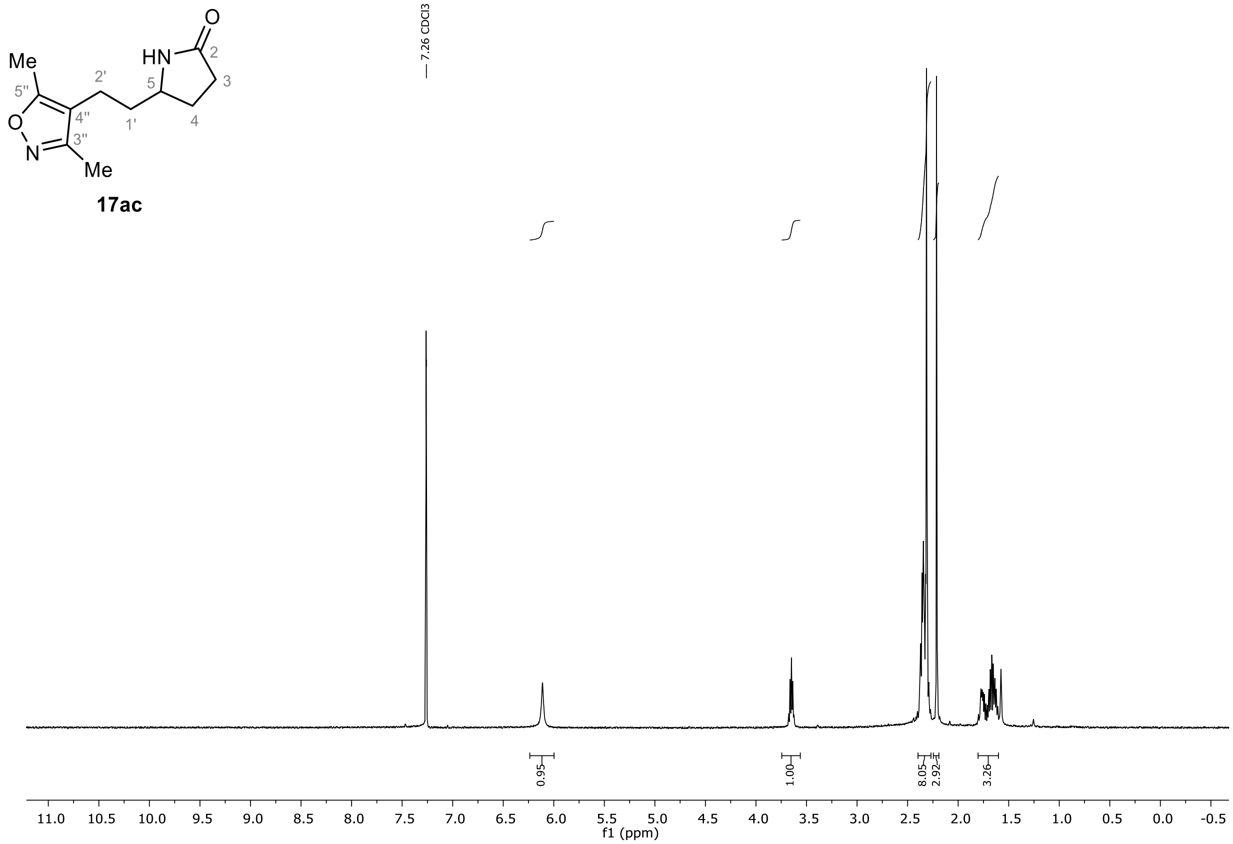

$^{13}C\{^1H\}$ NMR (126 MHz, $CDCl_3$)

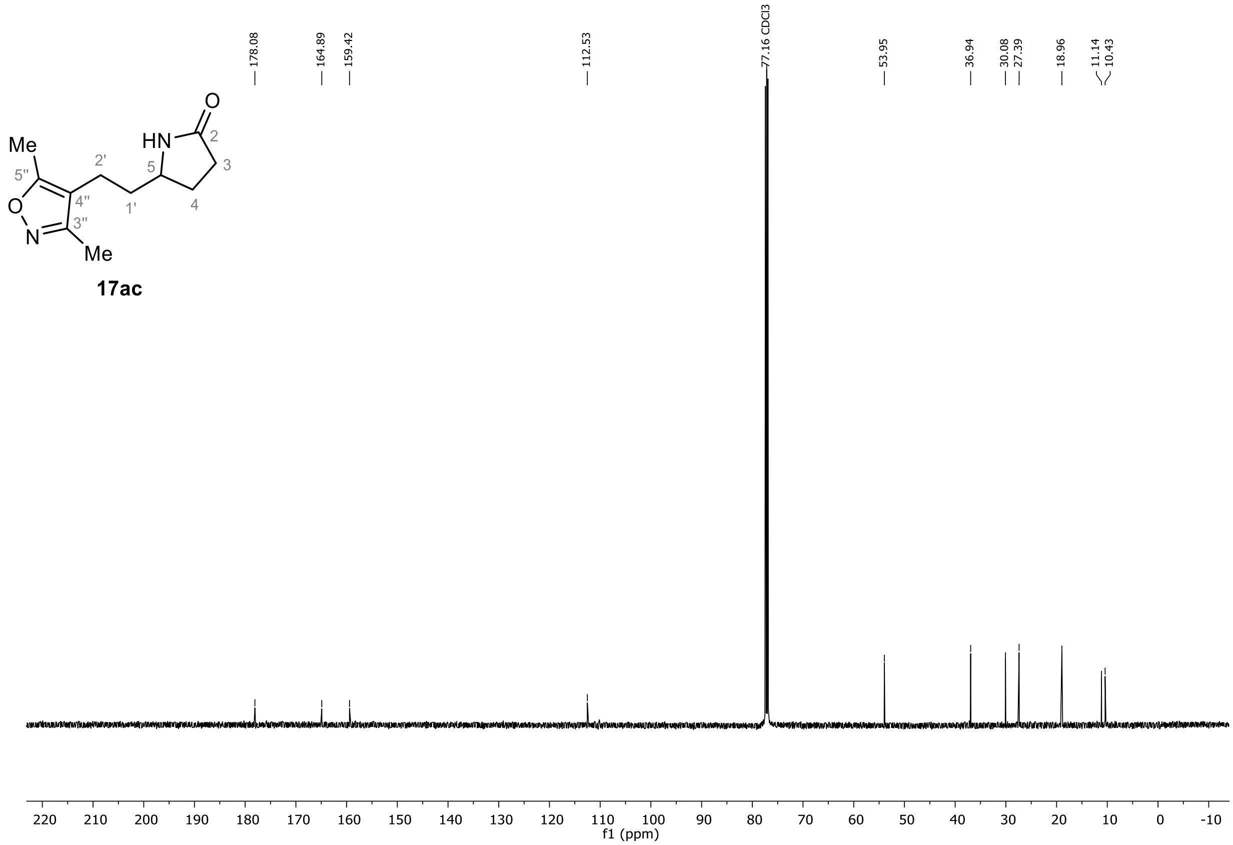

^1H NMR (500 MHz, $CDCl_3$)

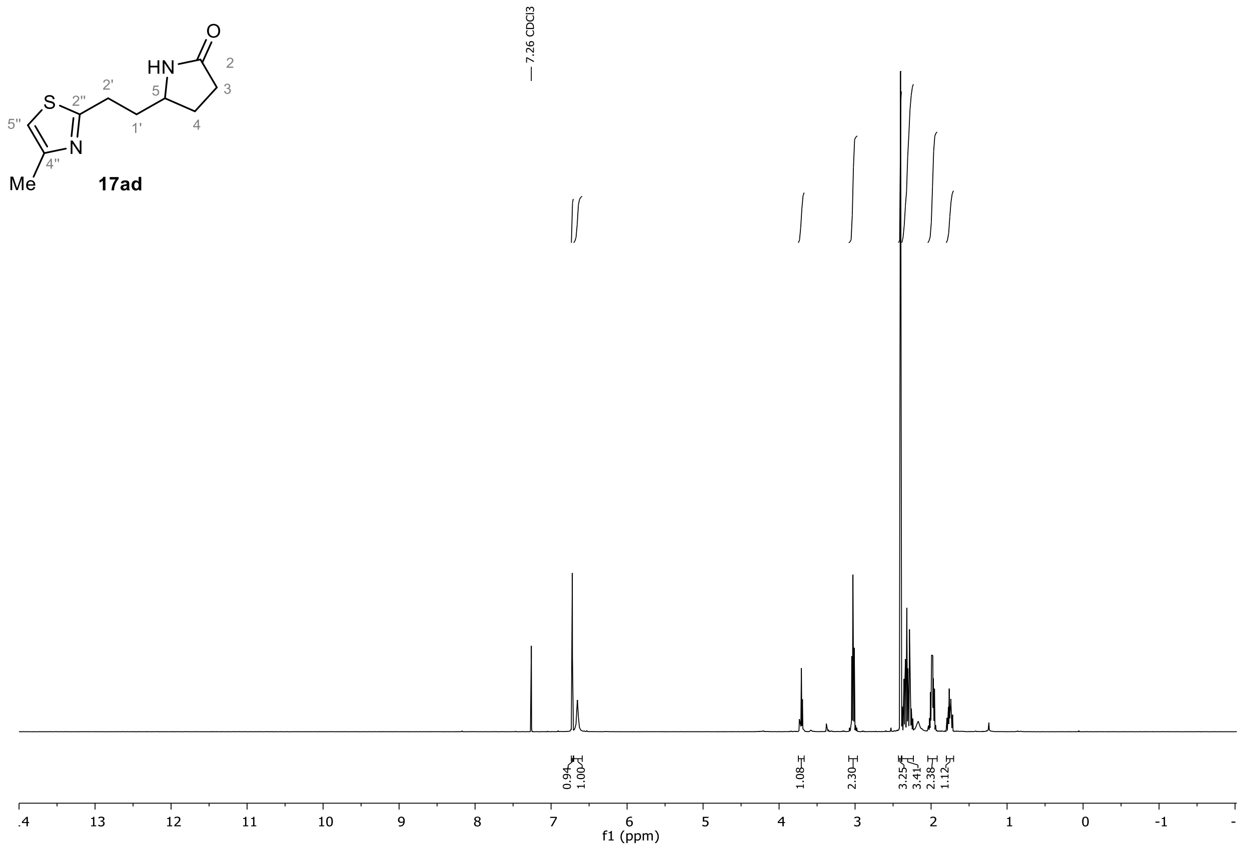

1H NMR (500 MHz, CDCl3)

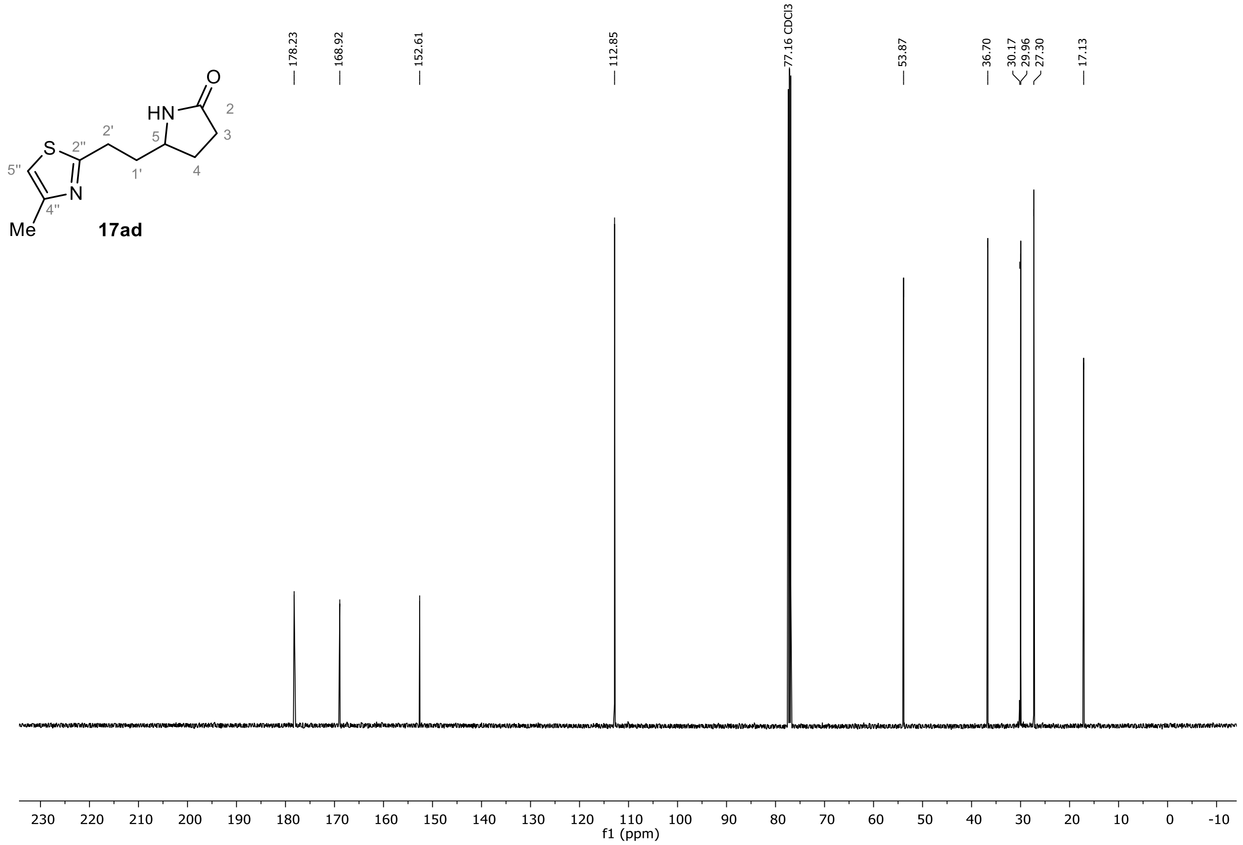

1H NMR (500 MHz, CDCl3)

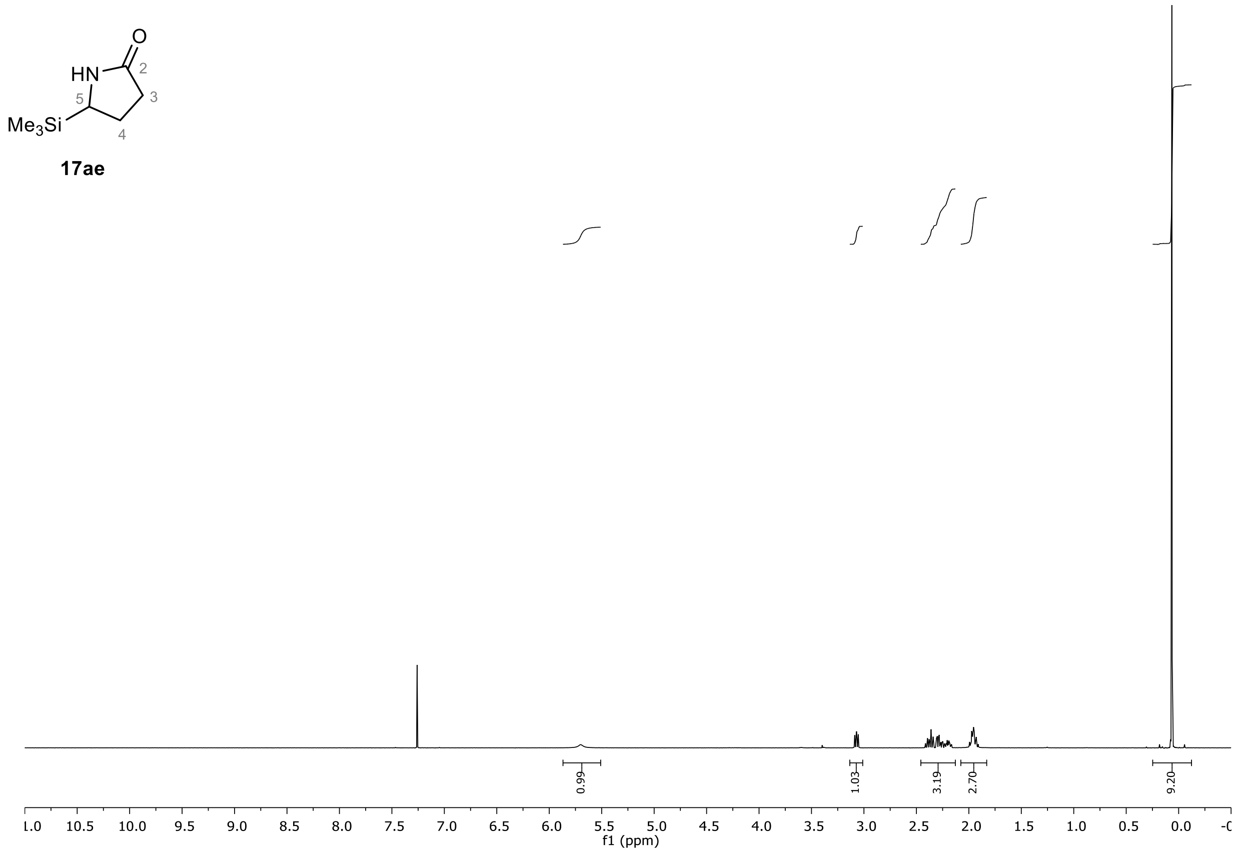

$^{13}C\{^1H\}$ NMR (126 MHz, CDCl$_3$)

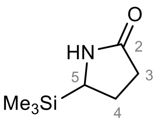

17ae

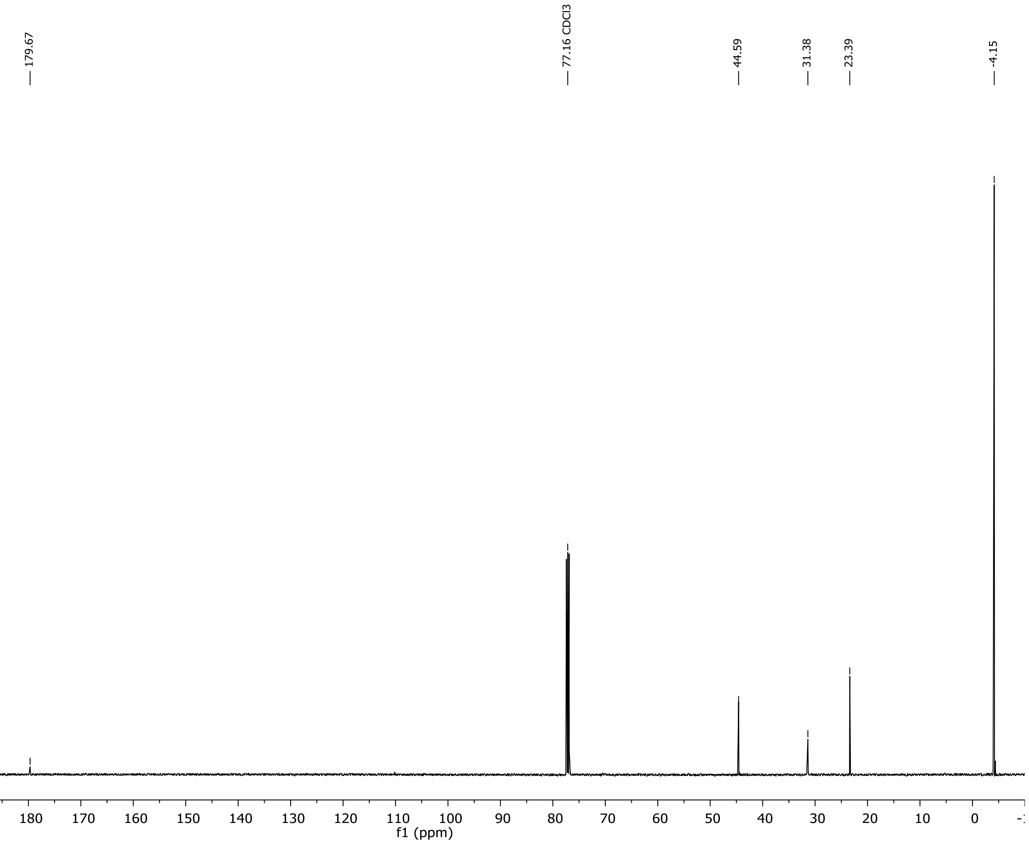

^{1}H NMR (500 MHz, $CDCl_3$)

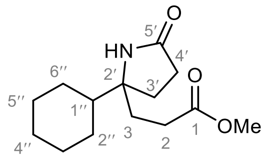

20f

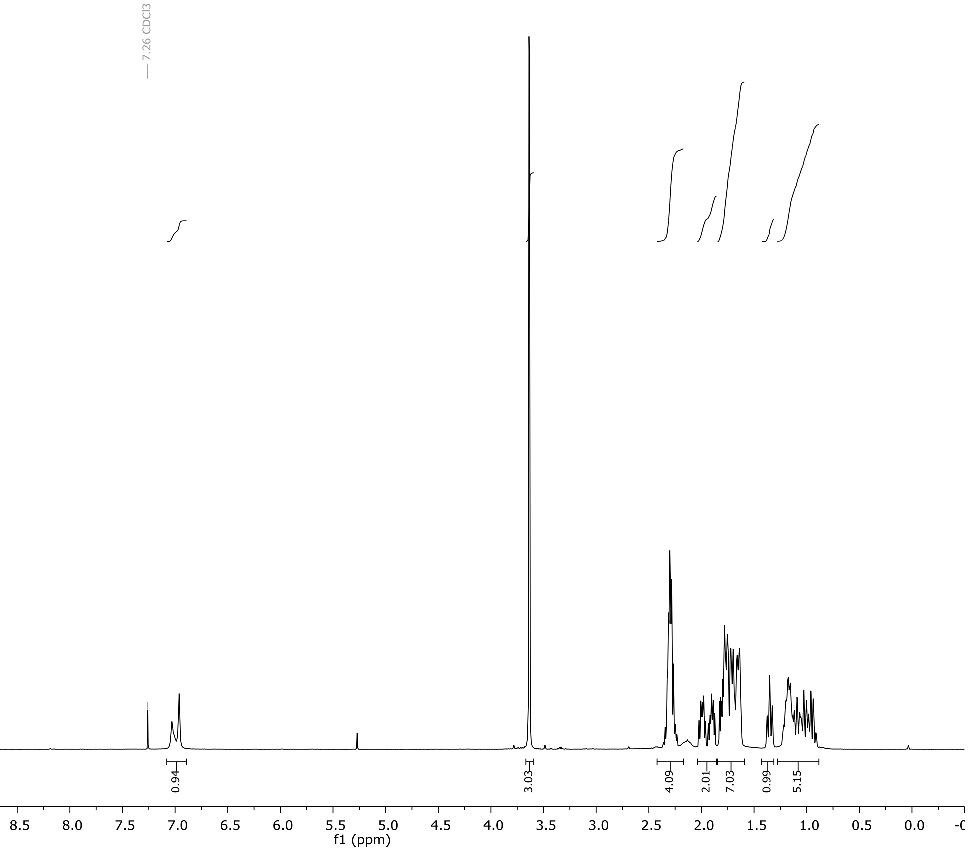

$^{13}C\{^{1}H\}$ NMR (126 MHz, $CDCl_3$)

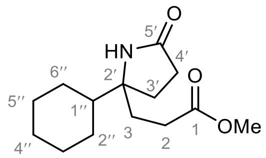
HN
O
O
OMe
5′
4′
3′
2′
1
2
3
1″
2″
3″
4″
5″
6″

20f

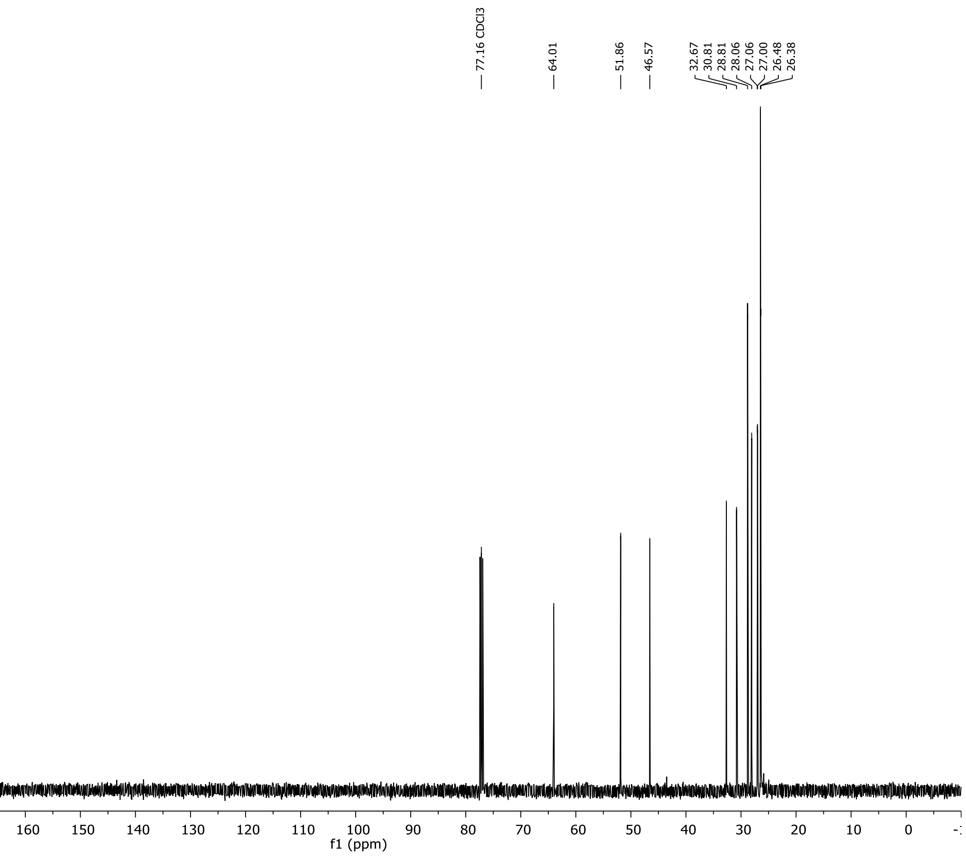
177.83
174.02
77.16 CDCl3
64.01
51.86
46.57
32.67
30.81
28.81
28.06
27.06
27.00
26.48
26.38
f1 (ppm)

^{1}H NMR (500 MHz, $CDCl_3$)

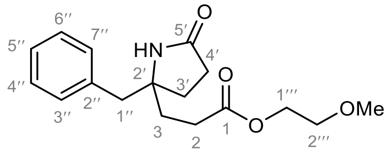

20y

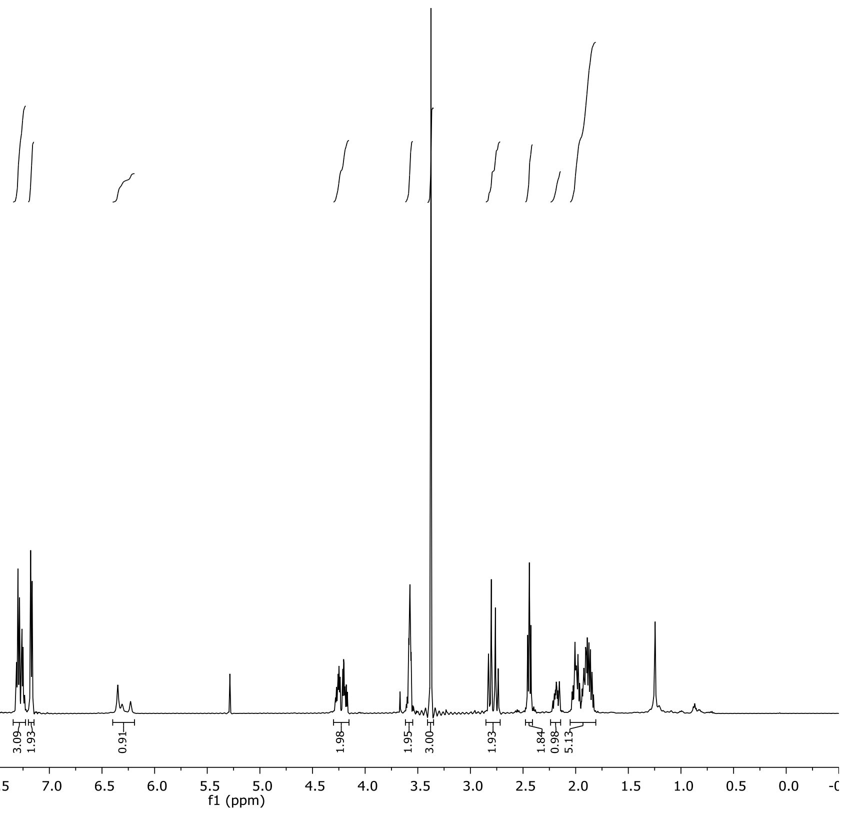

^{13}C{^{1}H} NMR (126 MHz, $CDCl_3$)

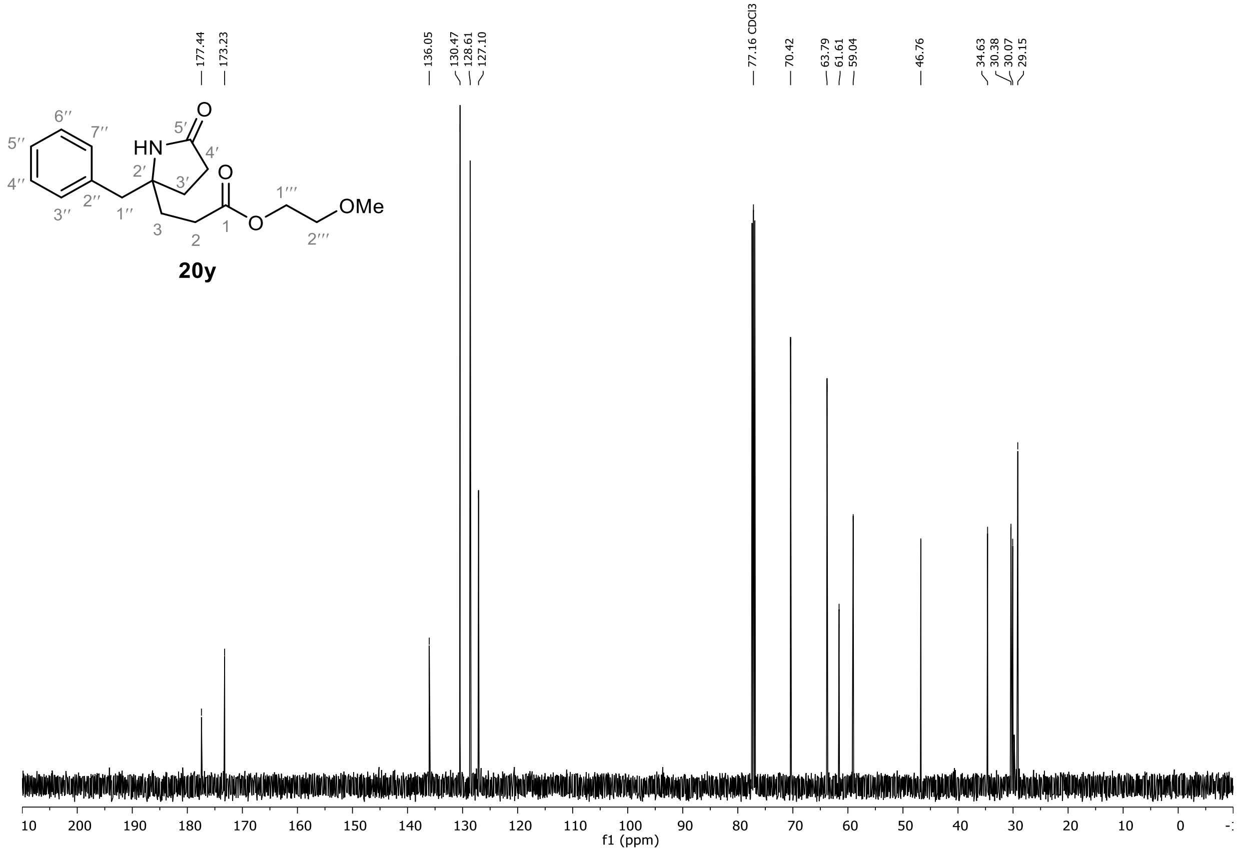
177.44
173.23
136.05
130.47
128.61
127.10
77.16 CDCl3
70.42
63.79
61.61
59.04
46.76
34.63
30.38
30.07
29.15
HN
O
OMe
20y
f1 (ppm)

^{1}H NMR (500 MHz, $CDCl_3$)

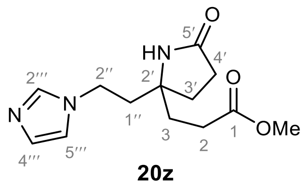

20z

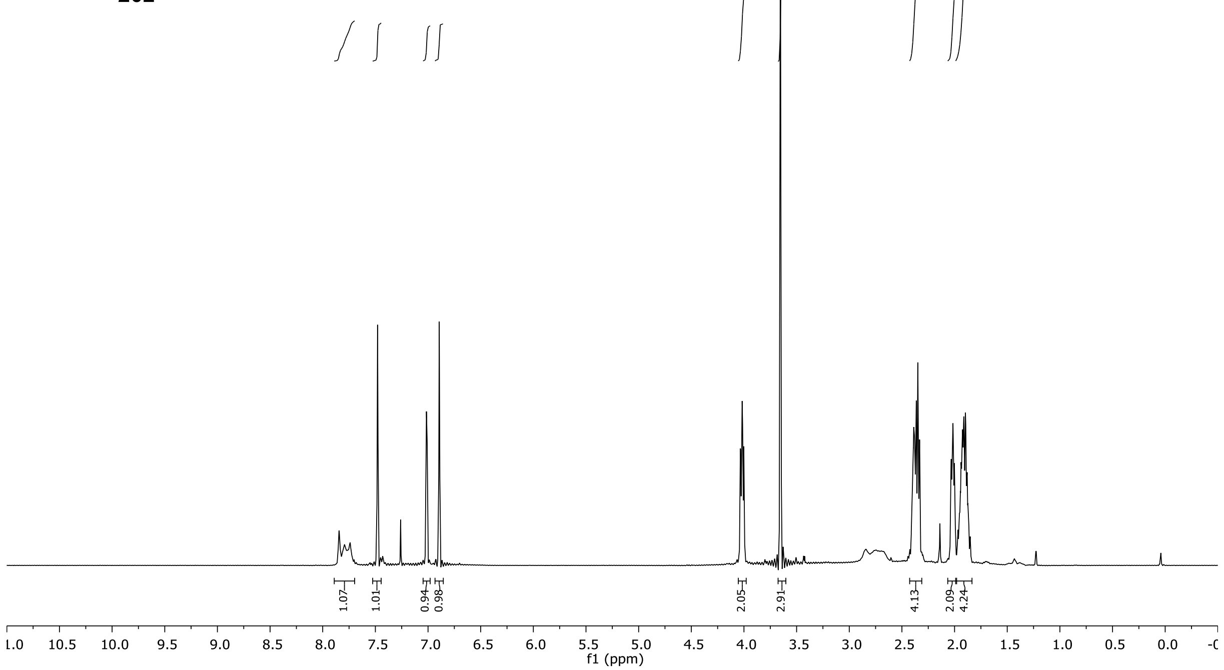

^{13}C{^{1}H} NMR (126 MHz, $CDCl_3$)

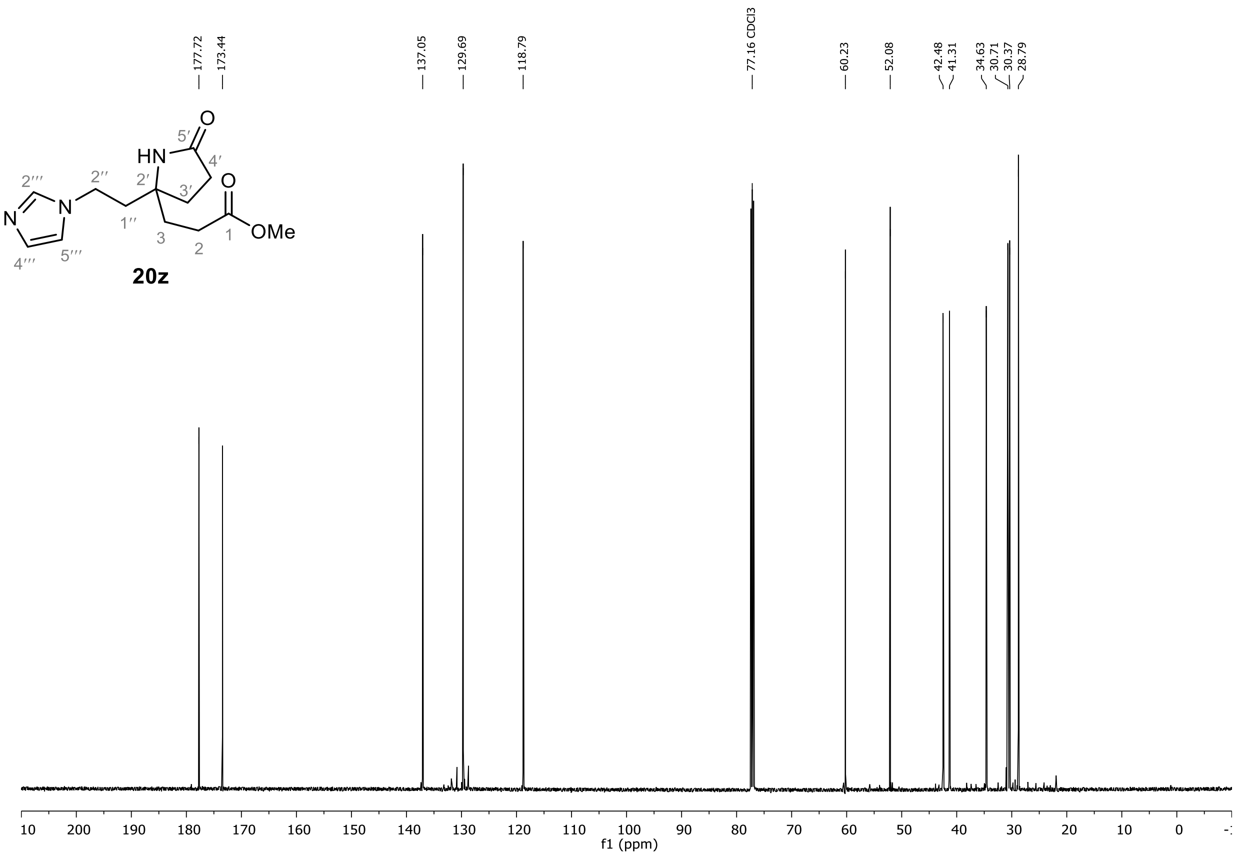
177.72
173.44
137.05
129.69
118.79
77.16 CDCl3
60.23
52.08
42.48
41.31
34.63
30.71
30.37
28.79
HN
O
OMe
N
20z
f1 (ppm)

^{1}H NMR (500 MHz, $CDCl_3$)

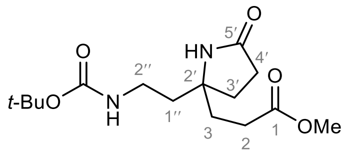

20aa

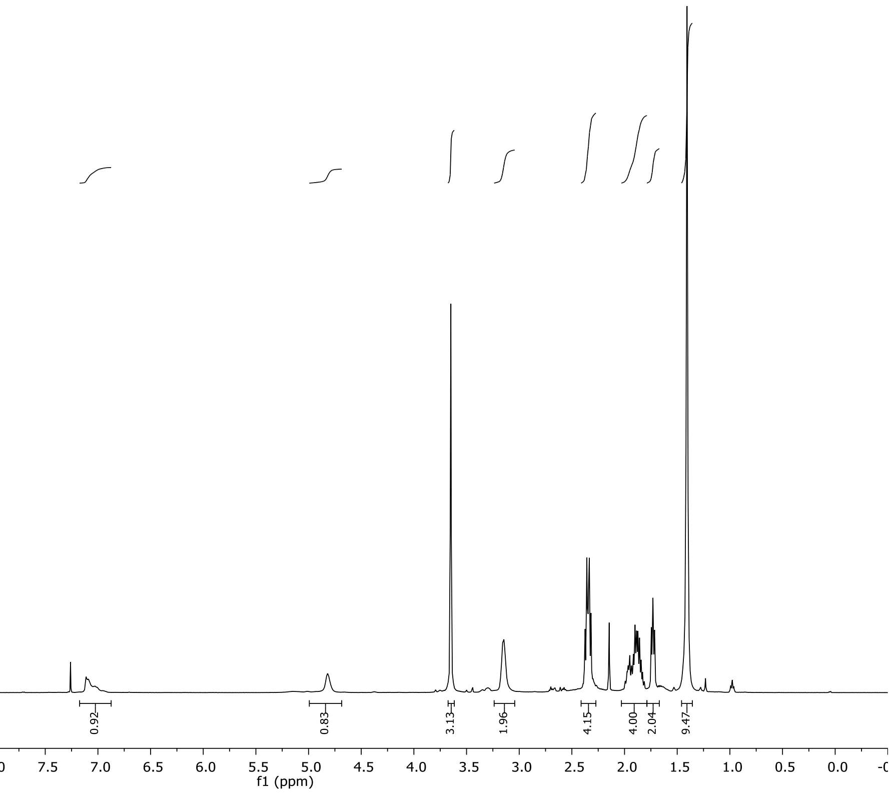

$^{13}C\{^{1}H\}$ NMR (126 MHz, $CDCl_3$)

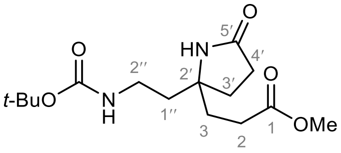
O
HN
5′
4′
2′′
2′
3′
O
t-BuO
N
H
1′′
3
2
1
OMe

20aa

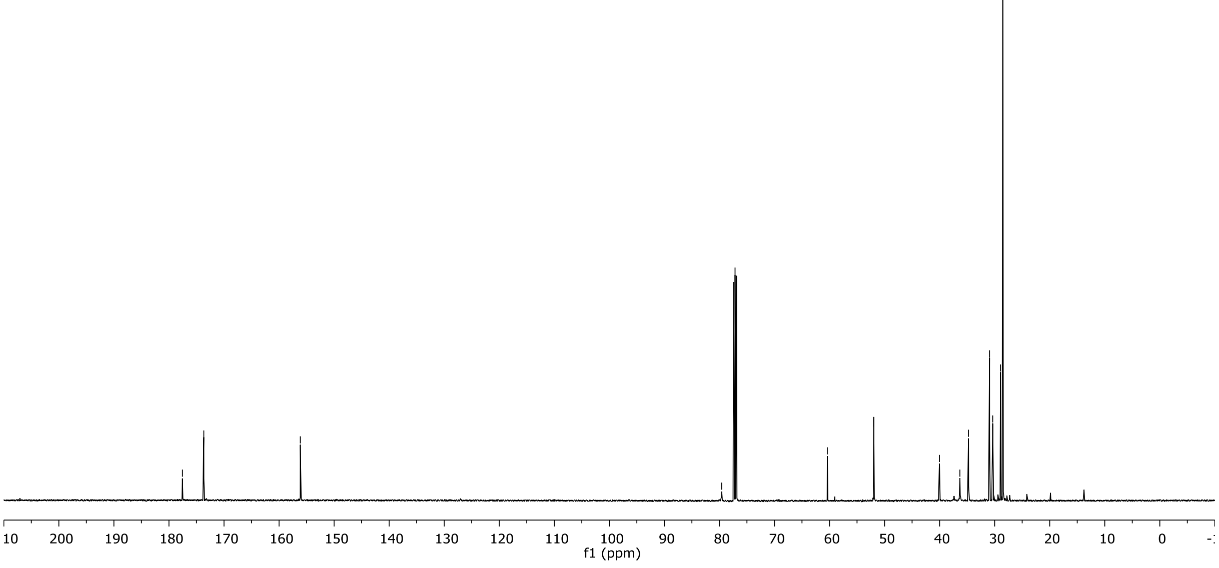
177.55
173.66
156.14
79.59
77.16 CDCl3
60.40
51.98
40.03
36.32
34.76
30.93
30.35
28.94
28.51
f1 (ppm)

^{1}H NMR (500 MHz, $CDCl_3$)

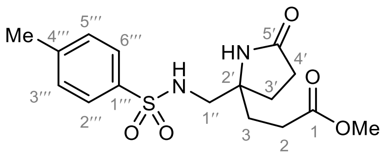

20ab

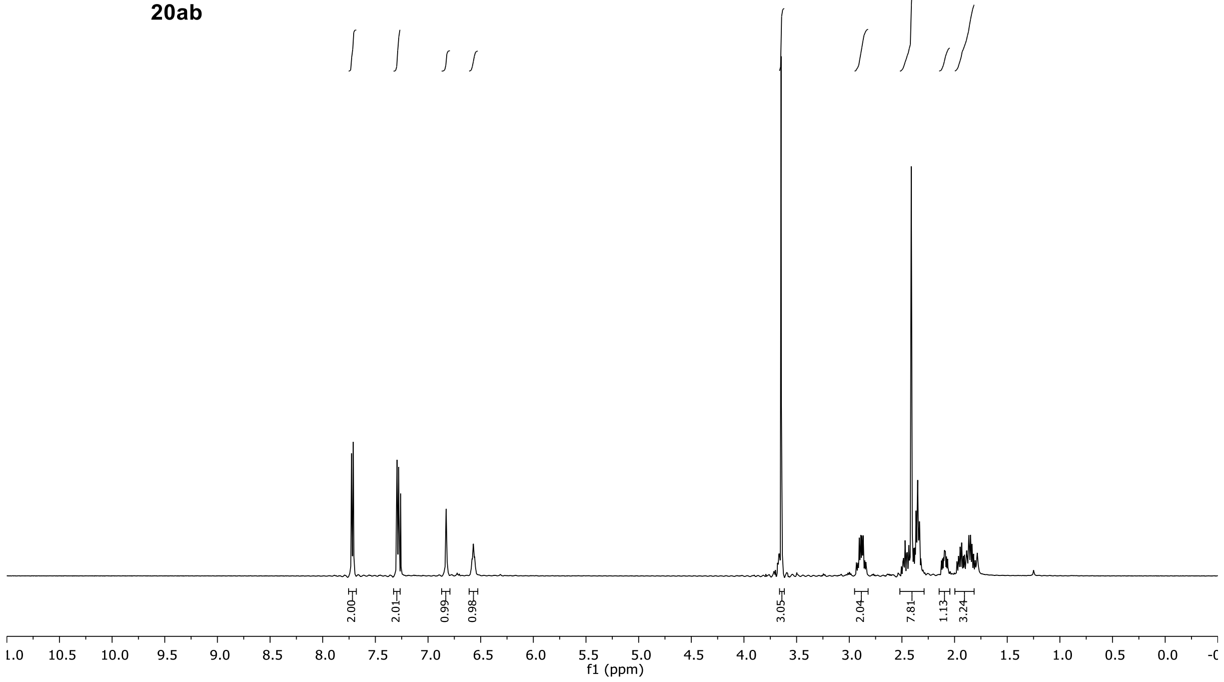

$^{13}C\{^{1}H\}$ NMR (126 MHz, $CDCl_3$)

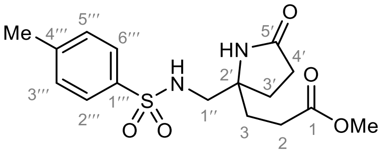

20ab

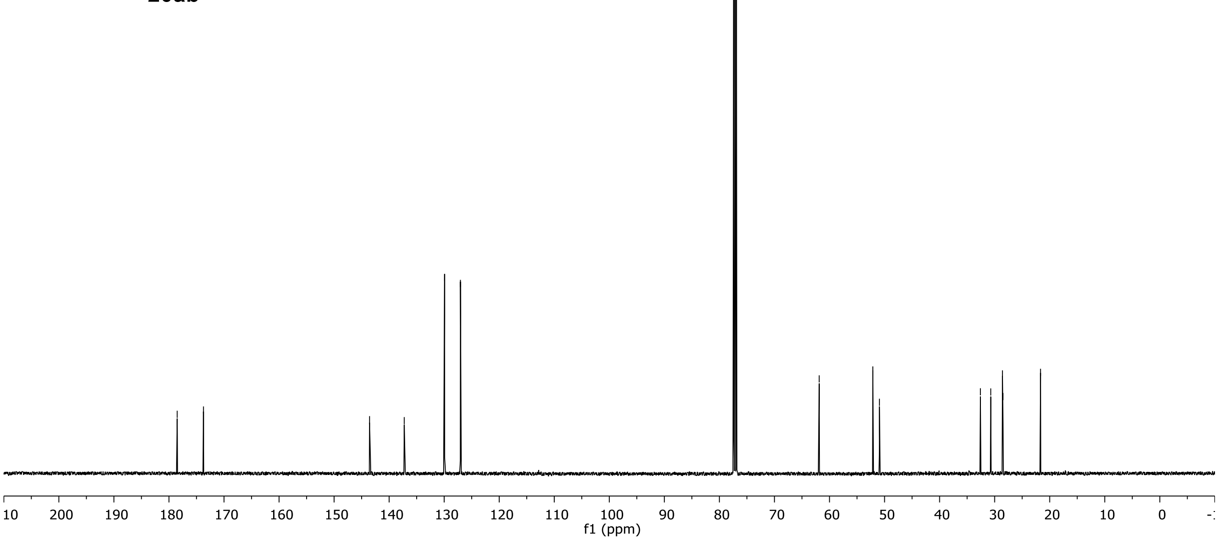

^{1}H NMR (500 MHz, CDCl$_3$)

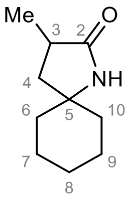

23a

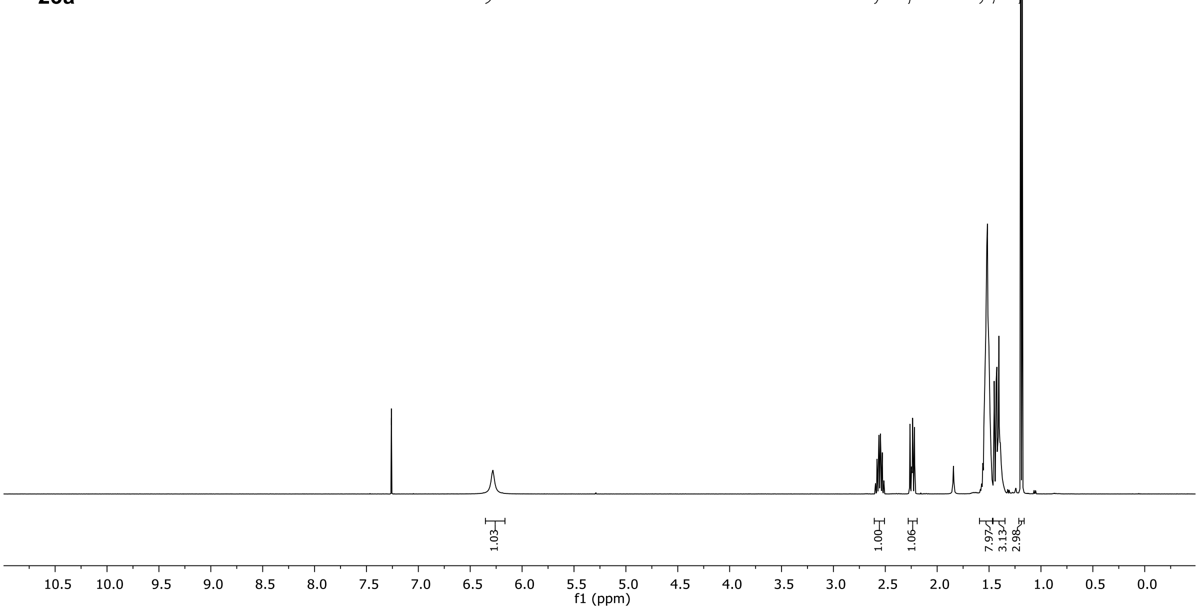

^{13}C{^{1}H} NMR (126 MHz, CDCl$_3$)

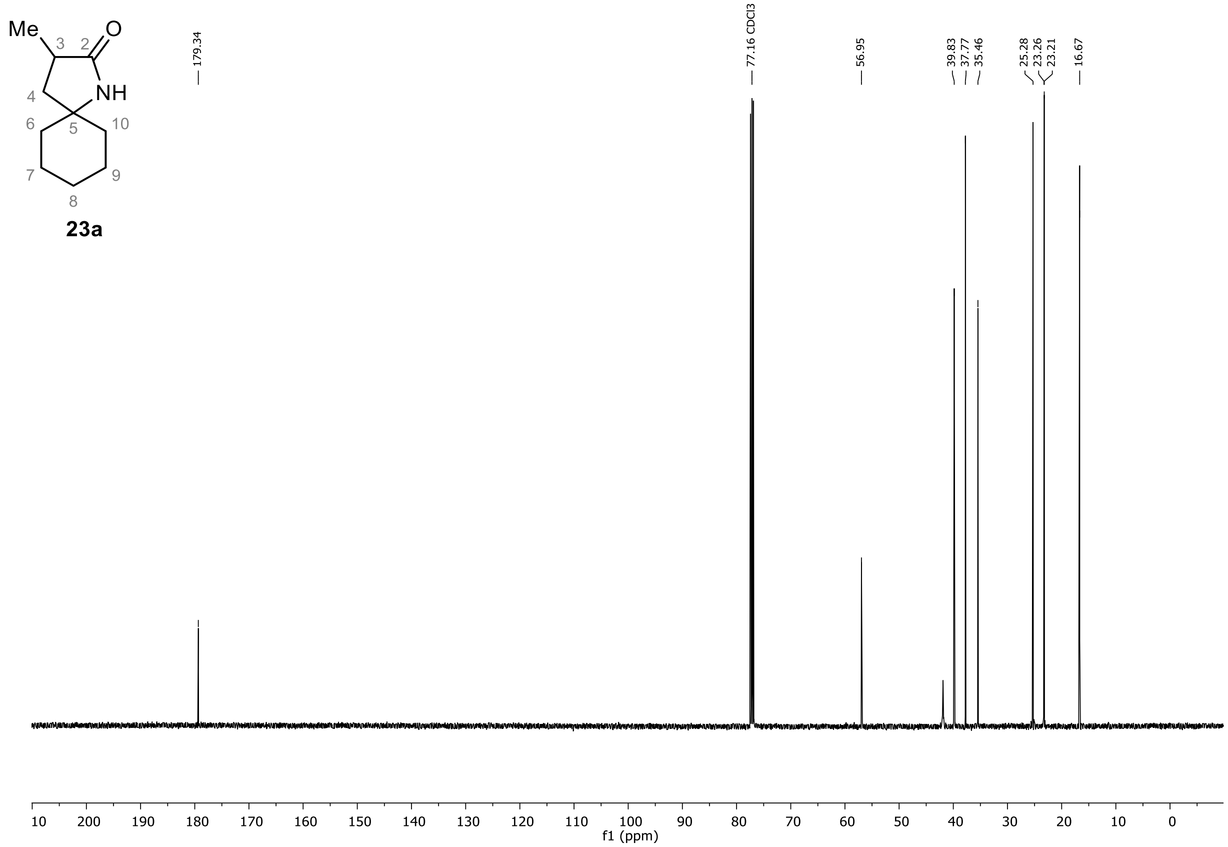

^{1}H NMR (500 MHz, CDCl3)

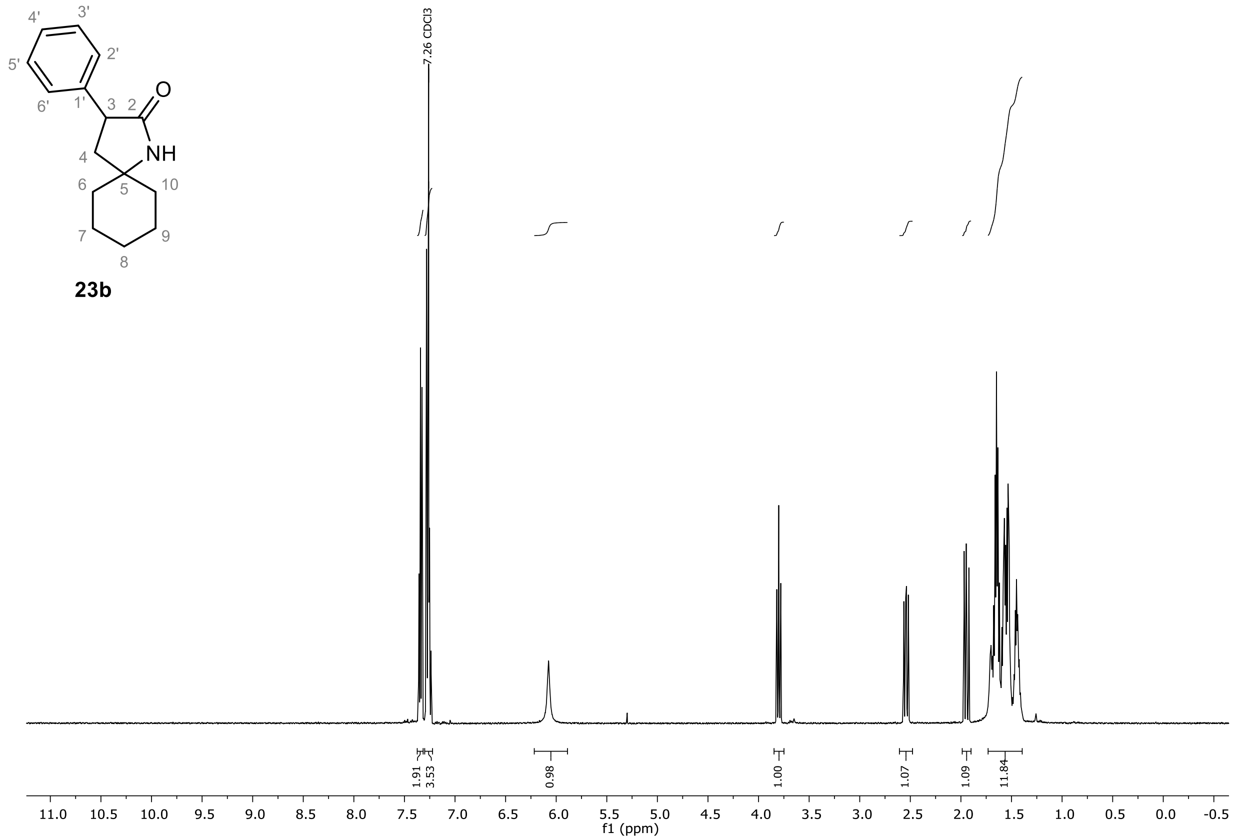

^{13}C{^{1}H} NMR (126 MHz, CDCl3)

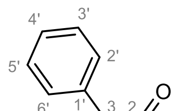

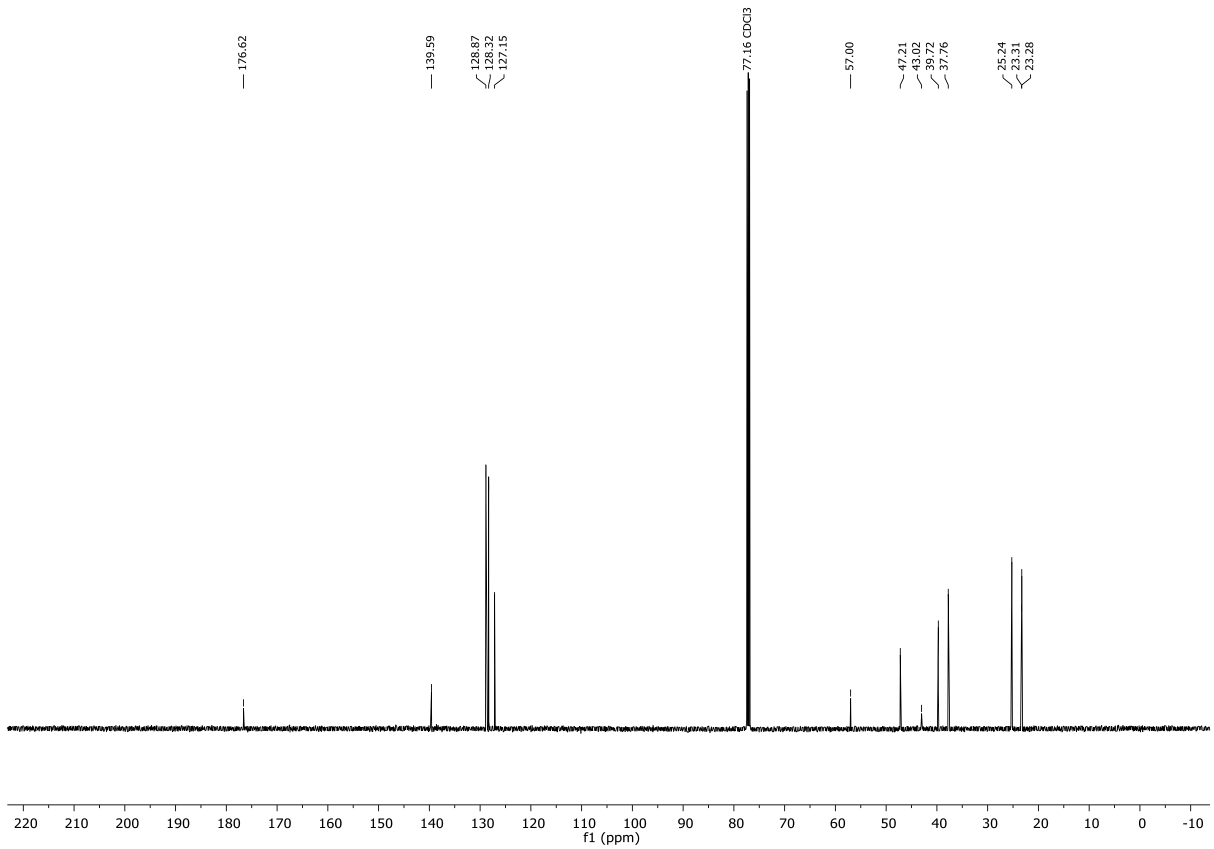

^{1}H NMR (500 MHz, CDCl$_3$)

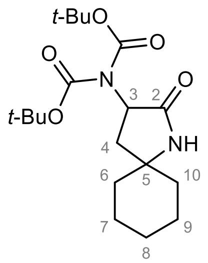

23c

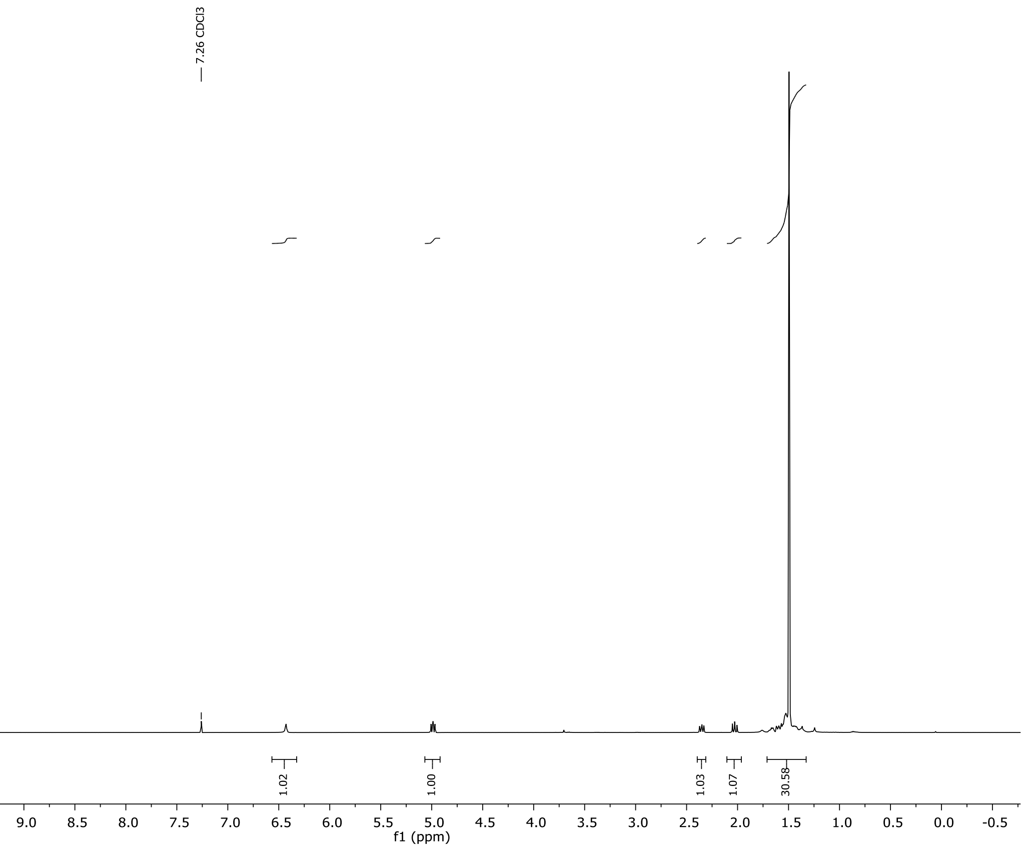

^{13}C{^{1}H} NMR (126 MHz, CDCl$_3$)

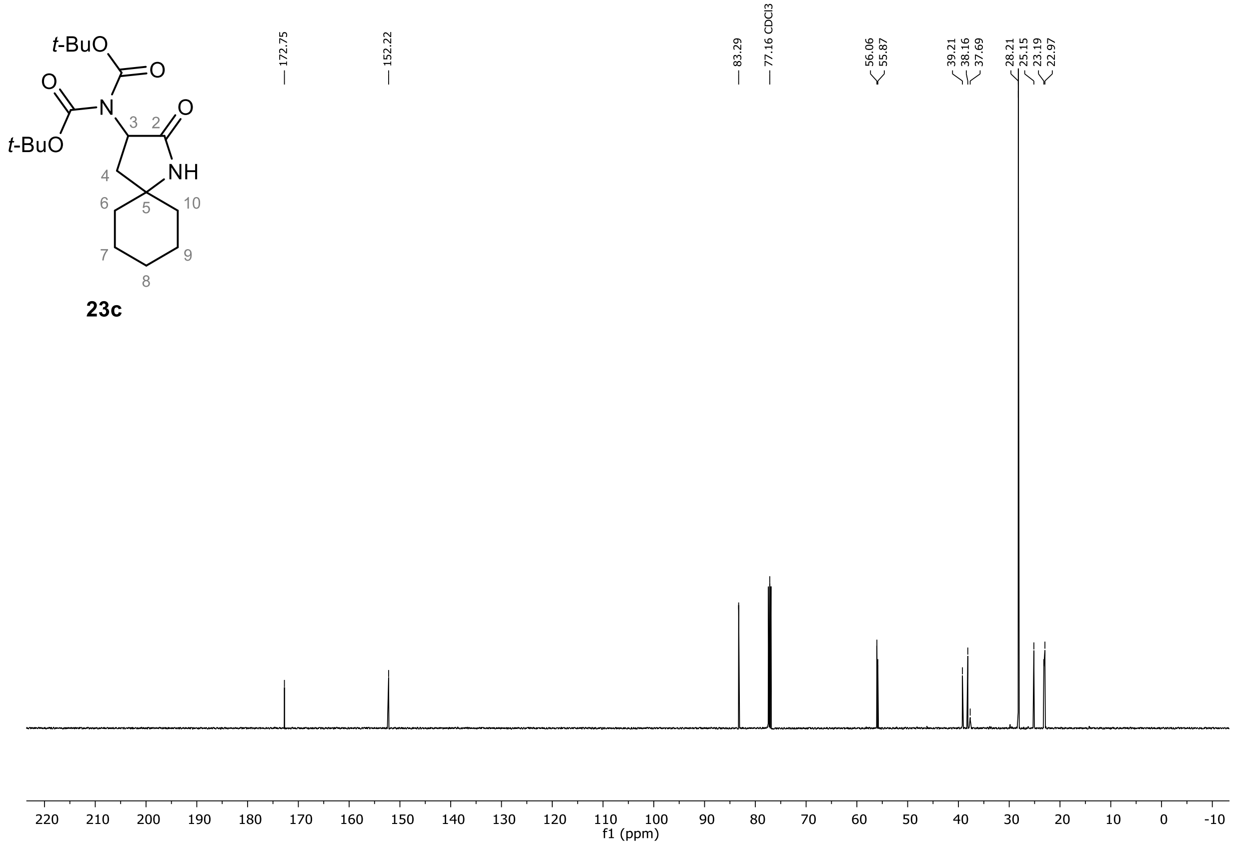

23c

^{1}H NMR (500 MHz, $CDCl_3$)

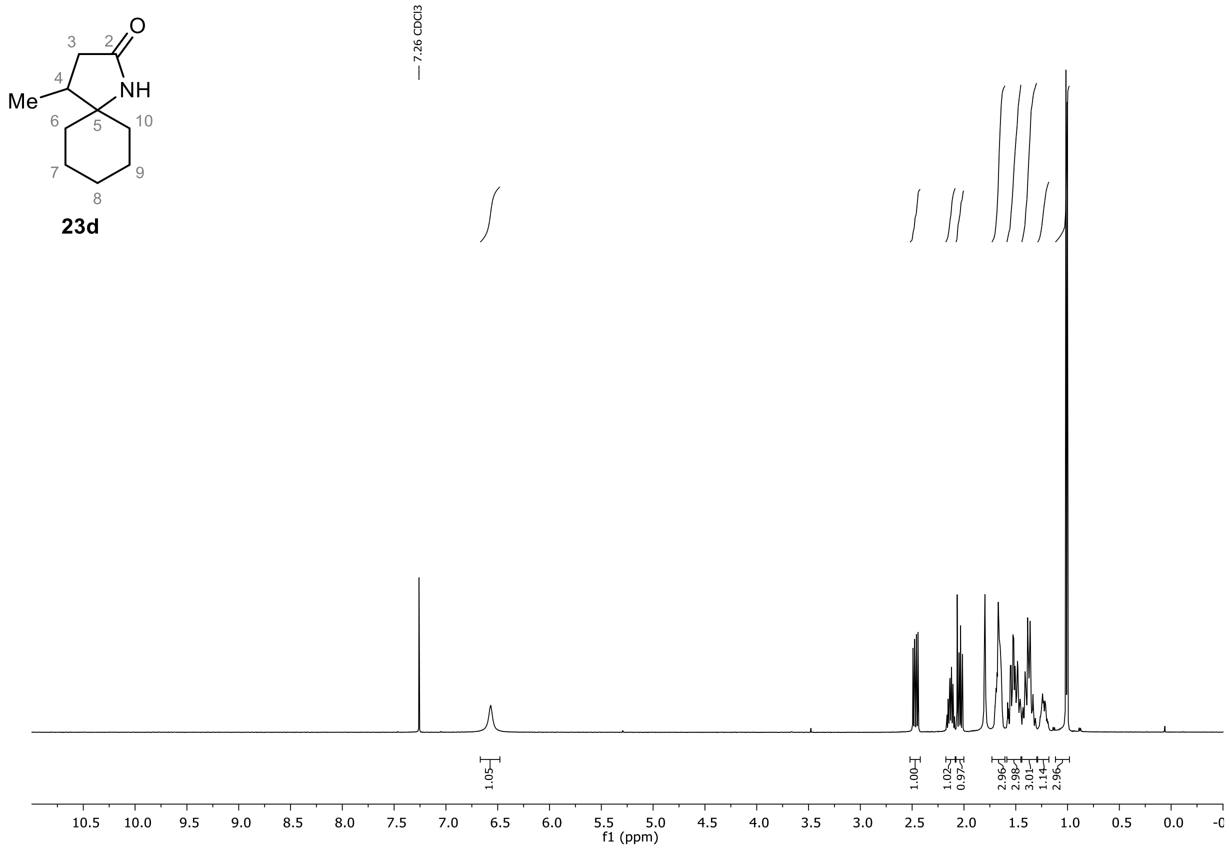

23d

$^{13}C\{^{1}H\}$ NMR (126 MHz, $CDCl_3$)

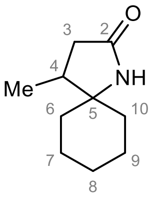
O
NH
Me
3
2
4
5
6
7
8
9
10

23d

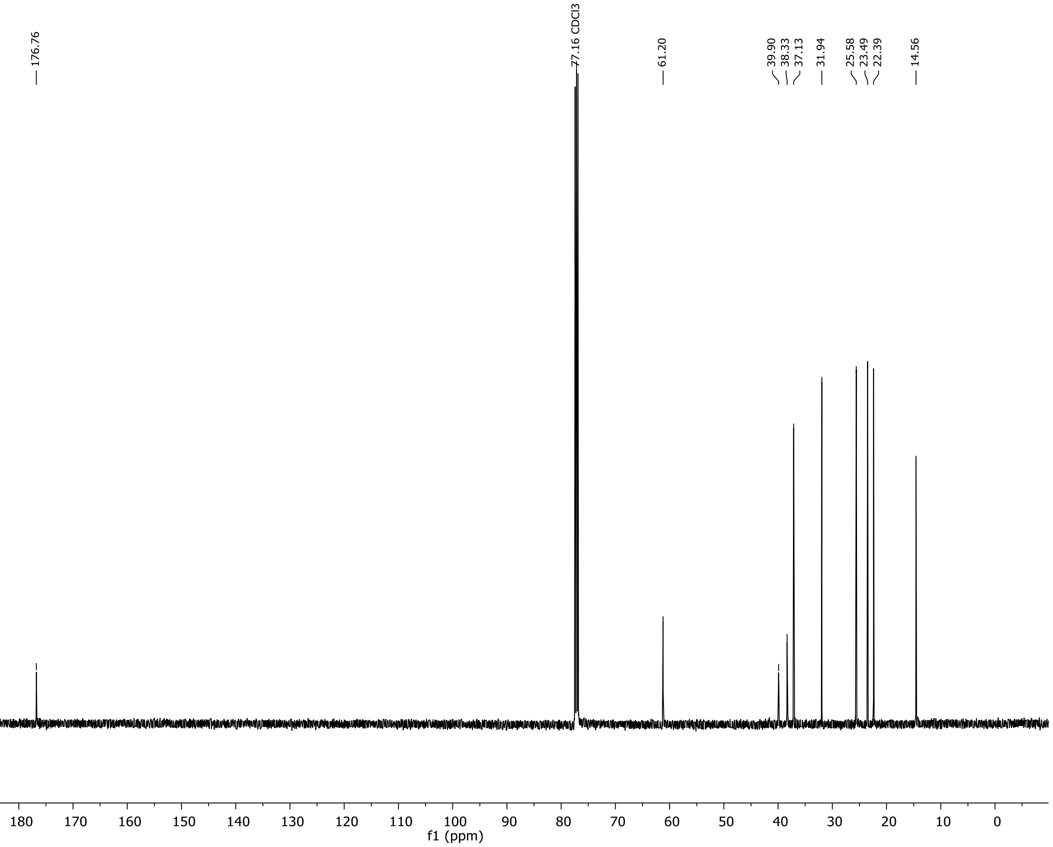
176.76
77.16 CDCl3
61.20
39.90
38.33
37.13
31.94
25.58
23.49
22.39
14.56
200
190
180
170
160
150
140
130
120
110
100
90
80
70
60
50
40
30
20
10
0
f1 (ppm)

^{1}H NMR (500 MHz, $CDCl_3$)

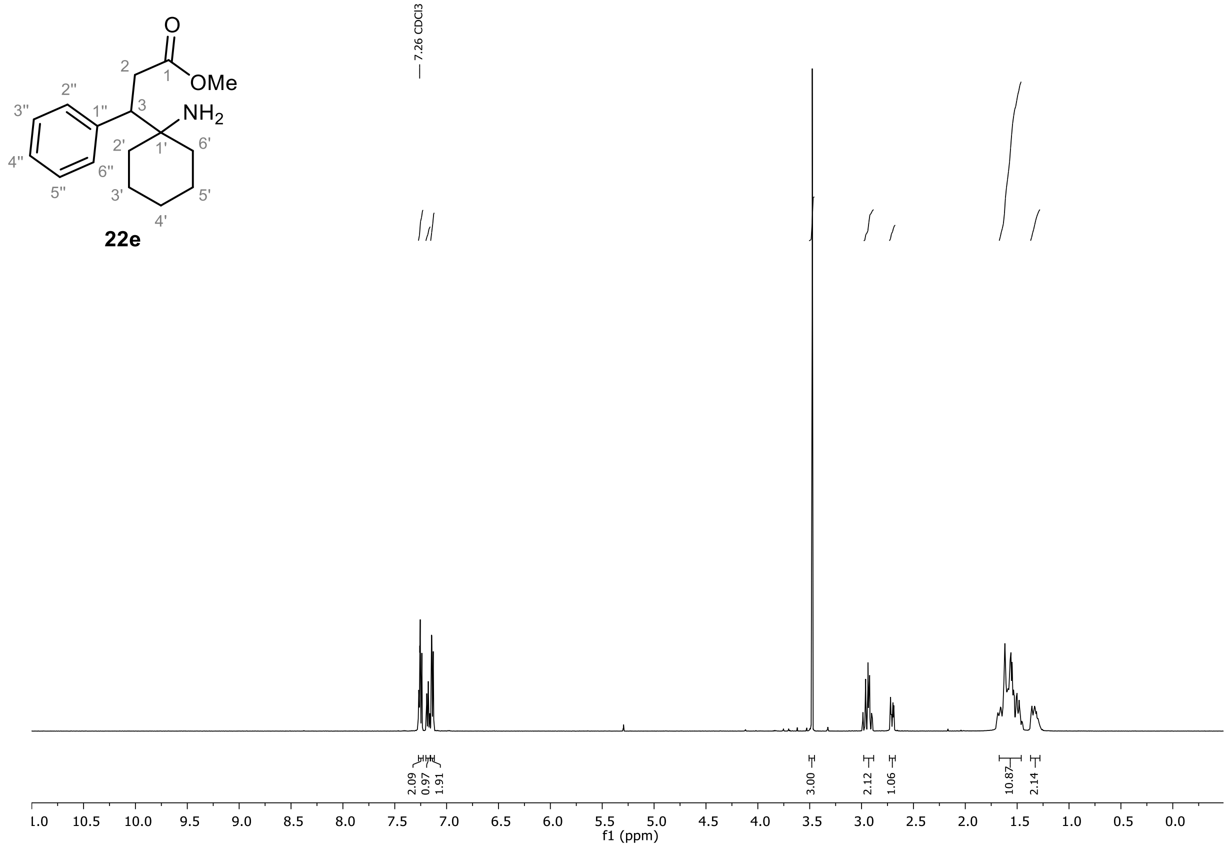

^{13}C{^{1}H} NMR (126 MHz, $CDCl_3$)

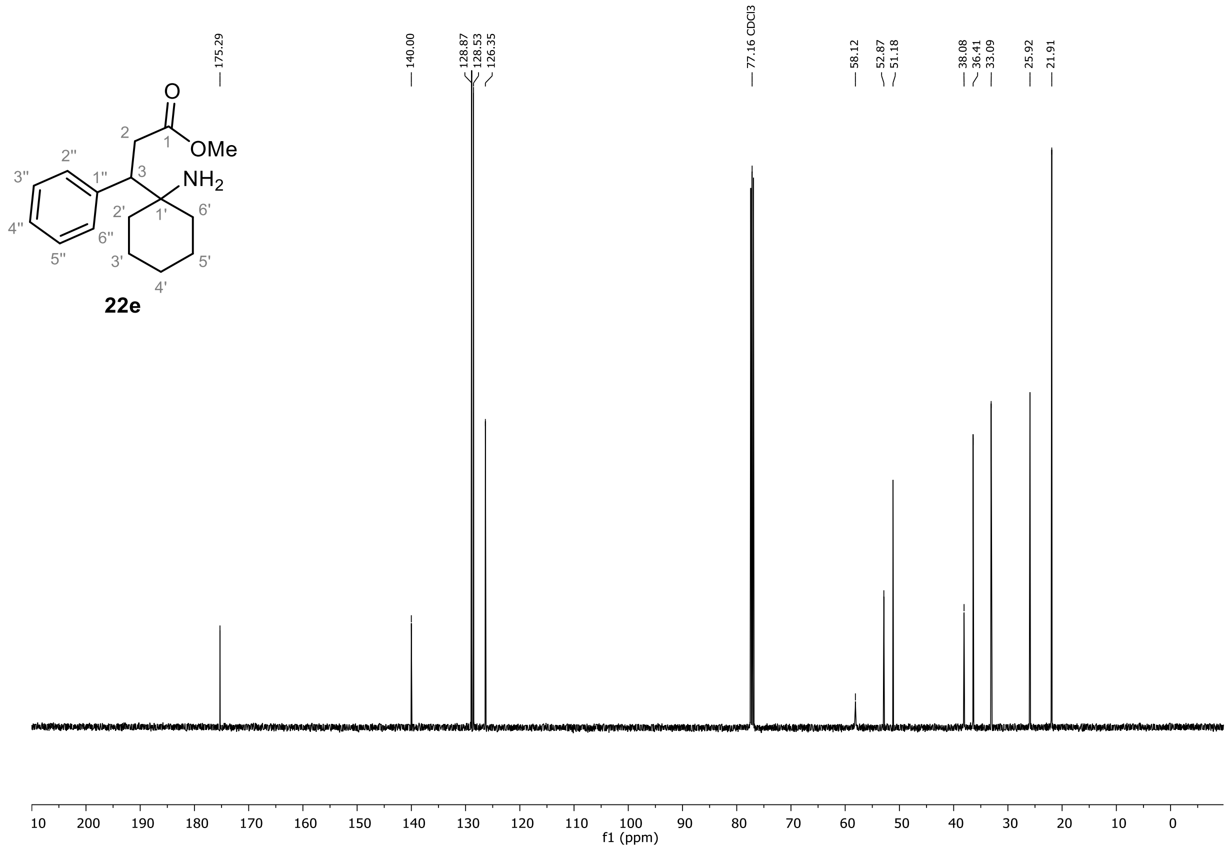

^{1}H NMR (500 MHz, MeOH-d_4)

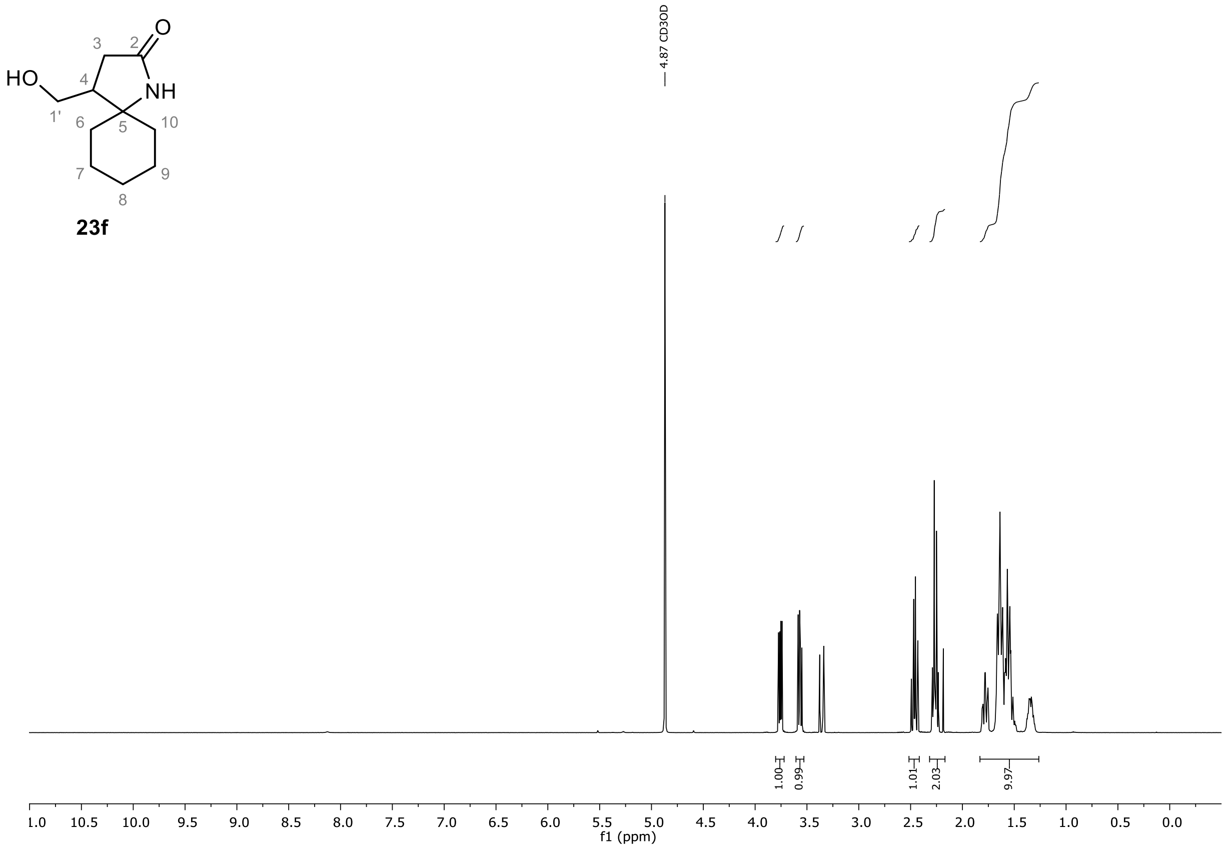

^{13}C{^{1}H} NMR (126 MHz, MeOH-d_4)

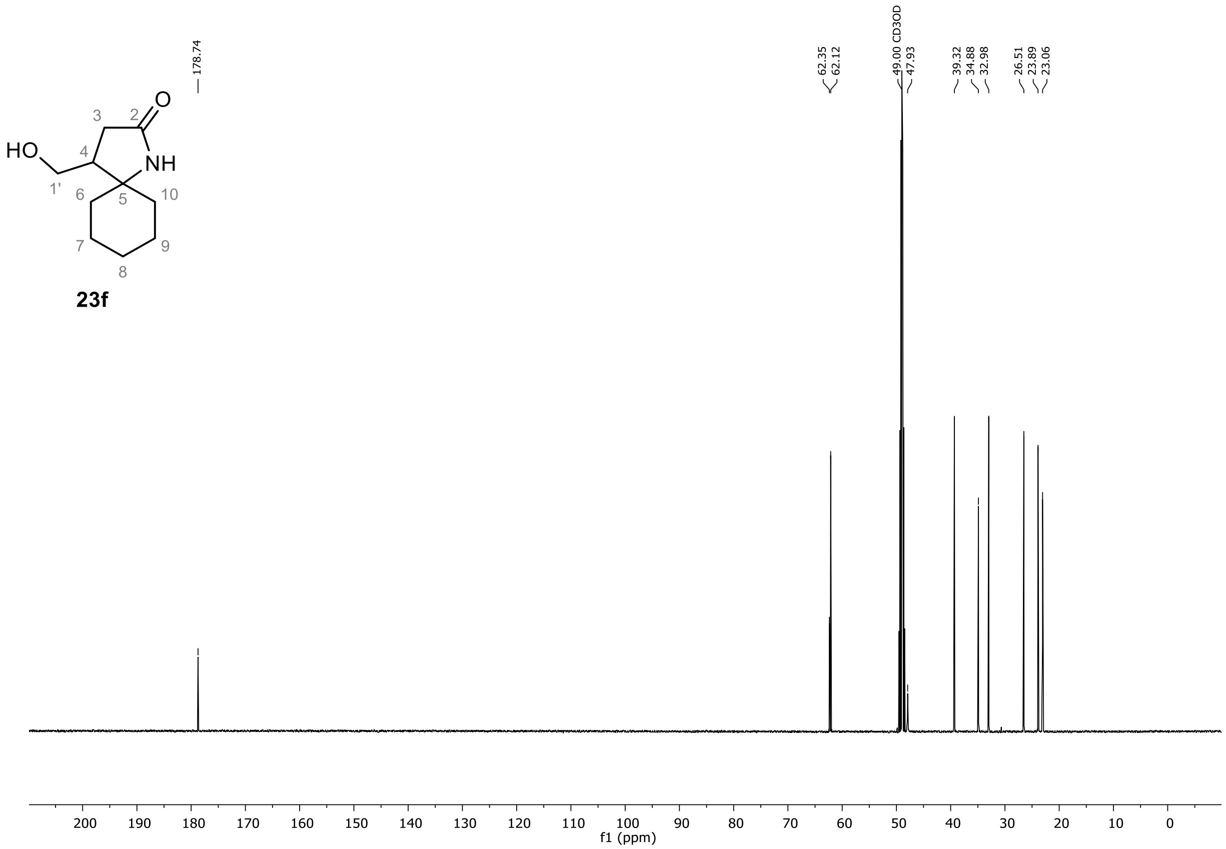

^{1}H NMR (500 MHz, CDCl$_3$)

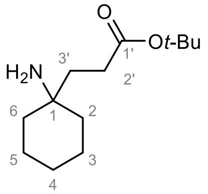

22g

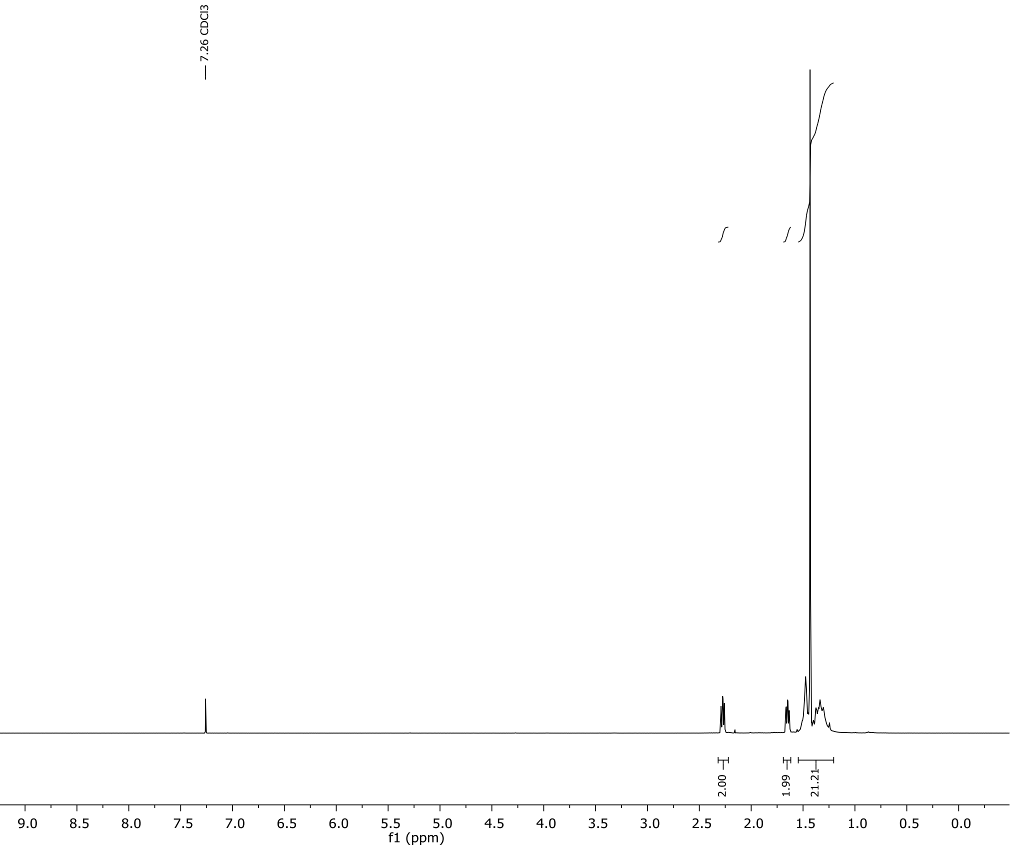

^{13}C{^{1}H} NMR (126 MHz, CDCl$_3$)

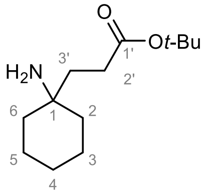

22g

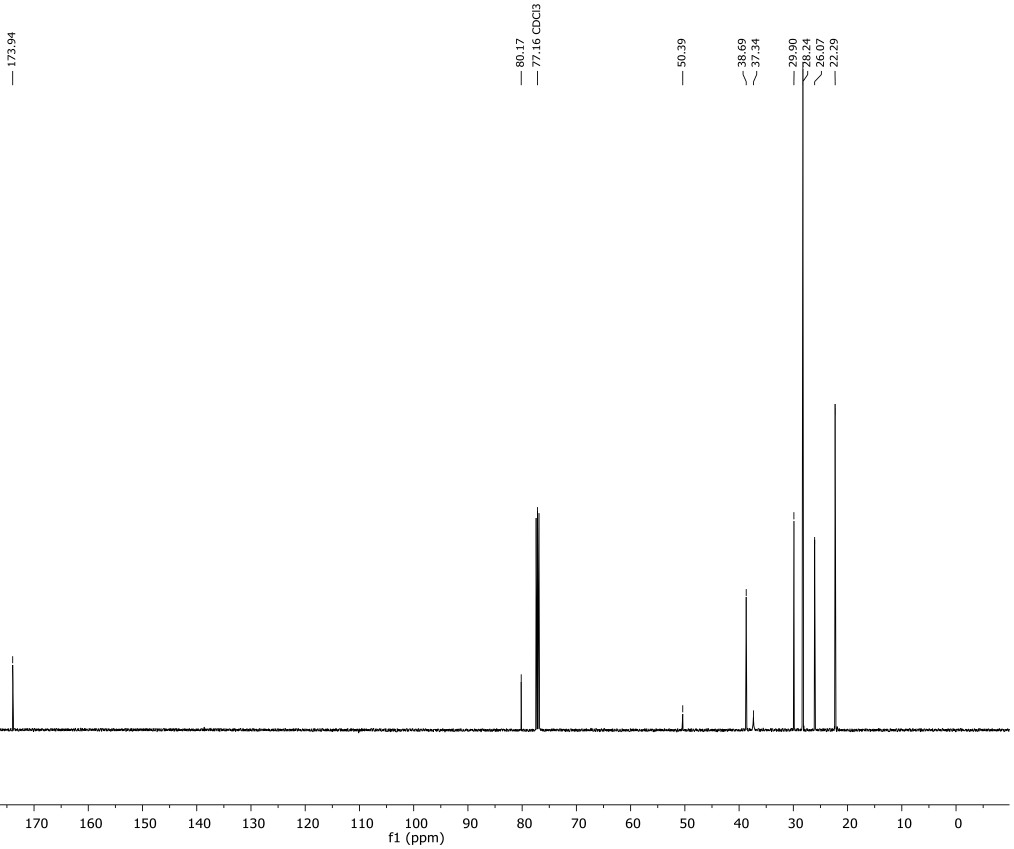

^{1}H NMR (500 MHz, $CDCl_3$)

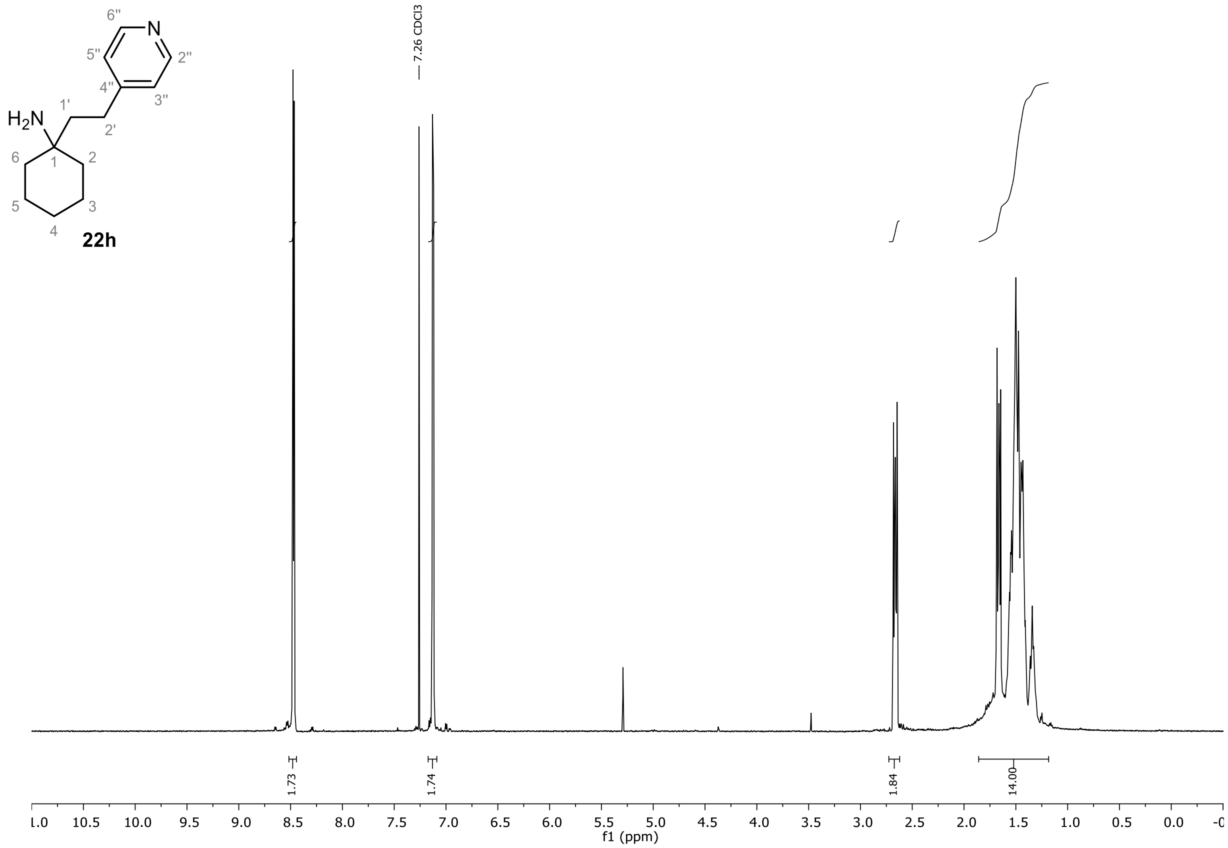

^{13}C{^{1}H} NMR (126 MHz, $CDCl_3$)

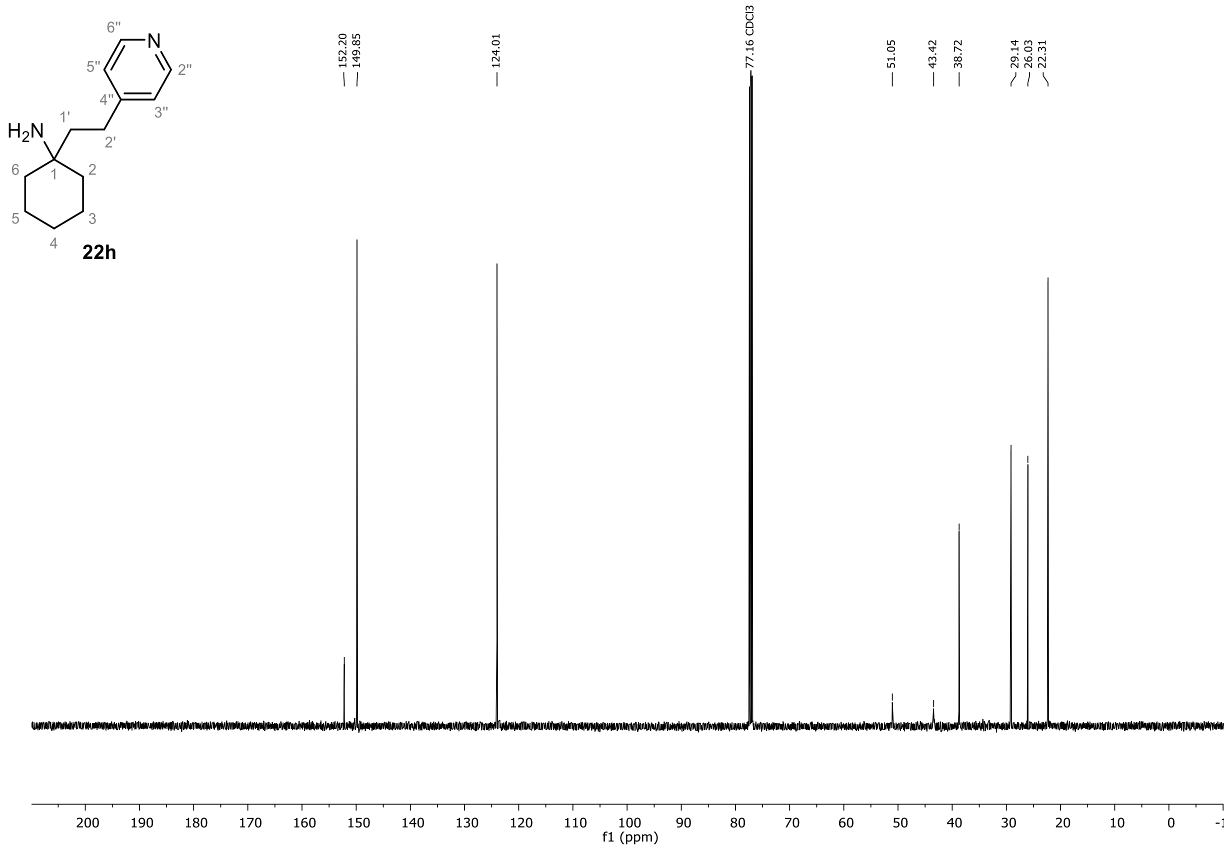

^{1}H NMR (500 MHz, $CDCl_3$)

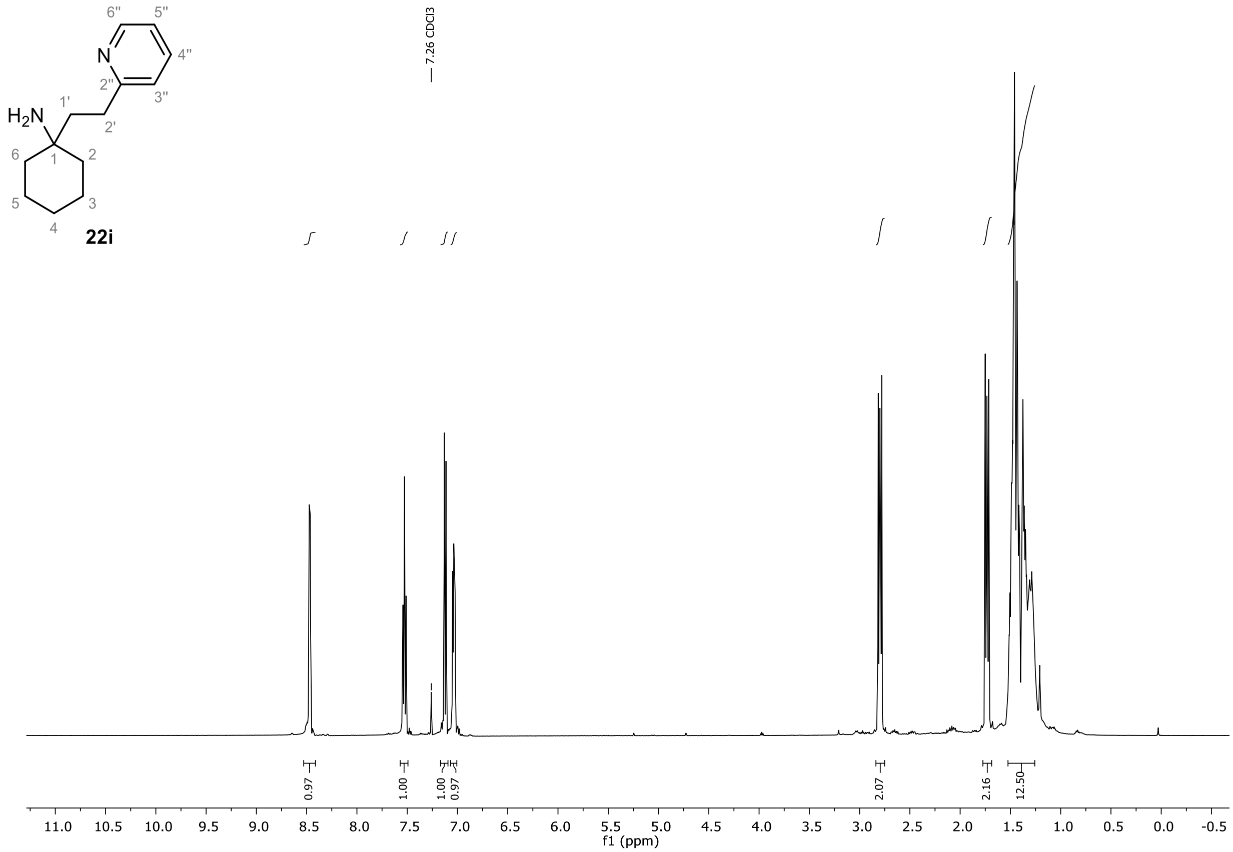

^{13}C{^{1}H} NMR (126 MHz, $CDCl_3$)

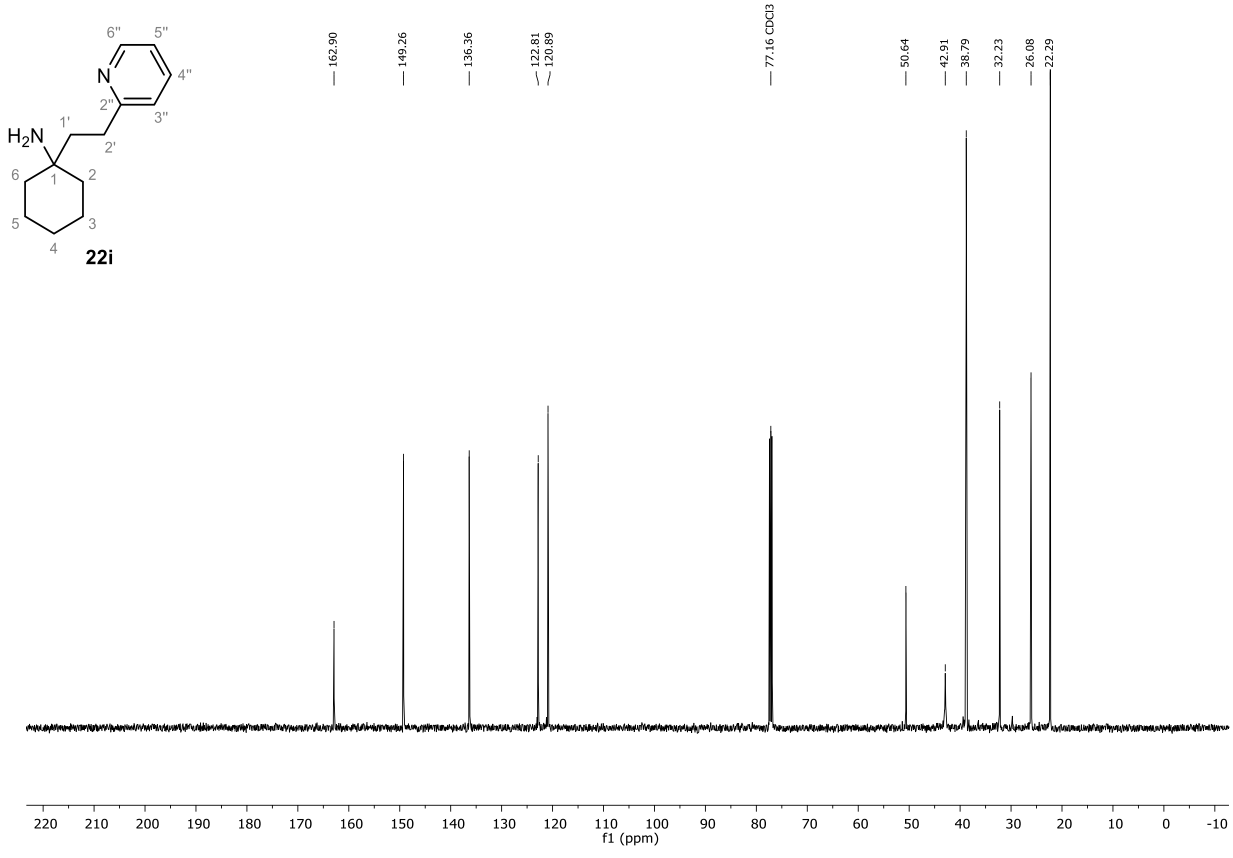

^{1}H NMR (500 MHz, CDCl$_3$)

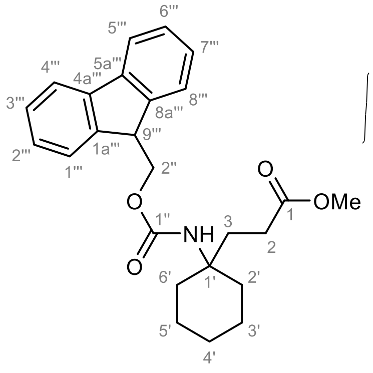

24a

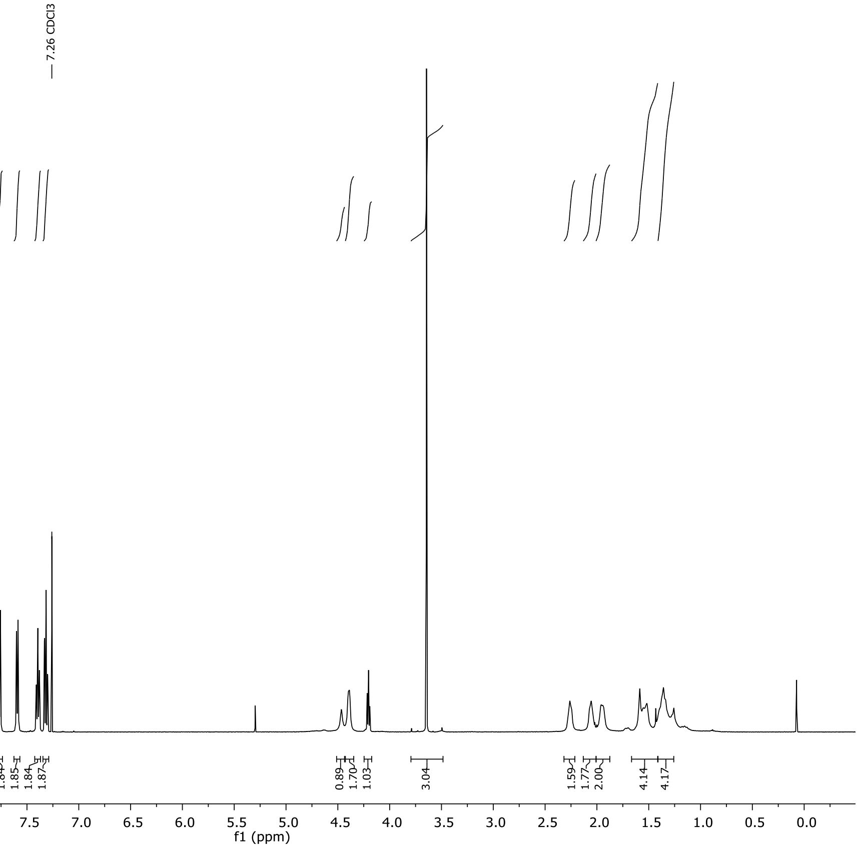

^{13}C{^{1}H} NMR (126 MHz, CDCl$_3$)

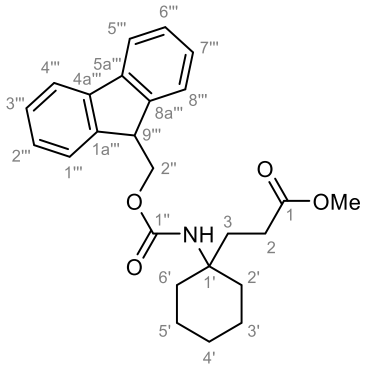

24a

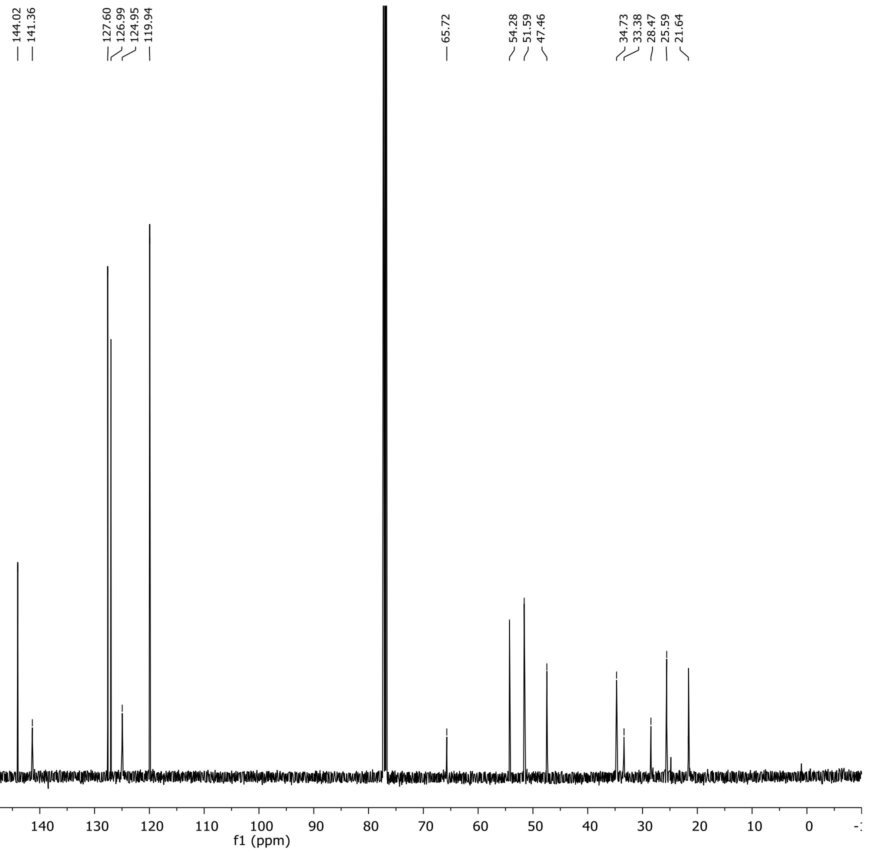

^{1}H NMR (500 MHz, $CDCl_3$)

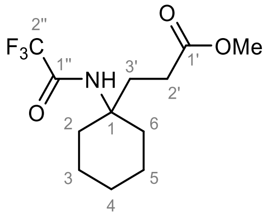

24b

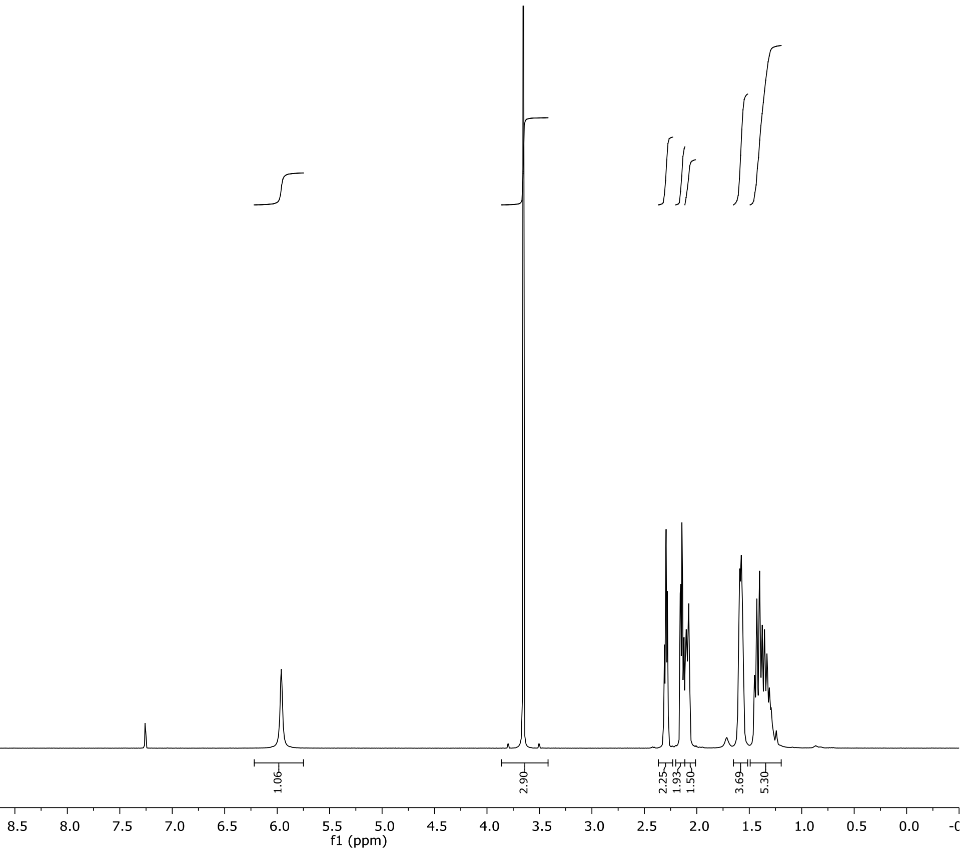

$^{13}C\{^{1}H\}$ NMR (126 MHz, $CDCl_3$)

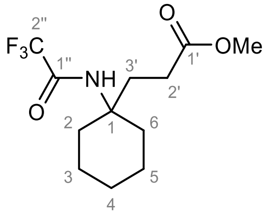

24b

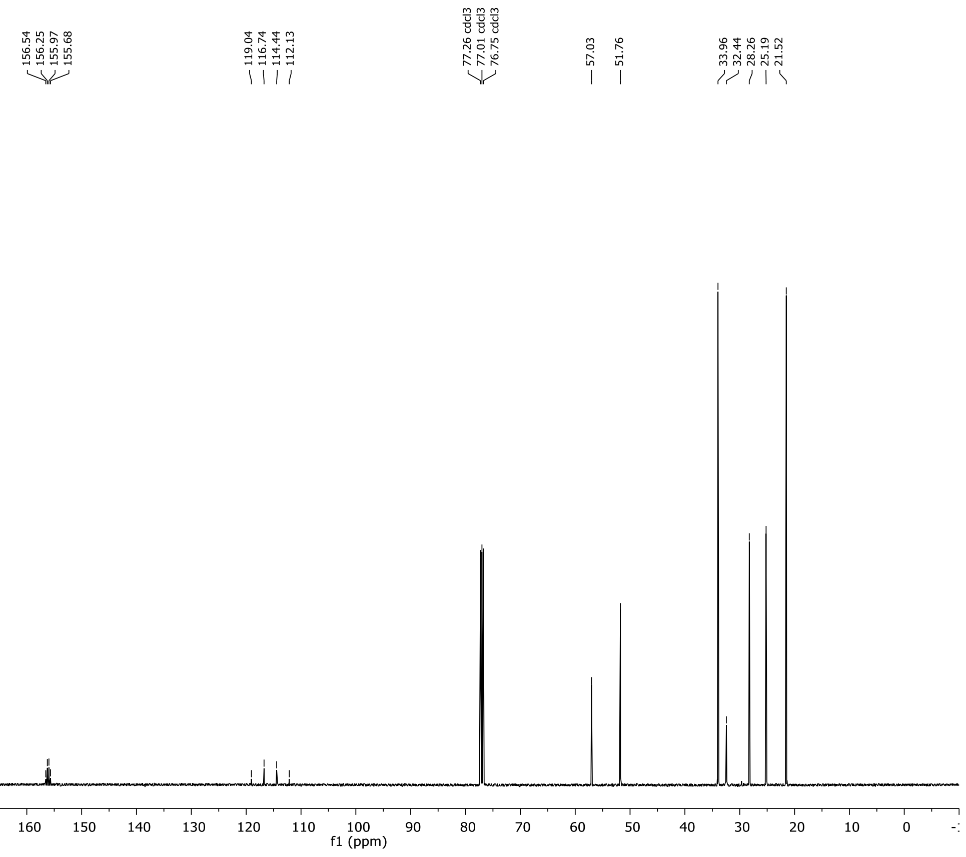

^{1}H NMR (500 MHz, $CDCl_3$)

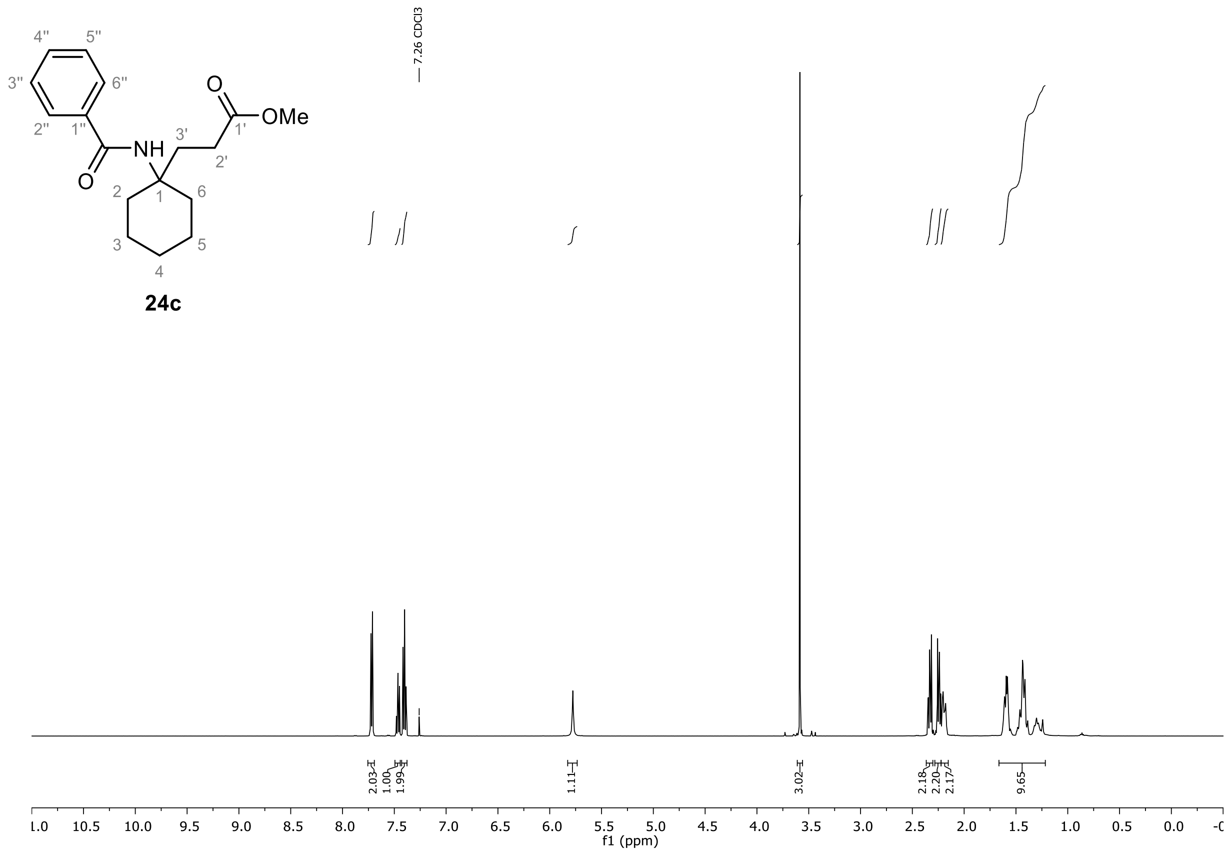

^{13}C{^{1}H} NMR (126 MHz, $CDCl_3$)

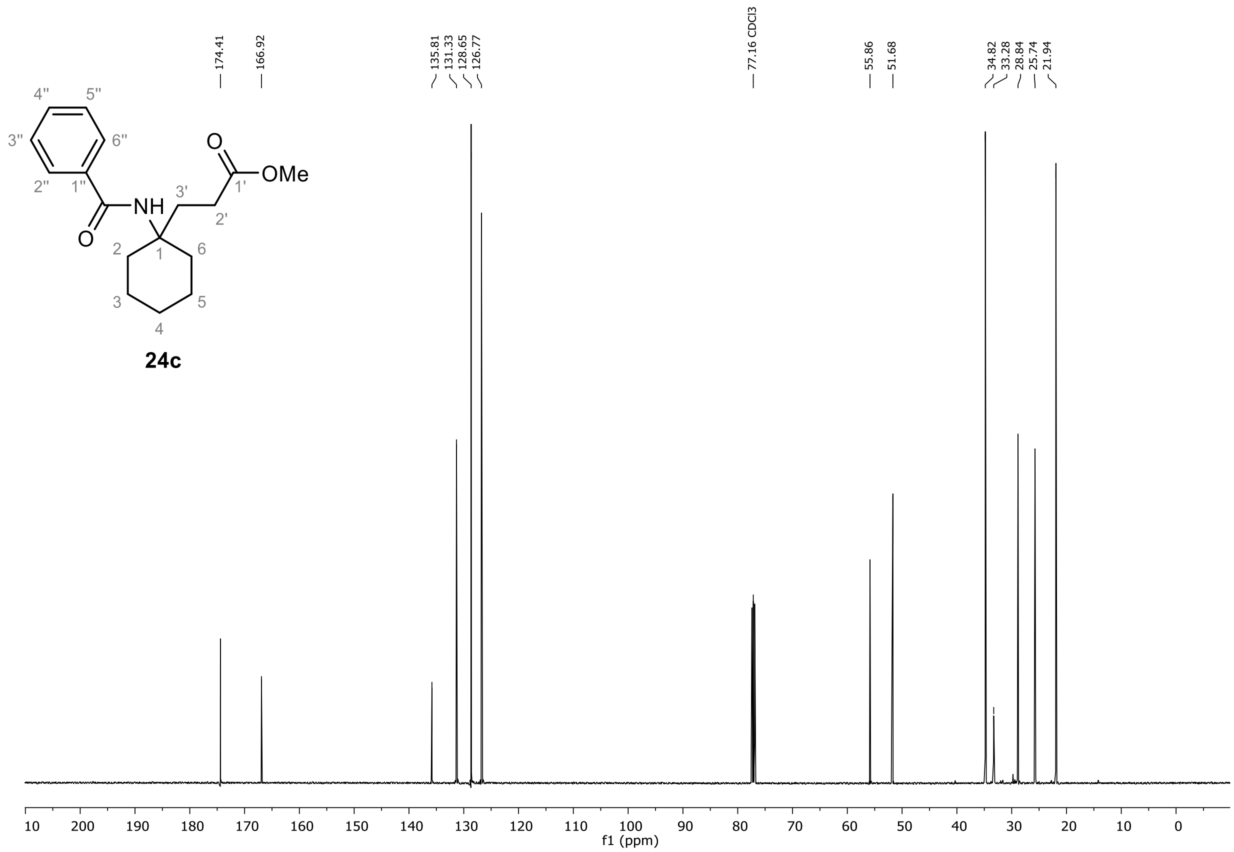

^{1}H NMR (500 MHz, CDCl$_3$)

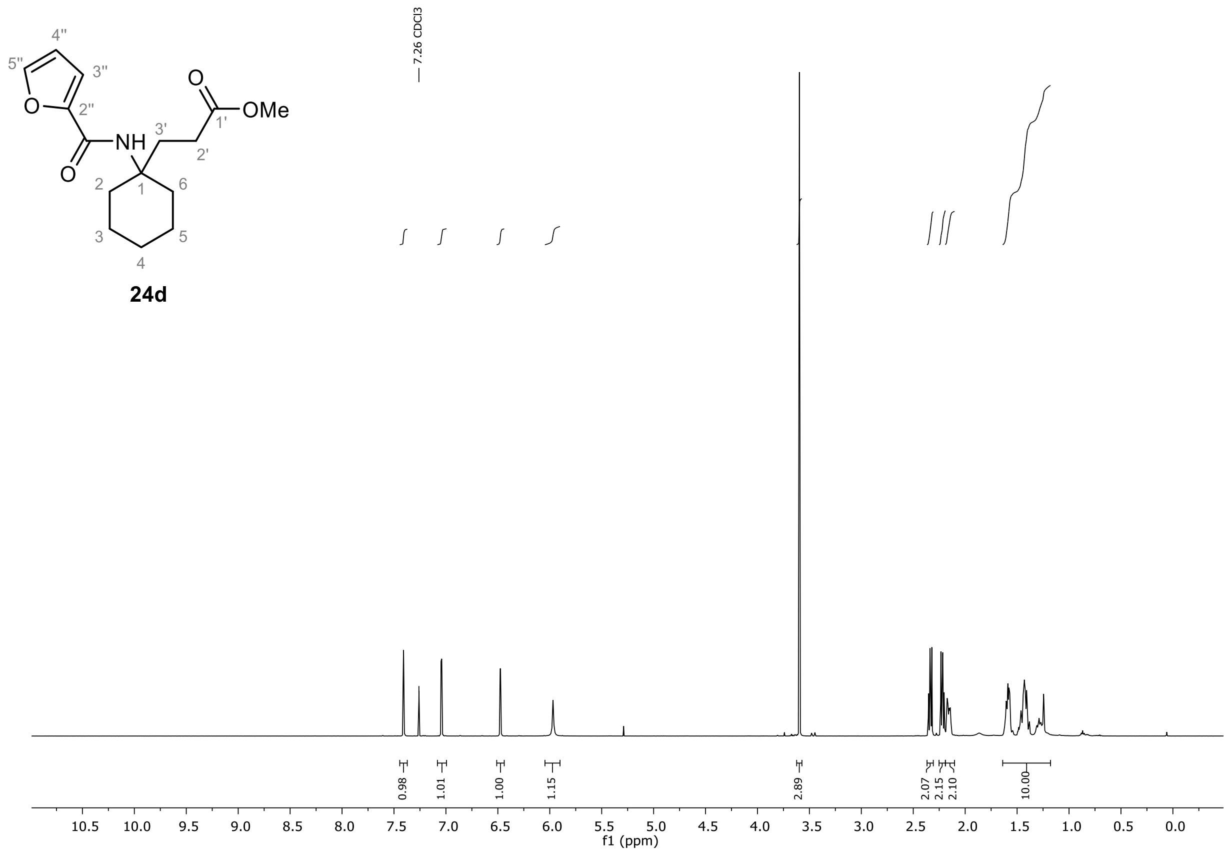

24d

^{13}C{^{1}H} NMR (126 MHz, CDCl$_3$)

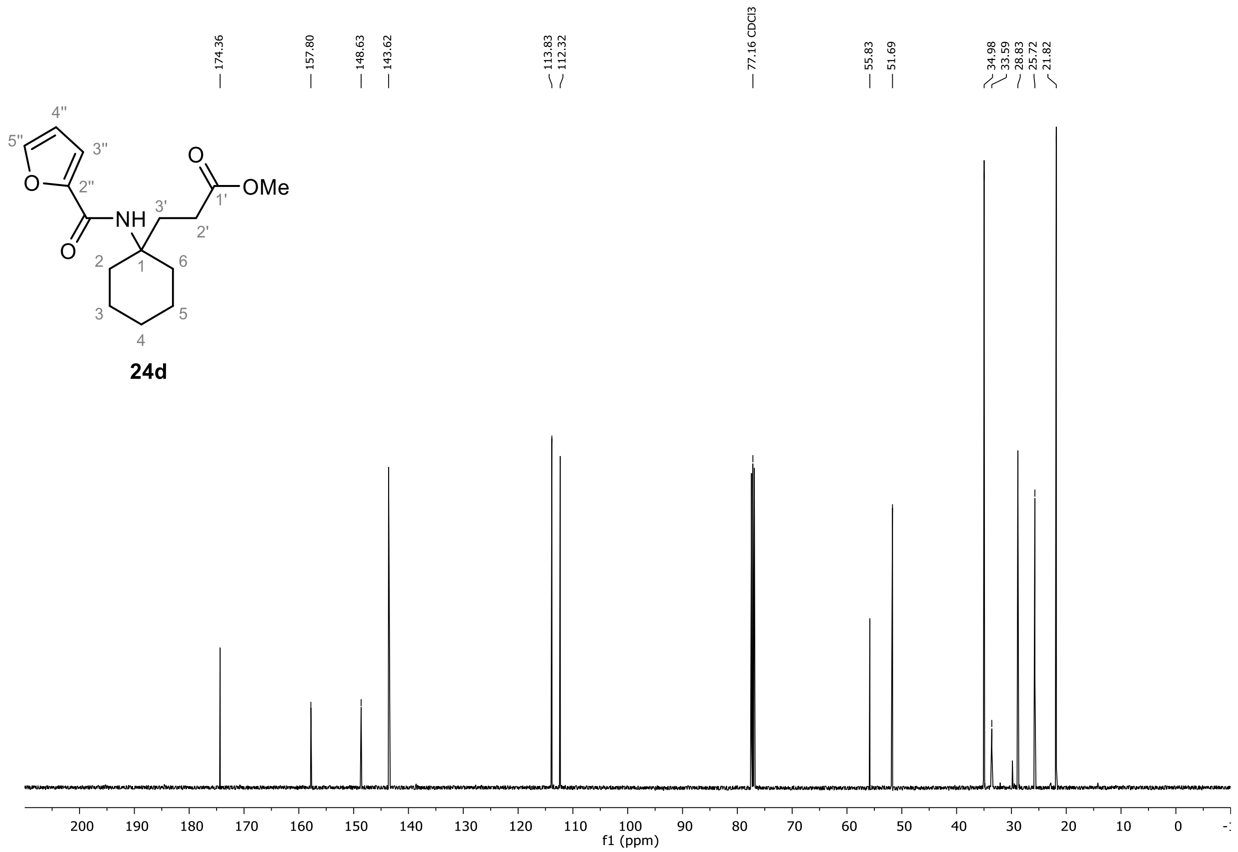

24d

^{1}H NMR (500 MHz, $CDCl_3$)

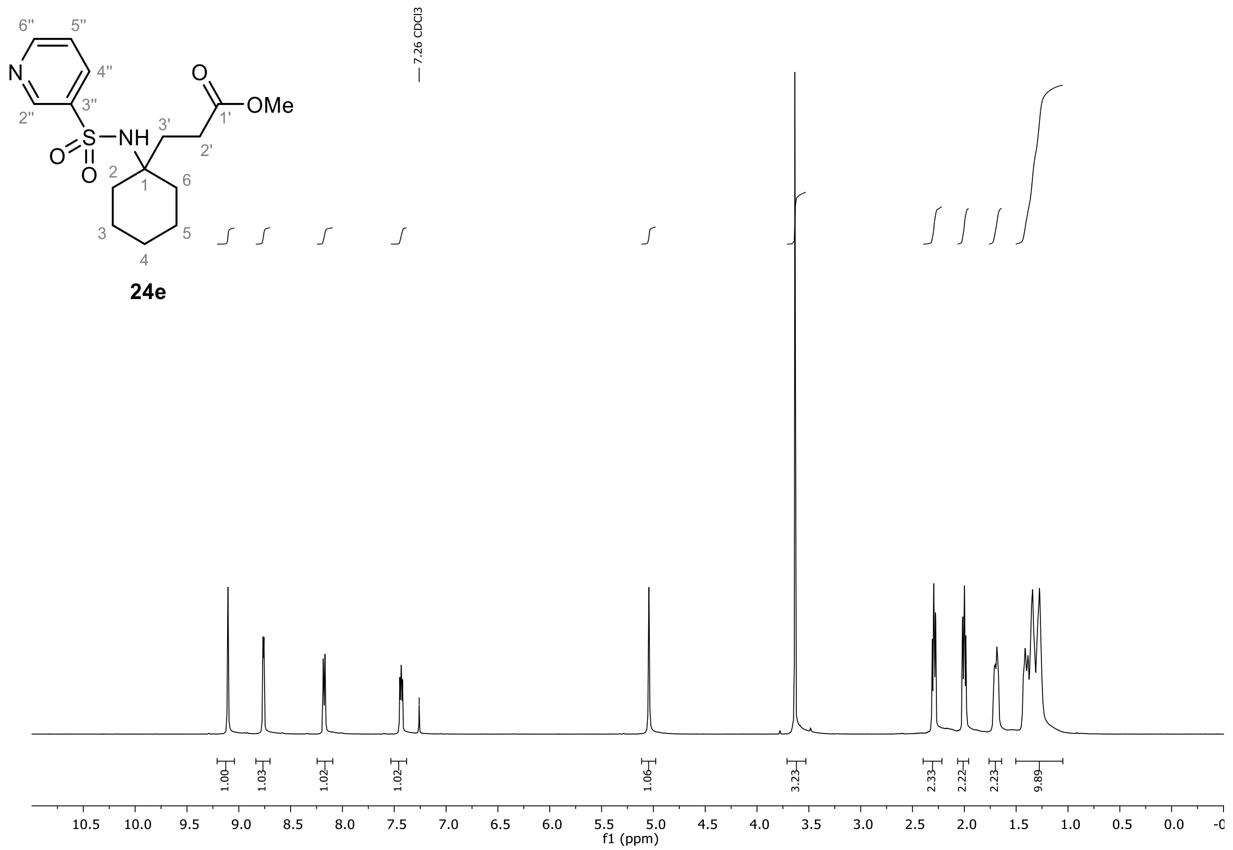

$^{13}C\{^1H\}$ NMR (126 MHz, $CDCl_3$)

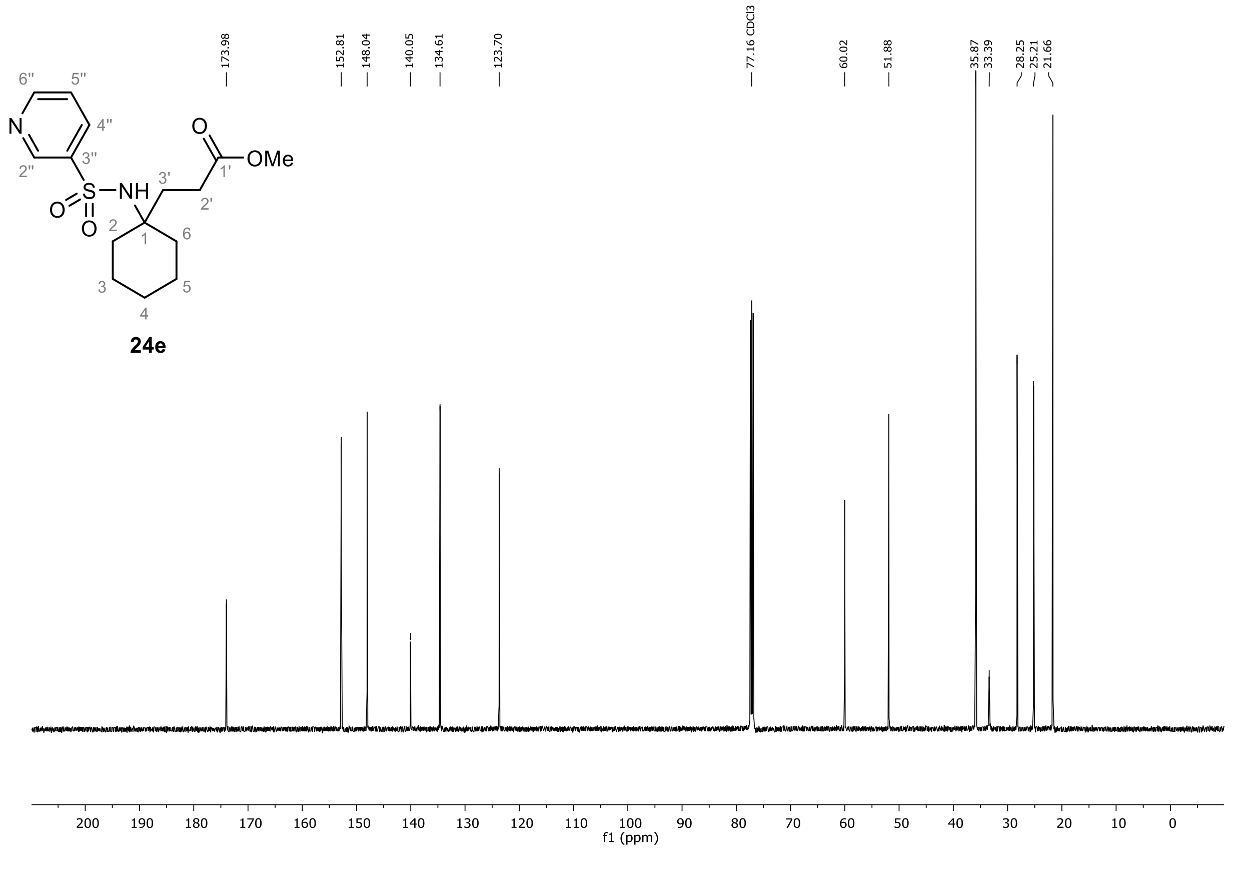

^{1}H NMR (500 MHz, $CDCl_3$)

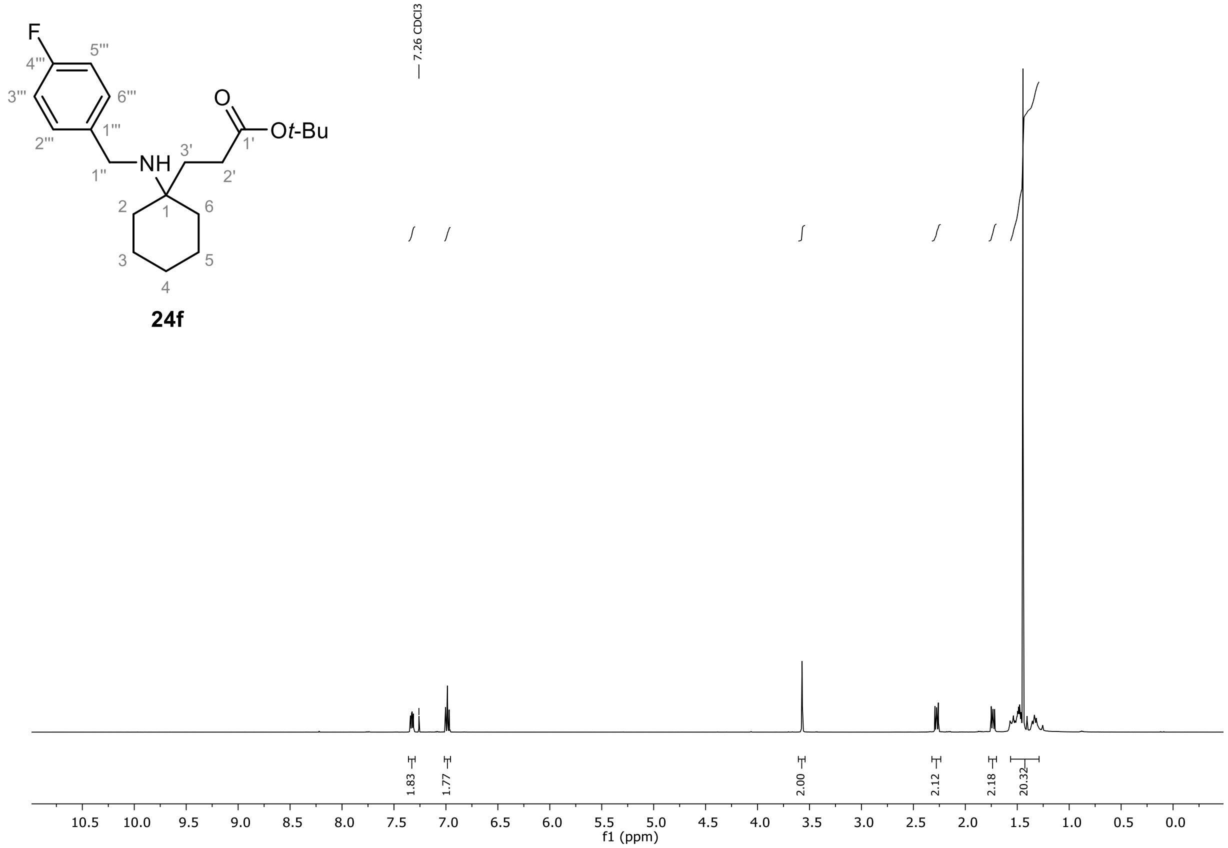

^{13}C{^{1}H} NMR (126 MHz, $CDCl_3$)

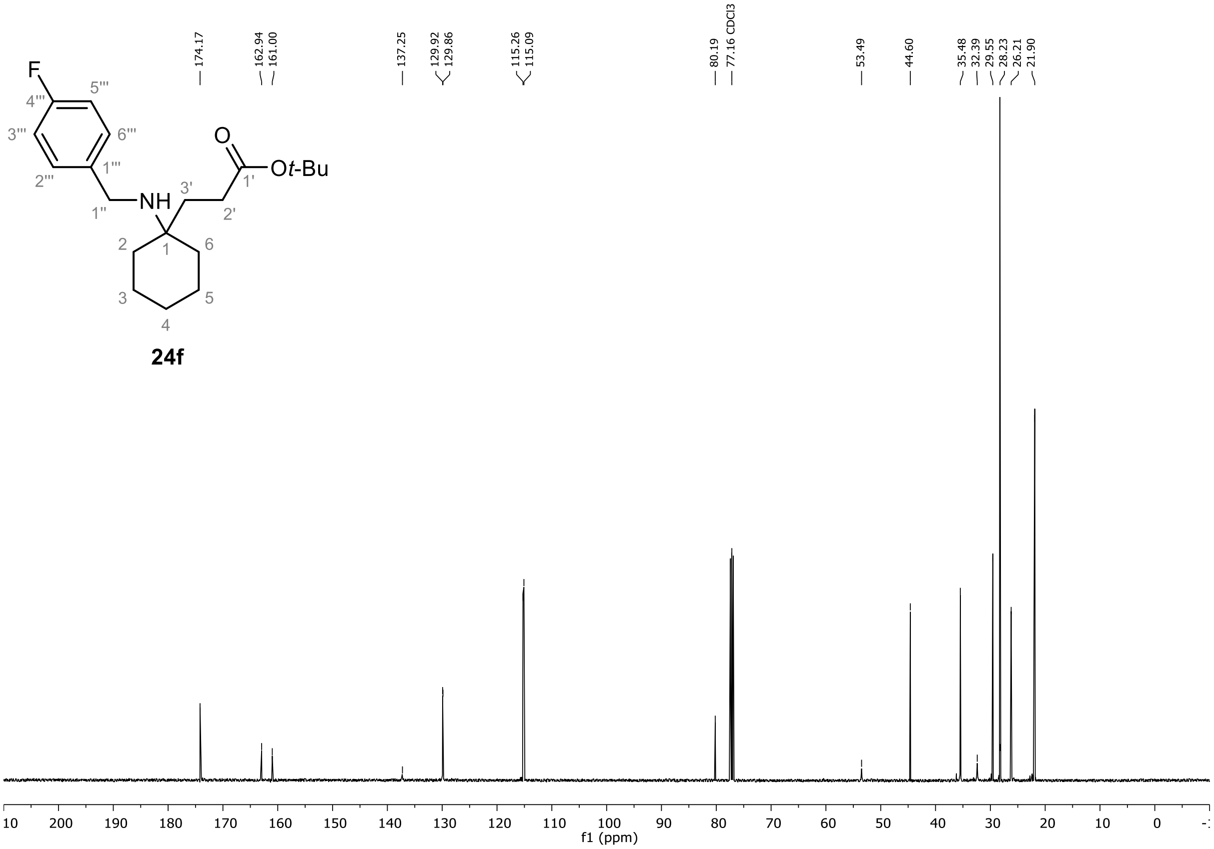

J. References and Notes

[1] A. M. R. Hall, J. C. Chouler, A. Codina, P. T. Gierth, J. P. Lowe, U. Hintermair, Practical aspects of real-time reaction monitoring using multi-nuclear high resolution FlowNMR spectroscopy. *Catal. Sci. Tech.* **2016**, *6*, 8406-8417.

[2] R. Hommelsheim, K. J. Hock, C. Schumacher, M. A. Hussein, T. V. Nguyen, R. M. Koenigs, Cyanomethyl anion transfer reagents for diastereoselective Corey–Chaykovsky cyclopropanation reactions. *Chem. Commun.* **2018**, *54*, 11439–11442.

[3] T. Rossolini, B. Ferko, D. J. Dixon, Photocatalytic Reductive Formation of α-Tertiary Ethers from Ketals. *Org. Lett.* **2019**, *21*, 6668–6673.

[4] M. Garreau, F. Le Vaillant, J. Waser, C-Terminal bioconjugation of peptides through photoredox catalyzed decarboxylative alkynylation. *Angew. Chem. Int. Ed.* **2019**, *58*, 8182–8186.

[5] X. Ma, H. Lubin, E. Ioja, O. Kékesi, A. Simon, Á. Apáti, T. I. Orbán, L. Héja, J. Kardos, I. E. Markó, Straightforward and effective synthesis of γ-aminobutyric acid transporter subtype 2-selective acyl-substituted azaspiro[4.5]decanes. *Bioorg. Med. Chem. Lett.* **2016**, *26*, 417–423.

[6] S. Das, J. S. Dileep Kumar, K. George Thomas, K. Shivaramayya, M. V. J. George, Photocatalyzed multiple additions of amines to α,β-unsaturated esters and nitriles. *J. Org. Chem. Soc.* **1994**, *59*, 628–634.

[7] F. Medina, C. Michon, F. Agbossou-Niedercorn, Intermolecular mono- and dihydroamination of activated alkenes using a recoverable gold catalyst. *Eur. J. Org. Chem.* **2012**, 6218–6227.

[8] G. Bosica, J. Spiteri, C. Borg, Aza-Michael reaction: selective mono- versus bis-addition under environmentally-friendly conditions. *Tetrahedron* **2014**, *70*, 2449–2454.

[9] N. Armanino, E. M. Carreira, Ruthenium-catalyzed intramolecular hydrocarbamoylation of allylic formamides: convenient access to chiral pyrrolidones. *J. Am. Chem. Soc.* **2013**, *135*, 6814–6817.

[10] P. Panchaud, C. Ollivier, P. Renaud, S. Zigmantas, Radical carboazidation: expedient assembly of the core structure of various alkaloid families. *J. Org. Chem.* **2004**, *69*, 2755–2759.

[11] M. Bertrand, A. Meou, A. Tubul, Cyclopropyliddènecyclanes oxydation par l'acide peroxycarboximidique. *Tetrahedron Lett.* **1982**, *23*, 3691–3694.

[12] E. Sinnreich, The light-induced addition of 2-pyrrolidone to olefins. *Tetrahedron* **1968**, *24*, 4509–4516.

[13] G. Zoidis, C. Fytas, I. Papanastasiou, G. B. Foscolos, G. Fytas, E. Padalko, E. De Clercq, L. Naesens, J. Neyts, N. Kolocouris, Heterocyclic rimantadine analogues with antiviral activity. *Bioorg. Med. Chem.* **2006**, *14*, 3341–3348.

[14] D. J. Wardrop, W. Zhang, *N*-Methoxy-*N*-acylnitrenium ions: application to the formal synthesis of (±)-desmethylamino FR901483. *Org. Lett.* **2001**, *3*, 2353–2356.

[15] J. Kang, H. Chung, S. Y. Kim, Y. Kim, J. Lee, N. E. Lewin, L. V. Pearce, P. M. Blumberg, V. E. Marquez, Conformationally constrained analogues of diacylglycerol (DAG). Effect on protein kinase C (PK-C) binding by the isosteric replacement of sn-1 and sn-2 esters in DAG-lactones. *Bioorg. Med. Chem.* **2003**, *11*, 2529–2539.

[16] T. J. Donohoe, C. K. A. Callens, A. L. Thompson, Tethered Aminohydroxylation (TA) Reaction of Amides. *Org. Lett.* **2009**, *11*, 2305–2307.

[17] K. L. Jensen, E. A. Standley, T. F. Jamison, Highly regioselective nickel-catalyzed cross-coupling of *N*-tosylaziridines and alkylzinc reagents. *J. Am. Chem. Soc.* **2014**, *136*, 11145–11152.

[18] M. Pfau, R. Dulou, Photochemical addition of amines to α,β-unsaturated esters. *Bull. Soc. Chim. Fr.* **1967**, 3336–3342.

[19] The residence is calculated using the relationship: *Residence time (min) = [photoreactor volume (mL)/total flow velocity (mL/min)]*. For example, given that the photoreactor volume is fixed at 10 mL, a total flow rate of 0.5 mL/min (i.e., 0.25 mL/min per reagent feed) gives a residence time of 20 min.

[20] The space-time yield (STY) for a continuous flow reaction is defined as the amount of product obtained per hour for one litre of reactor volume. Our flow reactor has an internal volume of 10 mL and a throughput of 0.32 mol L^{-1} h^{-1} (for compound **17b**).

[21] R. E. Conrow, W. D. Dean, Diazidomethane explosion. *Org. Proc. Res. Dev.* **2008**, *12*, 1285–1286.

[22] K. Kiyokawa, R. Ito, K. Takemotoa, S. Minakata, *Chem. Commun.* **2018**, *54*, 7609–7612.

[23] H. G. Roth, N. A. Romero, D. A. Nicewicz, Experimental and calculated electrochemical potentials of common organic molecules for applications to single-electron redox chemistry. *Synlett* **2016**, *27*, 714–723.

[24] M. Montalti, A. Credi, L. Prodi, M. T. Gandolfi, Handbook of Photochemistry, 3rd ed. (CRC/Taylor & Francis, Boca Raton, FL, 2006).

[25] M. A. Cismesia, T. P. Yoon, Characterizing chain processes in visible light photoredox catalysis. *Chem. Sci.* **2015**, *6*, 5426–5434.

[26] These calculations treat the LED light as monochromatic, but in reality there is a spread of wavelengths. This is inconsequential because the distribution of wavelengths is Gaussian, so the average wavelength is precisely centred on 425 nm.

[27] https://www.omnicalculator.com/physics/photon-energy.

[28] Gaussian 16, Revision A.03, M. J. Frisch, G. W. Trucks, H. B. Schlegel, G. E. Scuseria, M. A. Robb, J. R. Cheeseman, G. Scalmani, V. Barone, G. A. Petersson, H. Nakatsuji, X. Li, M. Caricato, A. V. Marenich, J. Bloino, B. G. Janesko, R. Gomperts, B. Mennucci, H. P. Hratchian, J. V. Ortiz, A. F. Izmaylov, J. L. Sonnenberg, D. Williams-Young, F. Ding, F. Lipparini, F. Egidi, J. Goings, B. Peng, A. Petrone, T. Henderson, D. Ranasinghe, V. G. Zakrzewski, J. Gao, N. Rega, G. Zheng, W. Liang, M. Hada, M. Ehara, K. Toyota, R. Fukuda, J. Hasegawa, M. Ishida, T. Nakajima, Y. Honda, O. Kitao, H. Nakai, T. Vreven, K. Throssell, J. A. Montgomery, Jr., J. E. Peralta, F. Ogliaro, M. J. Bearpark, J. J. Heyd, E. N. Brothers, K. N. Kudin, V. N. Staroverov, T. A. Keith, R. Kobayashi, J. Normand, K. Raghavachari, A. P. Rendell, J. C. Burant, S. S. Iyengar, J. Tomasi, M. Cossi, J. M. Millam, M. Klene, C. Adamo, R. Cammi, J. W. Ochterski, R. L. Martin, K. Morokuma, O. Farkas, J. B. Foresman, D. J. Fox, Gaussian, Inc., Wallingford CT, 2016.

[29] L. Simón, J. M. Goodman, How Reliable Are DFT Transition Structures? Comparison of GGA, Hybrid-Meta-GGA and Meta-GGA Functionals. *Org. Biomol. Chem.* **2011**, *9*, 689–700.

[30] I. Funes-Ardoiz, R. S. Paton, GoodVibes: GoodVibes 2.0.2 (2016); http://doi.org/10.5281/Zenodo.595246.

[31] C. Y. Legault, CYLView, 1.0b; Université de Sherbrooke: Sherbrooke, Quebec, Canada (2009); http://www.cylview.org.
